# Addendum: Access to stereodefined (*Z*)-allylsilanes and (*Z*)-allylic alcohols via cobalt-catalyzed regioselective hydrosilylation of allenes

**DOI:** 10.1038/s41467-018-03605-1

**Published:** 2018-03-27

**Authors:** Chao Wang, Wei Jie Teo, Shaozhong Ge

**Affiliations:** Department of Chemistry, National University of Singapore, 3 Science Drive 3, Singapore, 117543 Singapore

**Keywords:** Catalytic mechanisms, Homogeneous catalysis, Organometallic chemistry, Synthetic chemistry methodology

**Addendum to**: *Nature Communications* 10.1038/s41467-017-02382-7, Published online 22 December 2017.

During the peer review of this Article, a related cobalt-catalyzed hydrosilylation of allenes was reported in Yang, Z., Peng, D., Du, X., Huang, Z. & Ma, S. Identifying a cobalt catalyst for highly selective hydrosilylation of allenes. *Org. Chem. Front*. **4**, 1829–1832 (2017).

