# Supplementary material for: Access to stereodefined (Z)-allylsilanes and (Z)-allylic alcohols via cobalt-catalyzed regioselective hydrosilylation of allenes
Source: Nat Commun. 2017 Dec 22;8:2258. doi: 10.1038/s41467-017-02382-7 (PMC5741631; doi:10.1038/s41467-017-02382-7)
Supplement: Supplementary file 1 — Supplementary Information [file 41467_2017_2382_MOESM1_ESM.pdf]

Supplementary Figures.

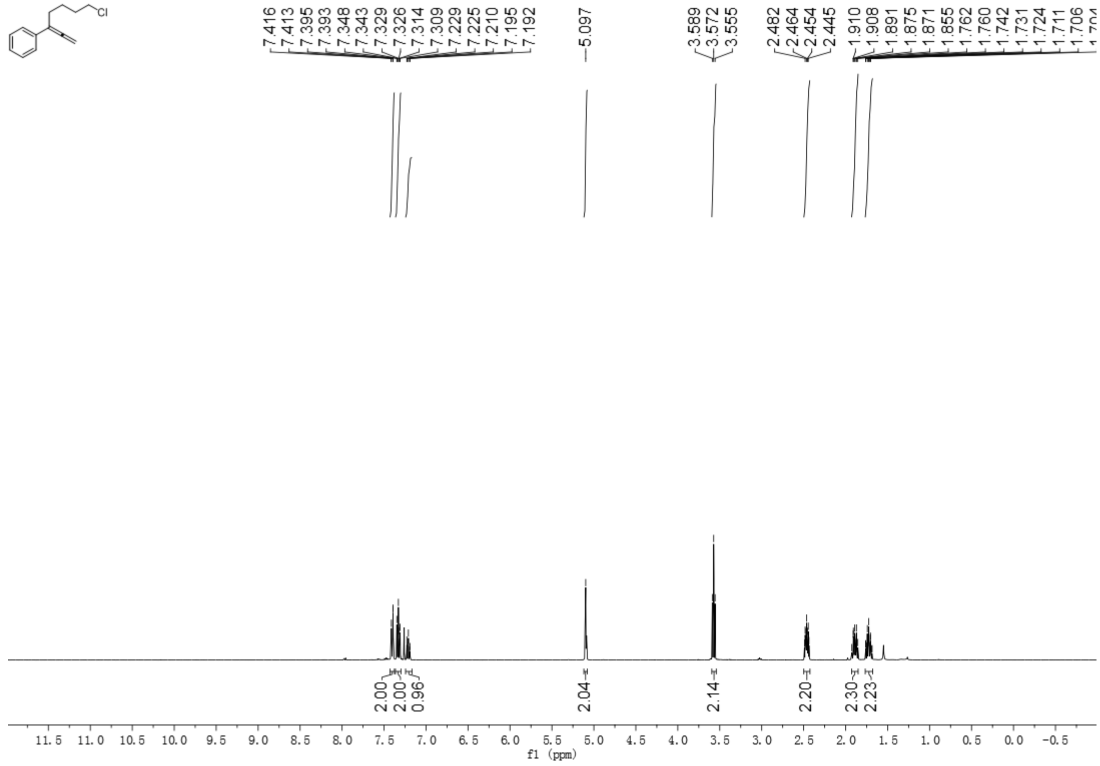

Supplementary Figure 1. ^{1}H NMR spectra for (7-chlorohepta-1,2-dien-3-yl)benzene

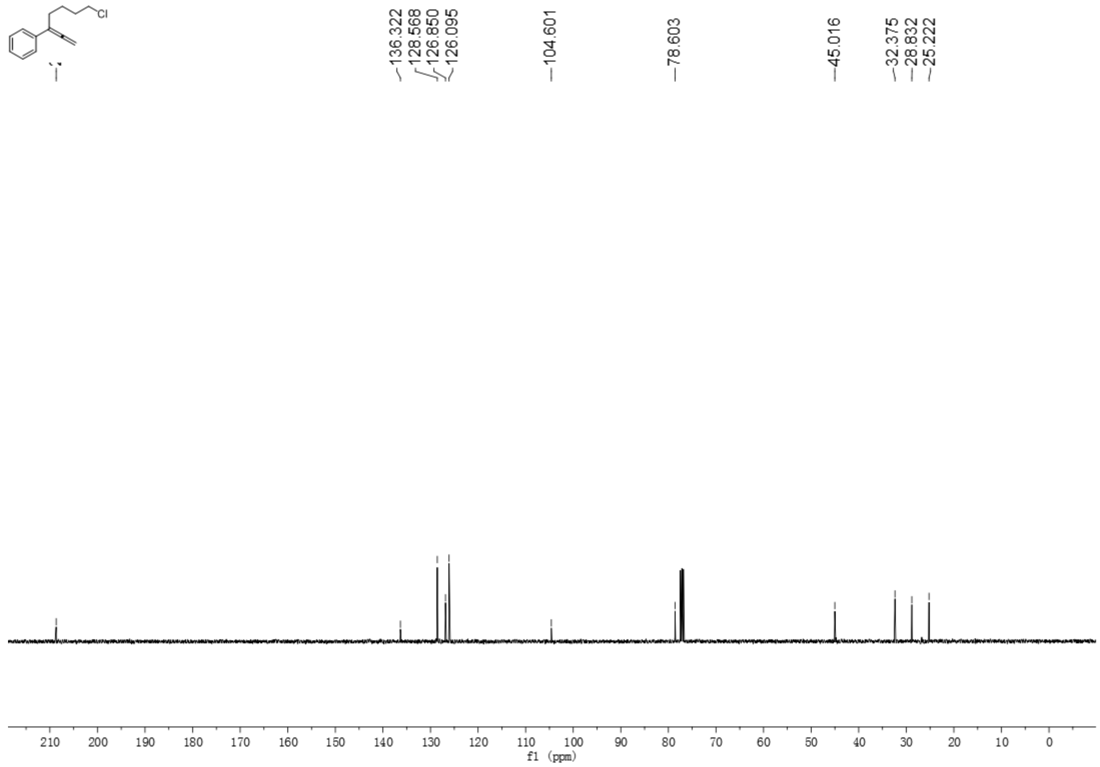

Supplementary Figure 2. ^{13}C NMR spectra for (7-chlorohepta-1,2-dien-3-yl)benzene

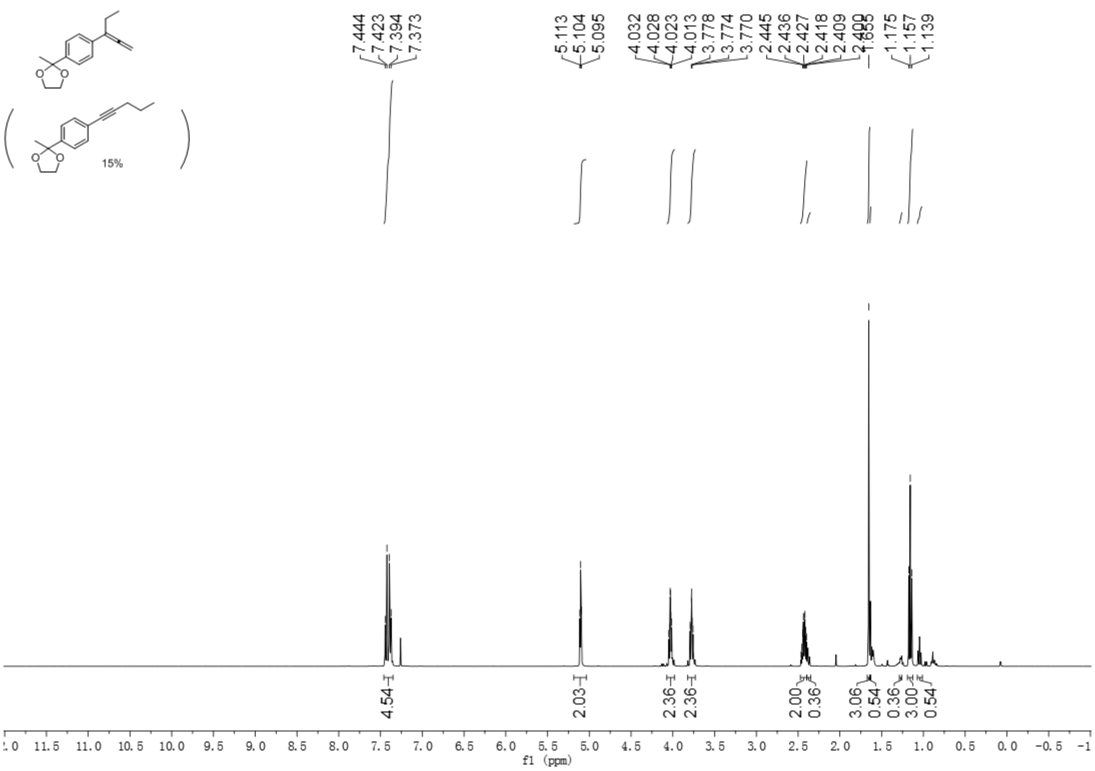

Supplementary Figure 3. ^{1}H NMR spectra for 2-methyl-2-(4-(penta-1,2-dien-3-yl)phenyl)-1,3-dioxolane

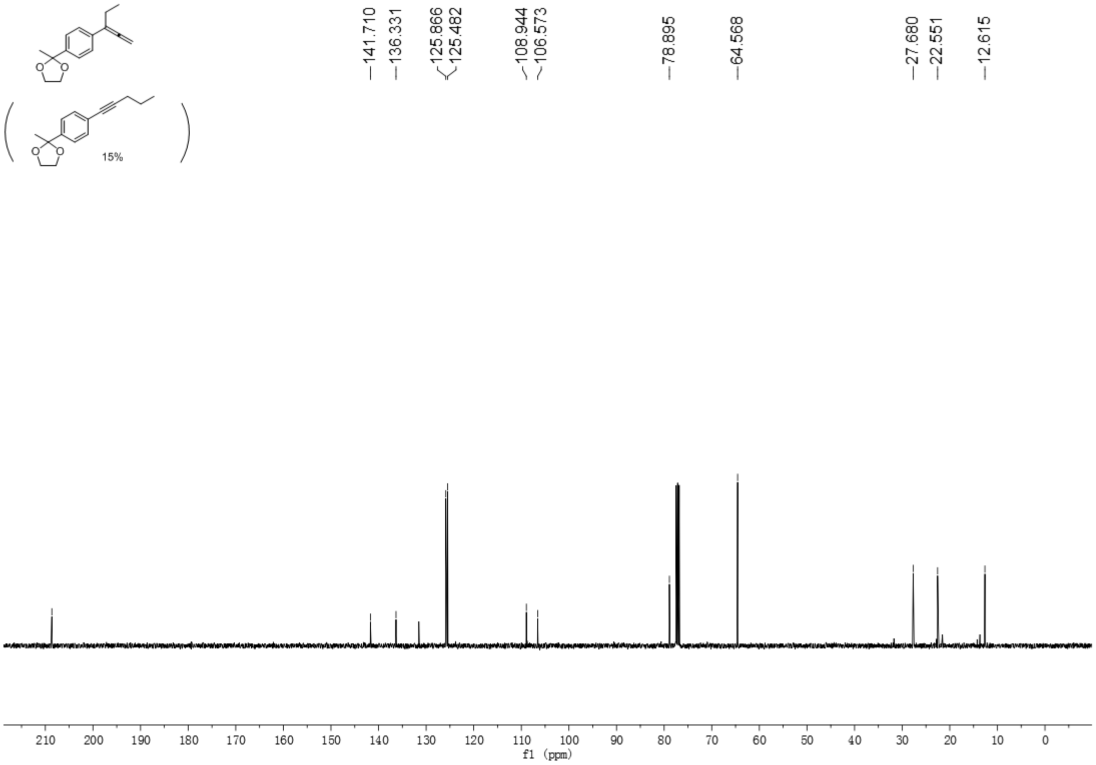

Supplementary Figure 4. ^{13}C NMR spectra for 2-methyl-2-(4-(penta-1,2-dien-3-yl)phenyl)-1,3-dioxolane

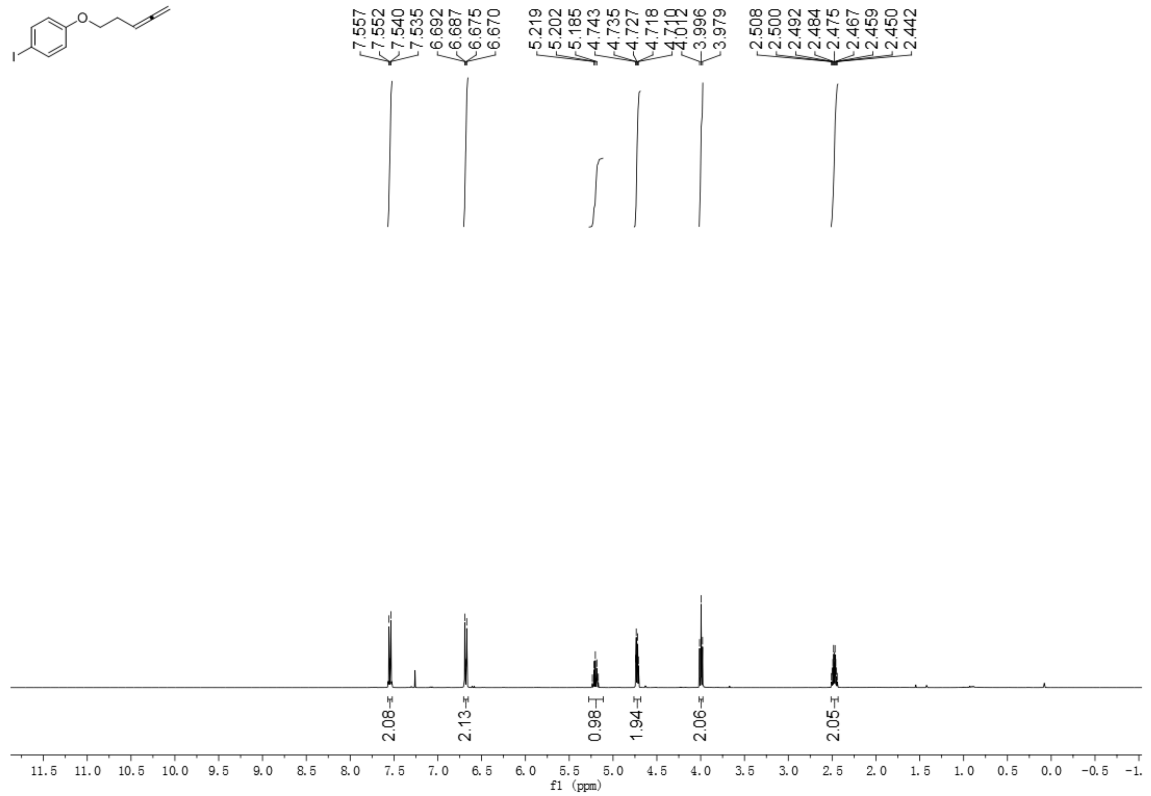

Supplementary Figure 5. ^{1}H NMR spectra for 1-iodo-4-(penta-3,4-dien-1-yloxy)benzene

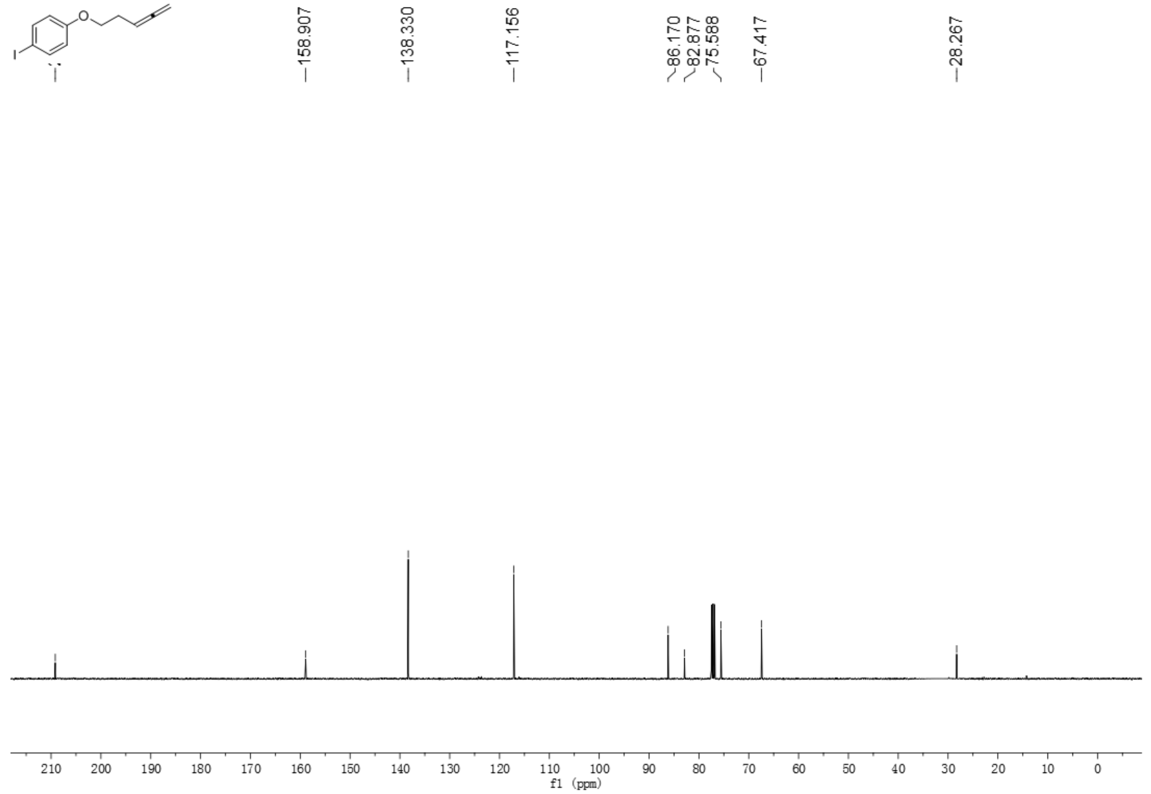

Supplementary Figure 6. ^{13}C NMR spectra for 1-iodo-4-(penta-3,4-dien-1-yloxy)benzene

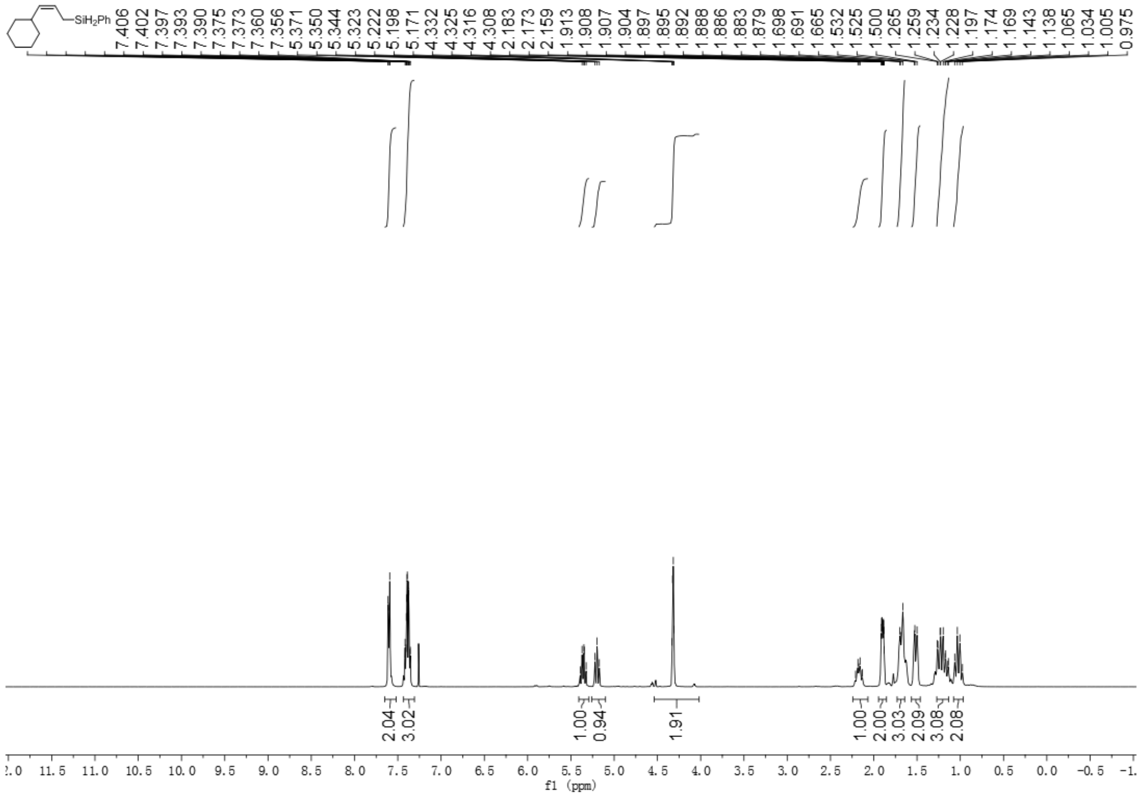

Supplementary Figure 7. ^{1}H NMR spectra for compound 1a

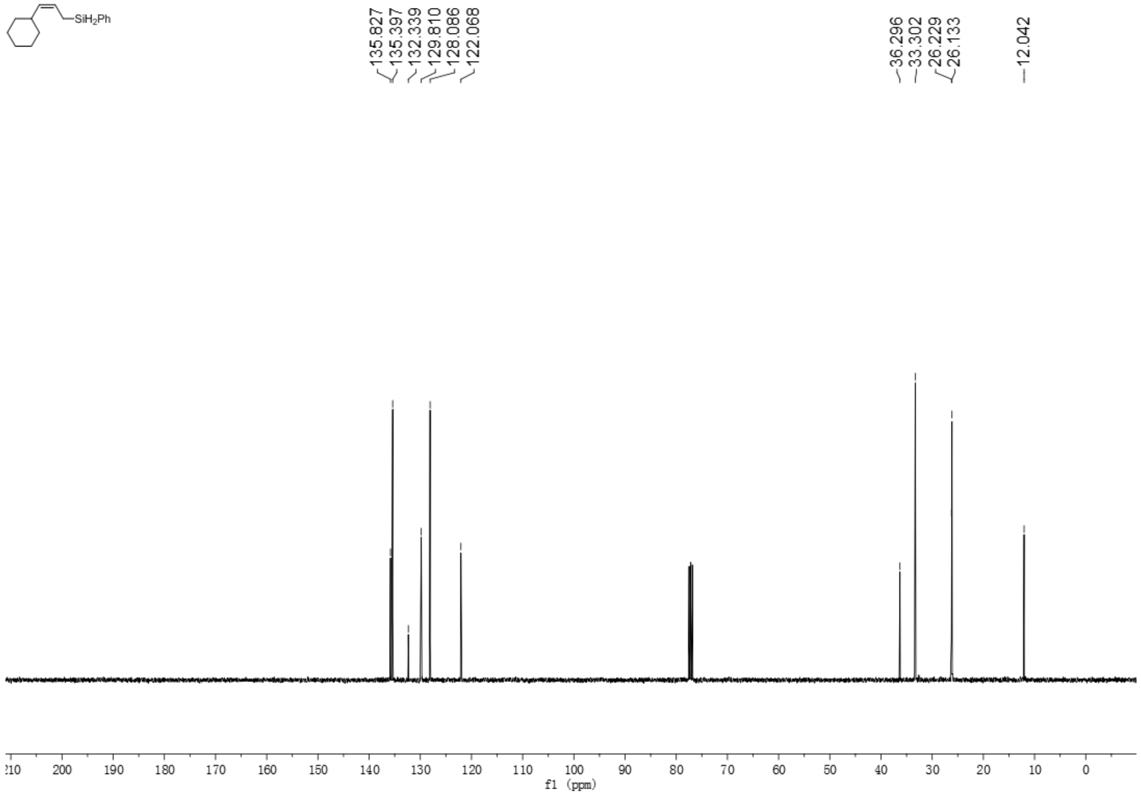

Supplementary Figure 8. ^{13}C NMR spectra for compound 1a

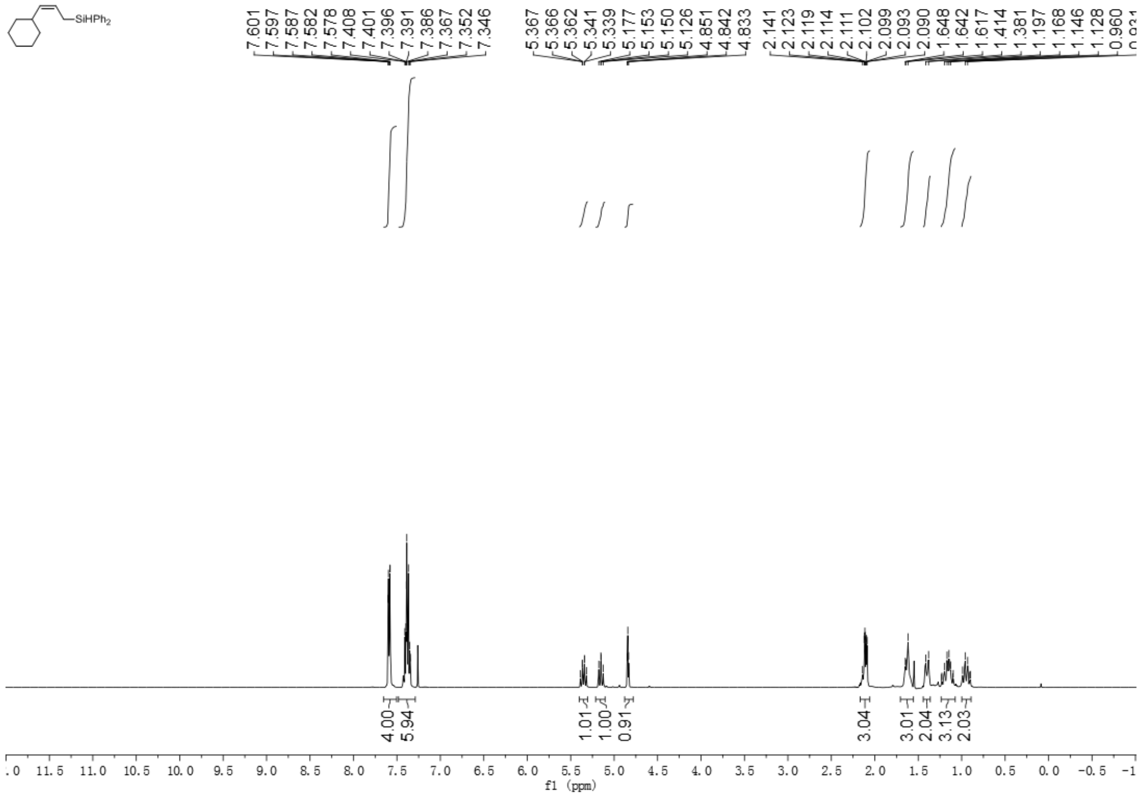

Supplementary Figure 9. ^{1}H NMR spectra for compound 1b

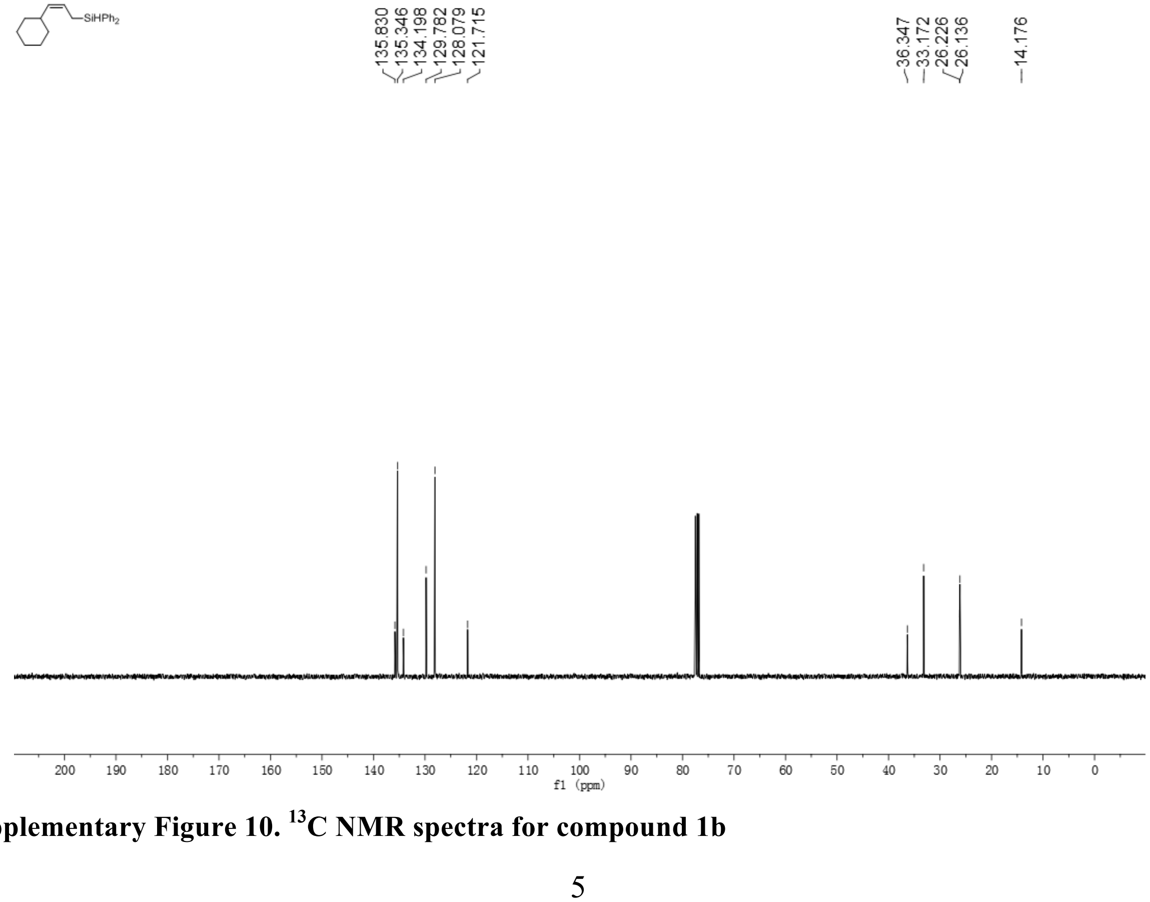

Supplementary Figure 10. ^{13}C NMR spectra for compound 1b

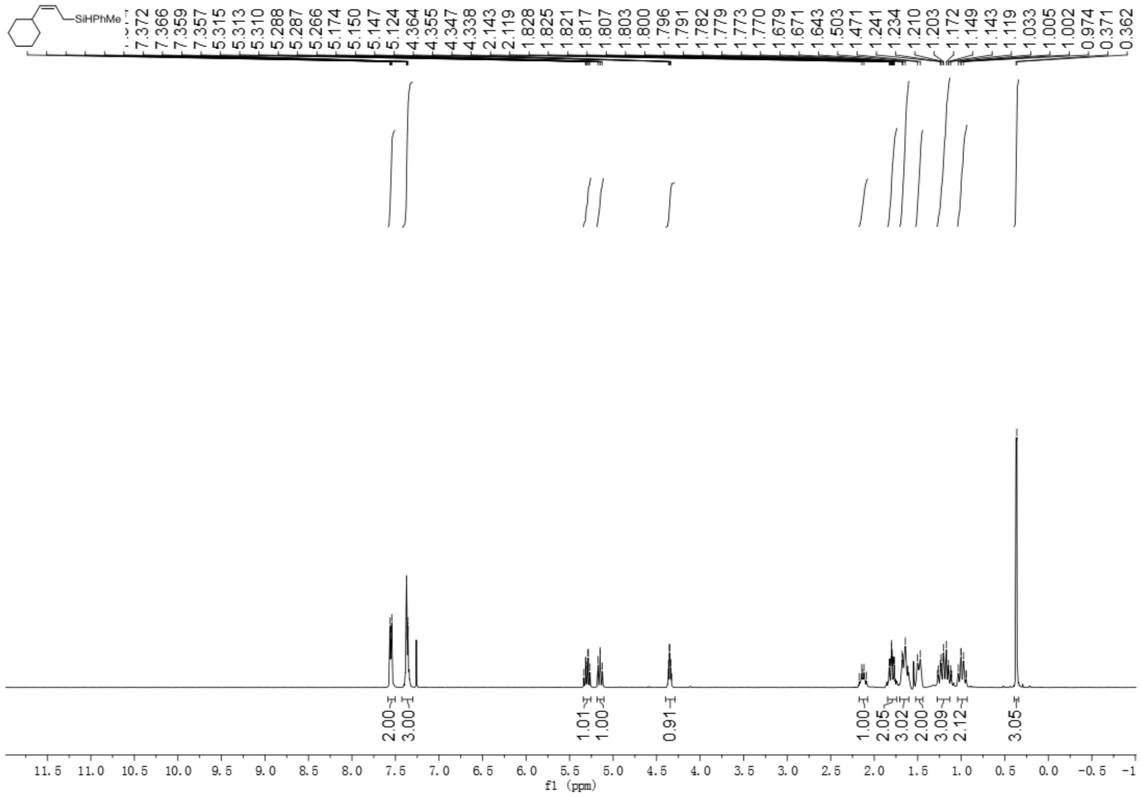

Supplementary Figure 11. ^{1}H NMR spectra for compound 1c

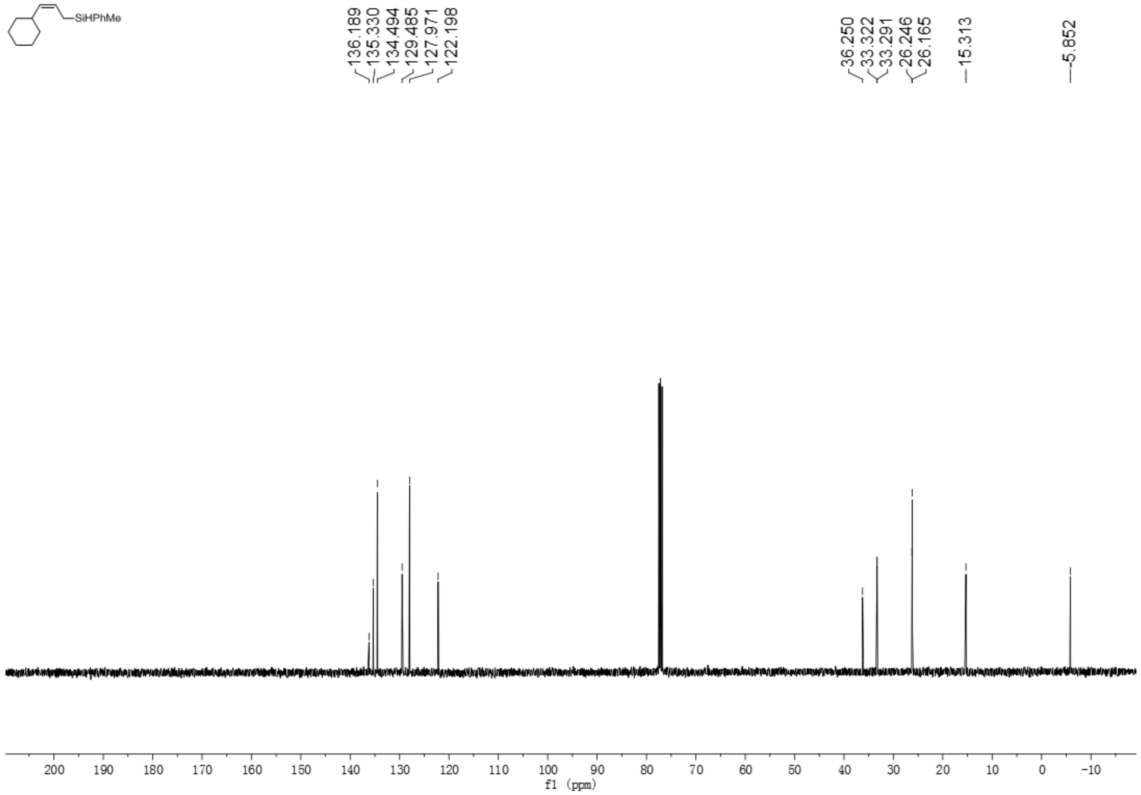

Supplementary Figure 12. ^{13}C NMR spectra for compound 1c

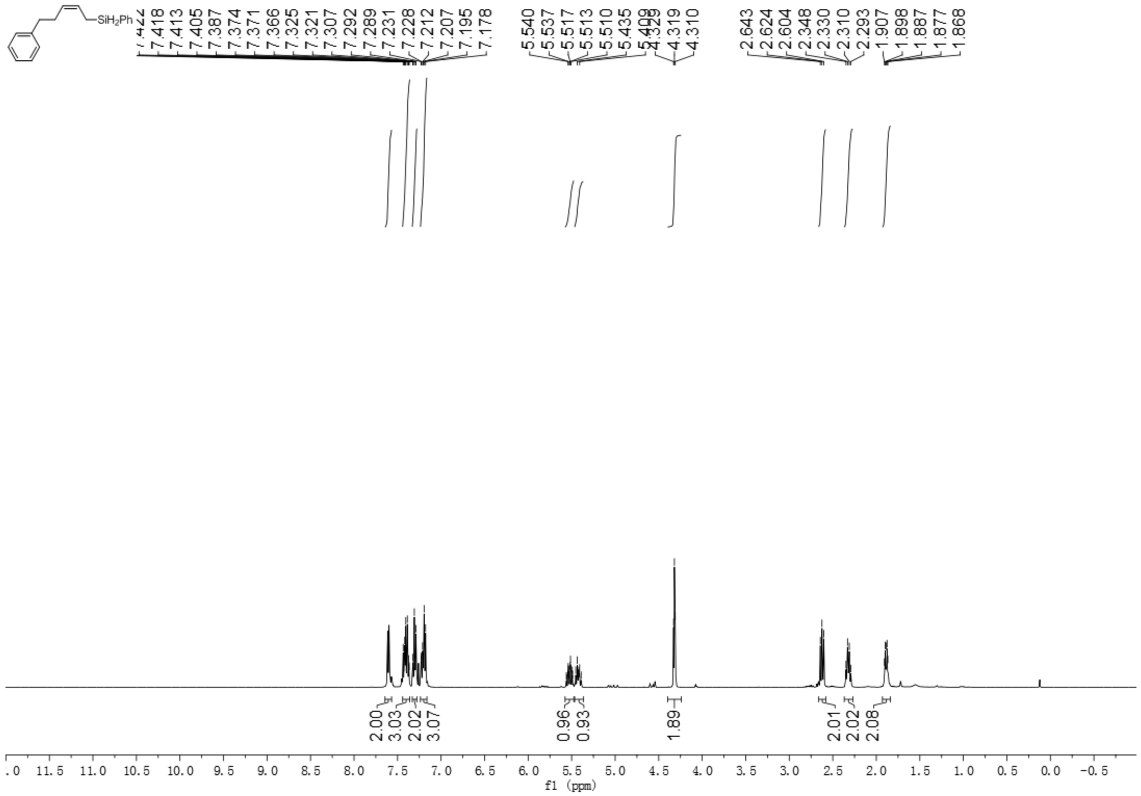

Supplementary Figure 13. ^{1}H NMR spectra for compound 1d

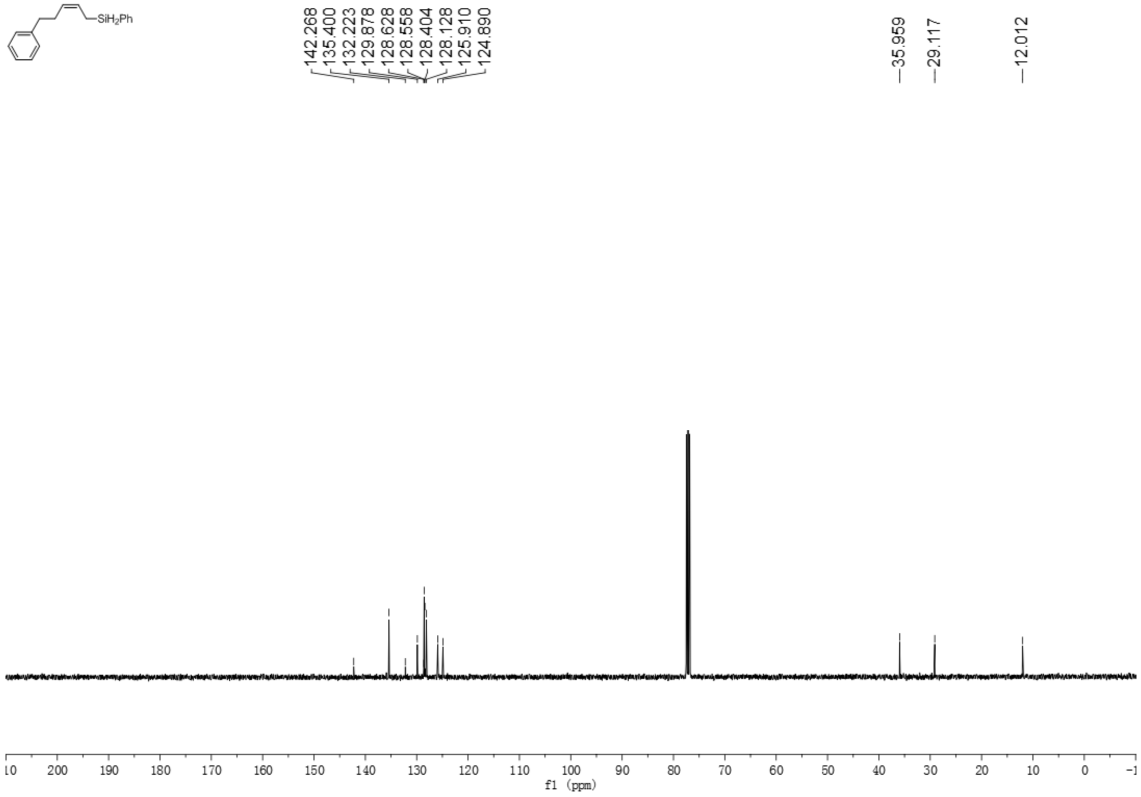

Supplementary Figure 14. ^{13}C NMR spectra for compound 1d

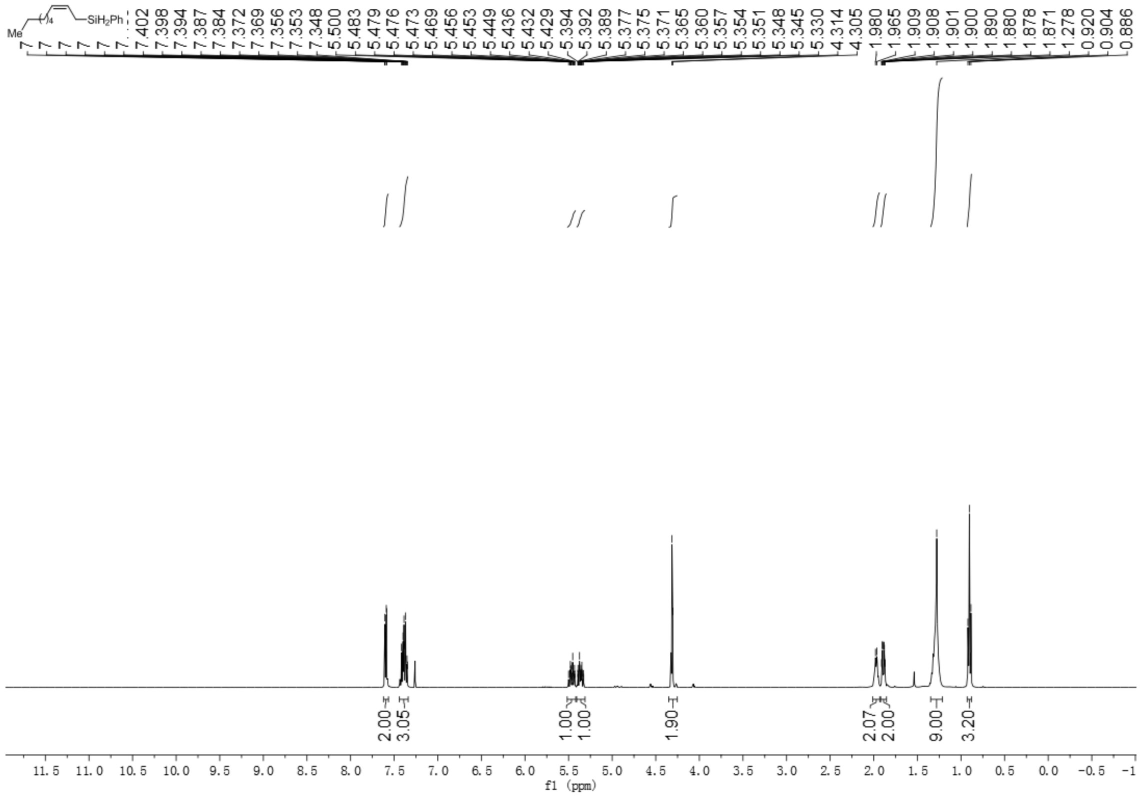

Supplementary Figure 15. ^{1}H NMR spectra for compound 1e

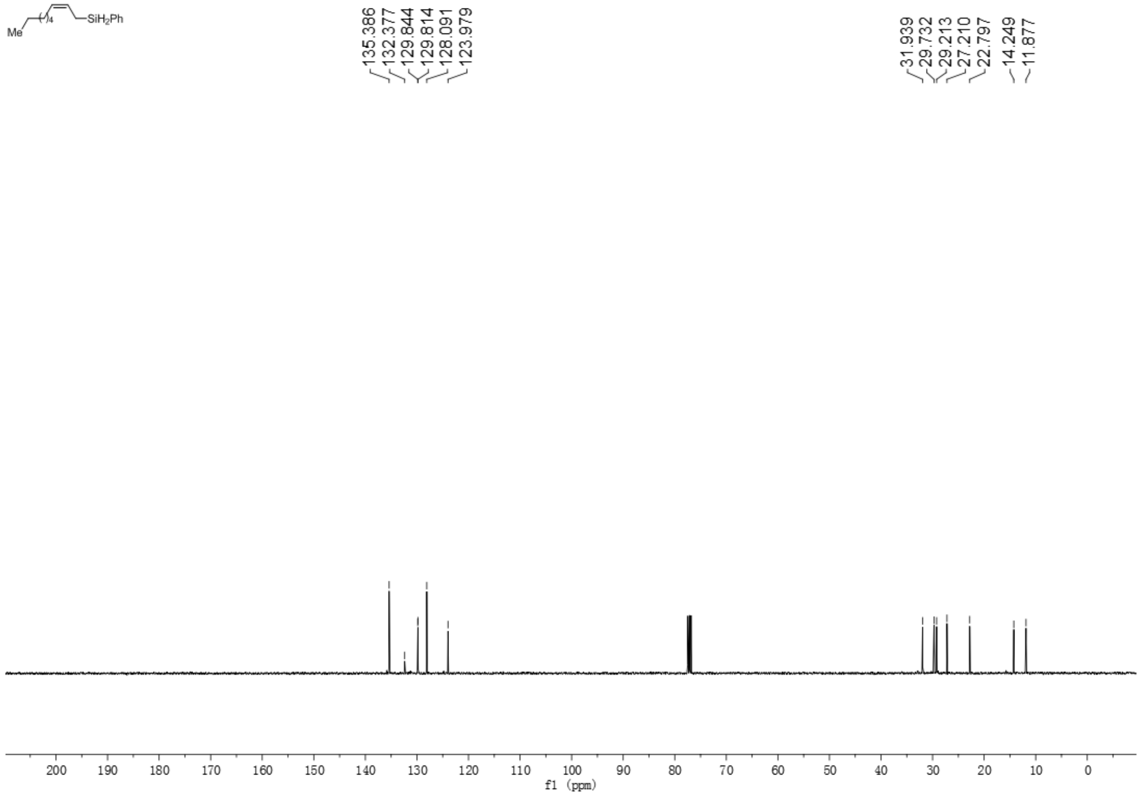

Supplementary Figure 16. ^{13}C NMR spectra for compound 1e

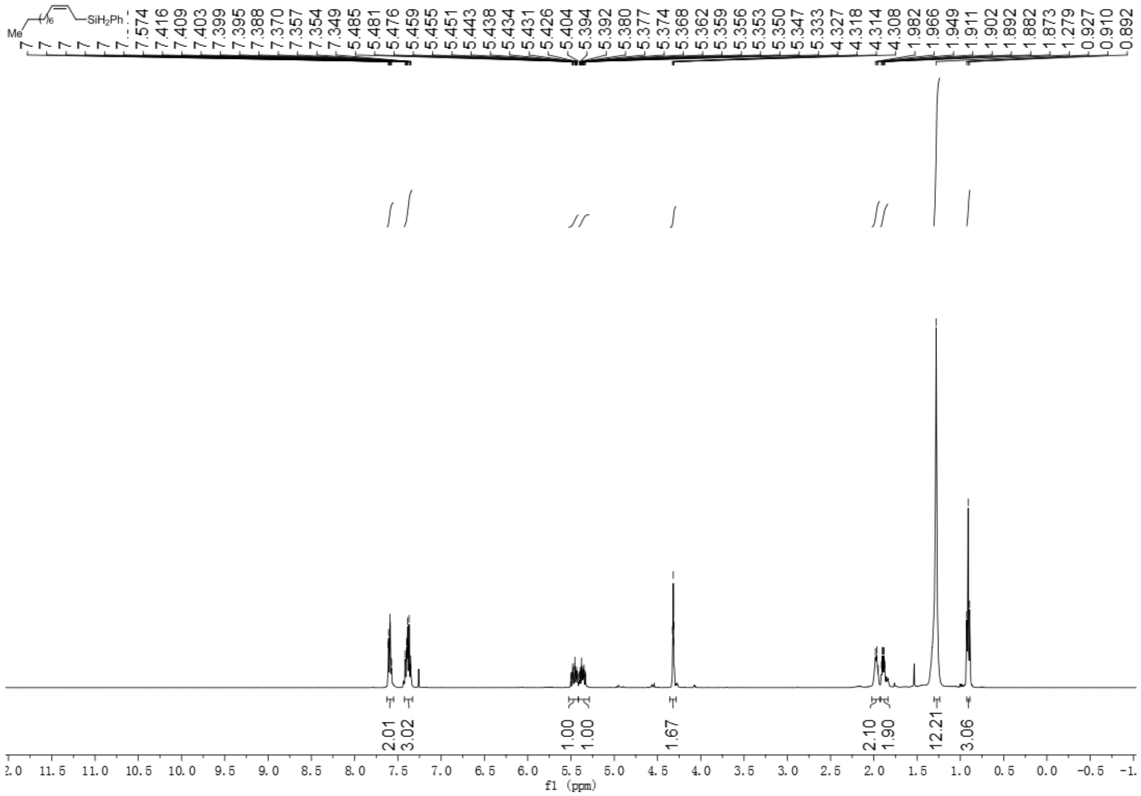

Supplementary Figure 17. ¹H NMR spectra for compound 1f

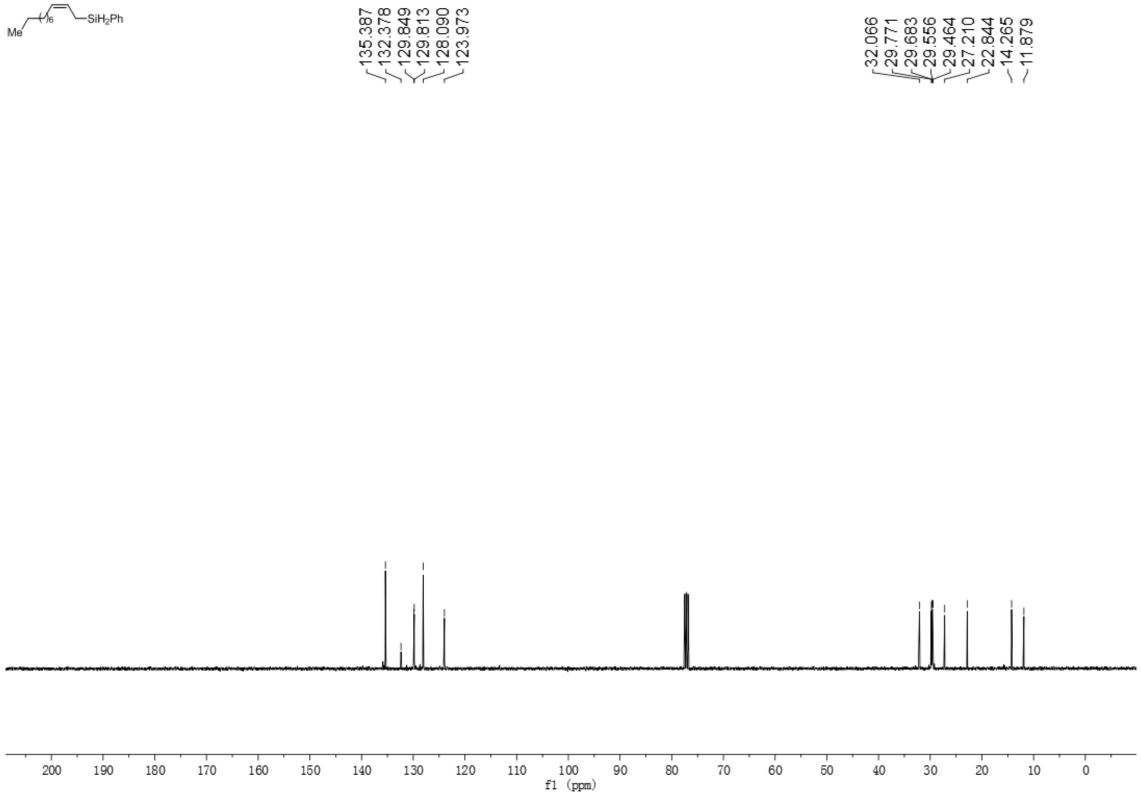

Supplementary Figure 18. ¹³C NMR spectra for compound 1f

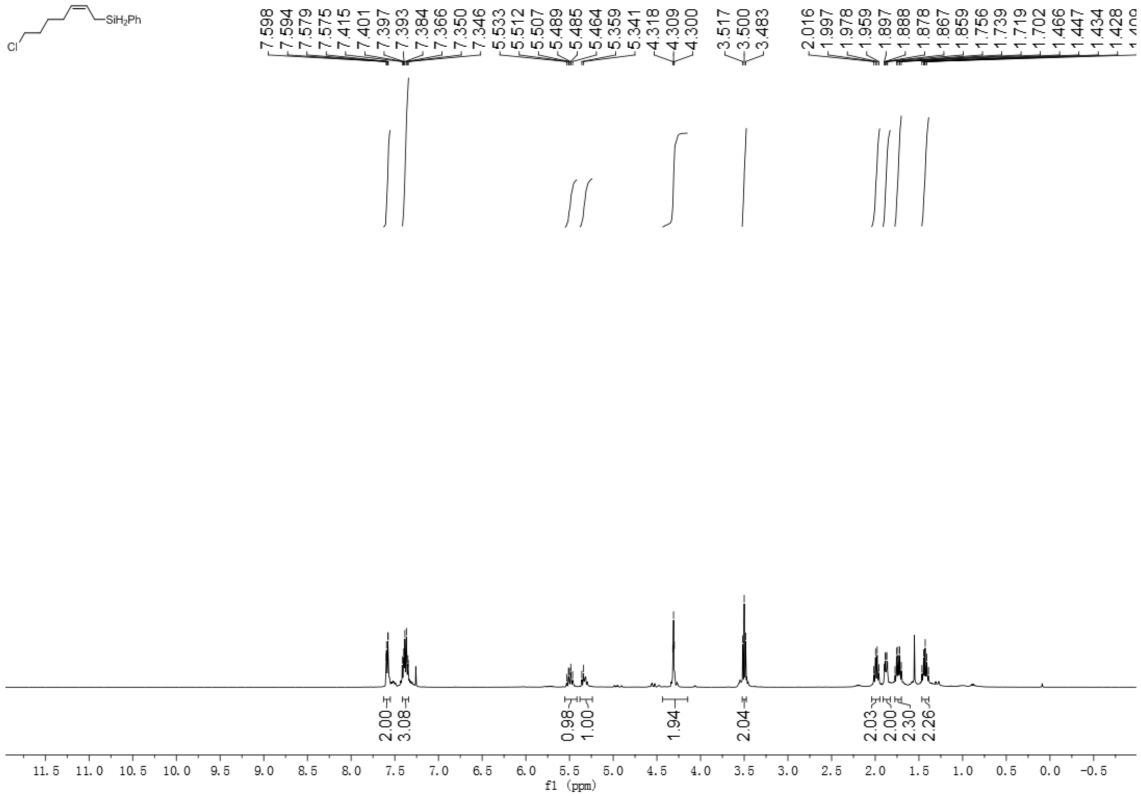

Supplementary Figure 19. ^{1}H NMR spectra for compound 1g

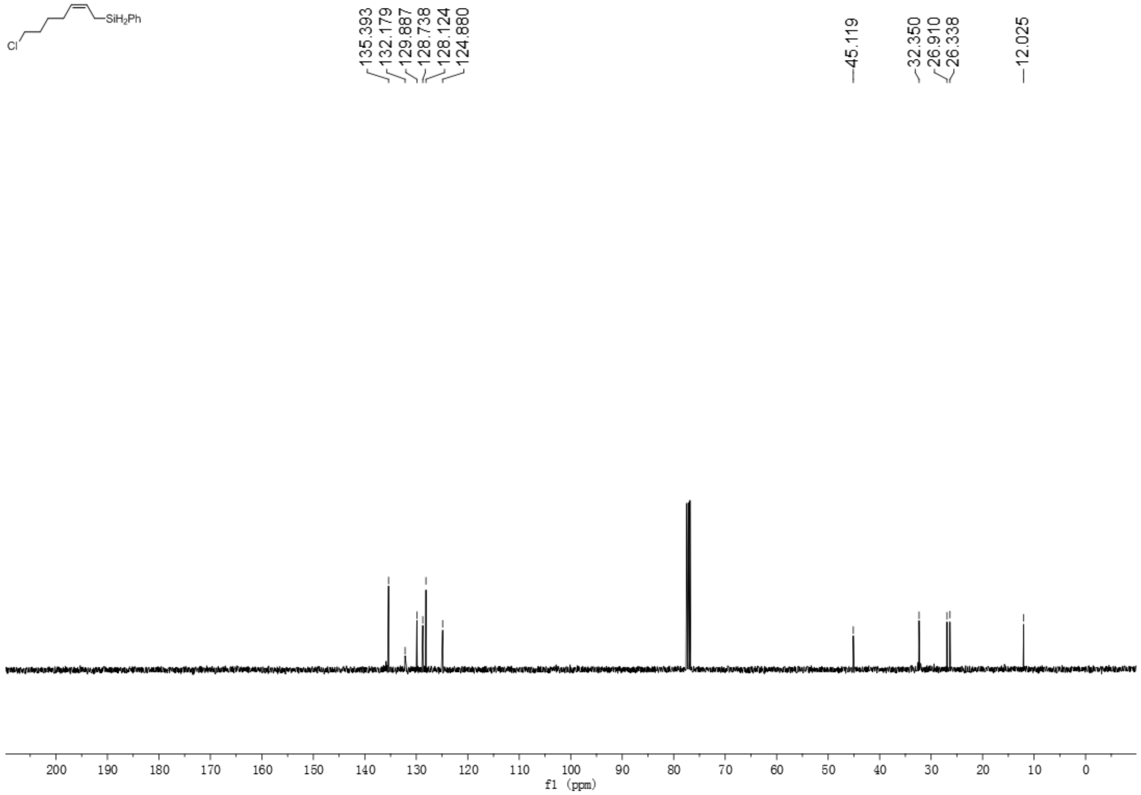

Supplementary Figure 20. ^{13}C NMR spectra for compound 1g

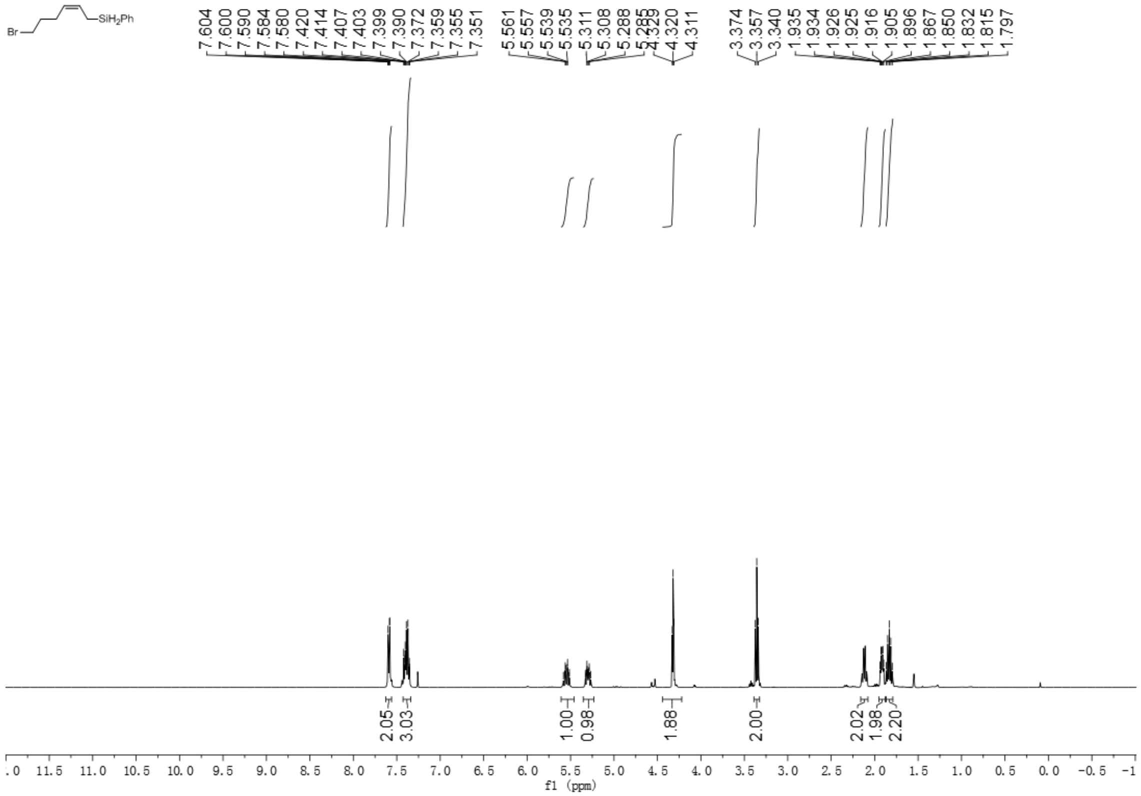

Supplementary Figure 21. ^{1}H NMR spectra for compound 1h

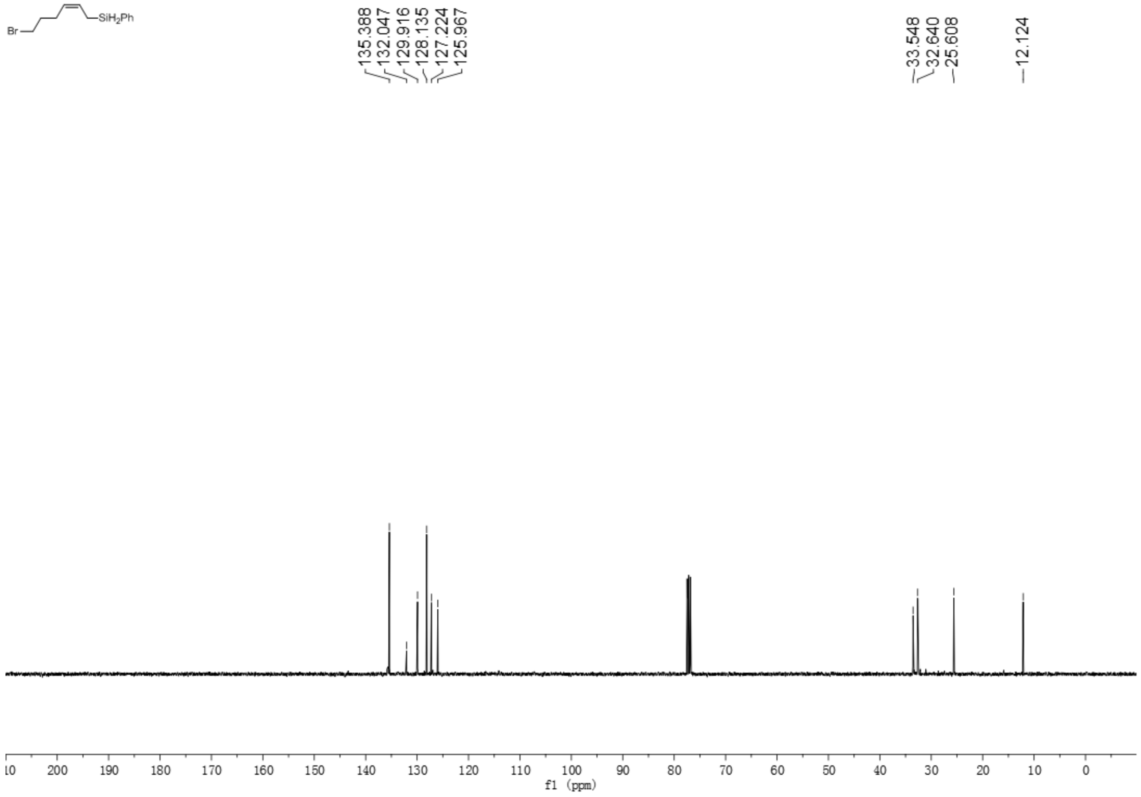

Supplementary Figure 22. ^{13}C NMR spectra for compound 1h

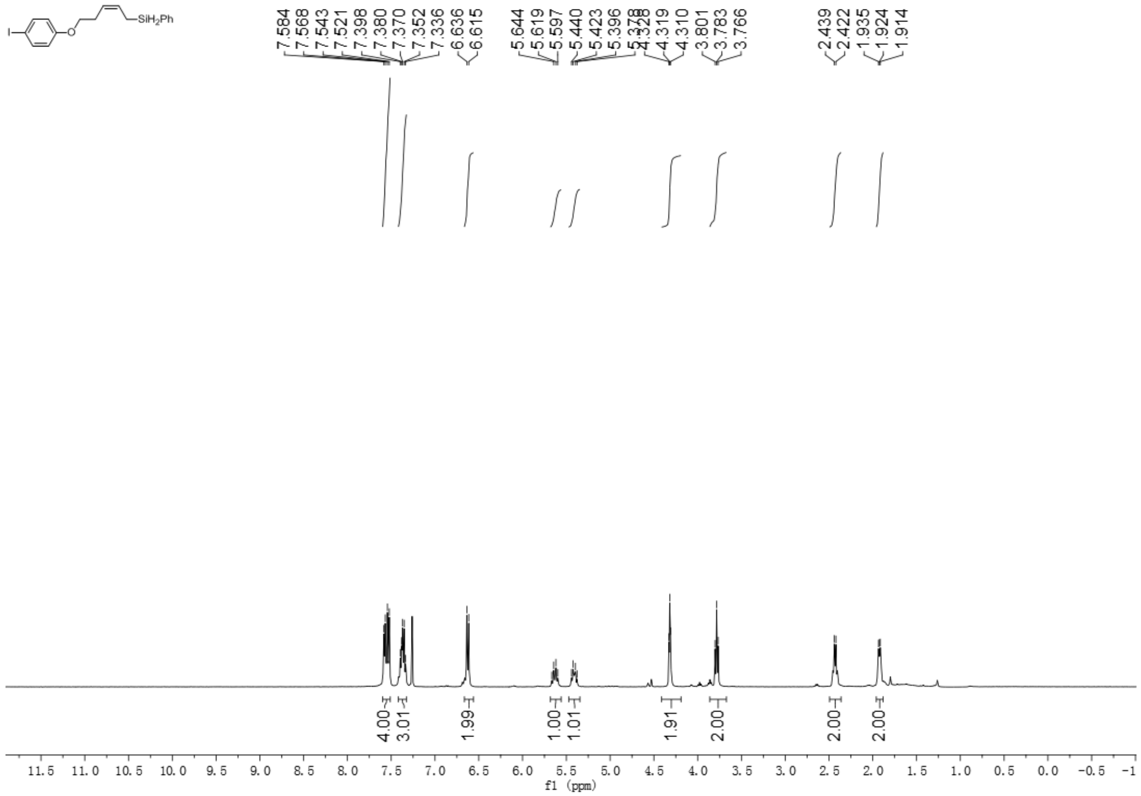

Supplementary Figure 23. ^{1}H NMR spectra for compound 1i

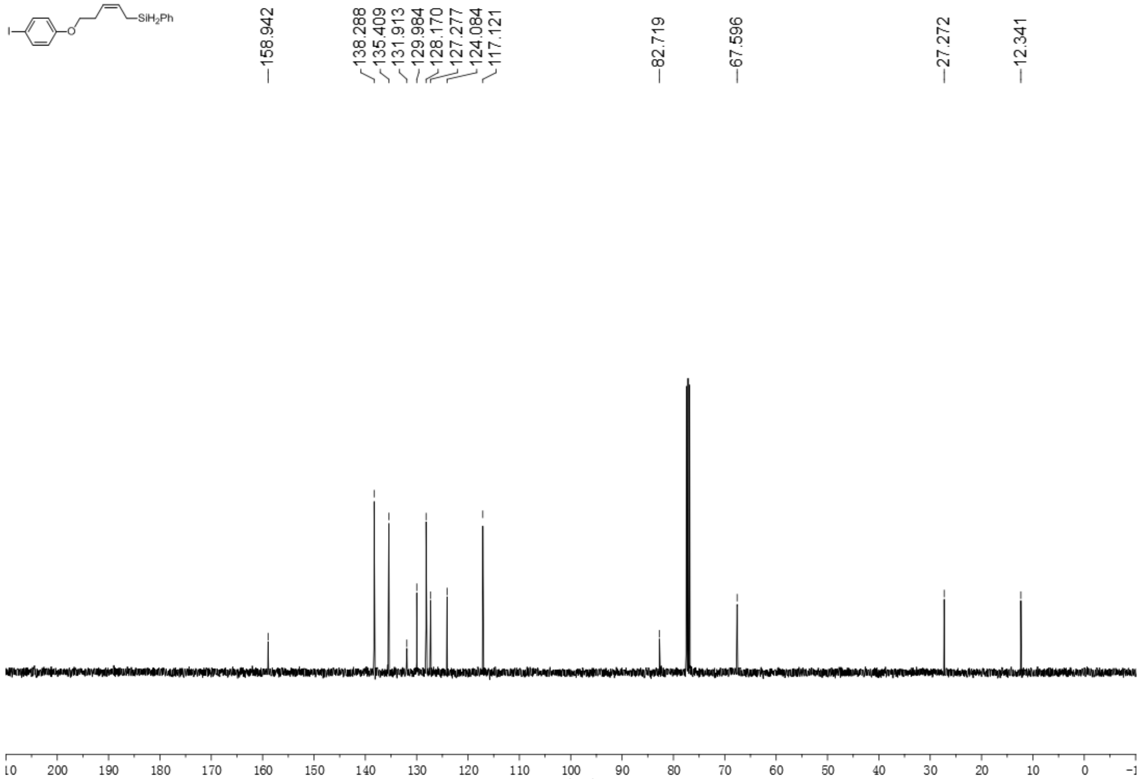

Supplementary Figure 24. ^{13}C NMR spectra for compound 1i

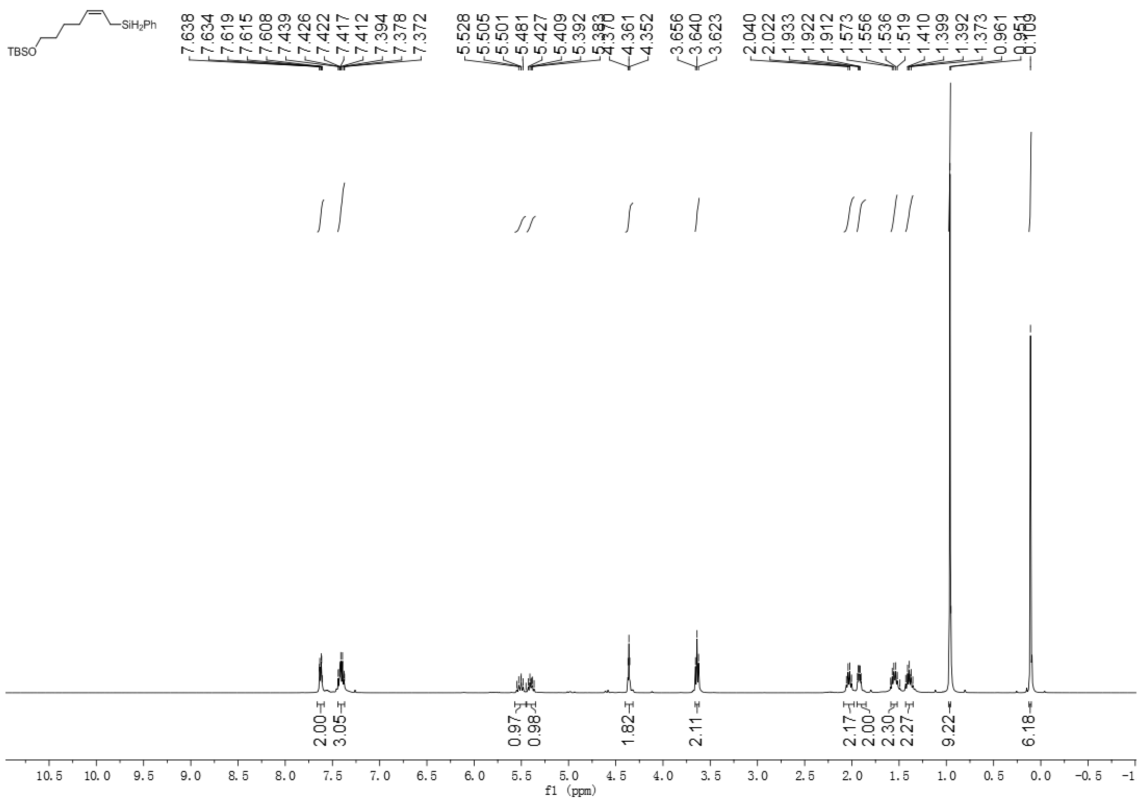

Supplementary Figure 25. ^{1}H NMR spectra for compound 1j

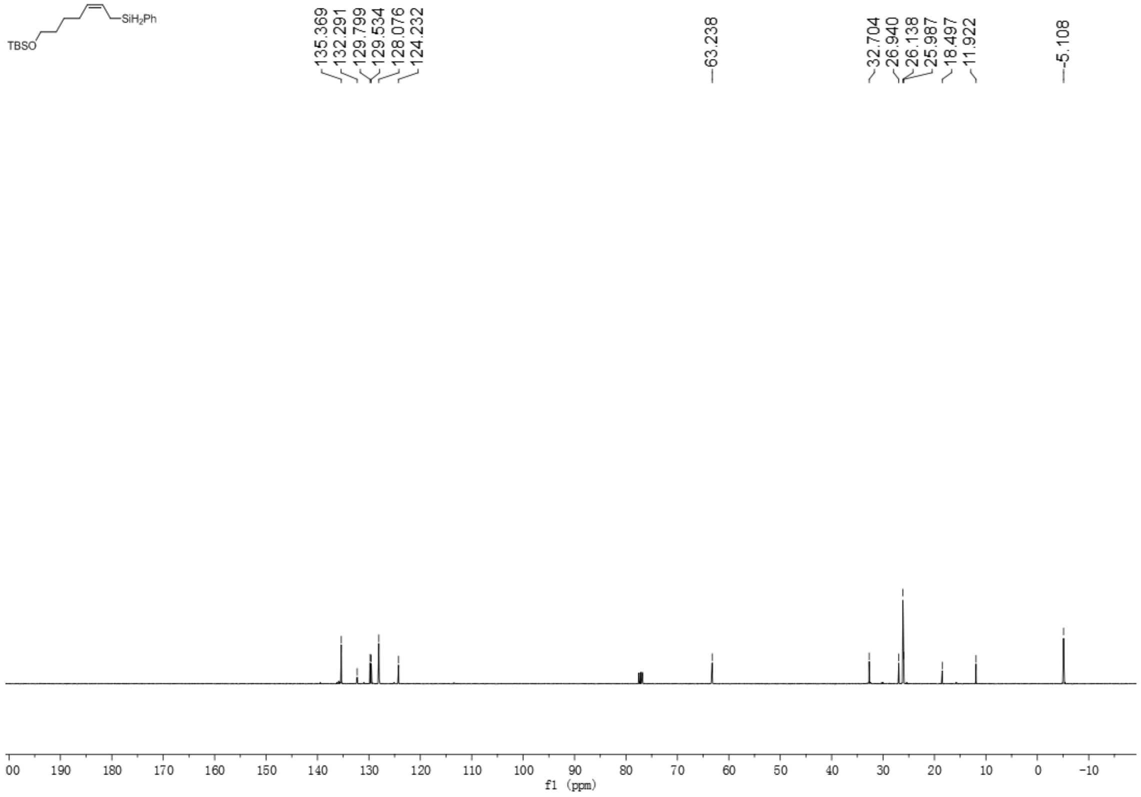

Supplementary Figure 26. ^{13}C NMR spectra for compound 1j

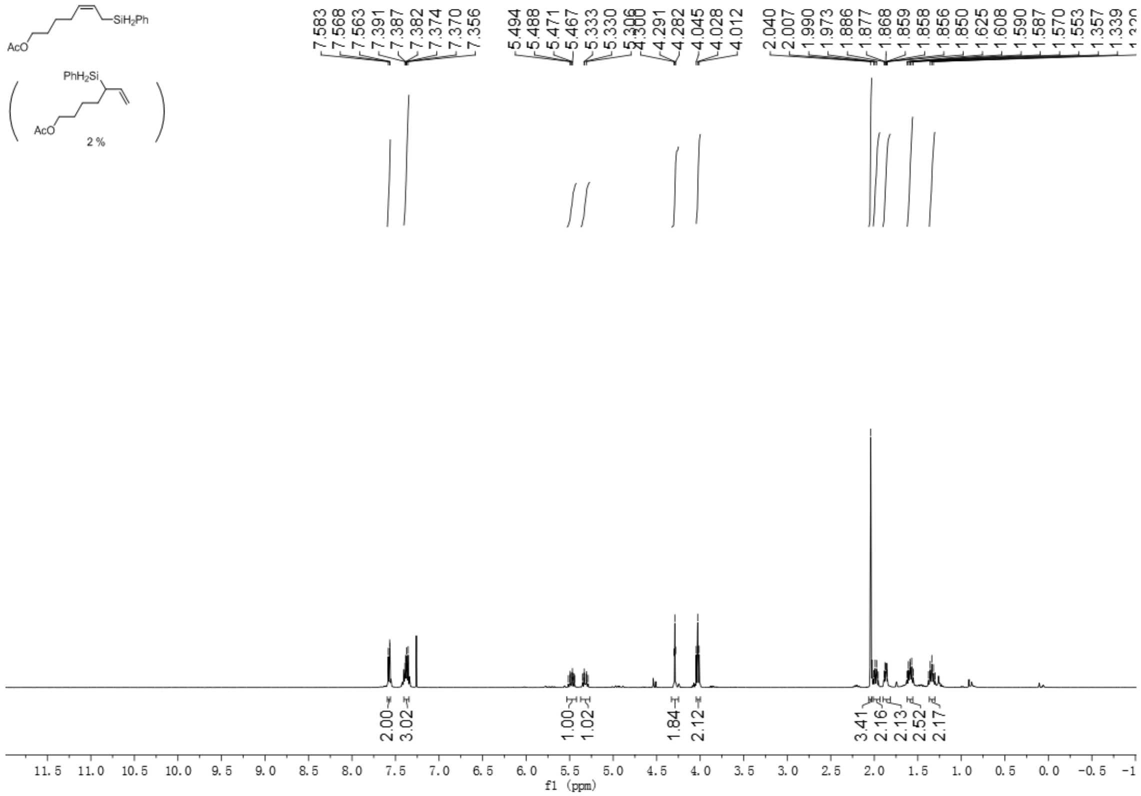

Supplementary Figure 27. ^{1}H NMR spectra for compound 1k

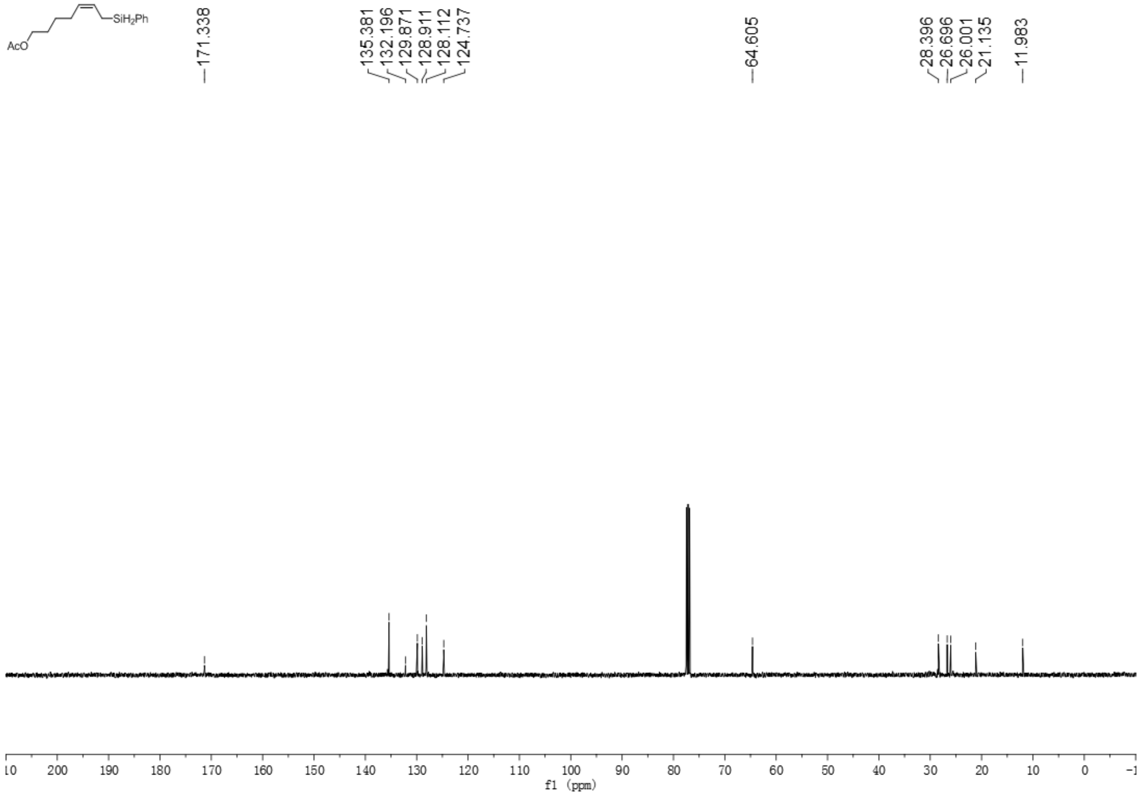

Supplementary Figure 28. ^{13}C NMR spectra for compound 1k

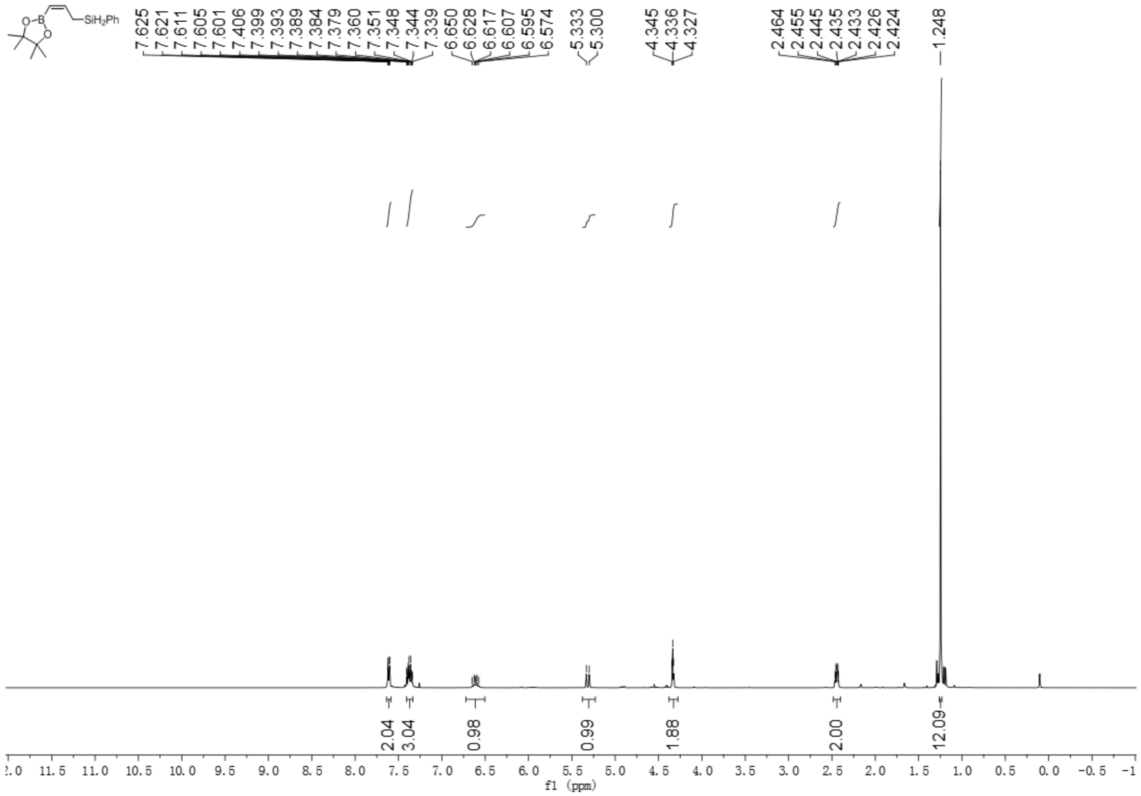

Supplementary Figure 29. ^{1}H NMR spectra for compound 1l

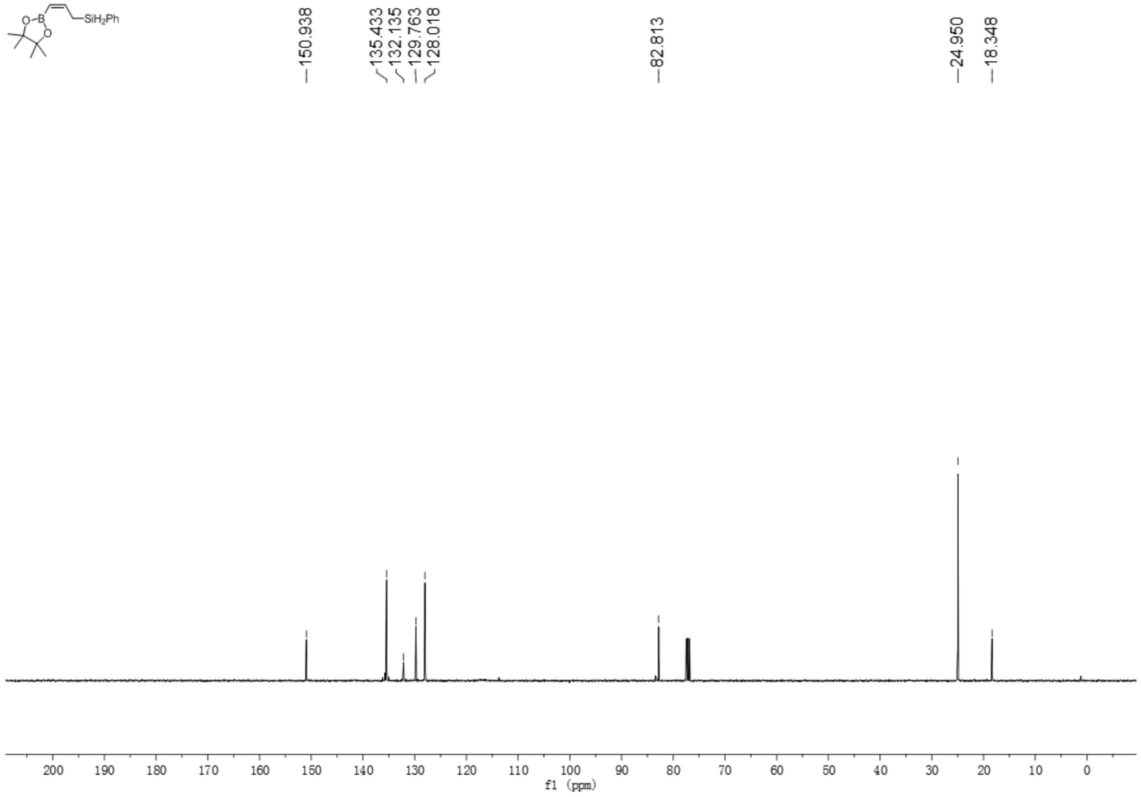

Supplementary Figure 30. ^{13}C NMR spectra for compound 1l

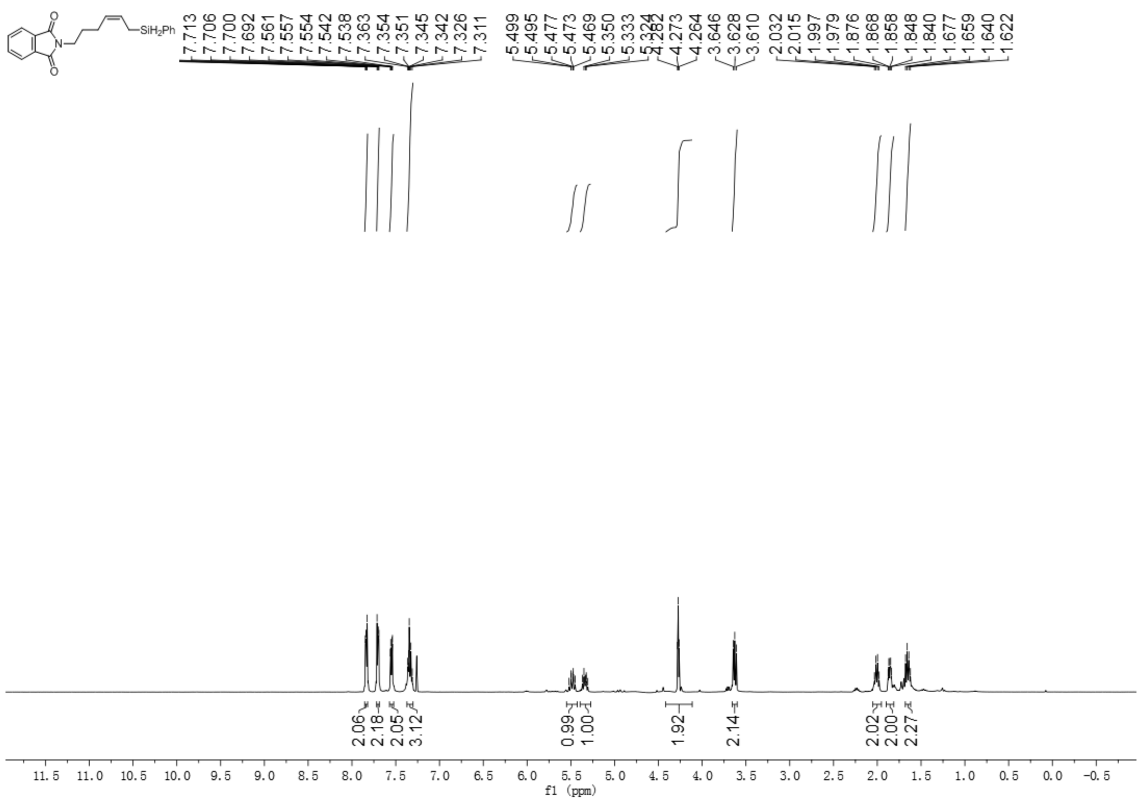

Supplementary Figure 31. ^{1}H NMR spectra for compound 1m

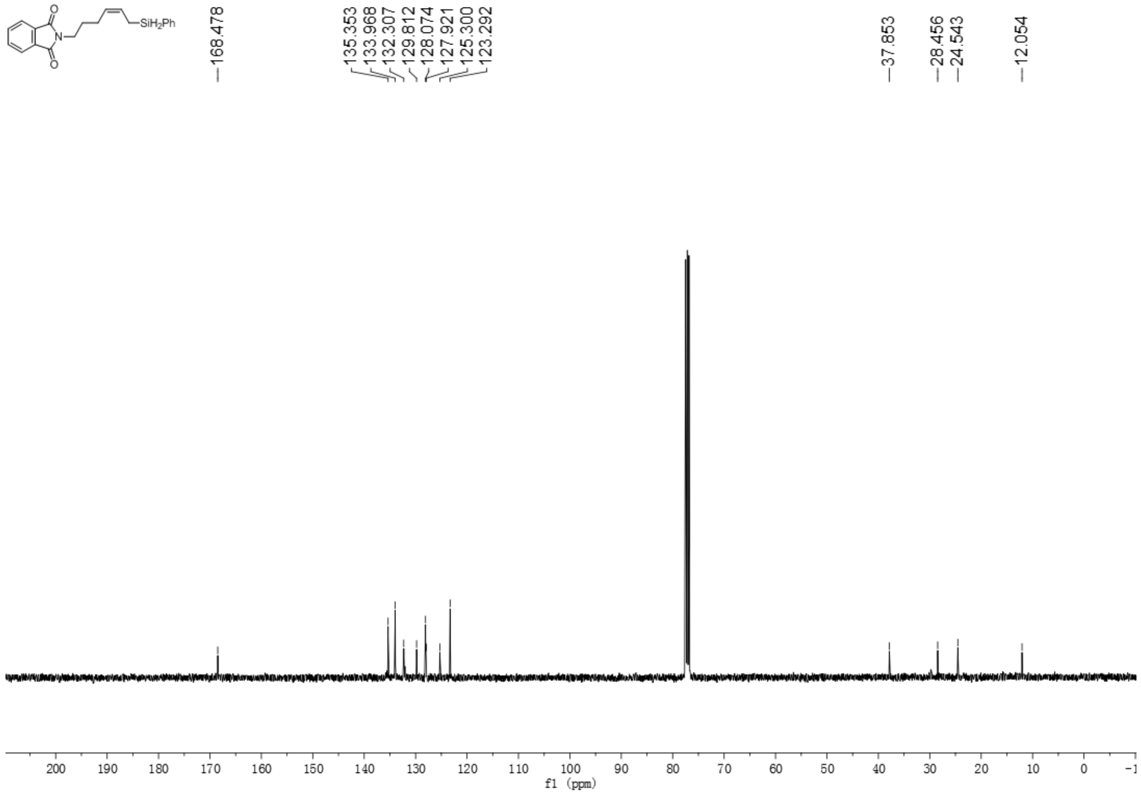

Supplementary Figure 32. ^{13}C NMR spectra for compound 1m

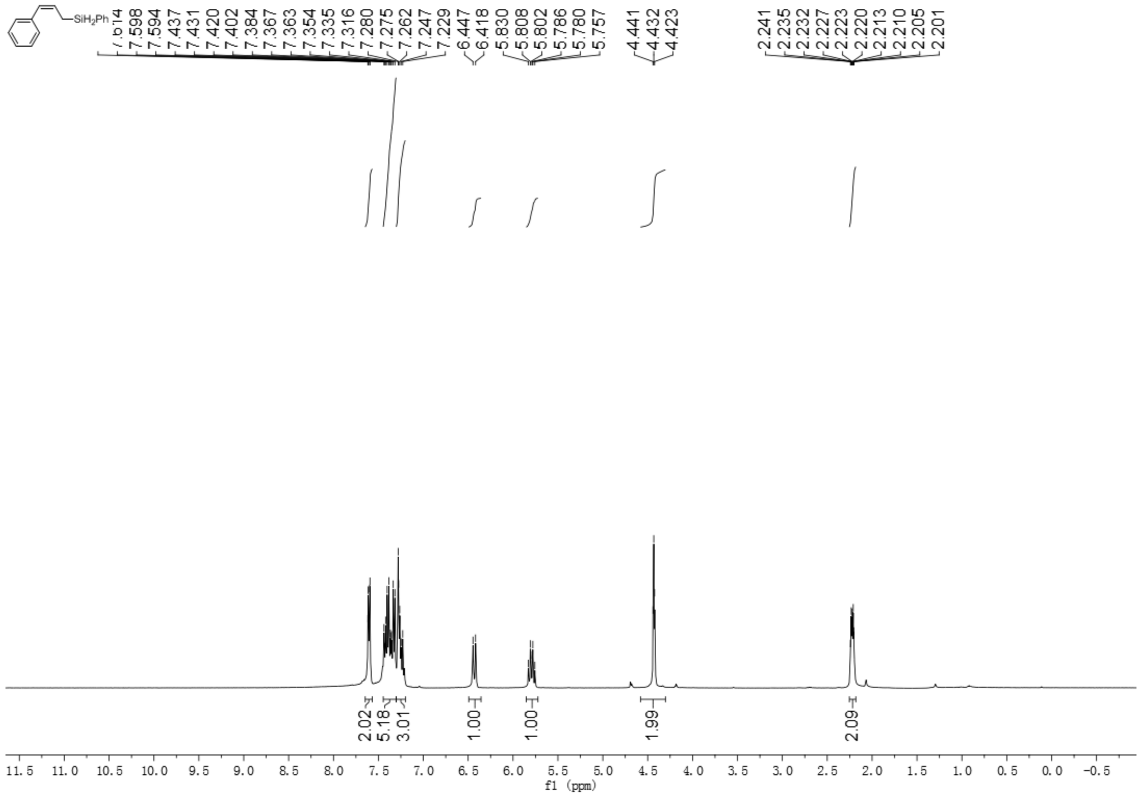

Supplementary Figure 33. ^{1}H NMR spectra for compound 1n

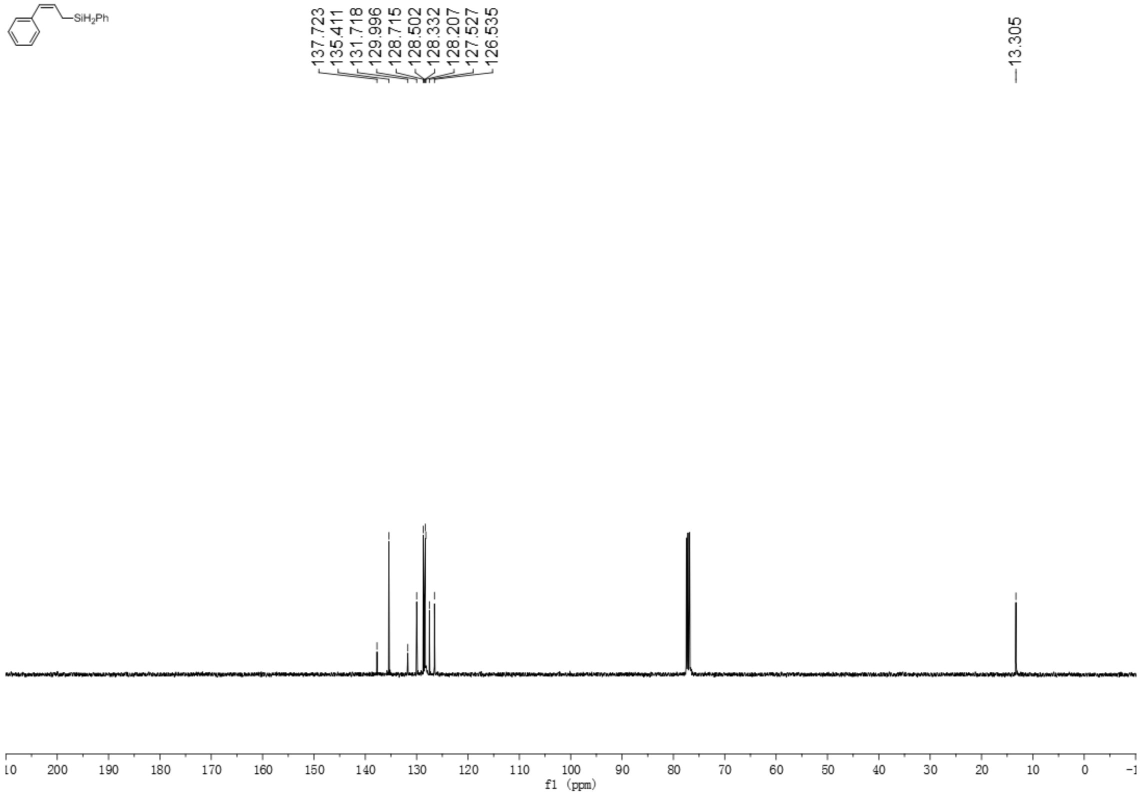

Supplementary Figure 34. ^{13}C NMR spectra for compound 1n

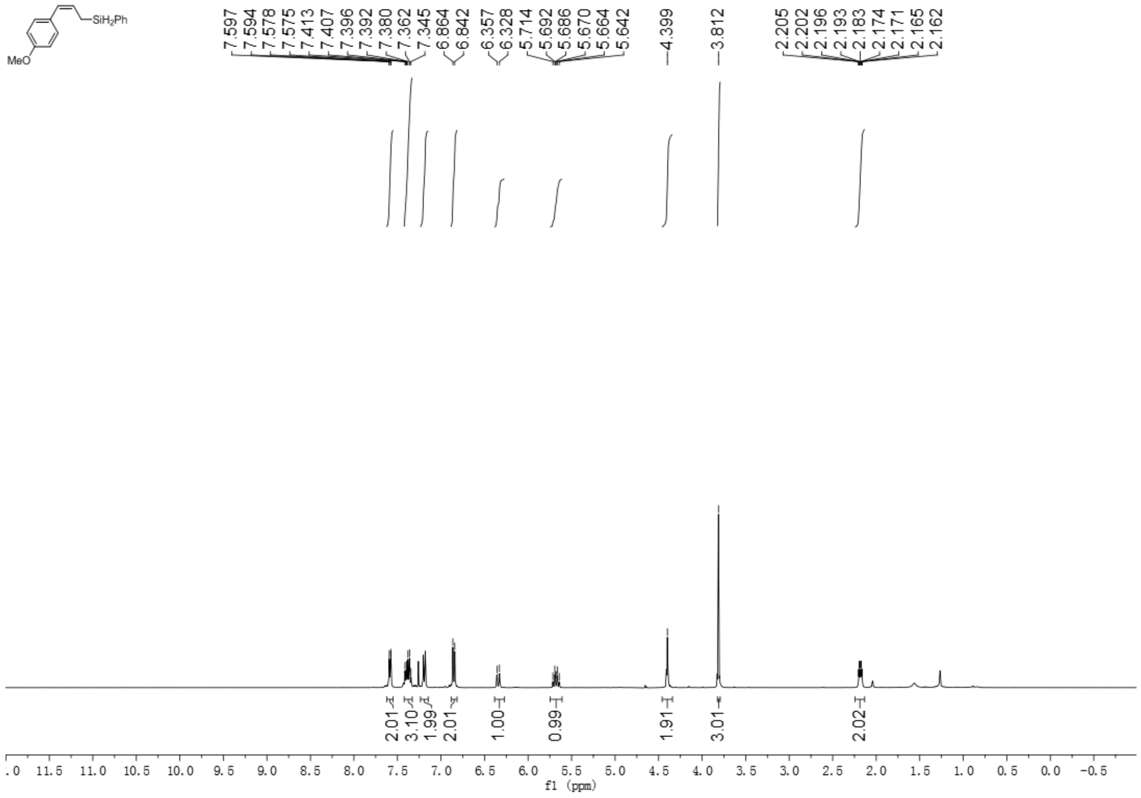

Supplementary Figure 35. ^{1}H NMR spectra for compound 1o

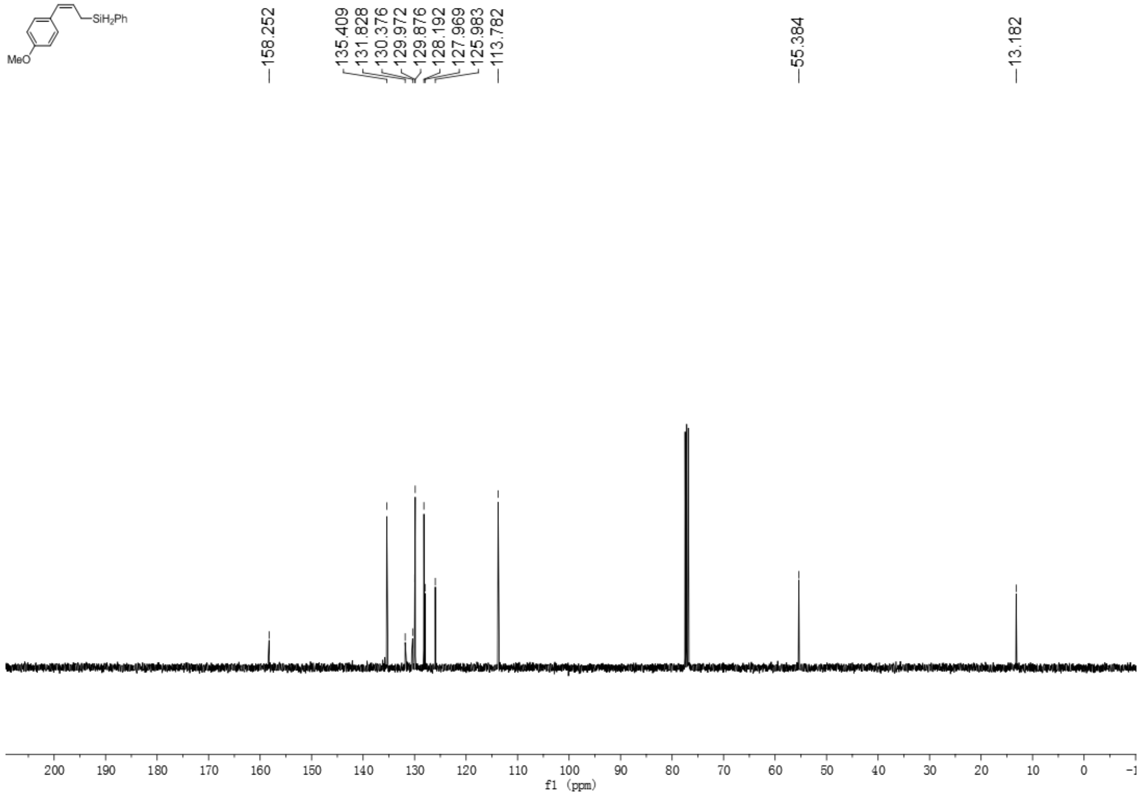

Supplementary Figure 36. ^{13}C NMR spectra for compound 1o

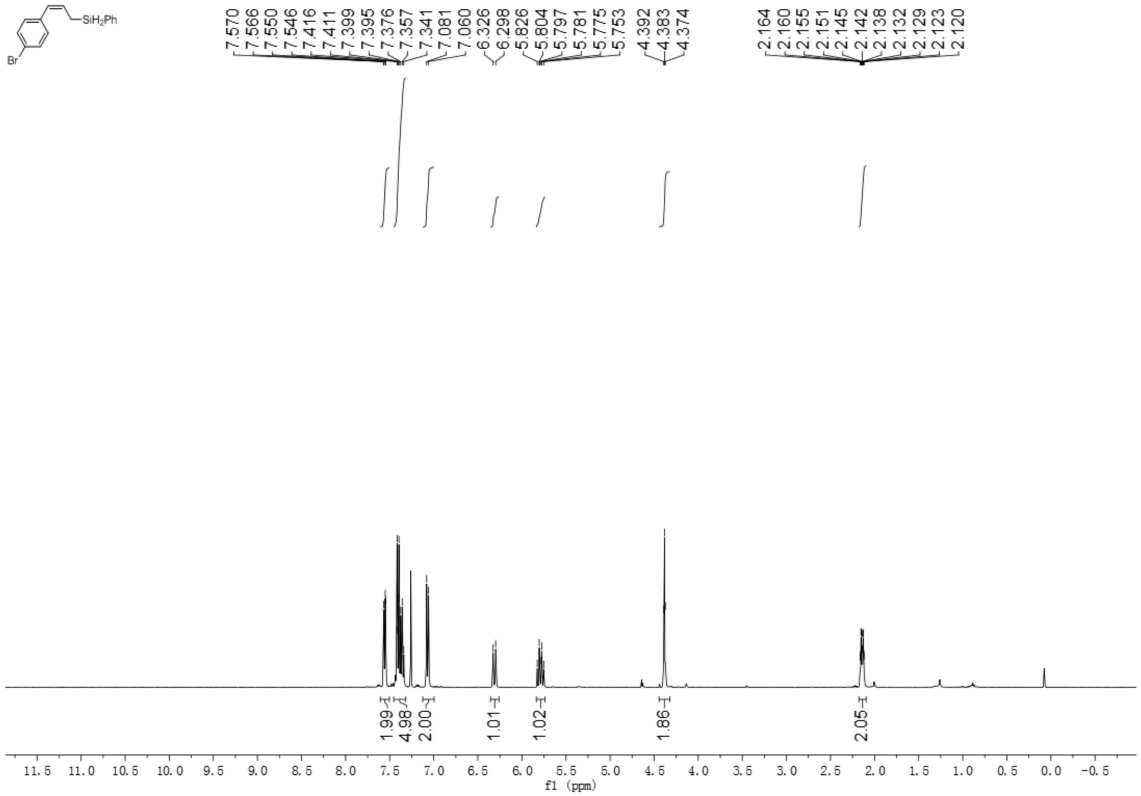

Supplementary Figure 37. ^{1}H NMR spectra for compound 1p

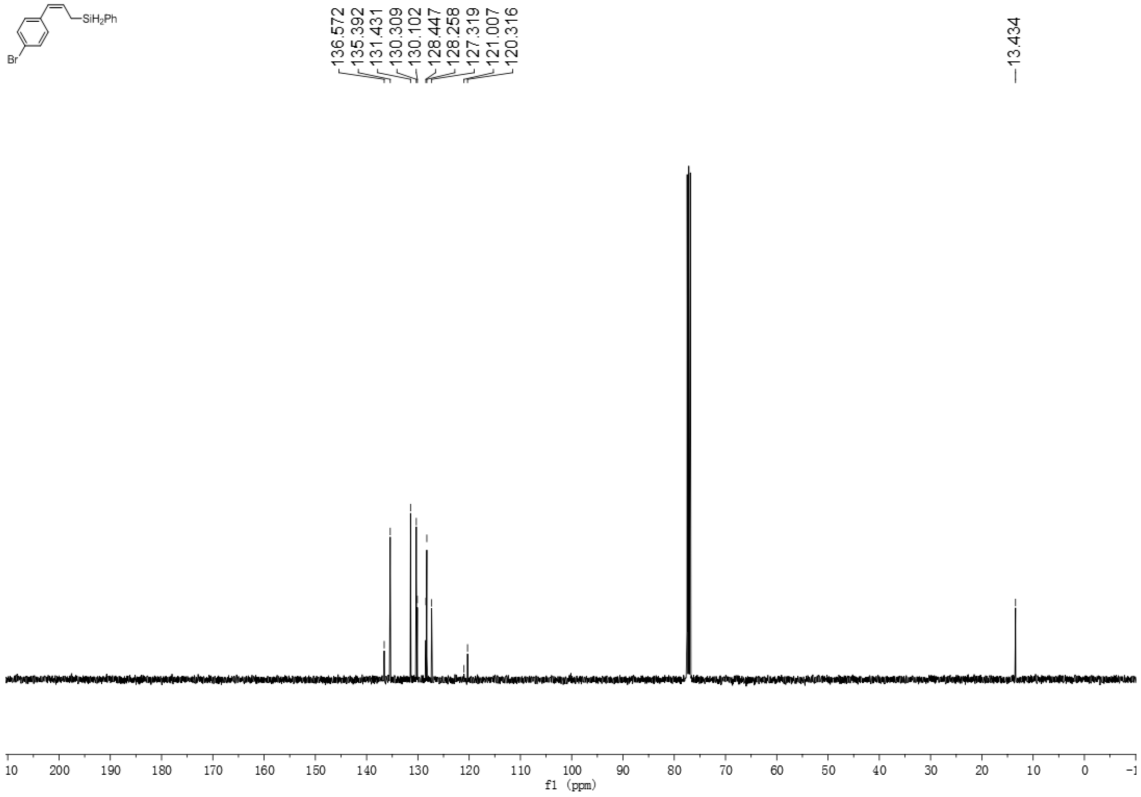

Supplementary Figure 38. ^{13}C NMR spectra for compound 1p

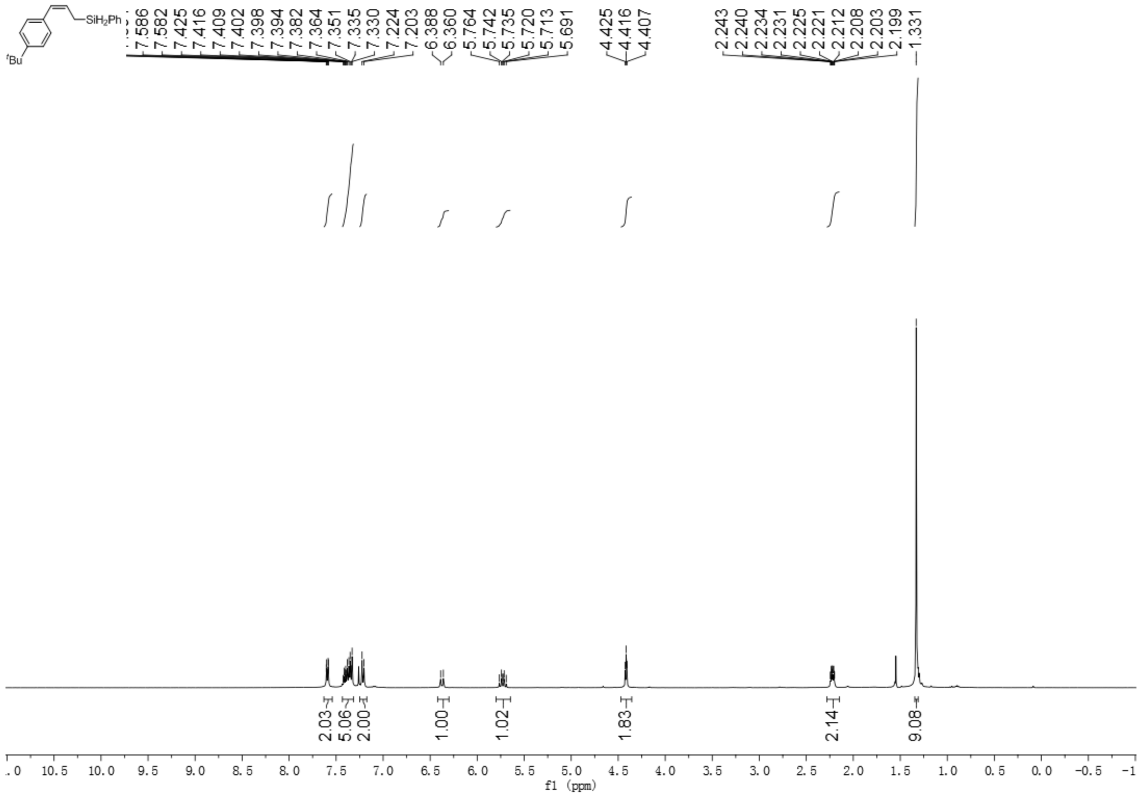

Supplementary Figure 39. ^{1}H NMR spectra for compound 1q

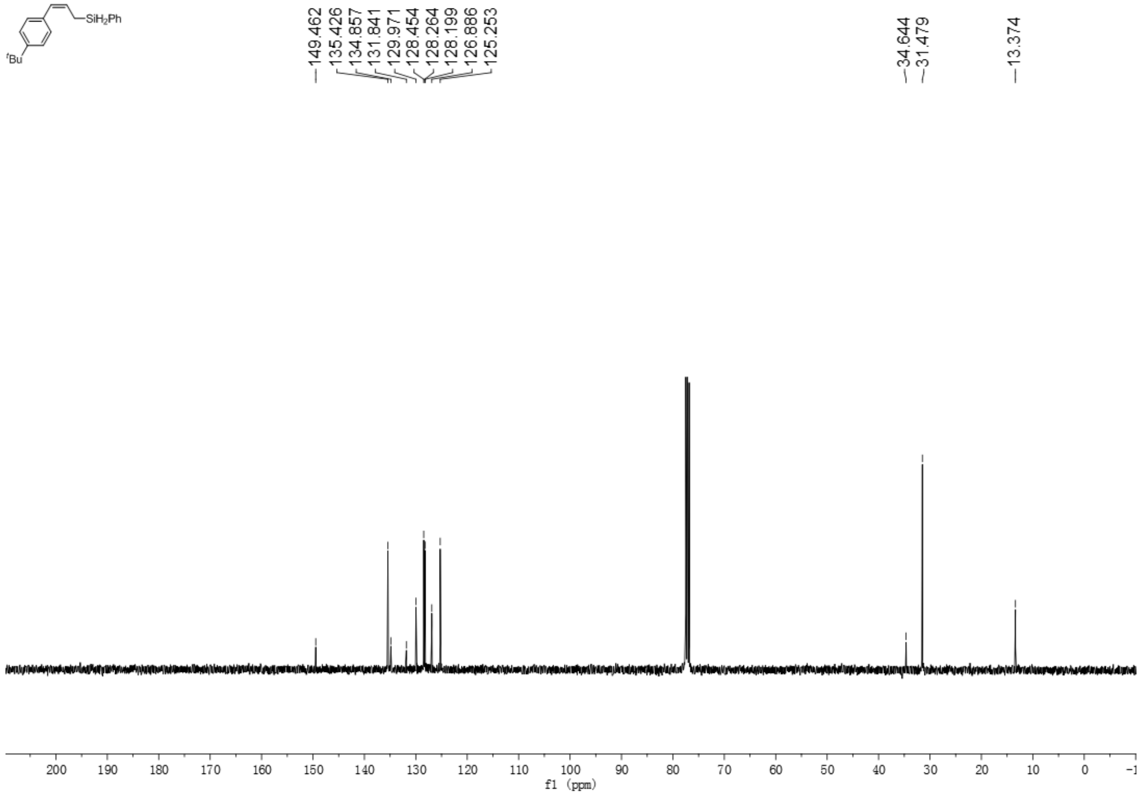

Supplementary Figure 40. ^{13}C NMR spectra for compound 1q

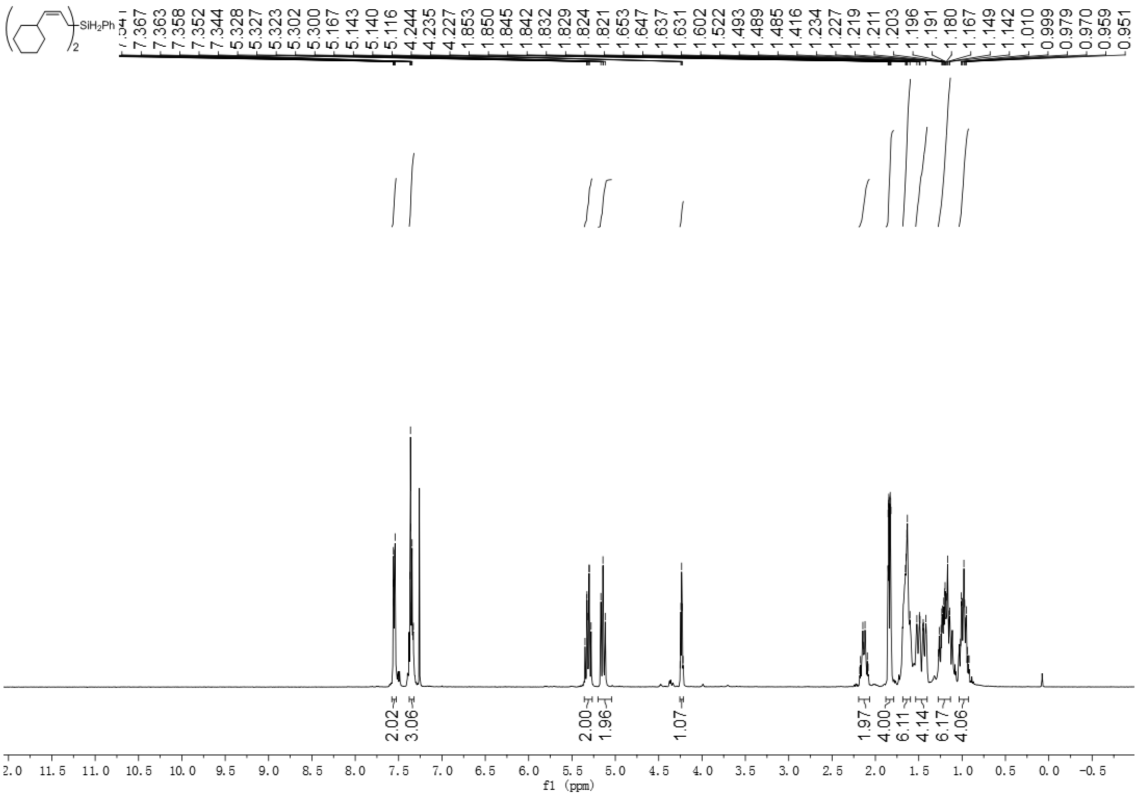

Supplementary Figure 41. ^{1}H NMR spectra for compound 2

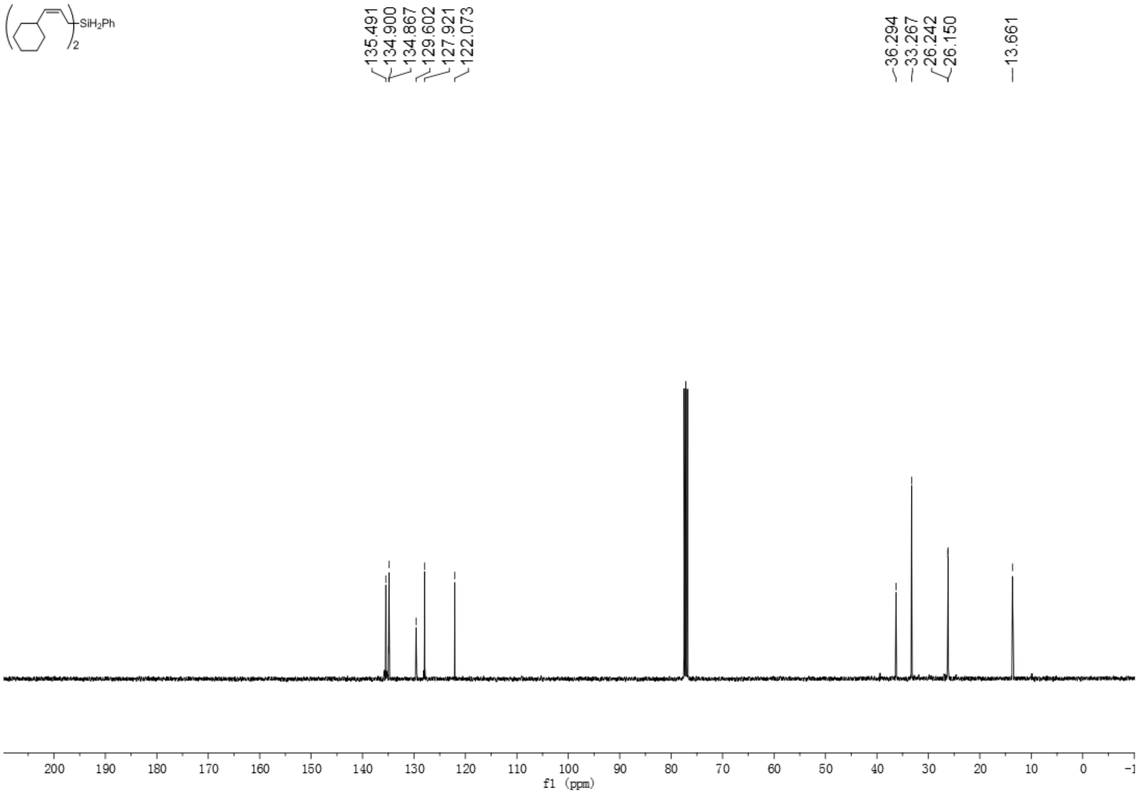

Supplementary Figure 42. ^{13}C NMR spectra for compound 2

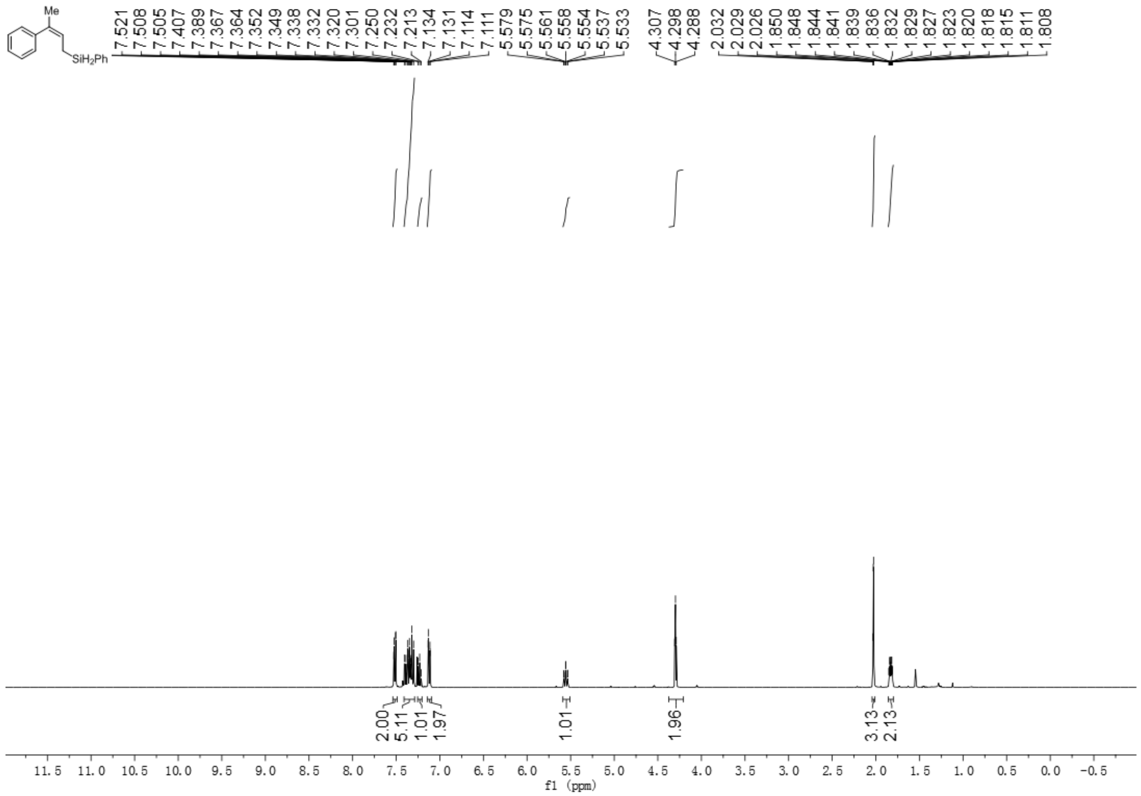

Supplementary Figure 43. ^{1}H NMR spectra for compound 3a

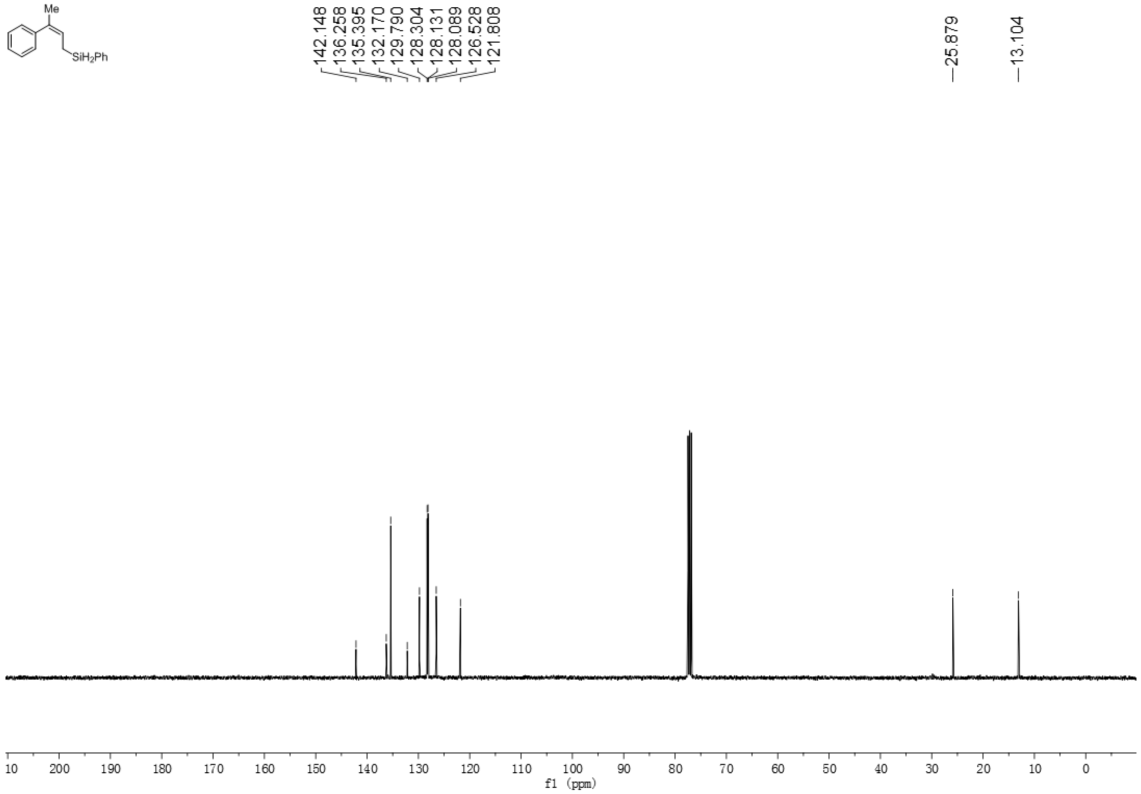

Supplementary Figure 44. ^{13}C NMR spectra for compound 3a

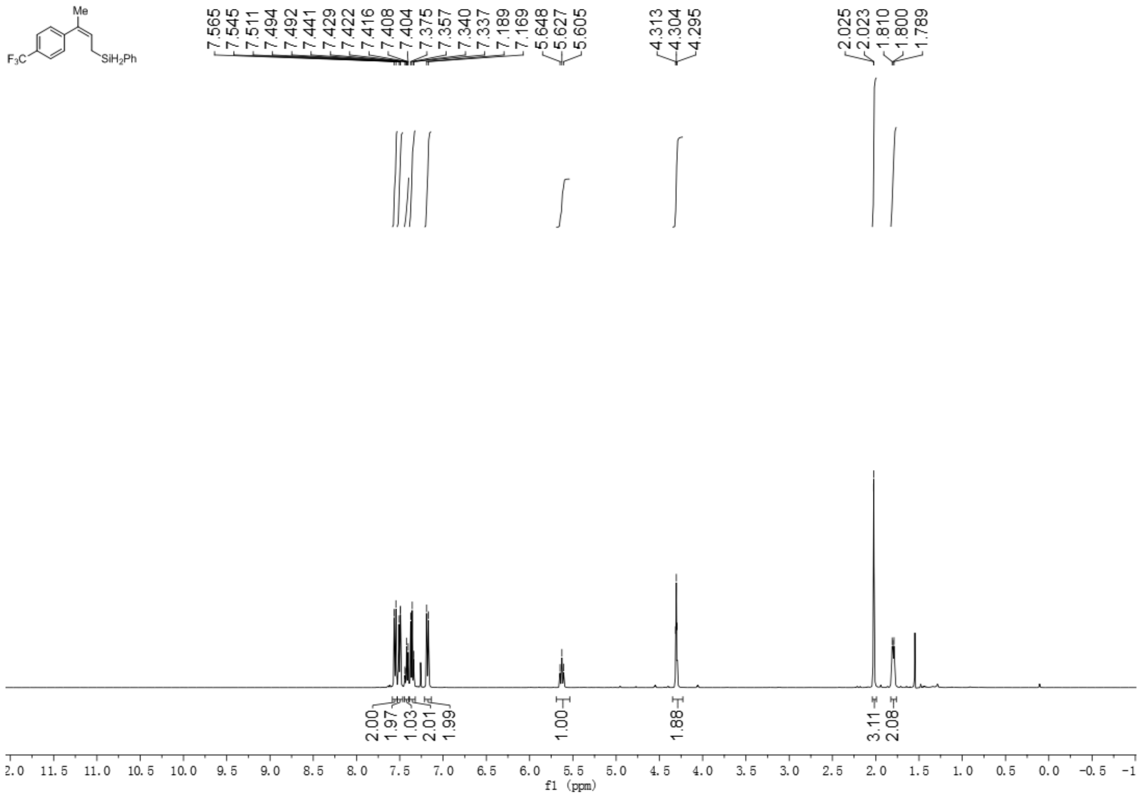

Supplementary Figure 45. ^{1}H NMR spectra for compound 3b

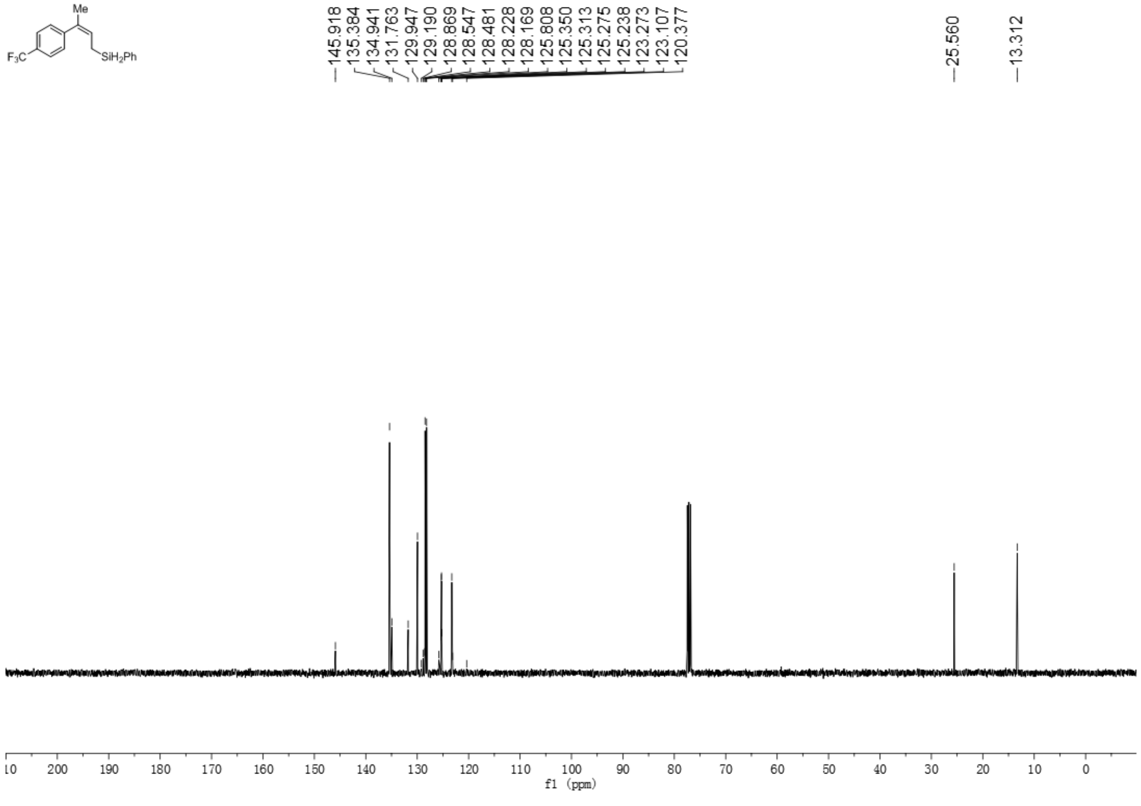

Supplementary Figure 46. ^{13}C NMR spectra for compound 3b

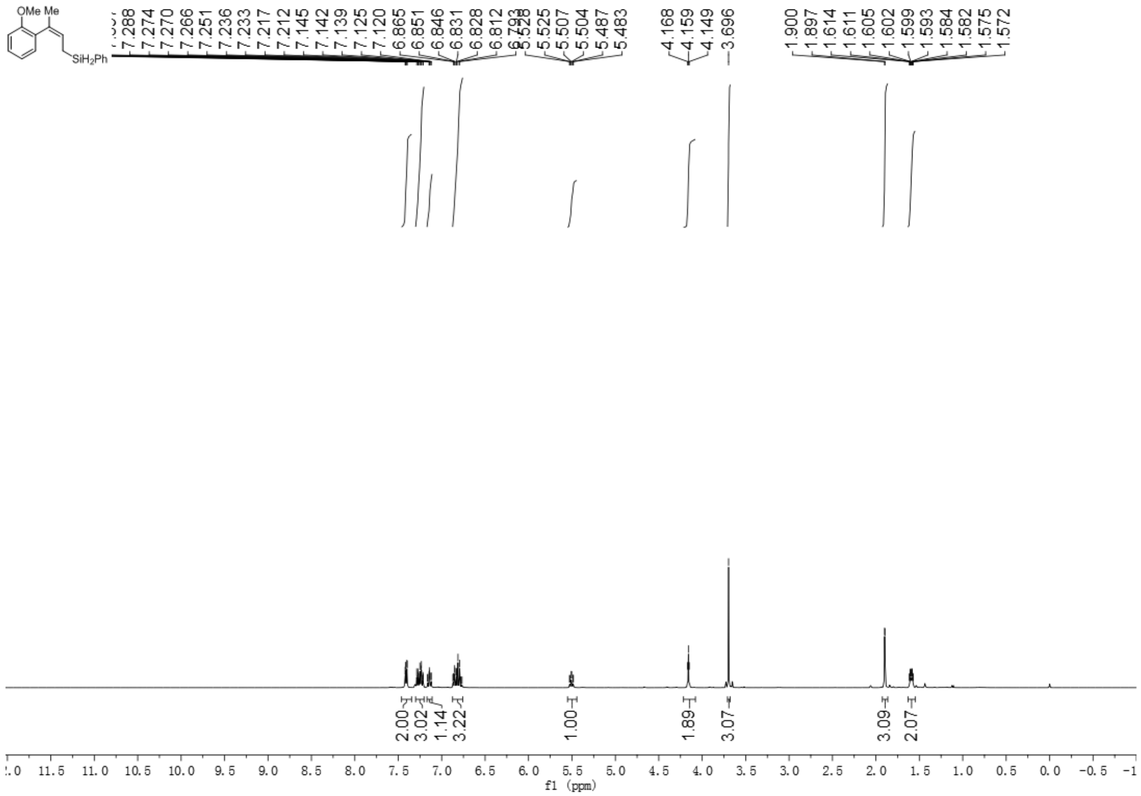

Supplementary Figure 47. ^{1}H NMR spectra for compound 3c

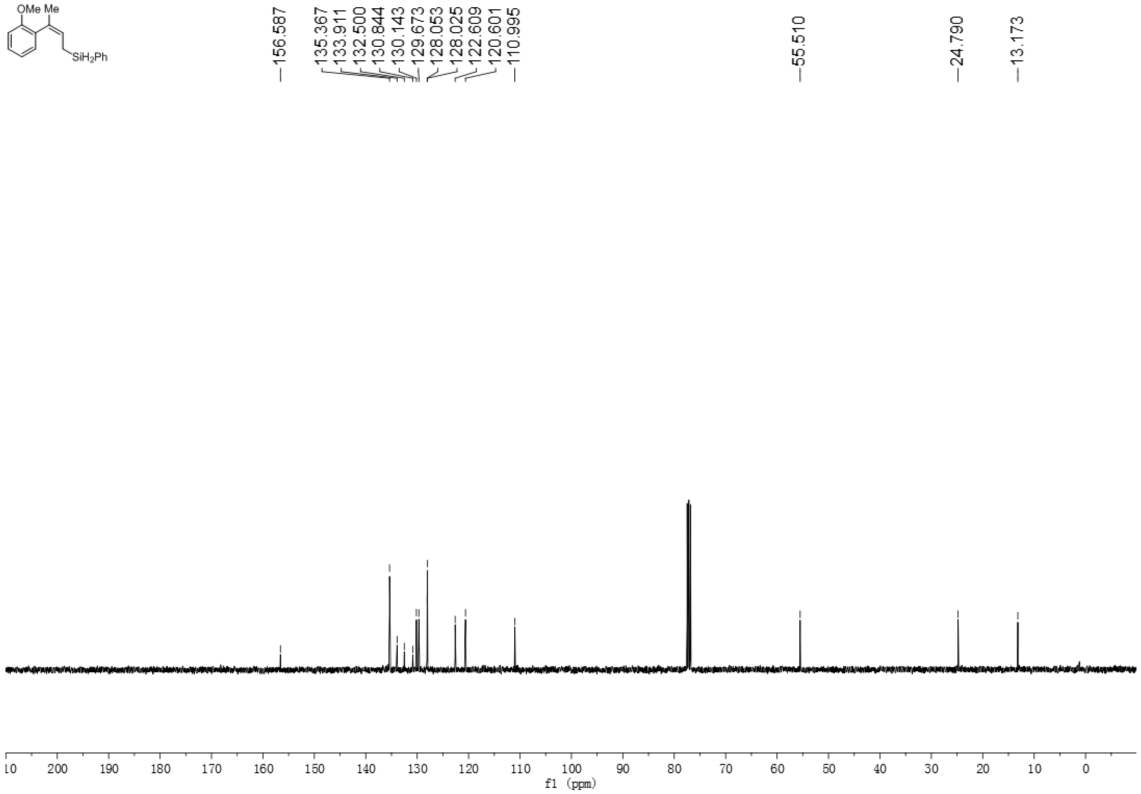

Supplementary Figure 48. ^{13}C NMR spectra for compound 3c

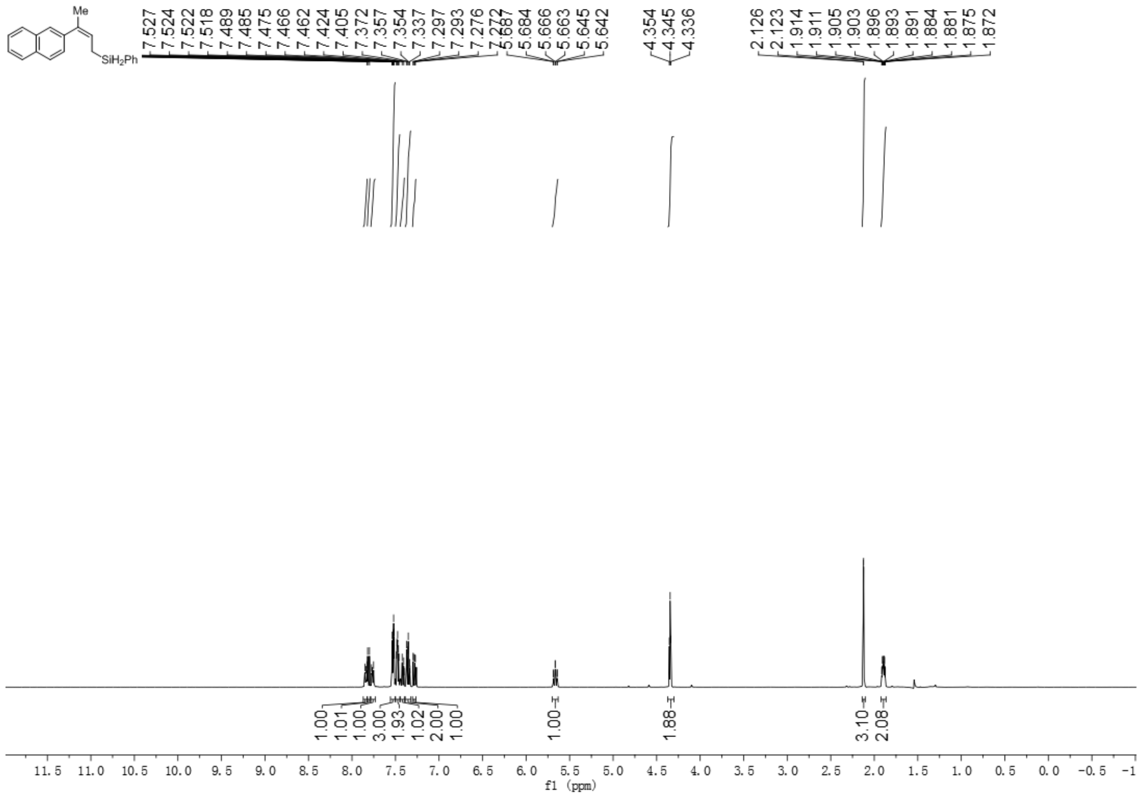

Supplementary Figure 49. ^{1}H NMR spectra for compound 3d

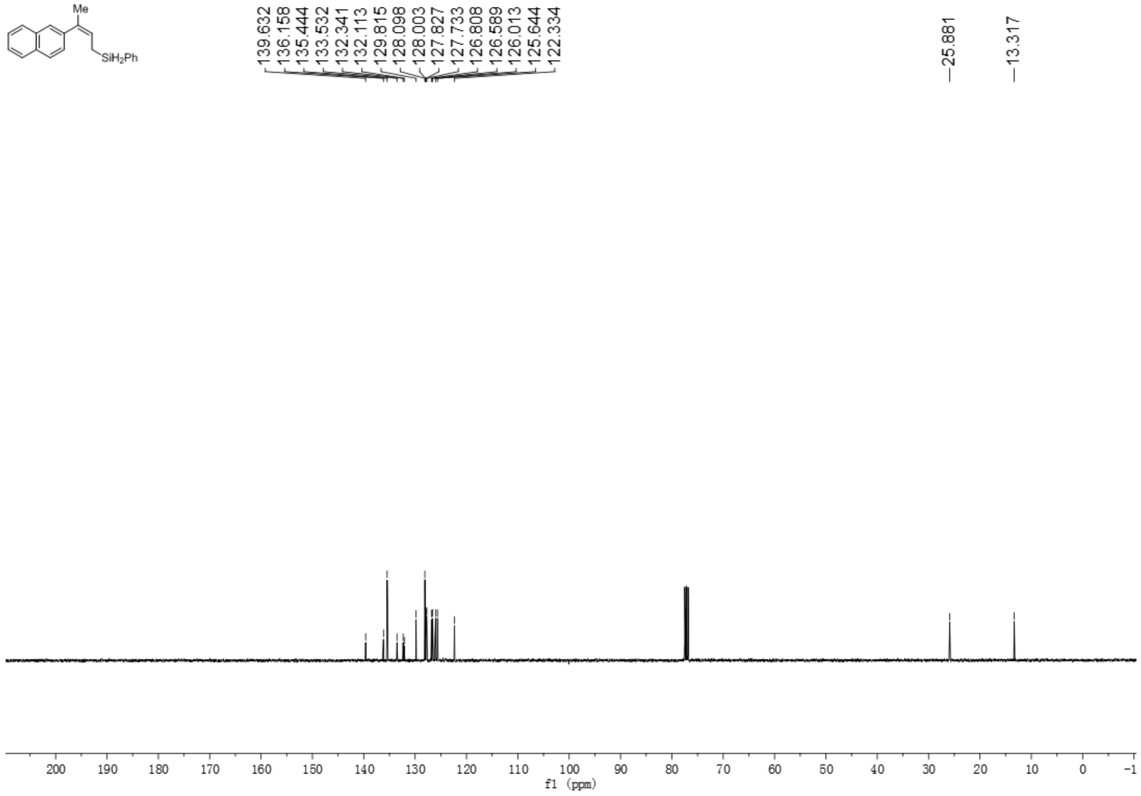

Supplementary Figure 50. ^{13}C NMR spectra for compound 3d

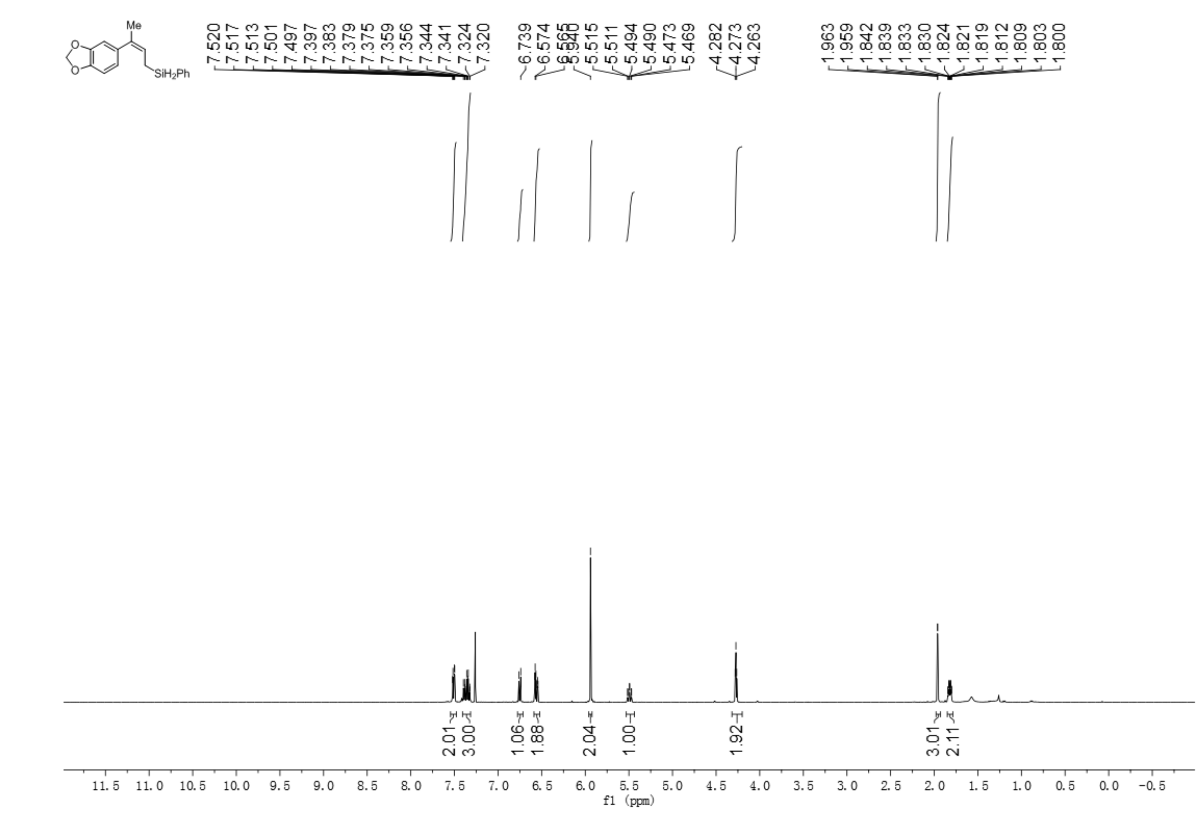

Supplementary Figure 51. ^{1}H NMR spectra for compound 3e

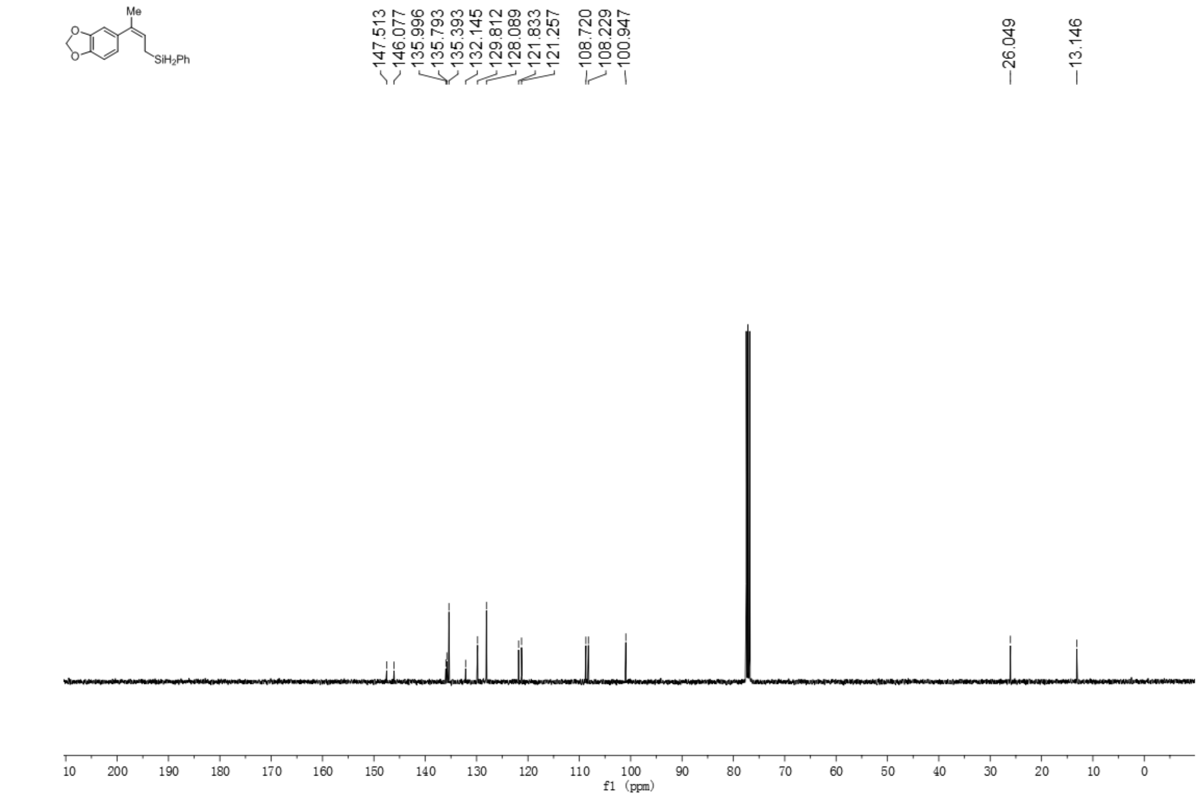

Supplementary Figure 52. ^{13}C NMR spectra for compound 3e

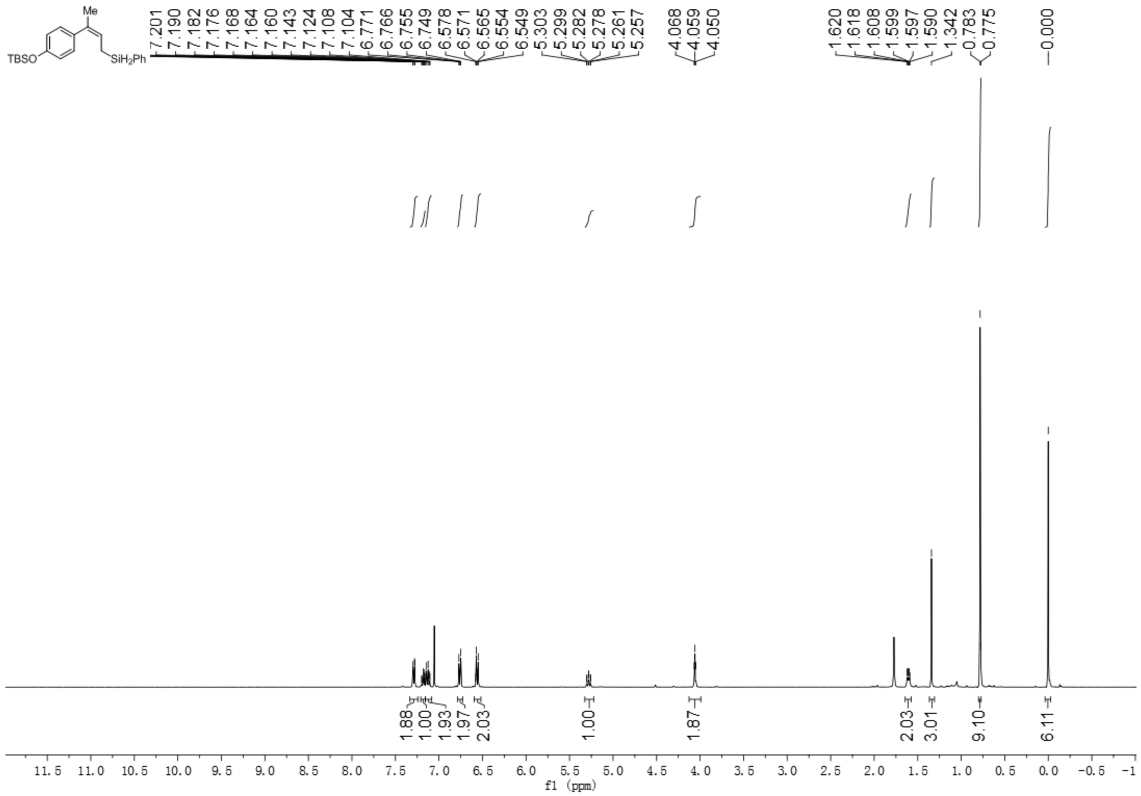

Supplementary Figure 53. ^{1}H NMR spectra for compound 3f

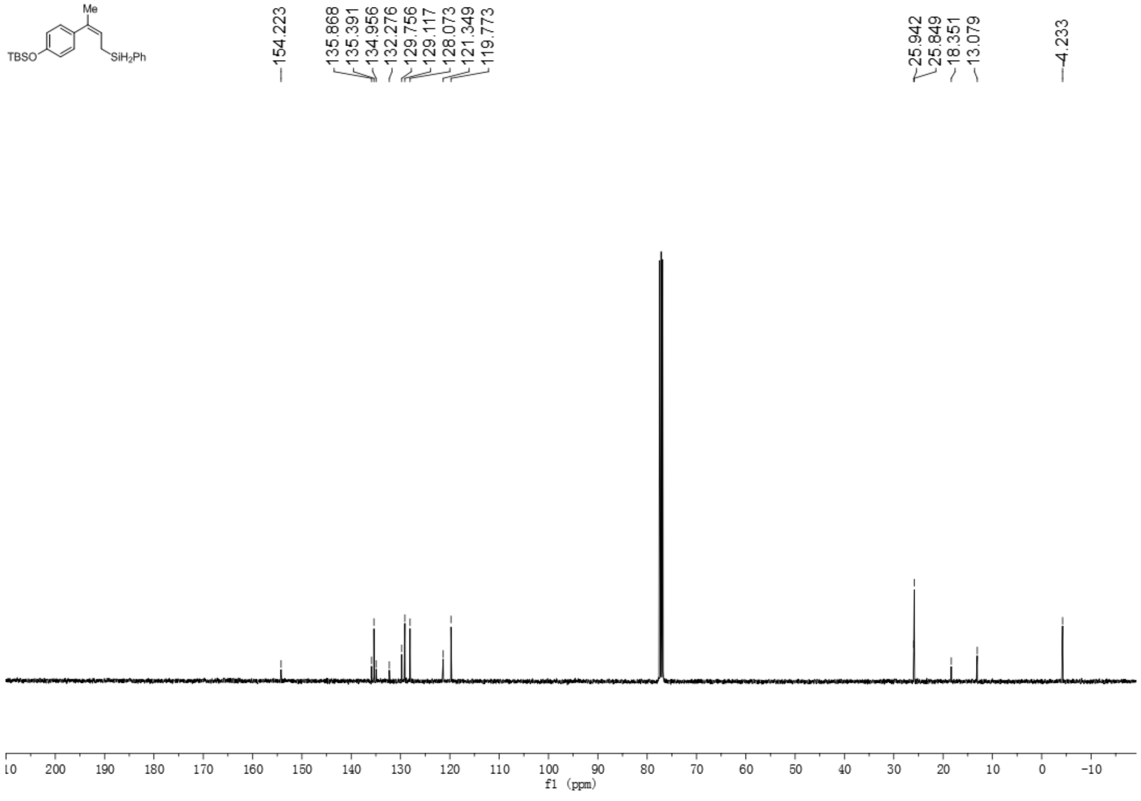

Supplementary Figure 54. ^{13}C NMR spectra for compound 3f

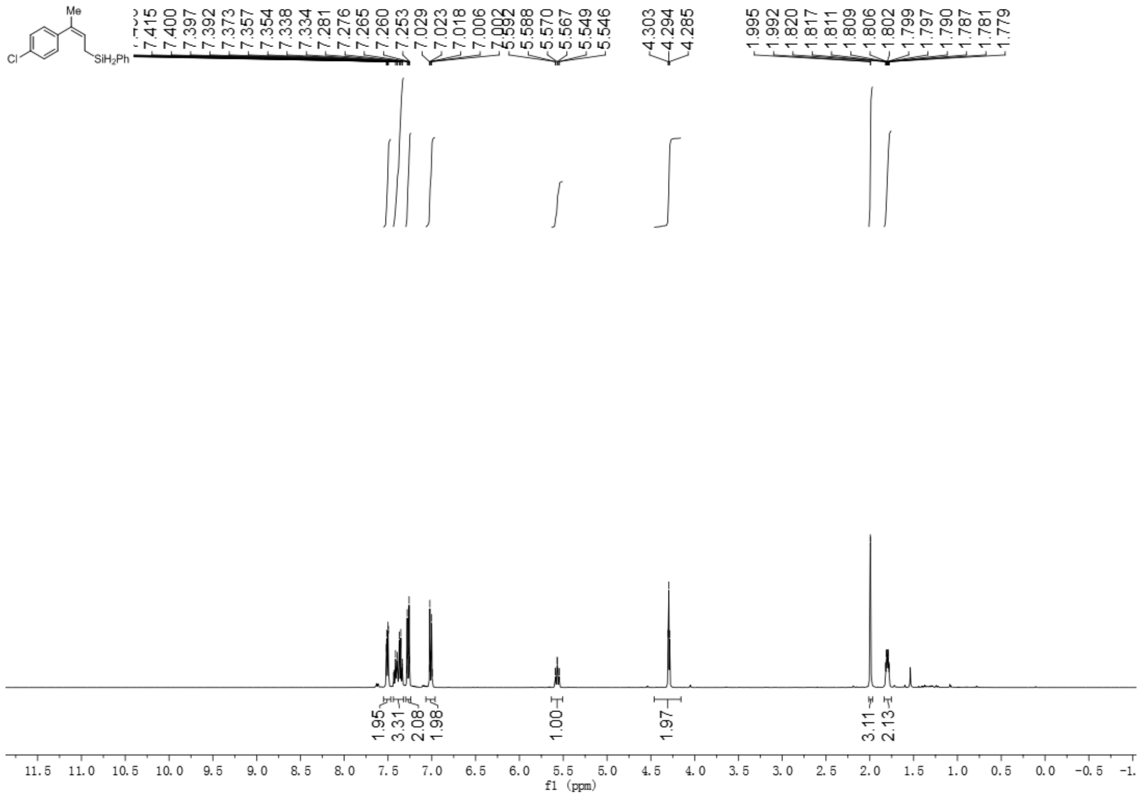

Supplementary Figure 55. ^{1}H NMR spectra for compound 3g

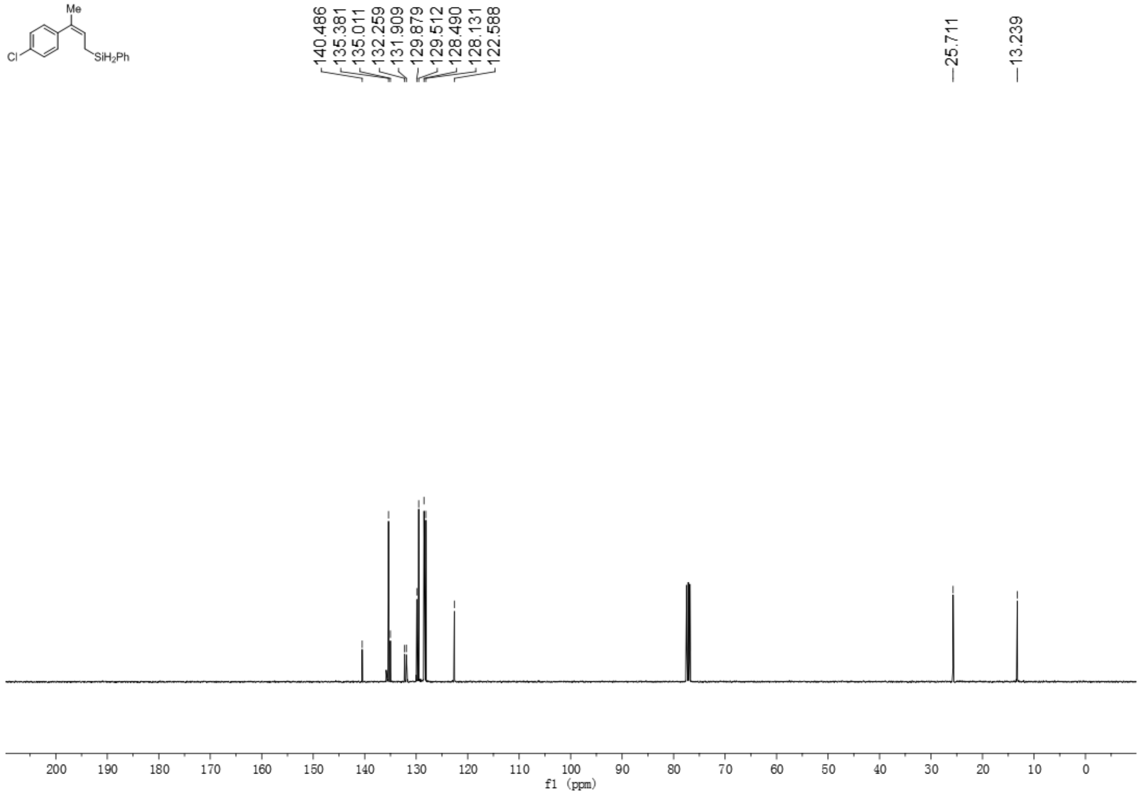

Supplementary Figure 56. ^{13}C NMR spectra for compound 3g

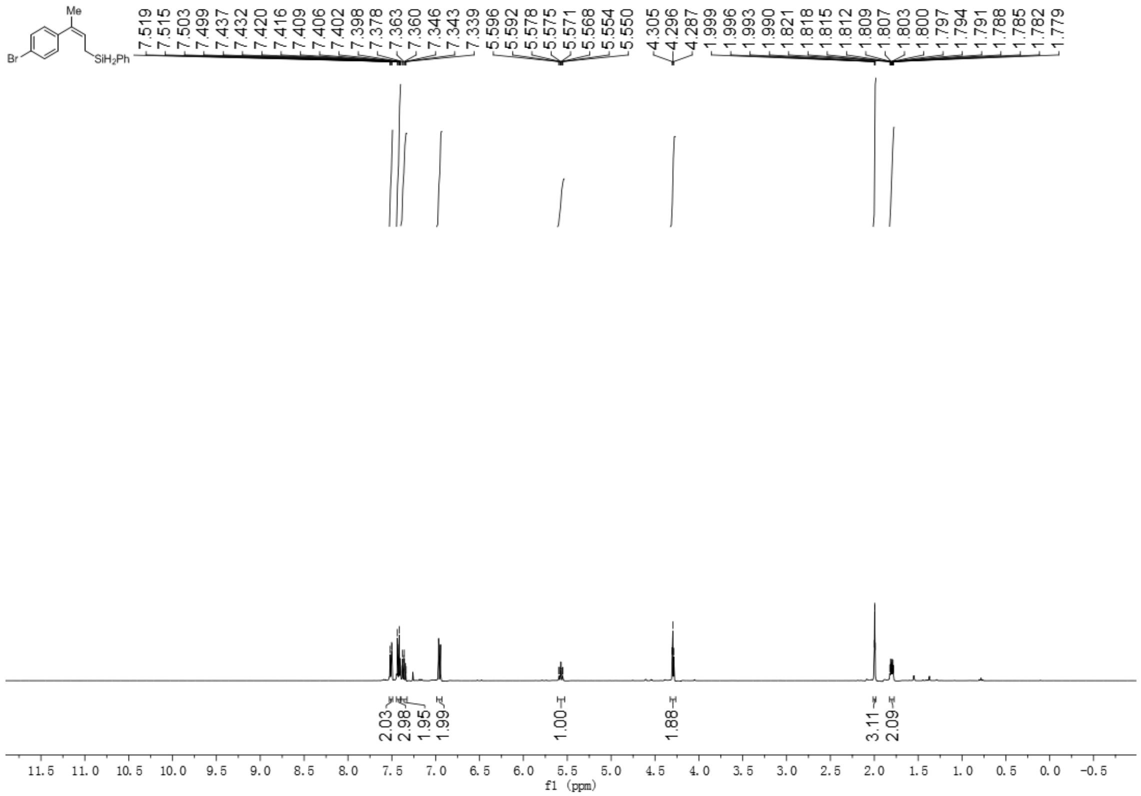

Supplementary Figure 57. ^{1}H NMR spectra for compound 3h

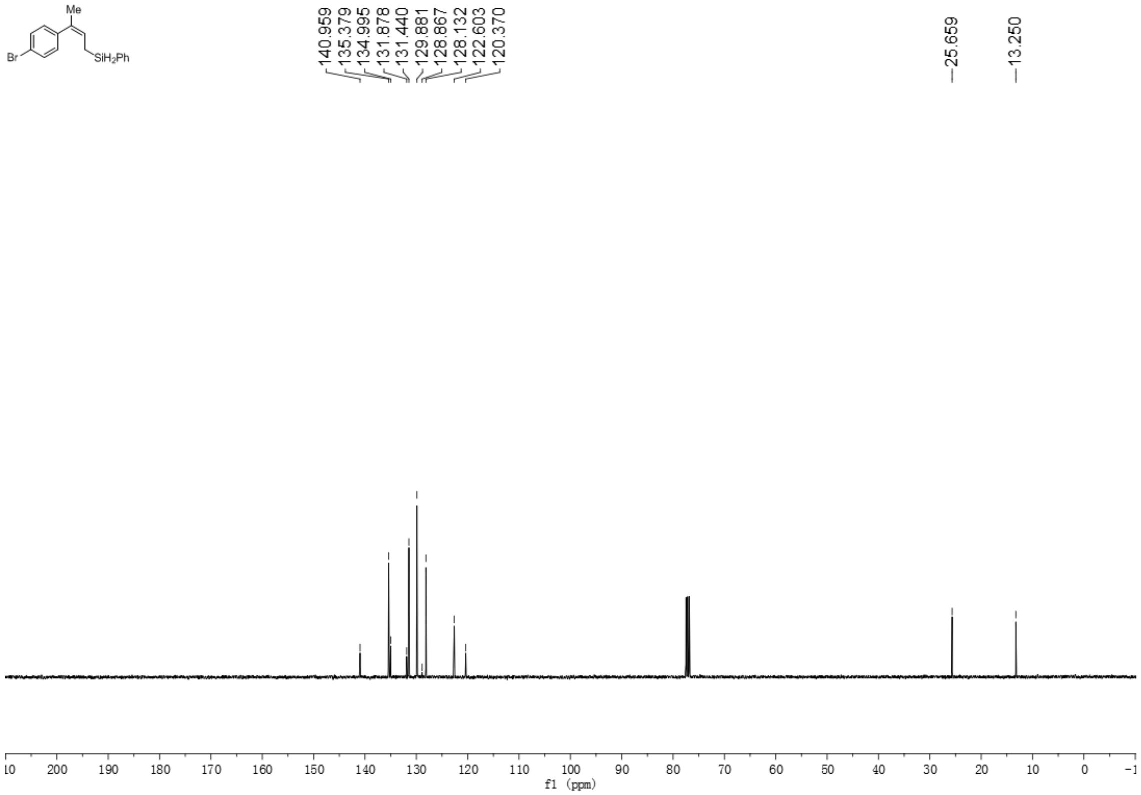

Supplementary Figure 58. ^{13}C NMR spectra for compound 3h

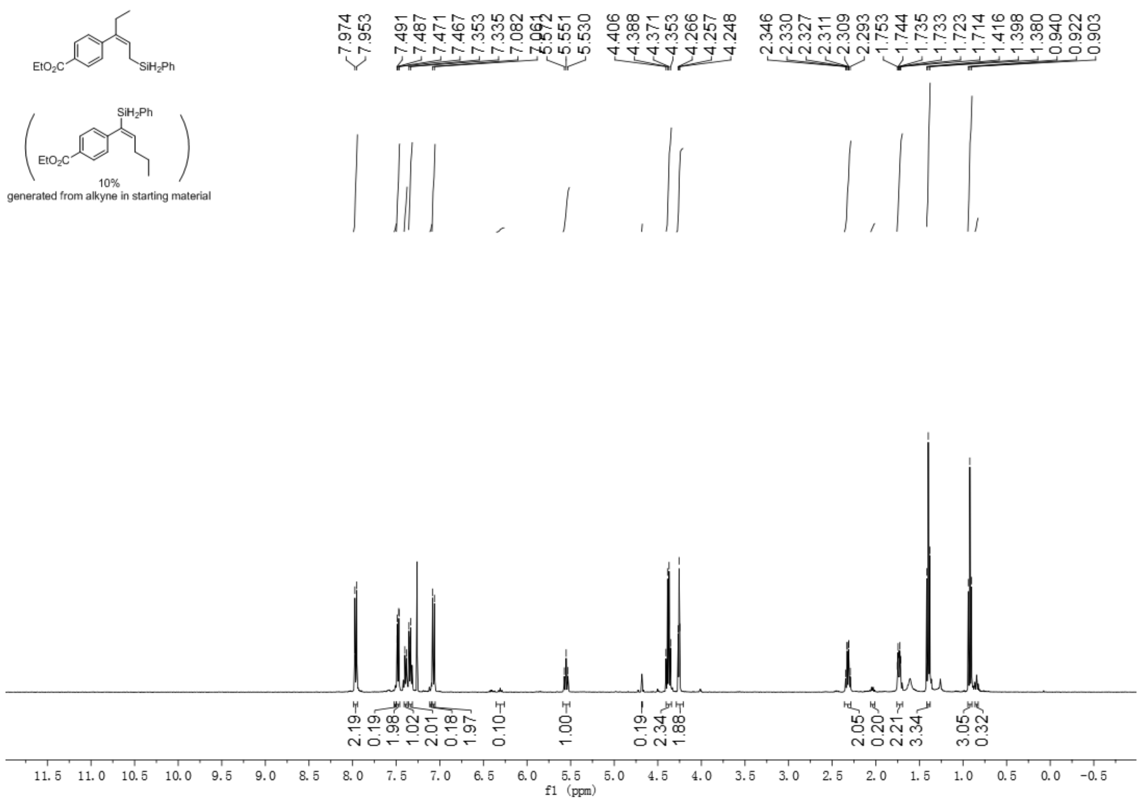

Supplementary Figure 59. ^{1}H NMR spectra for compound 3i

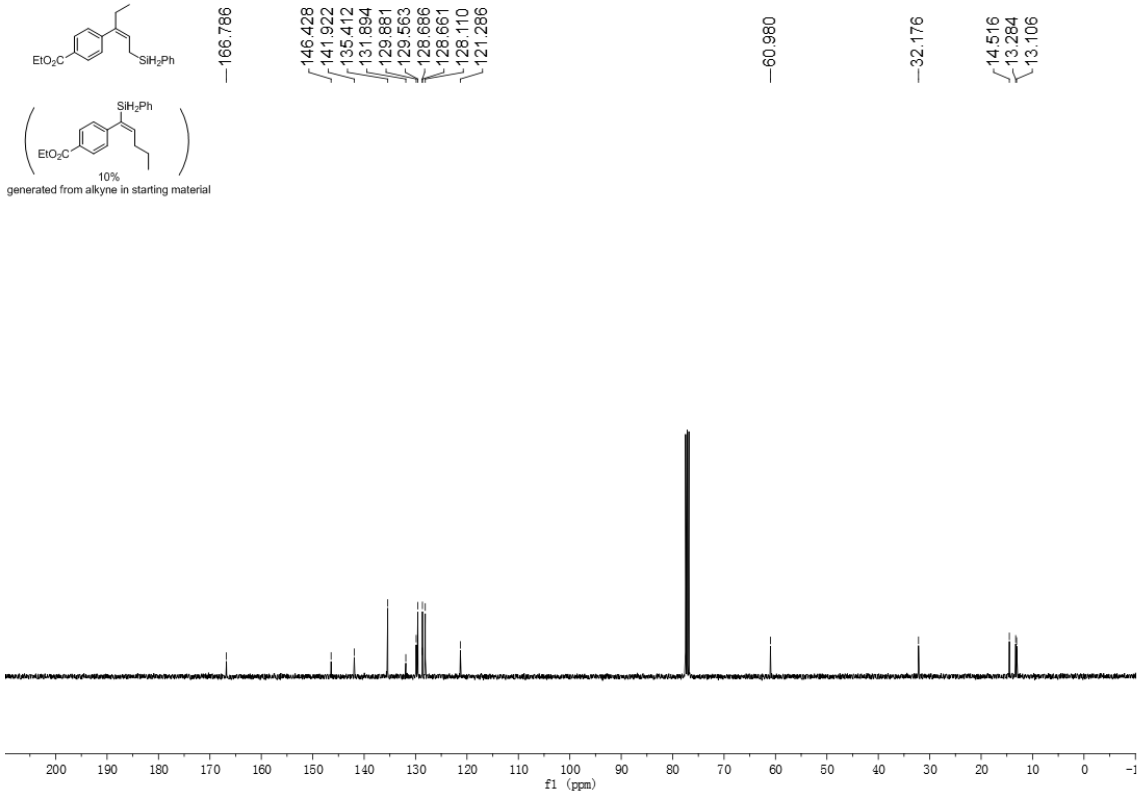

Supplementary Figure 60. ^{13}C NMR spectra for compound 3i

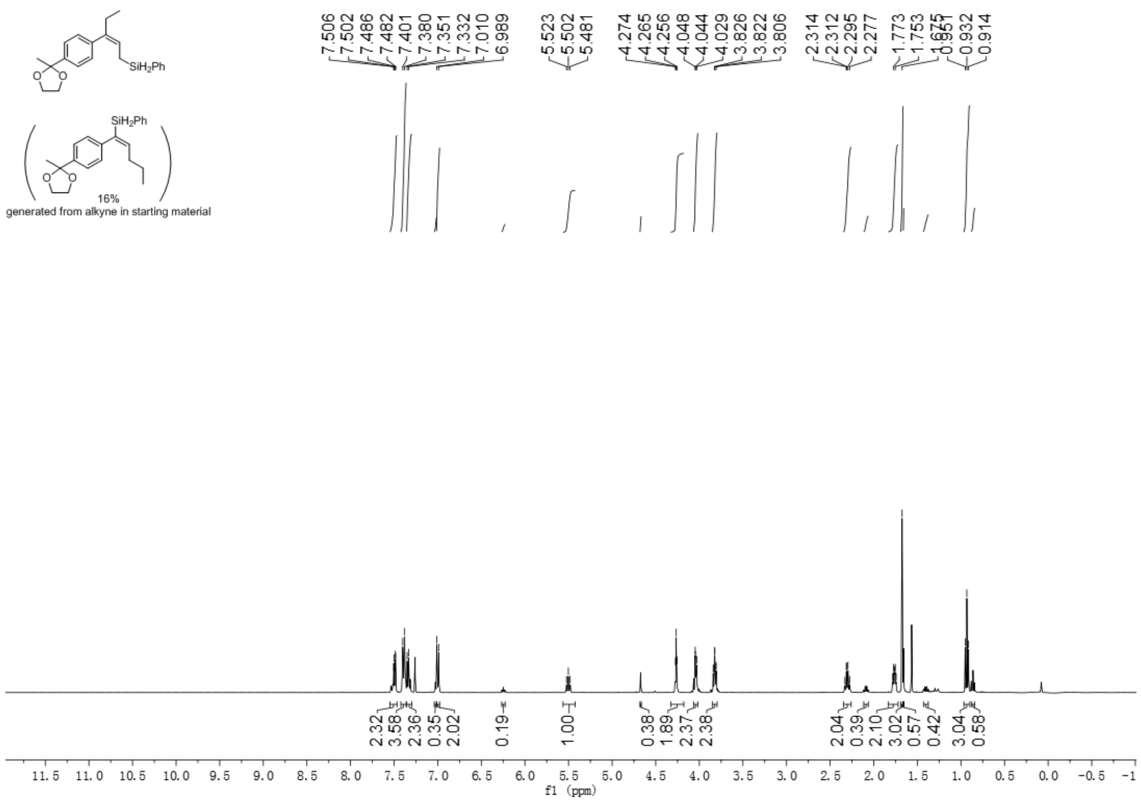

Supplementary Figure 61. ^{1}H NMR spectra for compound 3j

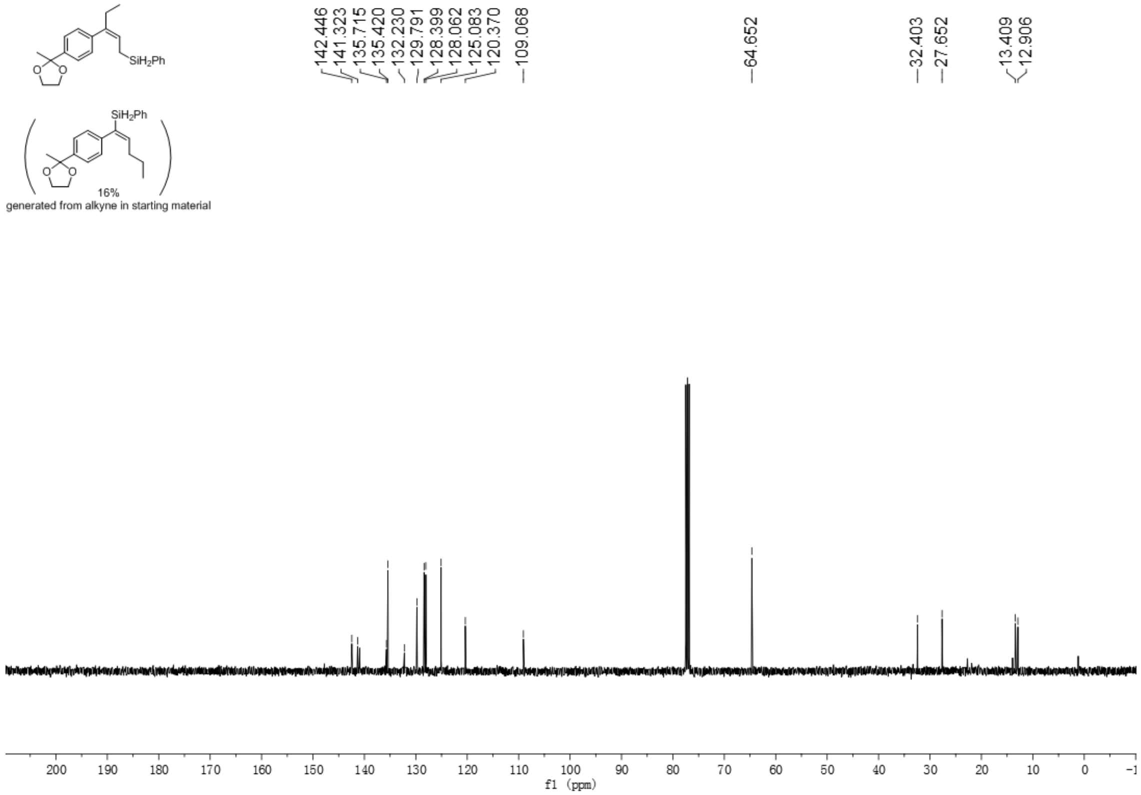

Supplementary Figure 62. ^{13}C NMR spectra for compound 3j

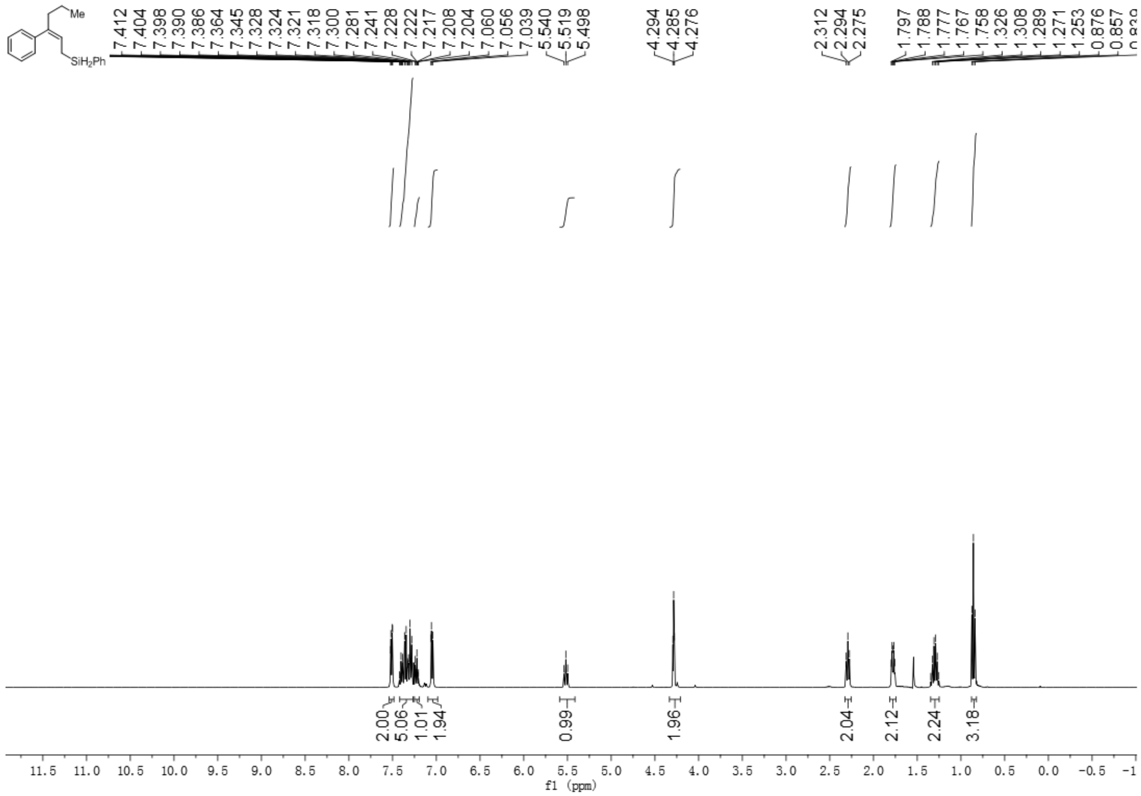

Supplementary Figure 63. ^{1}H NMR spectra for compound 3k

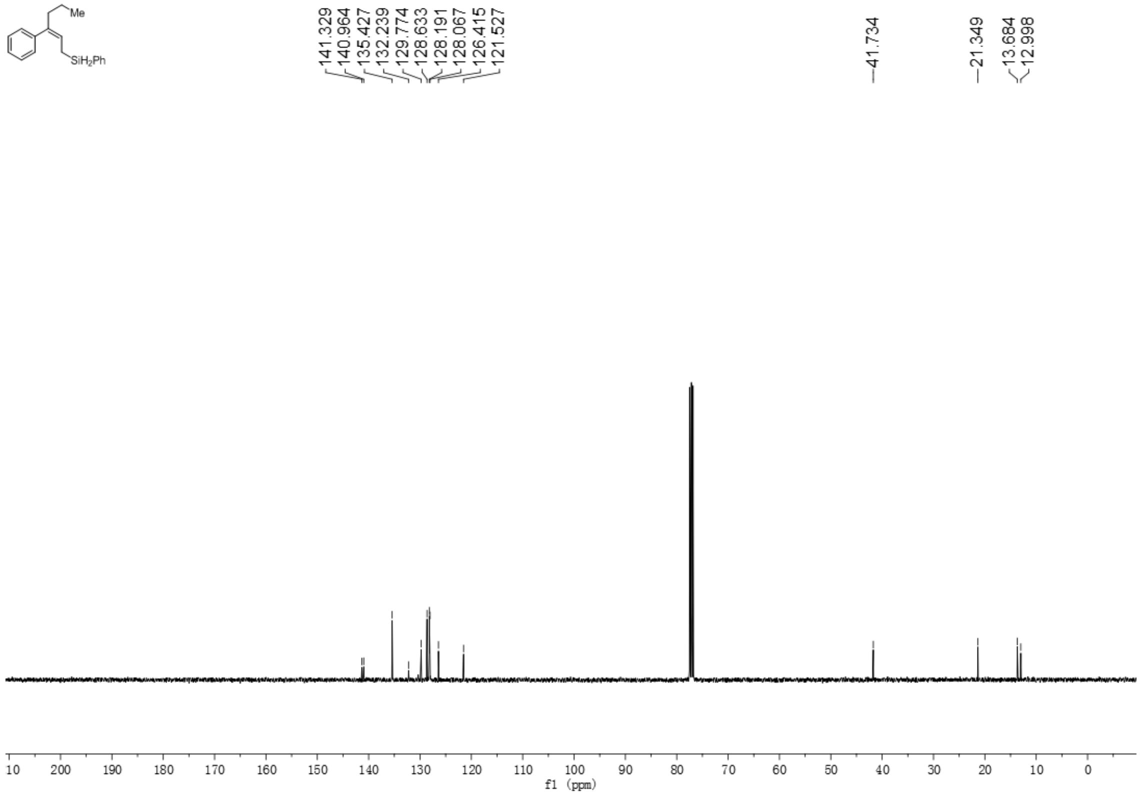

Supplementary Figure 64. ^{13}C NMR spectra for compound 3k

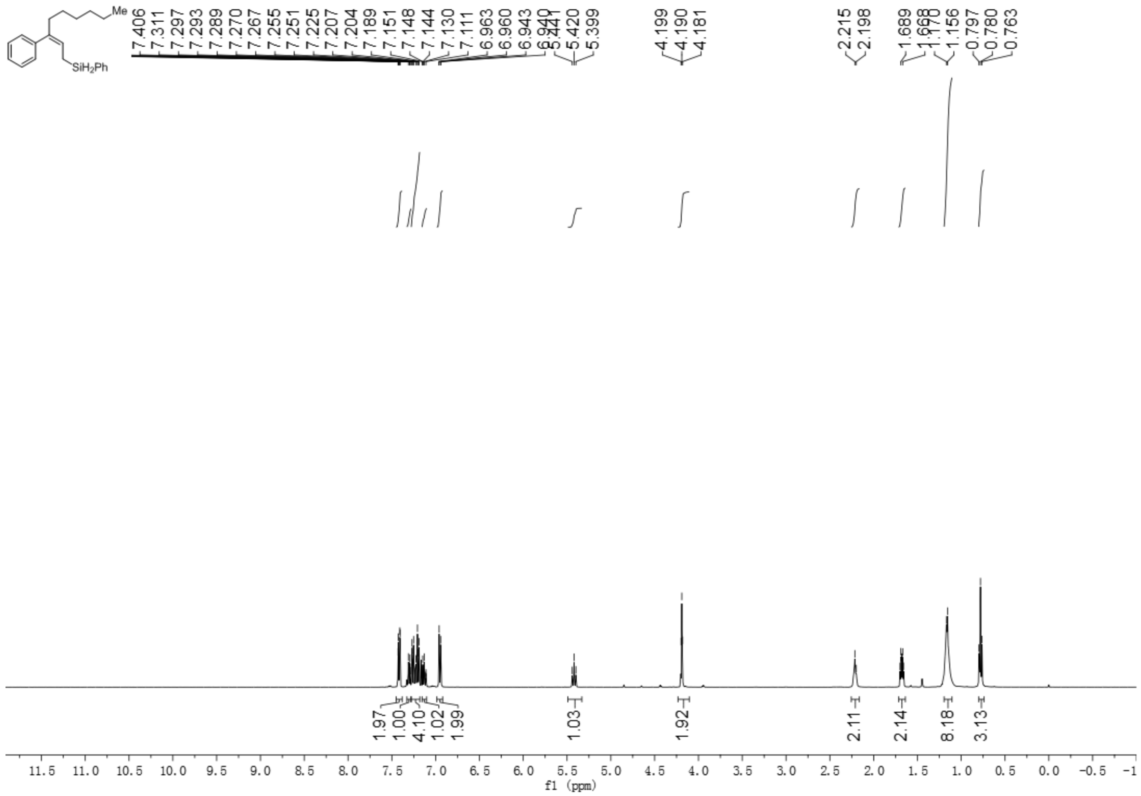

Supplementary Figure 65. ^{1}H NMR spectra for compound 3l

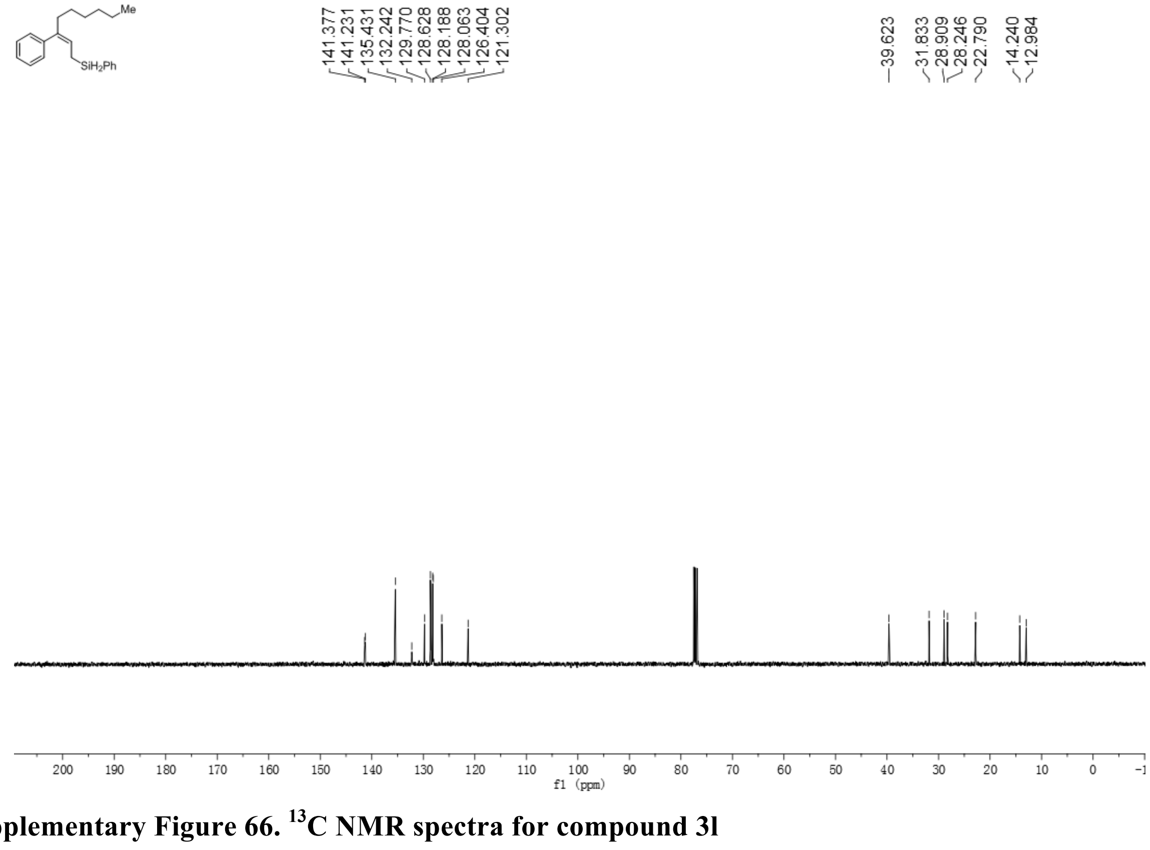

Supplementary Figure 66. ^{13}C NMR spectra for compound 3l

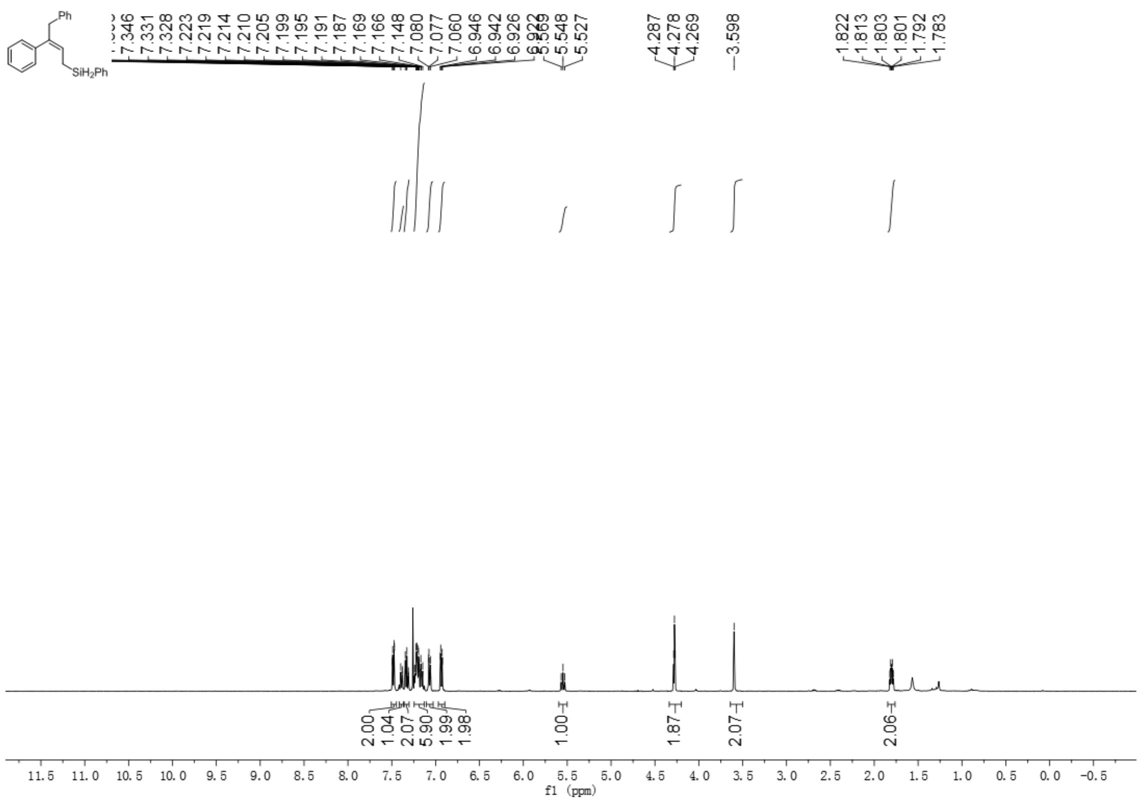

Supplementary Figure 67. ^{1}H NMR spectra for compound 3m

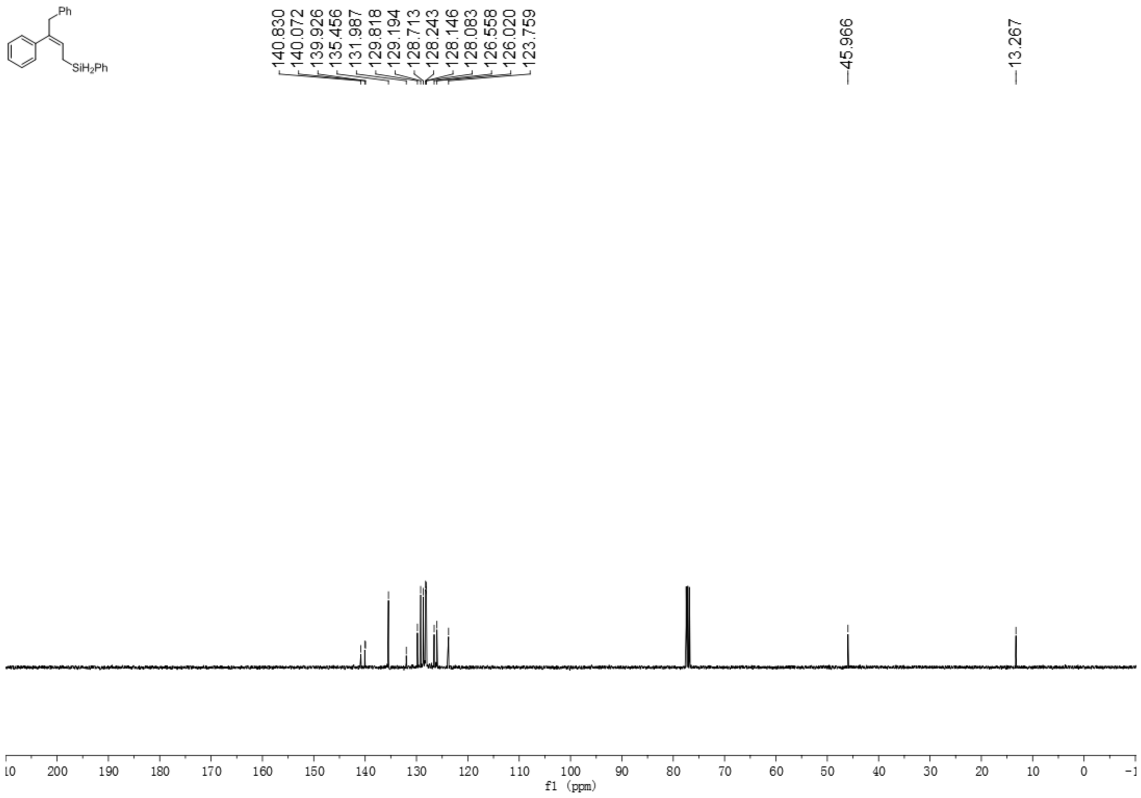

Supplementary Figure 68. ^{13}C NMR spectra for compound 3m

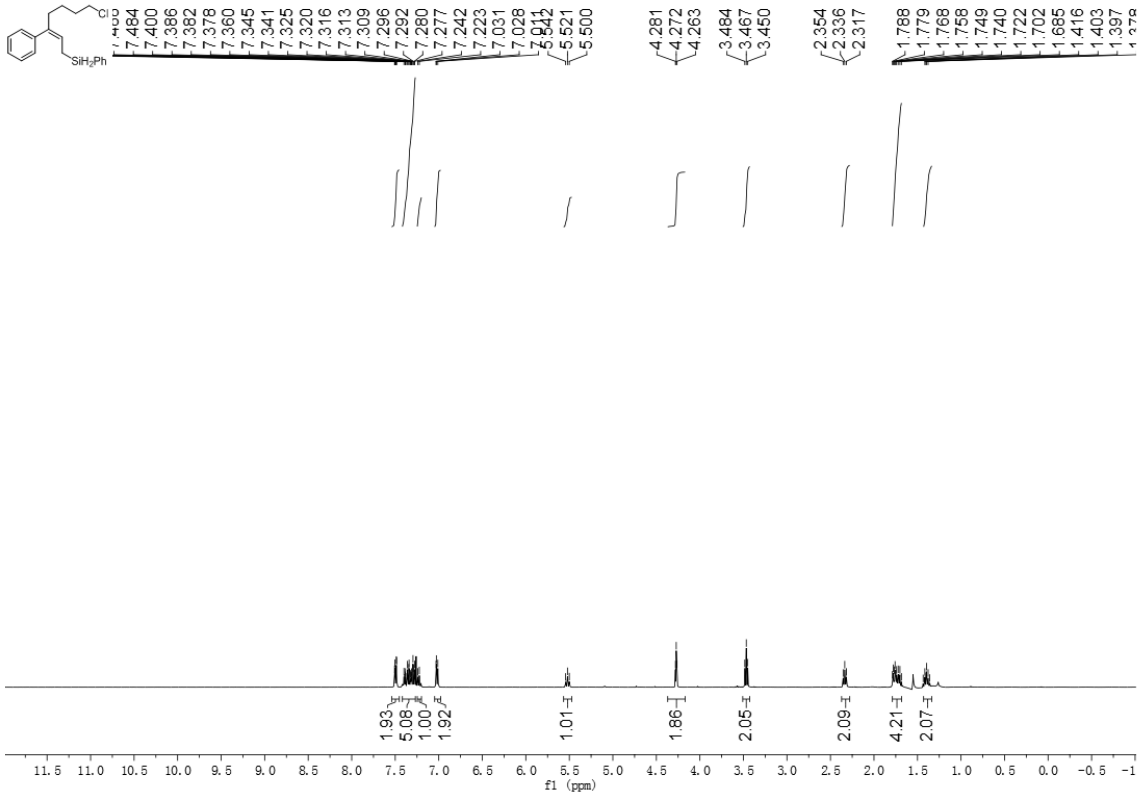

Supplementary Figure 69. ^{1}H NMR spectra for compound 3n

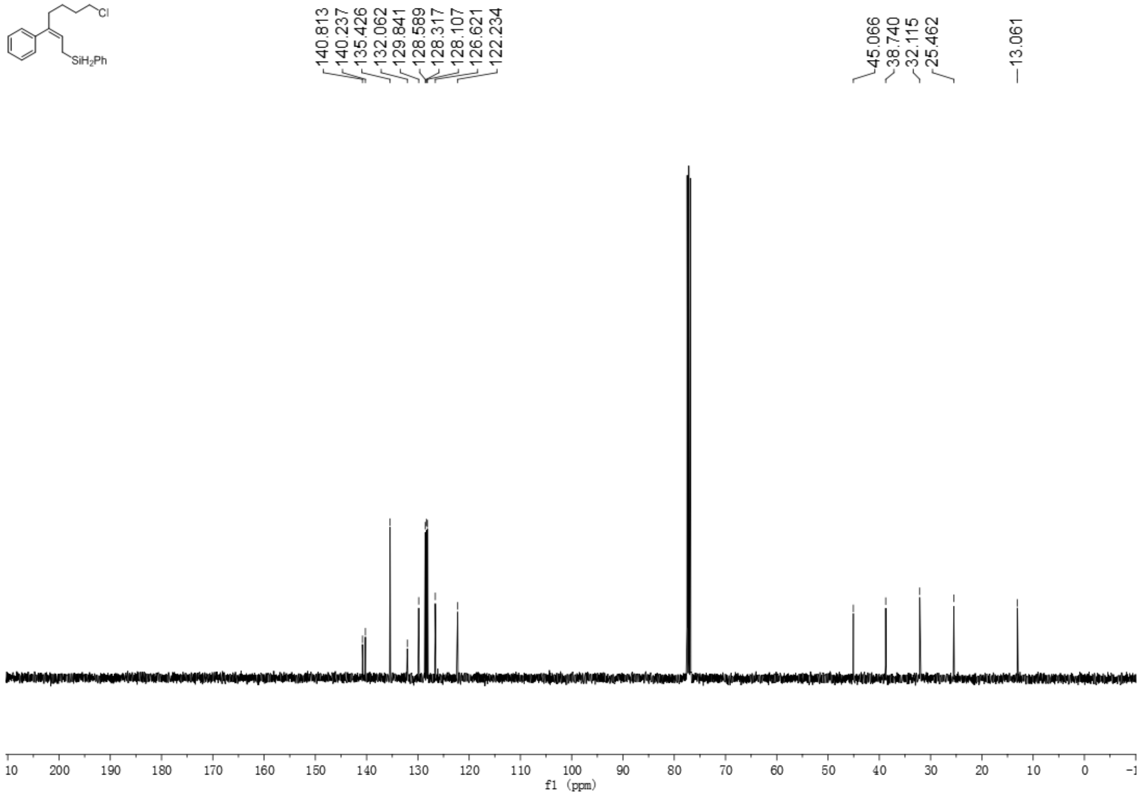

Supplementary Figure 70. ^{13}C NMR spectra for compound 3n

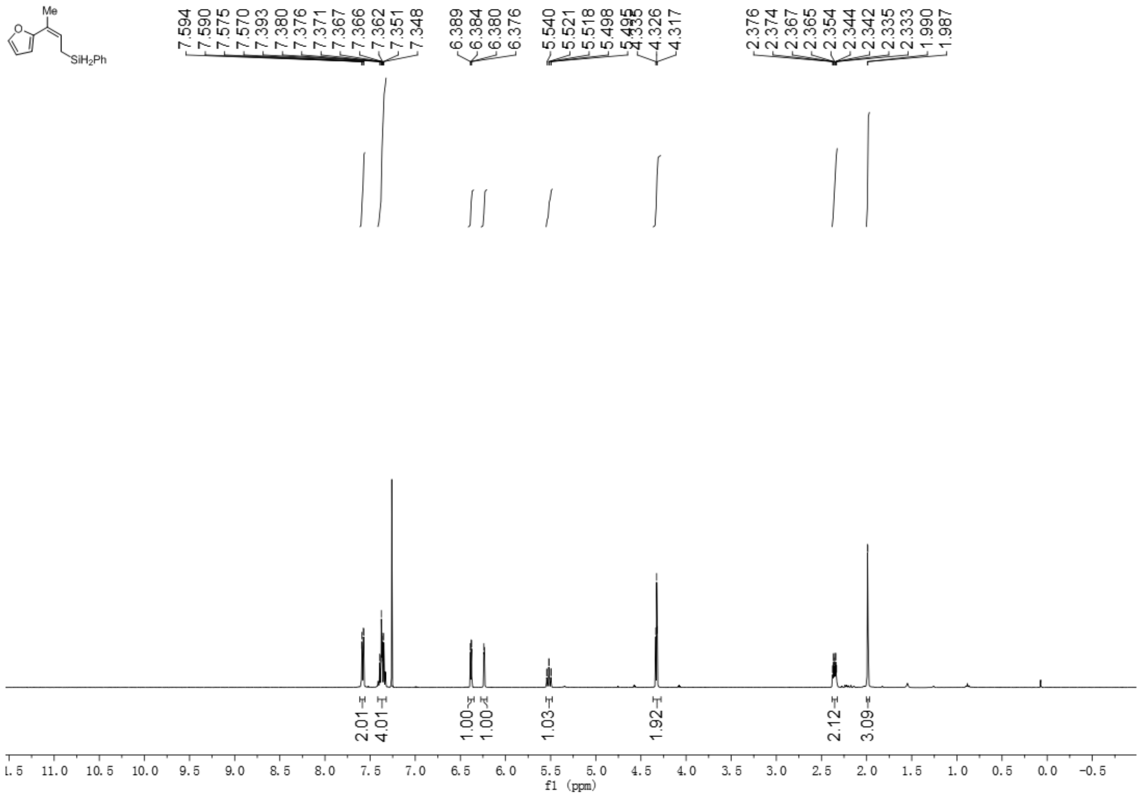

Supplementary Figure 71. ^{1}H NMR spectra for compound 3o

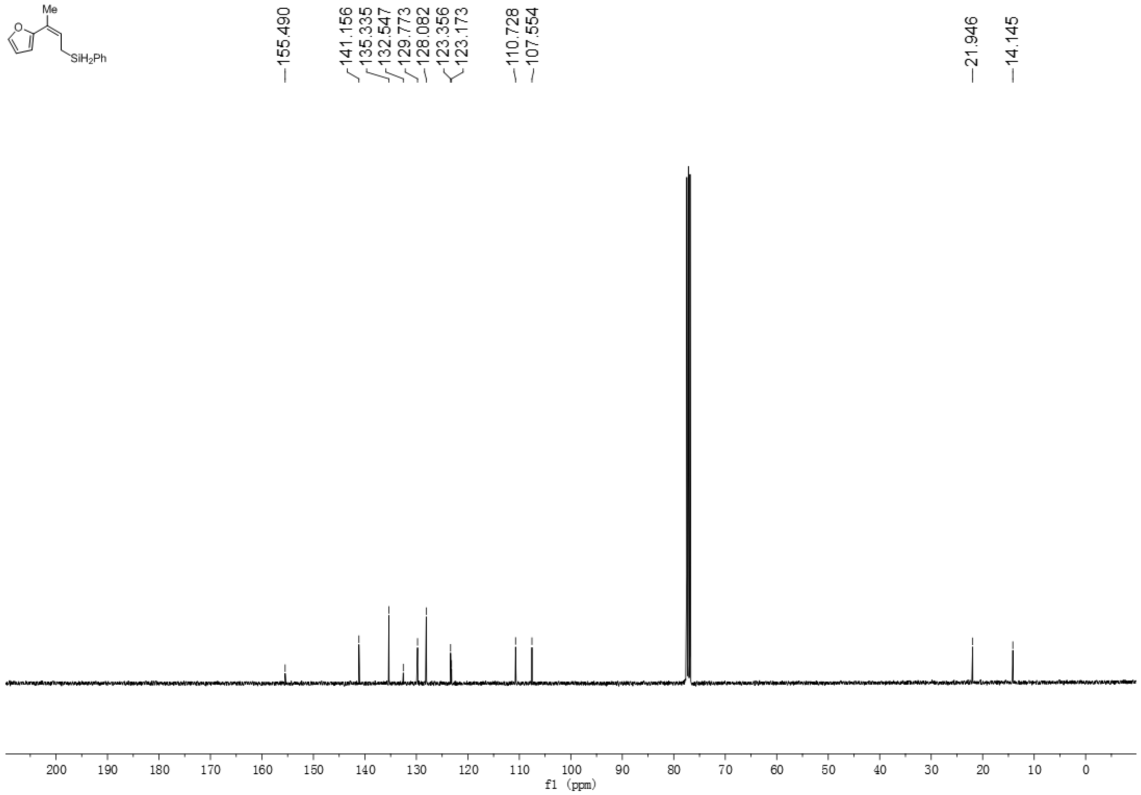

Supplementary Figure 72. ^{13}C NMR spectra for compound 3o

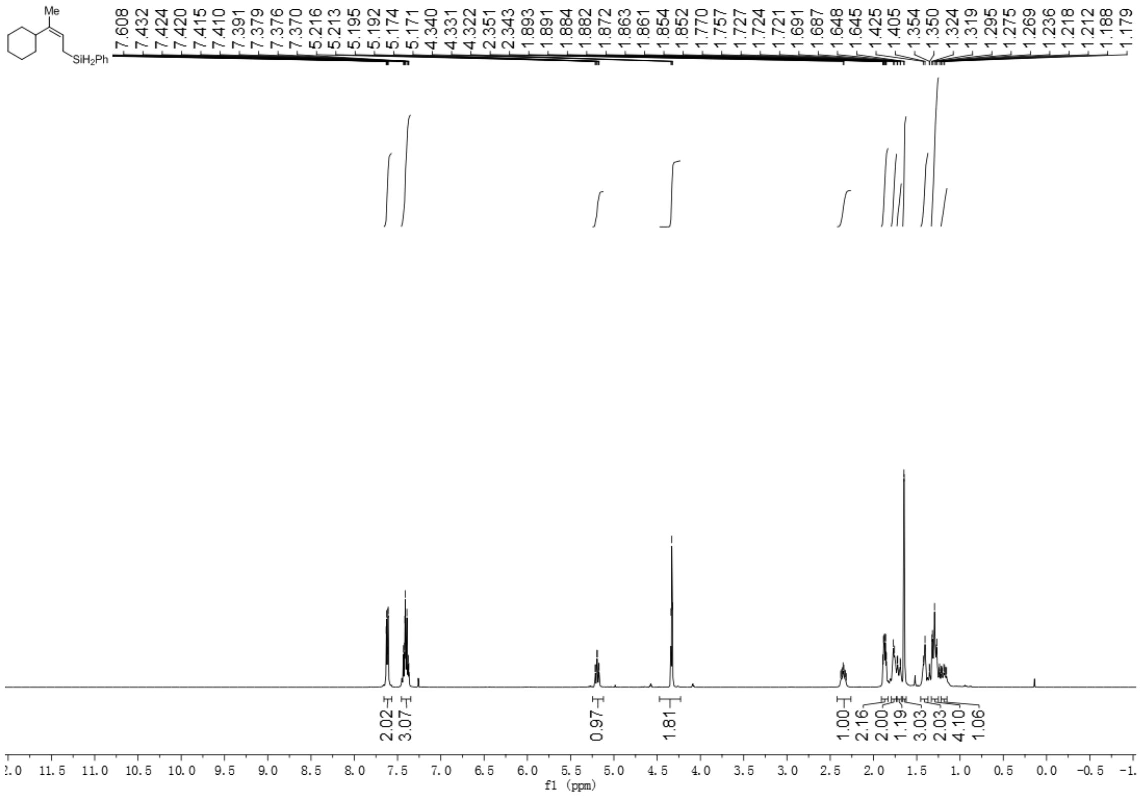

Supplementary Figure 73. ^{1}H NMR spectra for compound 3p

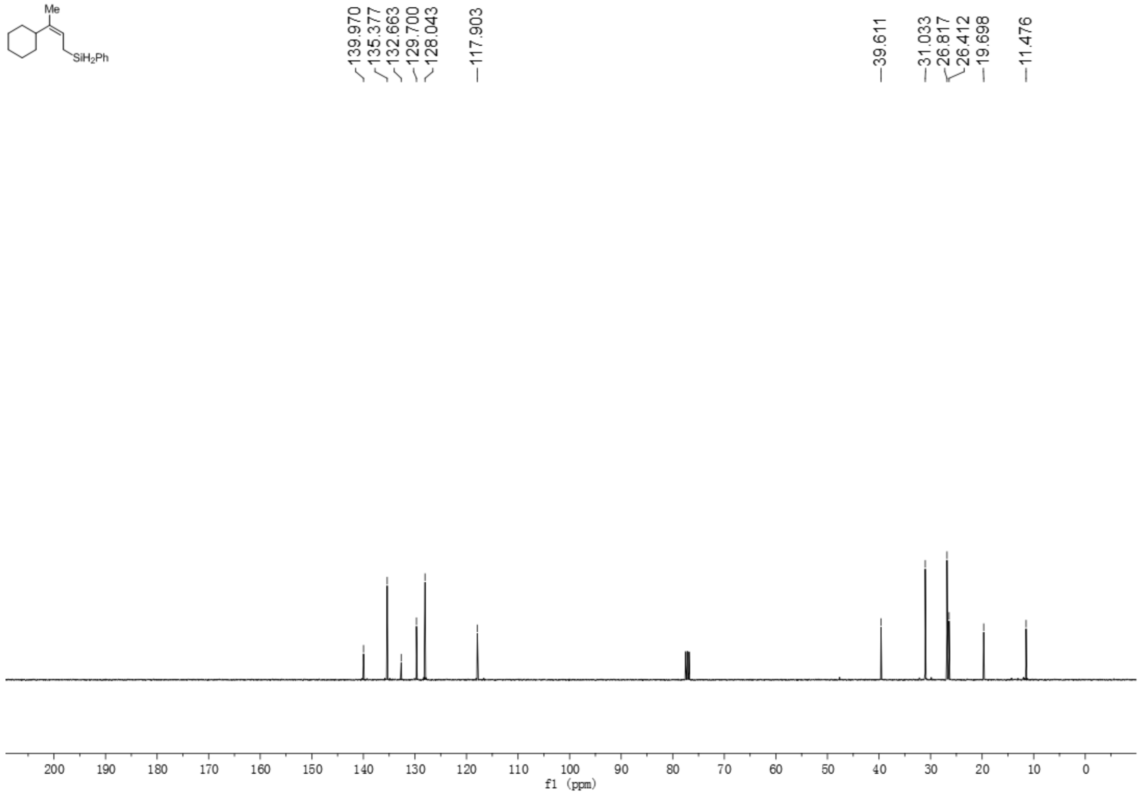

Supplementary Figure 74. ^{13}C NMR spectra for compound 3p

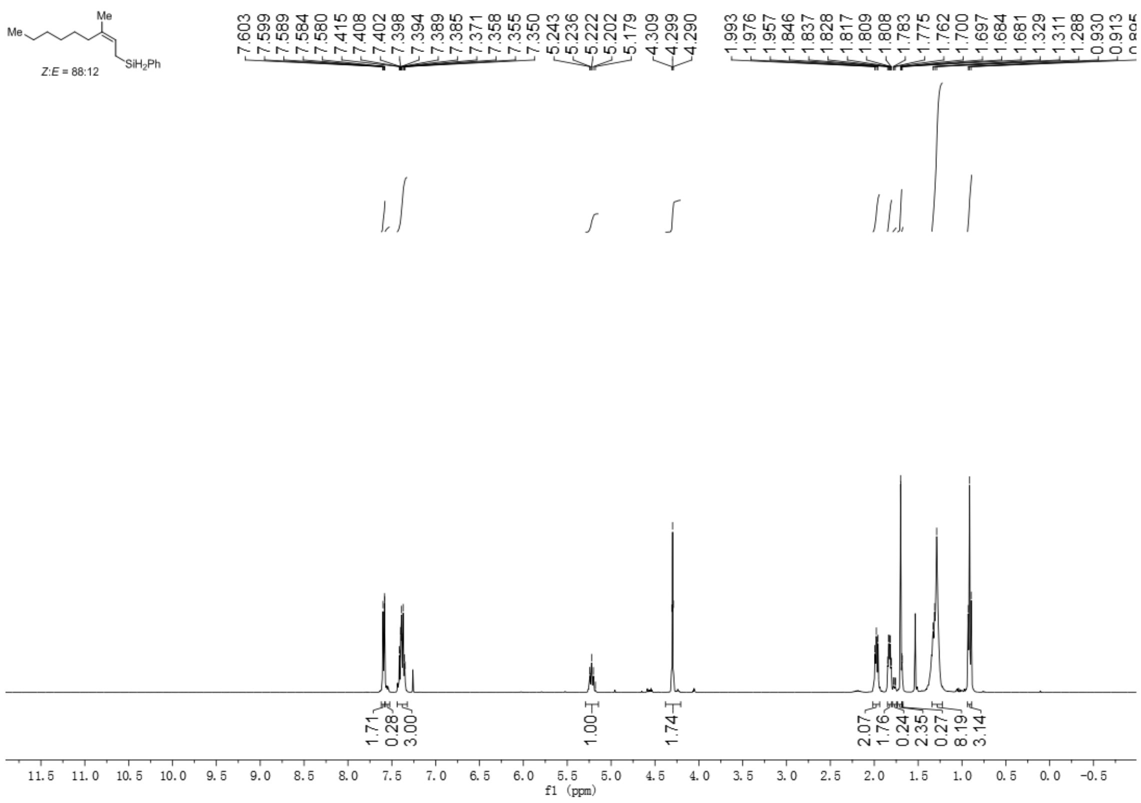

Supplementary Figure 75. ^{1}H NMR spectra for compound 3q

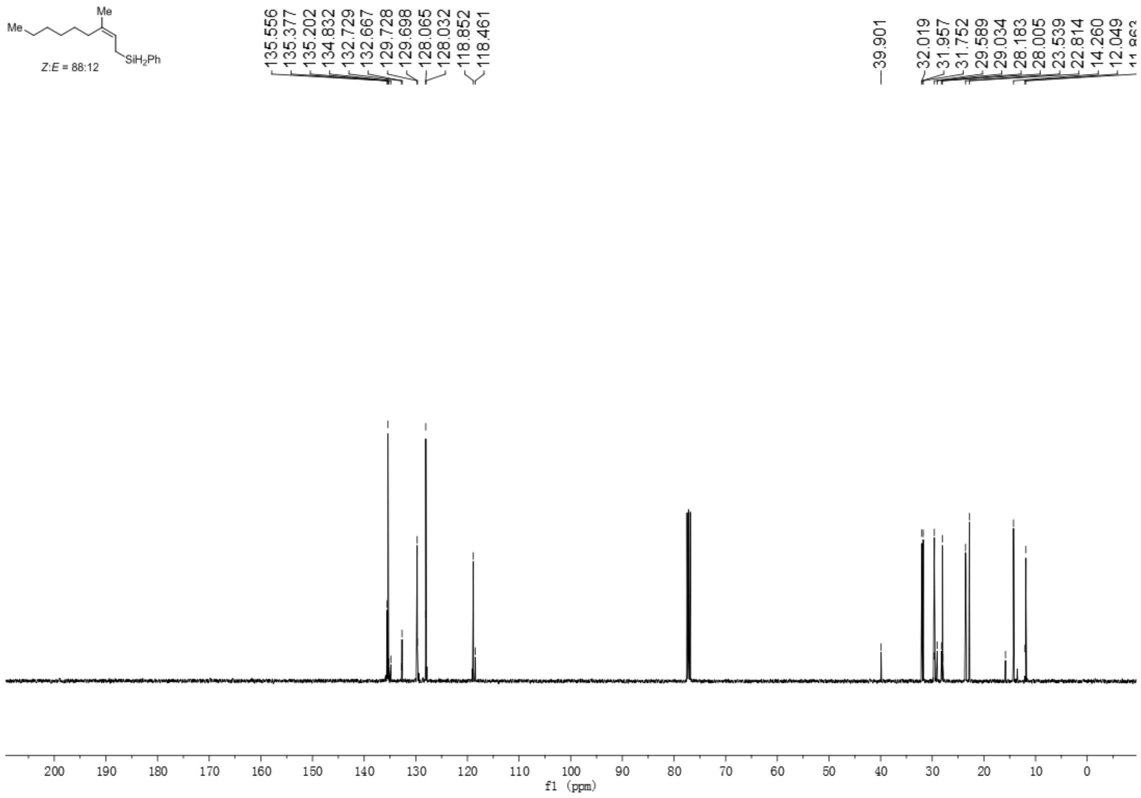

Supplementary Figure 76. ^{13}C NMR spectra for compound 3q

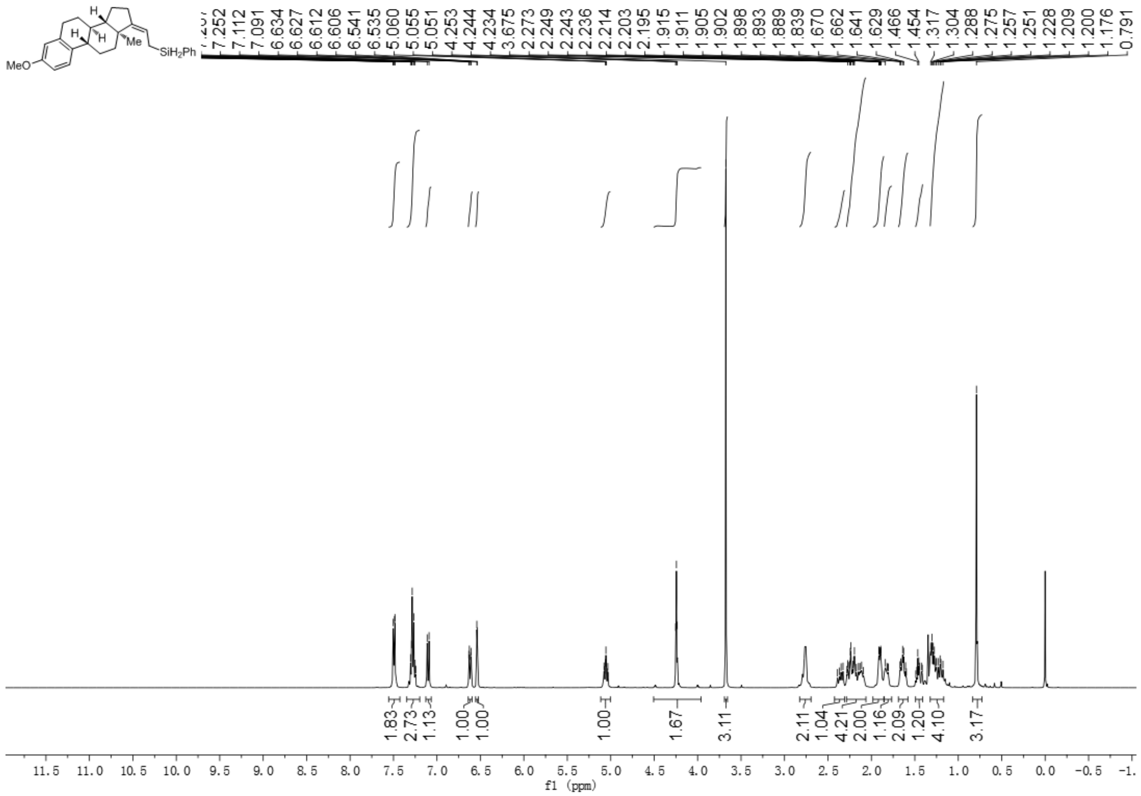

Supplementary Figure 77. ^{1}H NMR spectra for compound 3r

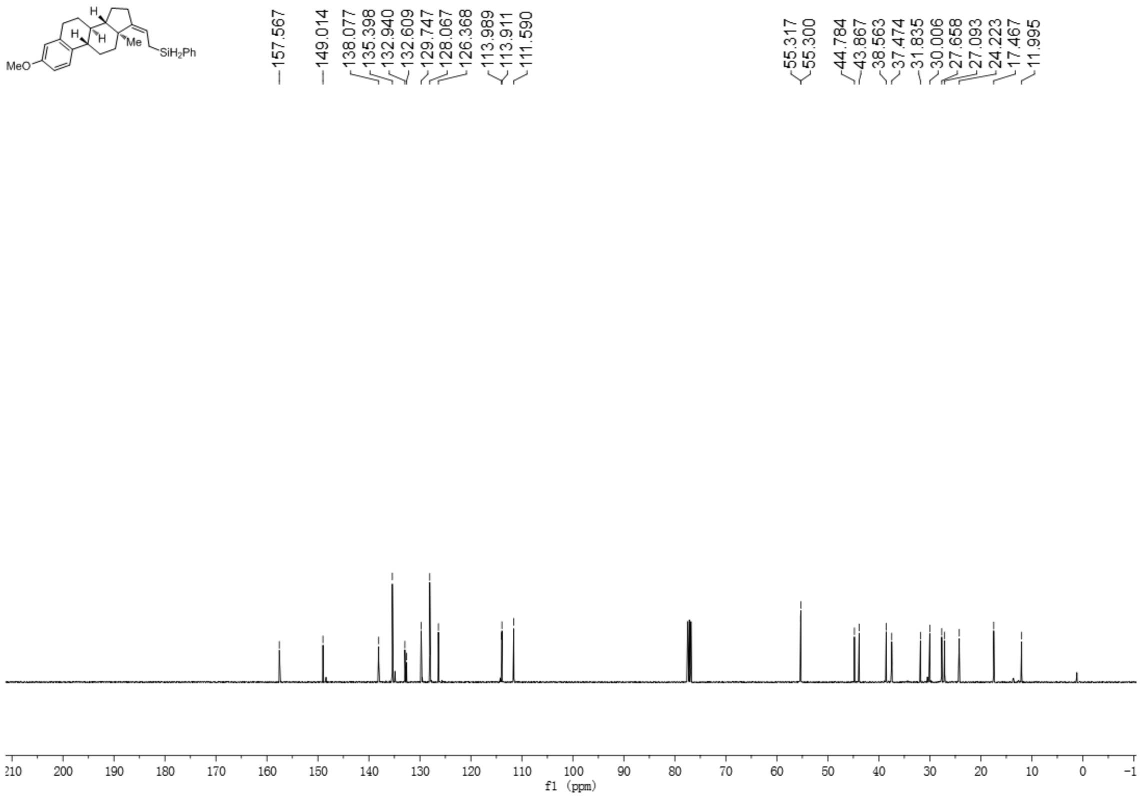

Supplementary Figure 78. ^{13}C NMR spectra for compound 3r

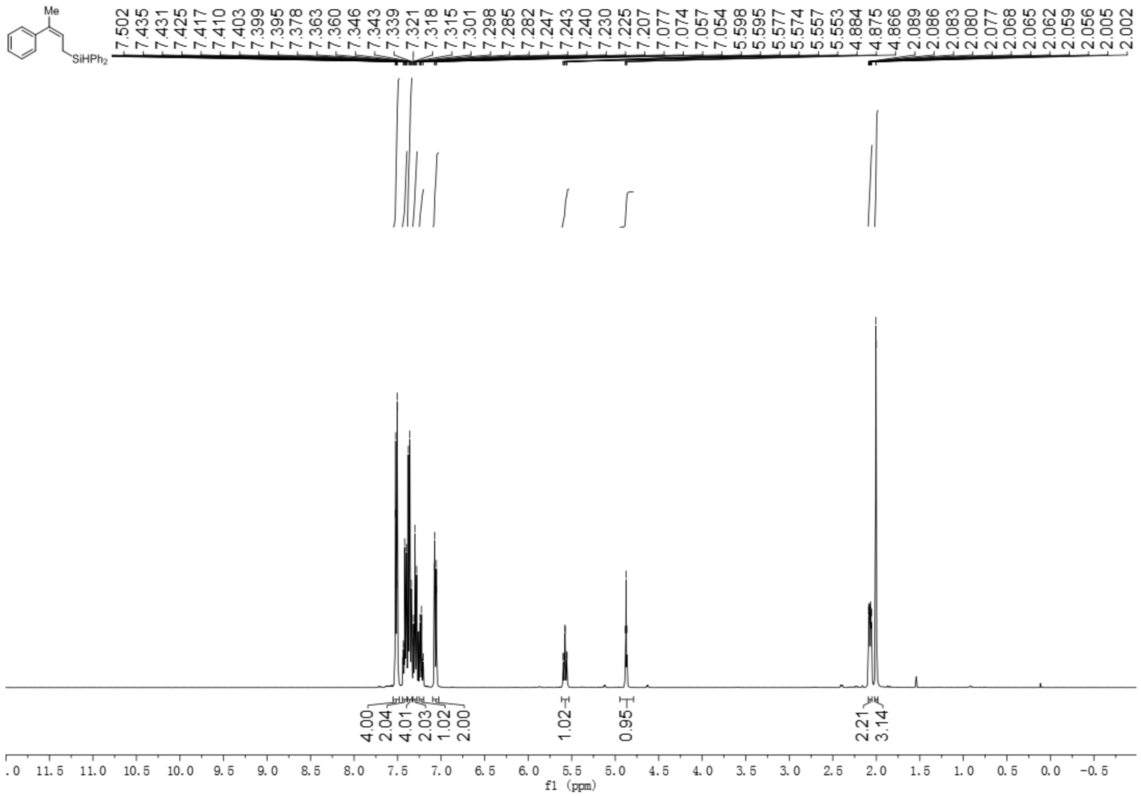

Supplementary Figure 79. ^{1}H NMR spectra for compound 3s

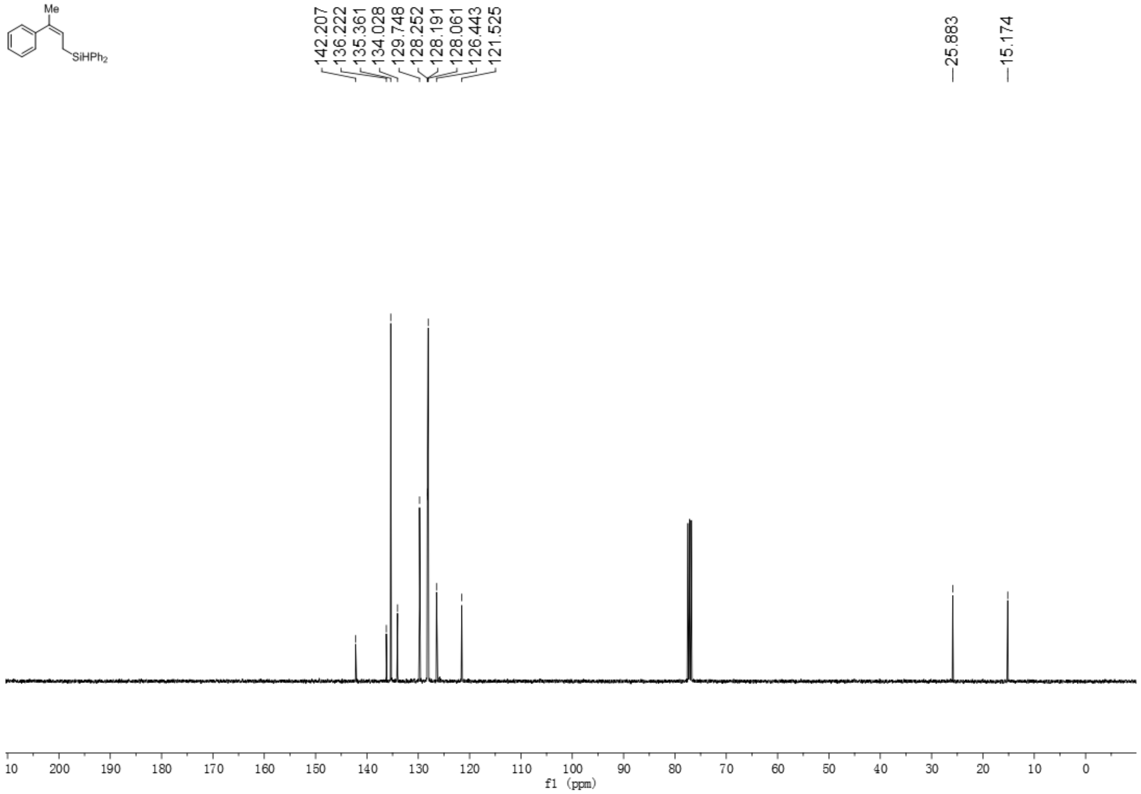

Supplementary Figure 80. ^{13}C NMR spectra for compound 3s

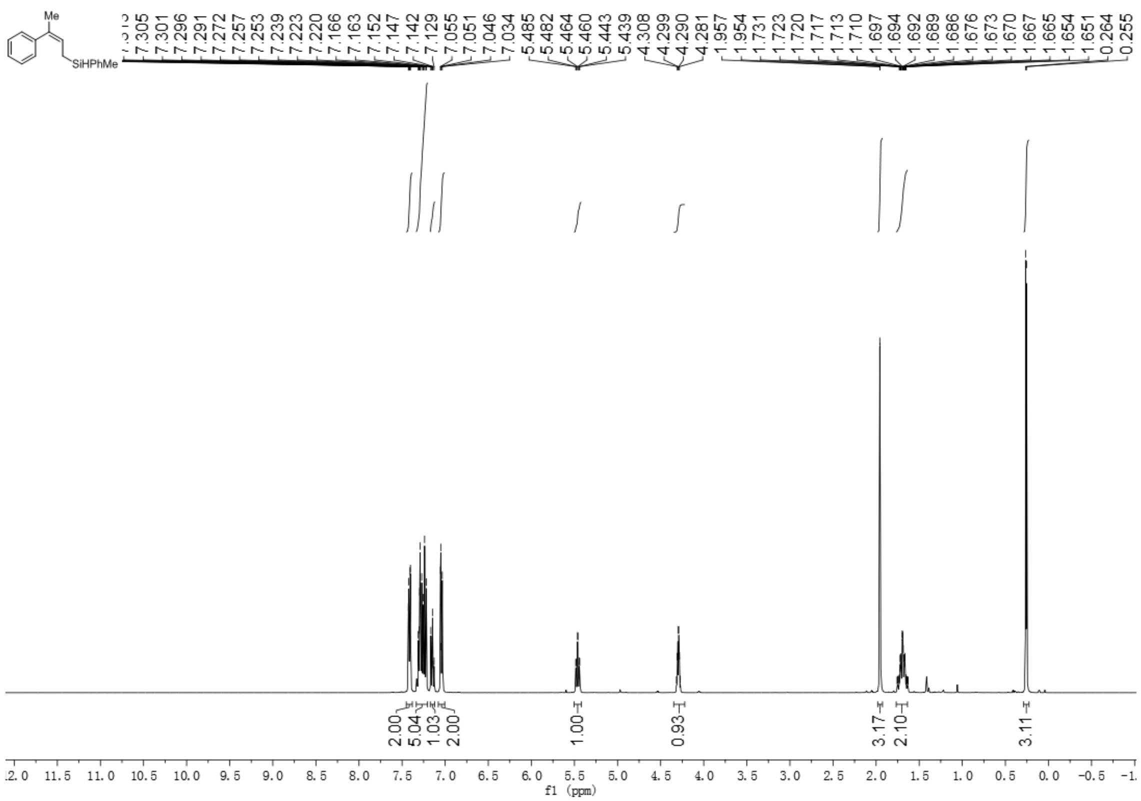

Supplementary Figure 81. ^{1}H NMR spectra for compound 3t

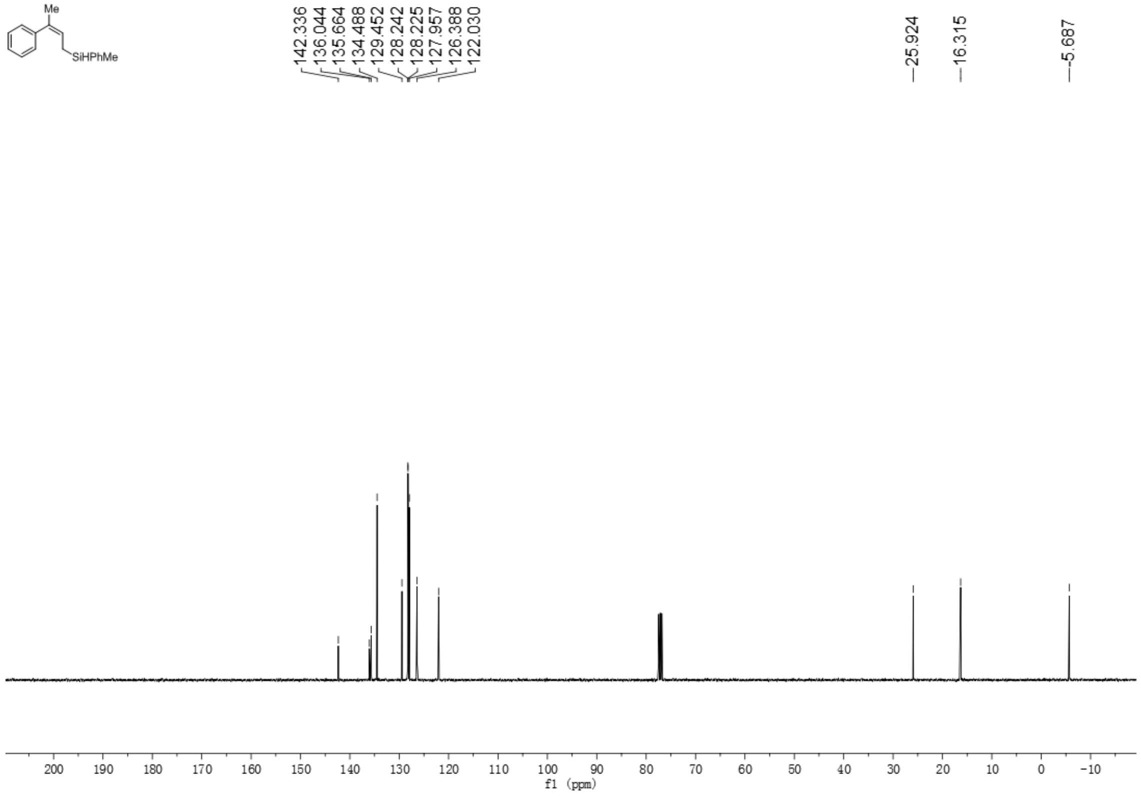

Supplementary Figure 82. ^{13}C NMR spectra for compound 3t

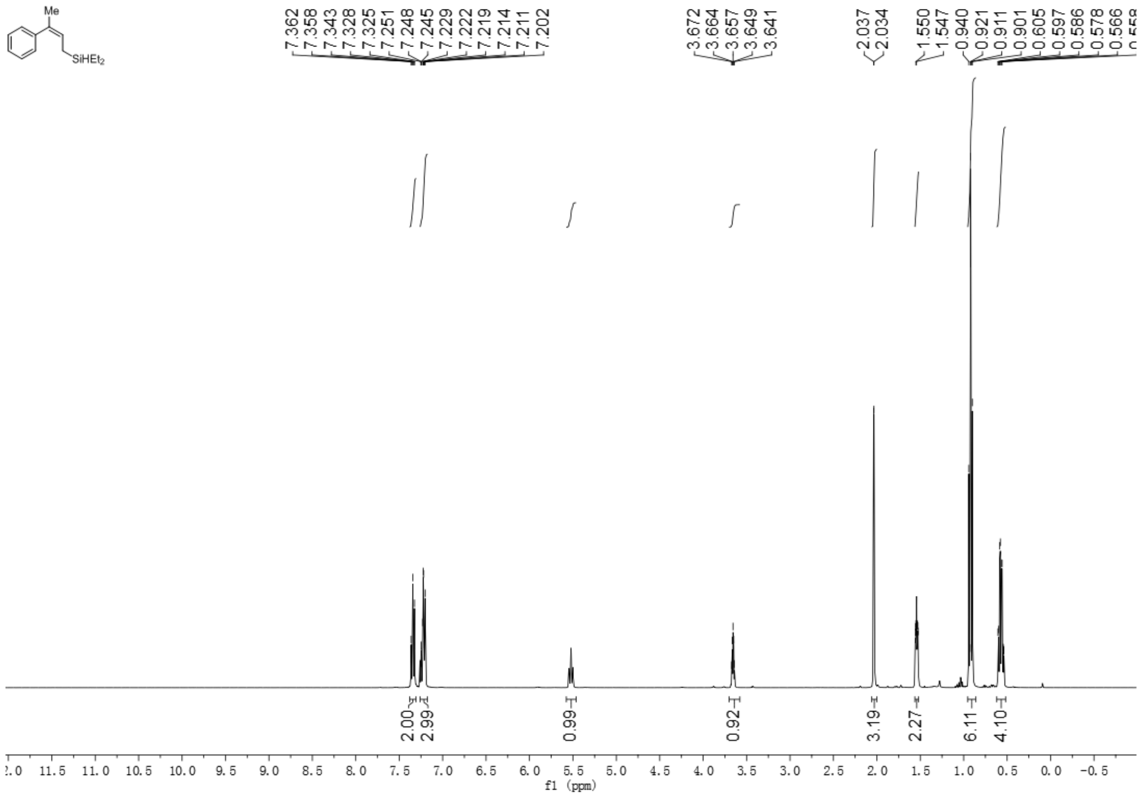

Supplementary Figure 83. ^{1}H NMR spectra for compound 3u

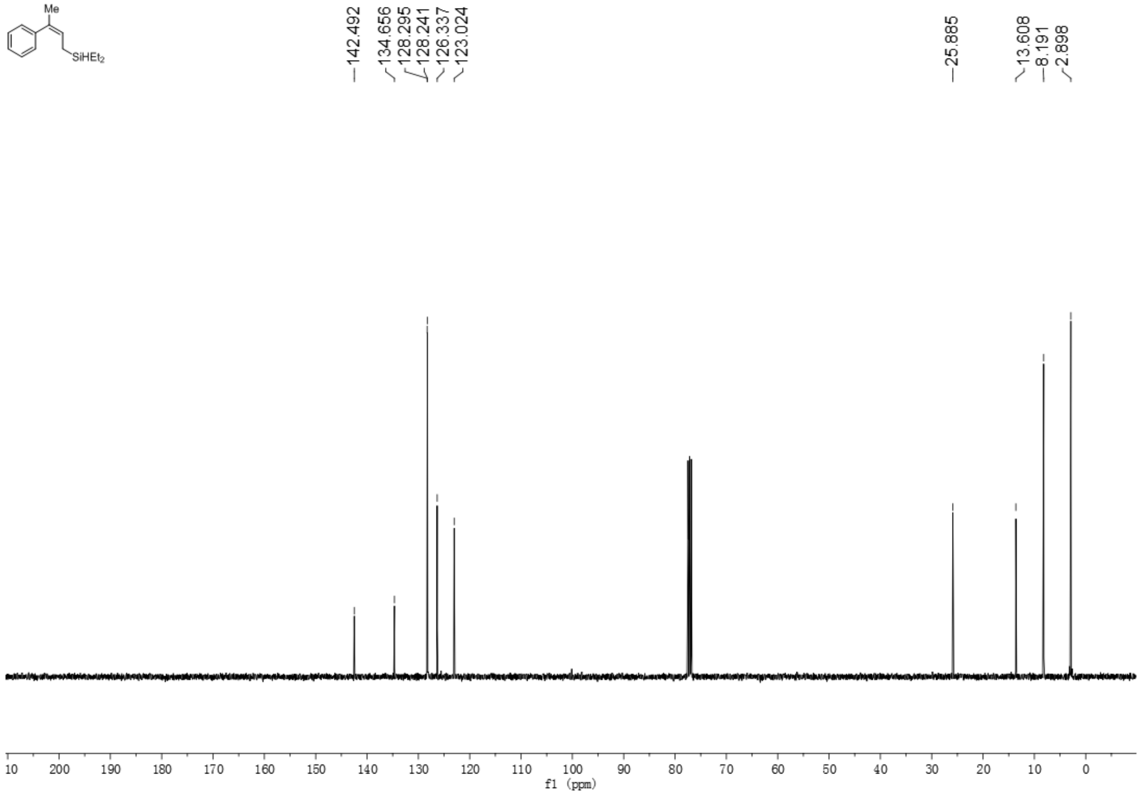

Supplementary Figure 84. ^{13}C NMR spectra for compound 3u

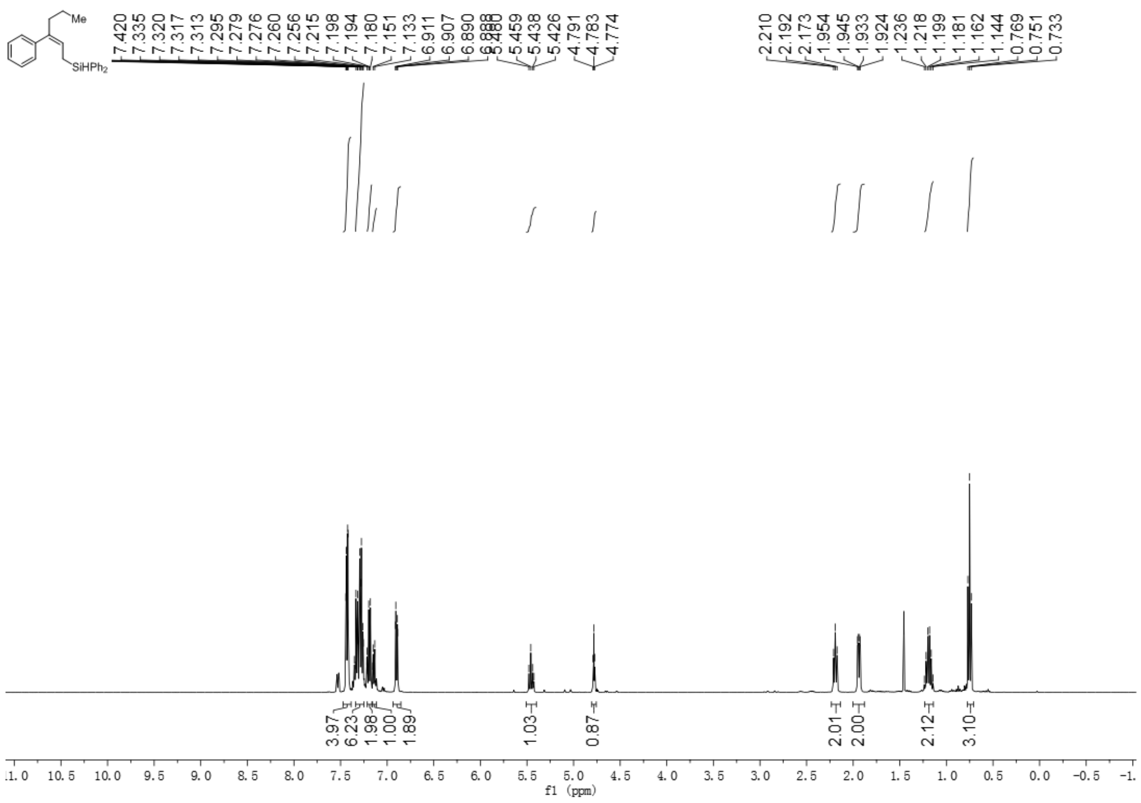

Supplementary Figure 85. ^{1}H NMR spectra for compound 3v

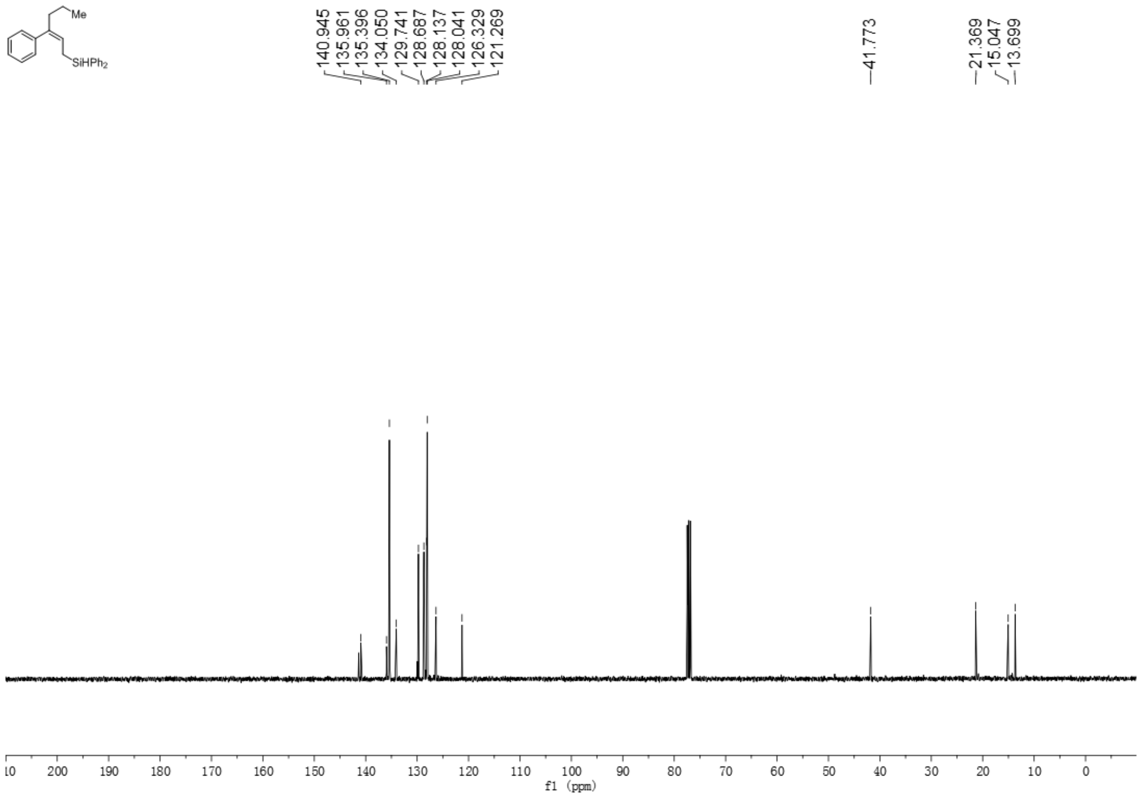

Supplementary Figure 86. ^{13}C NMR spectra for compound 3v

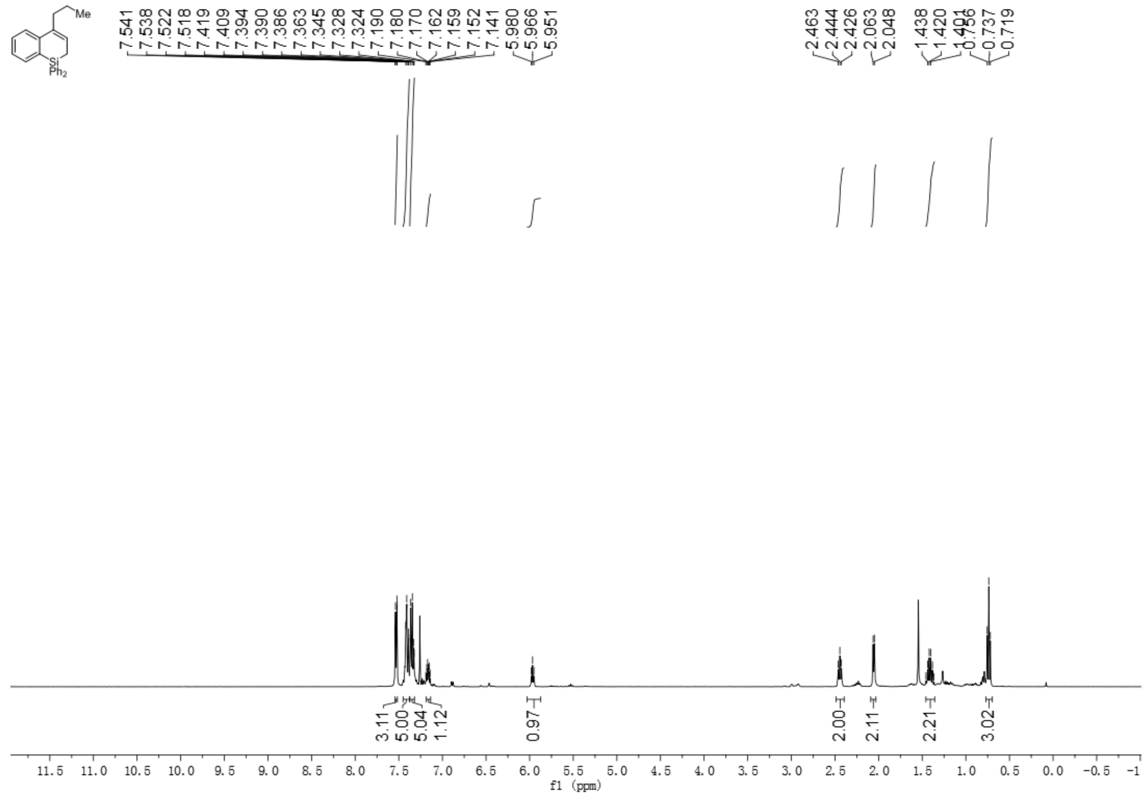

Supplementary Figure 87. ^{1}H NMR spectra for compound 4

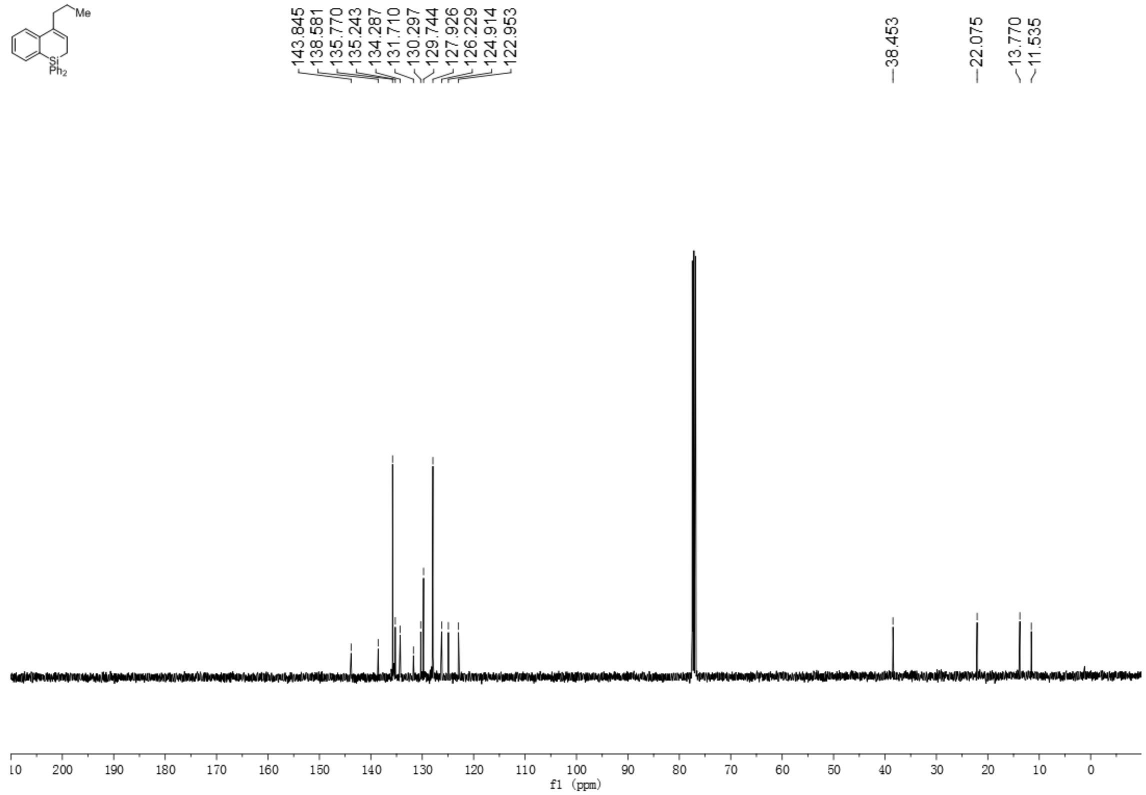

Supplementary Figure 88. ^{13}C NMR spectra for compound 4

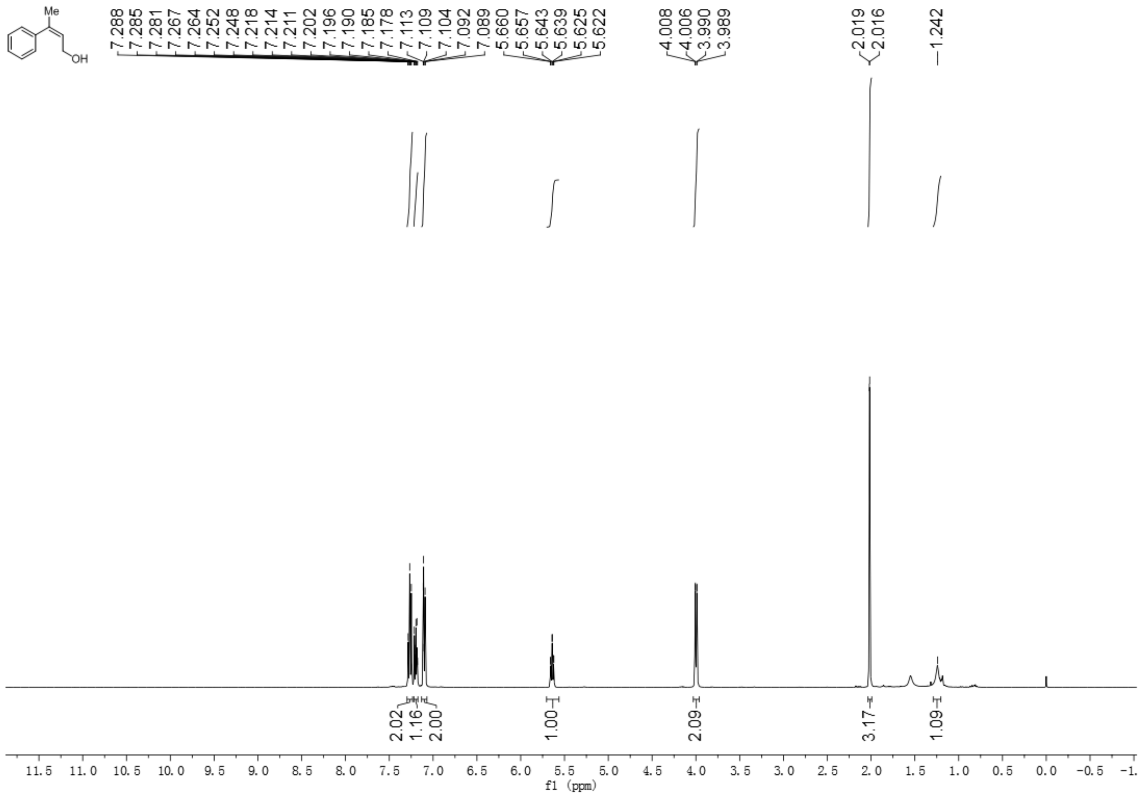

Supplementary Figure 89. ^{1}H NMR spectra for compound 5a

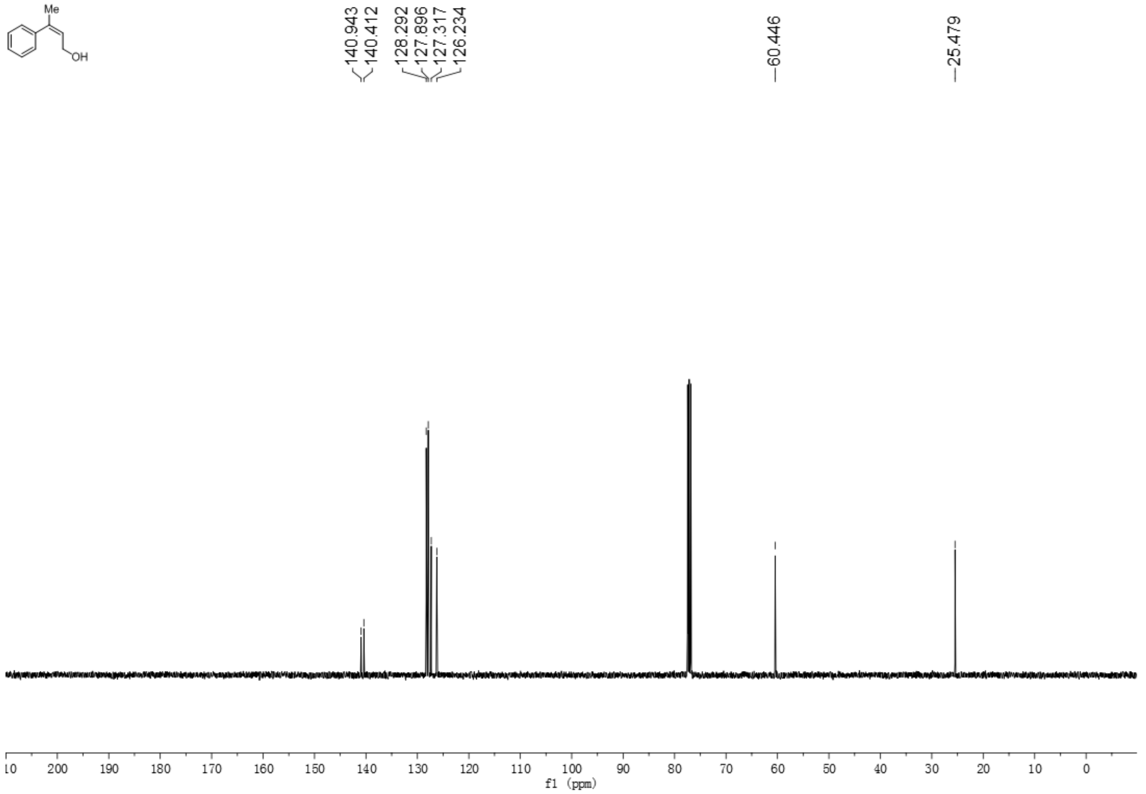

Supplementary Figure 90. ^{13}C NMR spectra for compound 5a

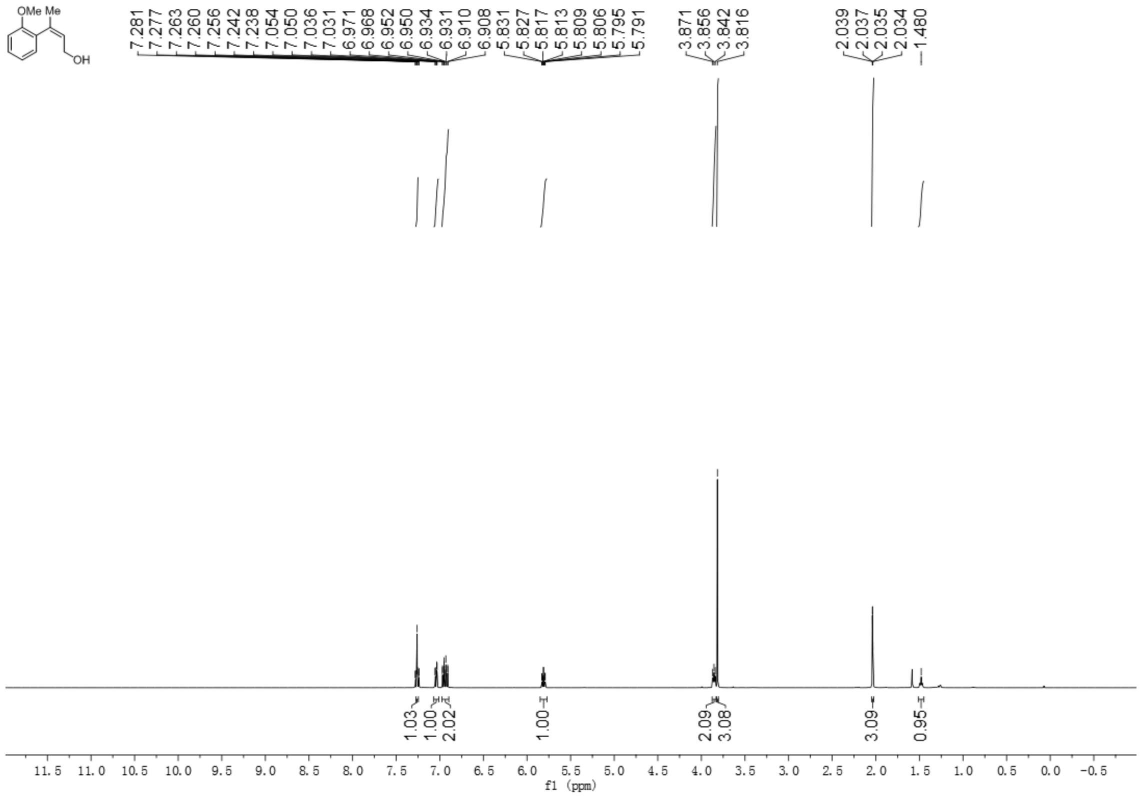

Supplementary Figure 91. ^{1}H NMR spectra for compound 5b

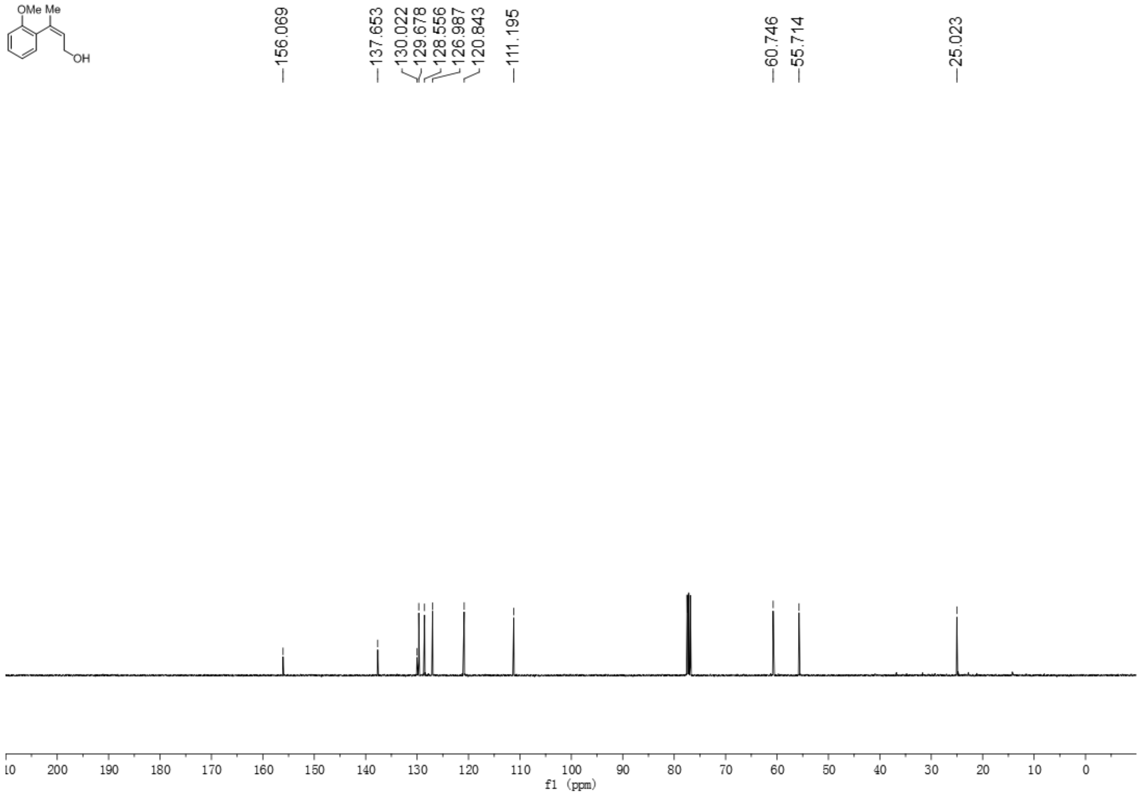

Supplementary Figure 92. ^{13}C NMR spectra for compound 5b

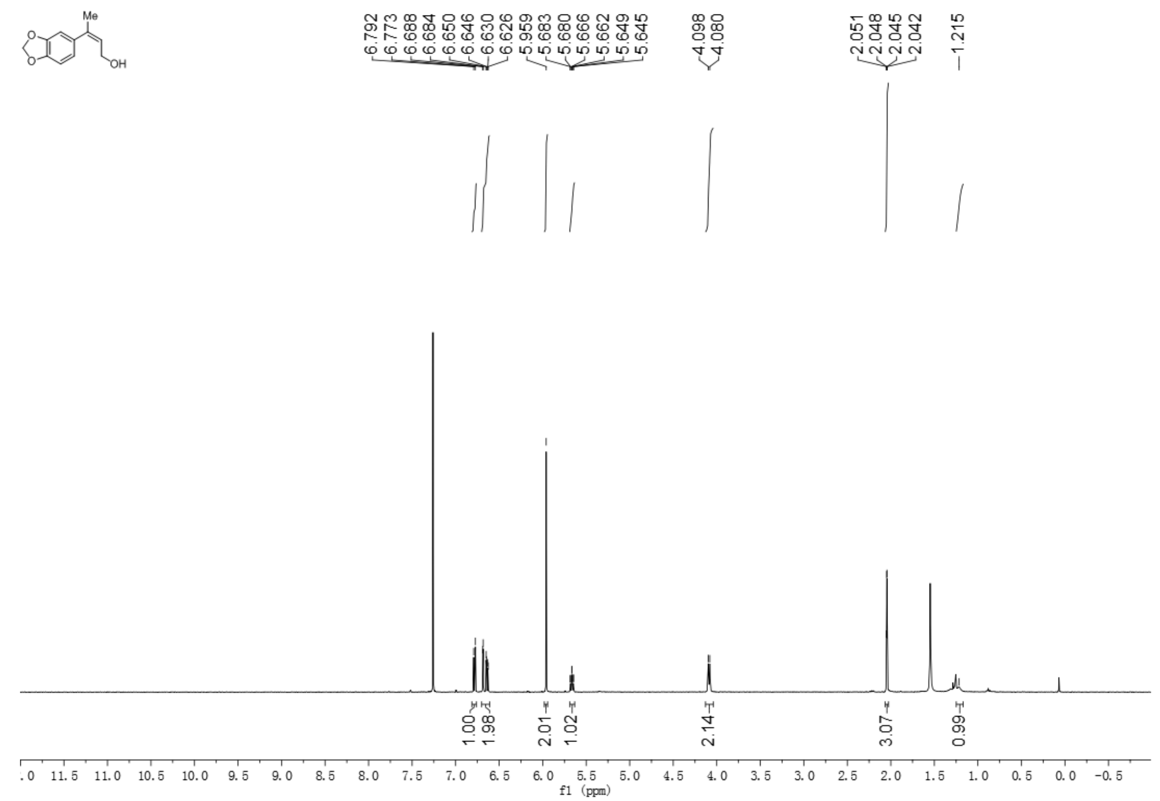

Supplementary Figure 93. ^{1}H NMR spectra for compound 5c

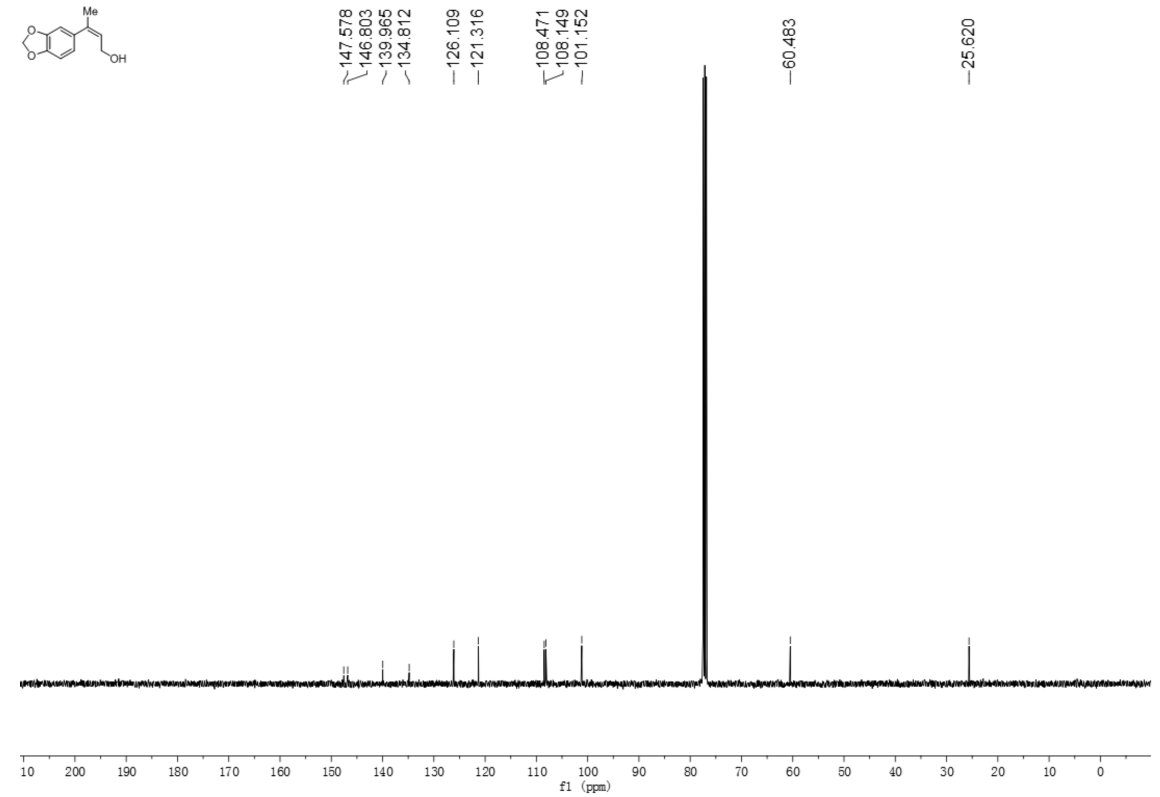

Supplementary Figure 94. ^{13}C NMR spectra for compound 5c

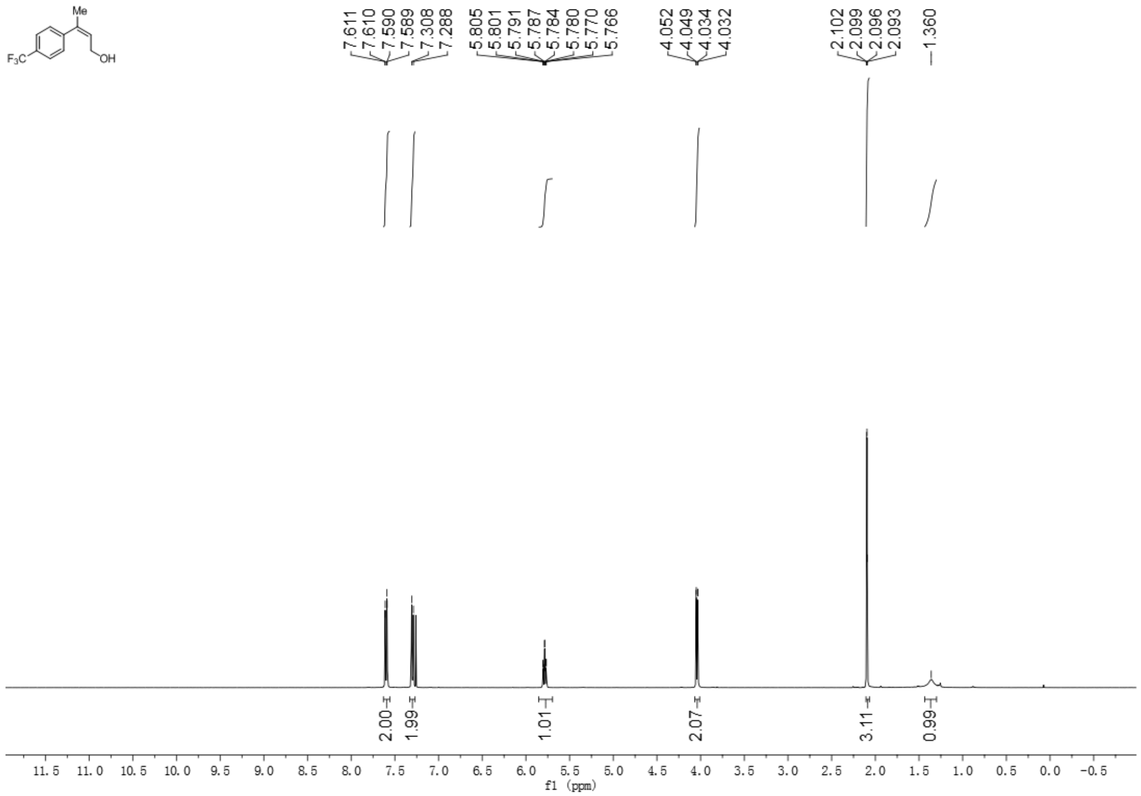

Supplementary Figure 95. ^{1}H NMR spectra for compound 5d

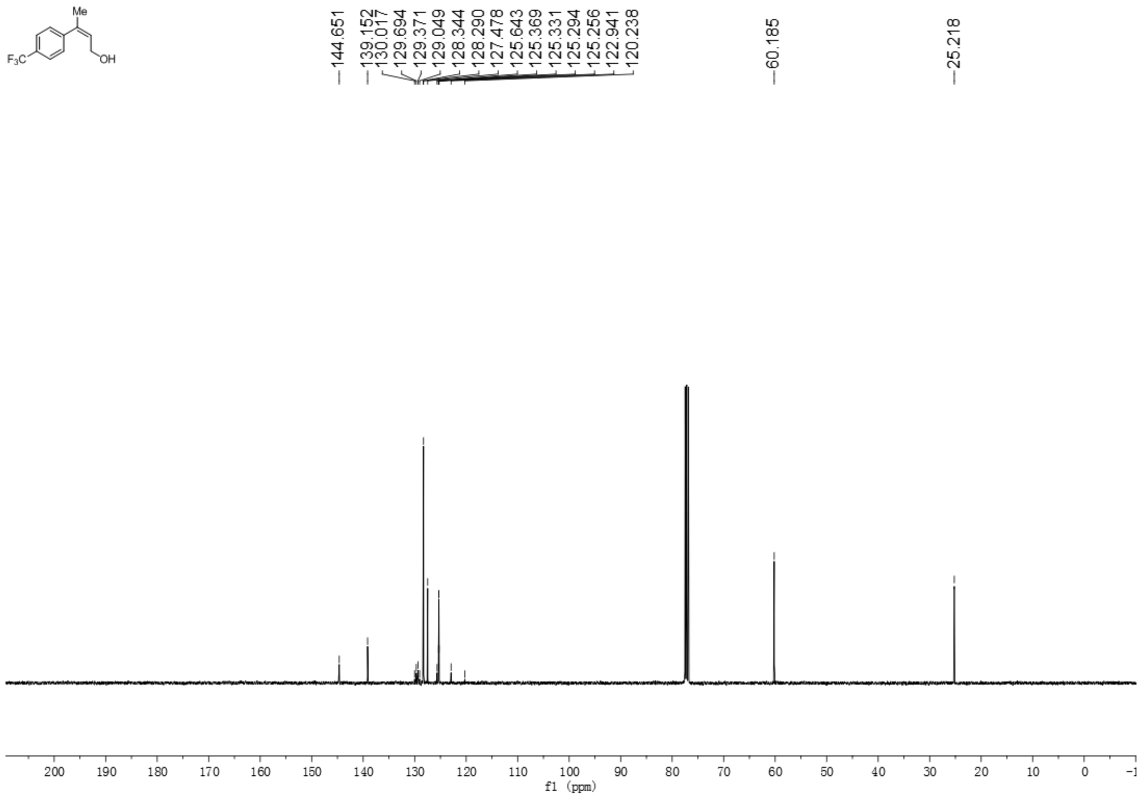

Supplementary Figure 96. ^{13}C NMR spectra for compound 5d

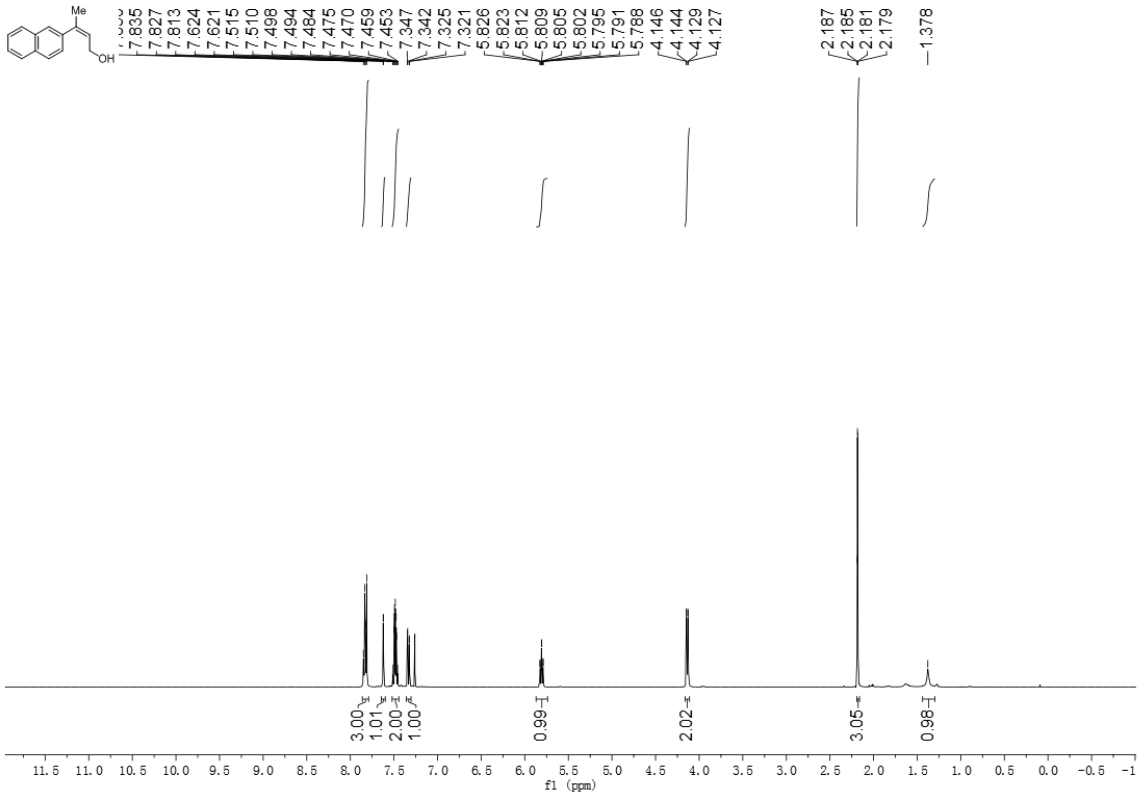

Supplementary Figure 97. ^{1}H NMR spectra for compound 5e

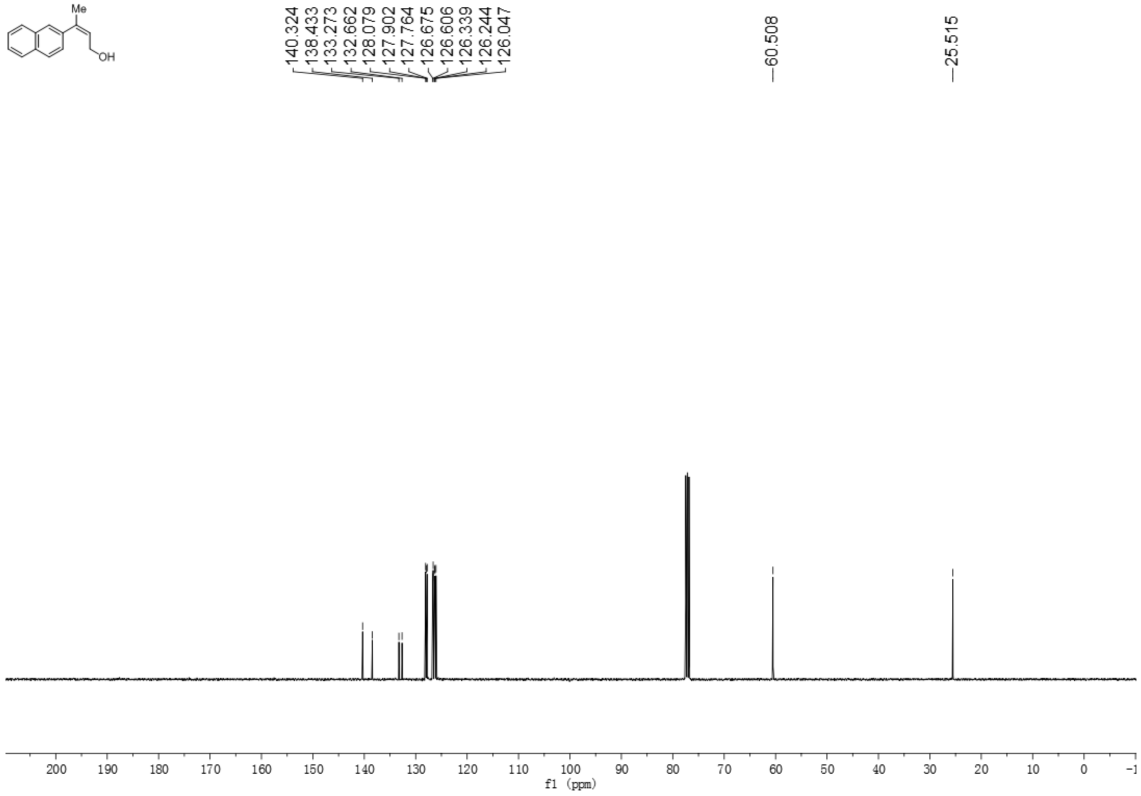

Supplementary Figure 98. ^{13}C NMR spectra for compound 5e

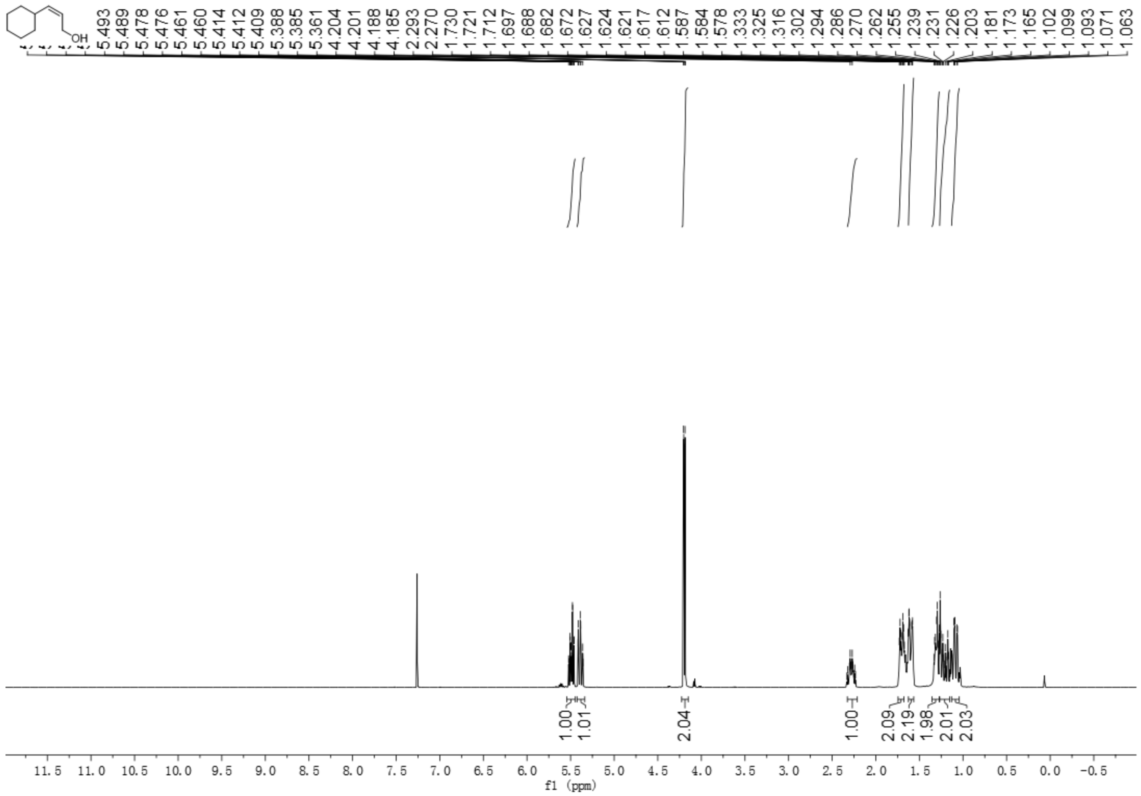

Supplementary Figure 99. ^{1}H NMR spectra for compound 5f

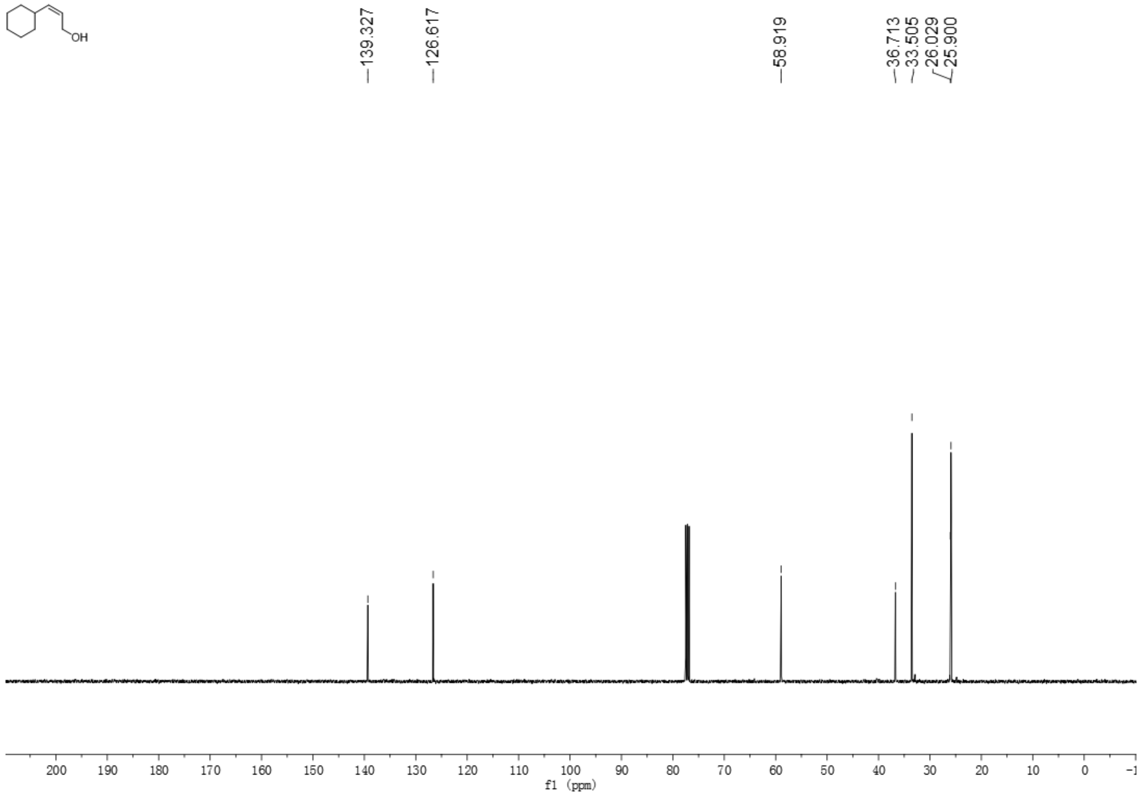

Supplementary Figure 100. ^{13}C NMR spectra for compound 5f

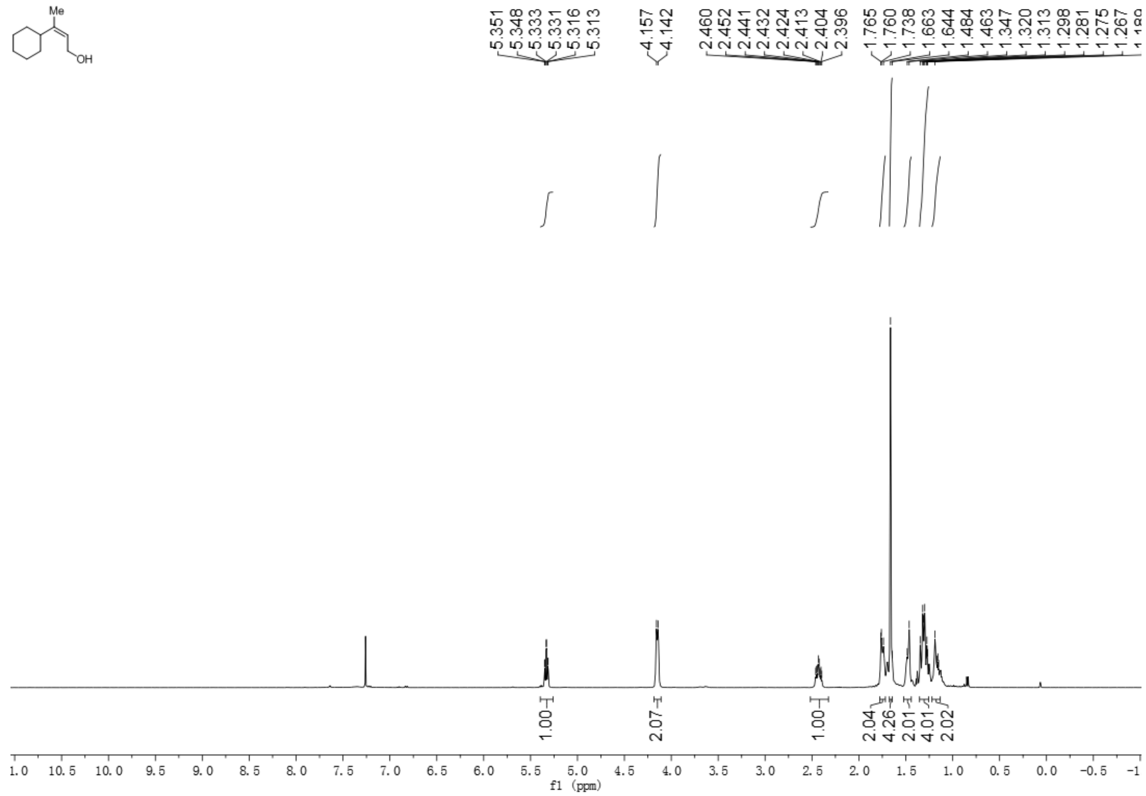

Supplementary Figure 101. ^{1}H NMR spectra for compound 5g

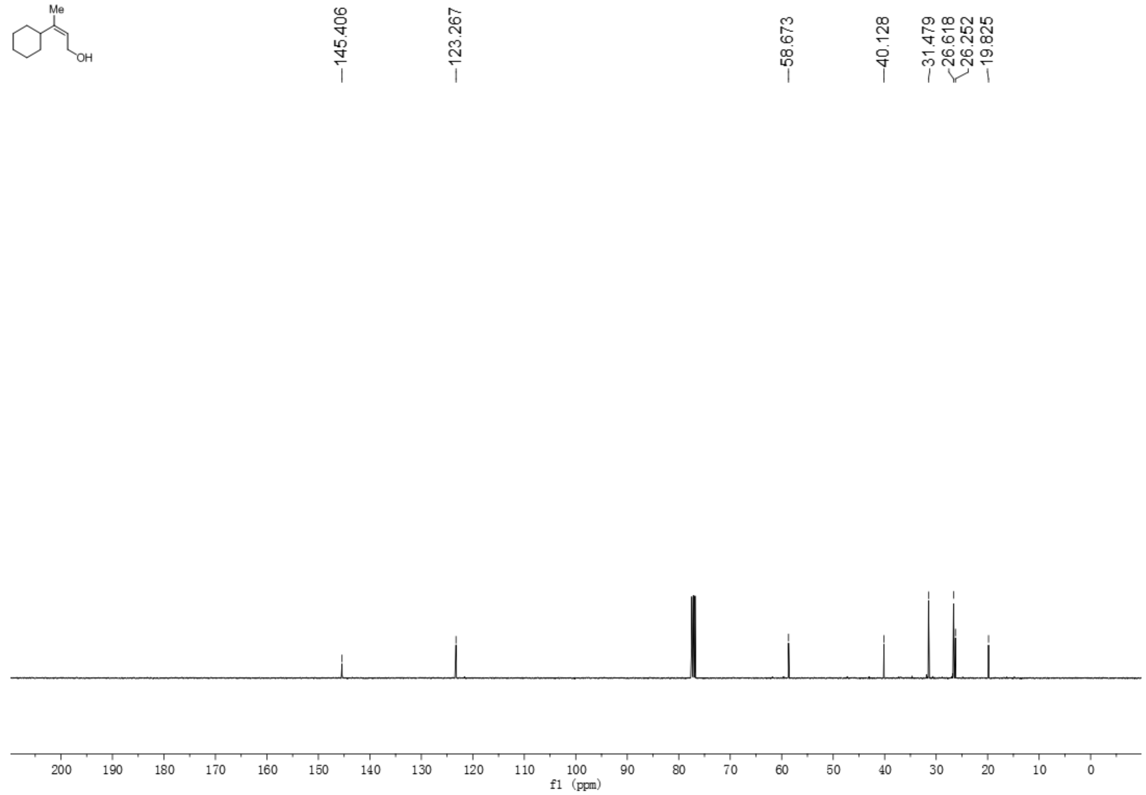

Supplementary Figure 102. ^{13}C NMR spectra for compound 5g

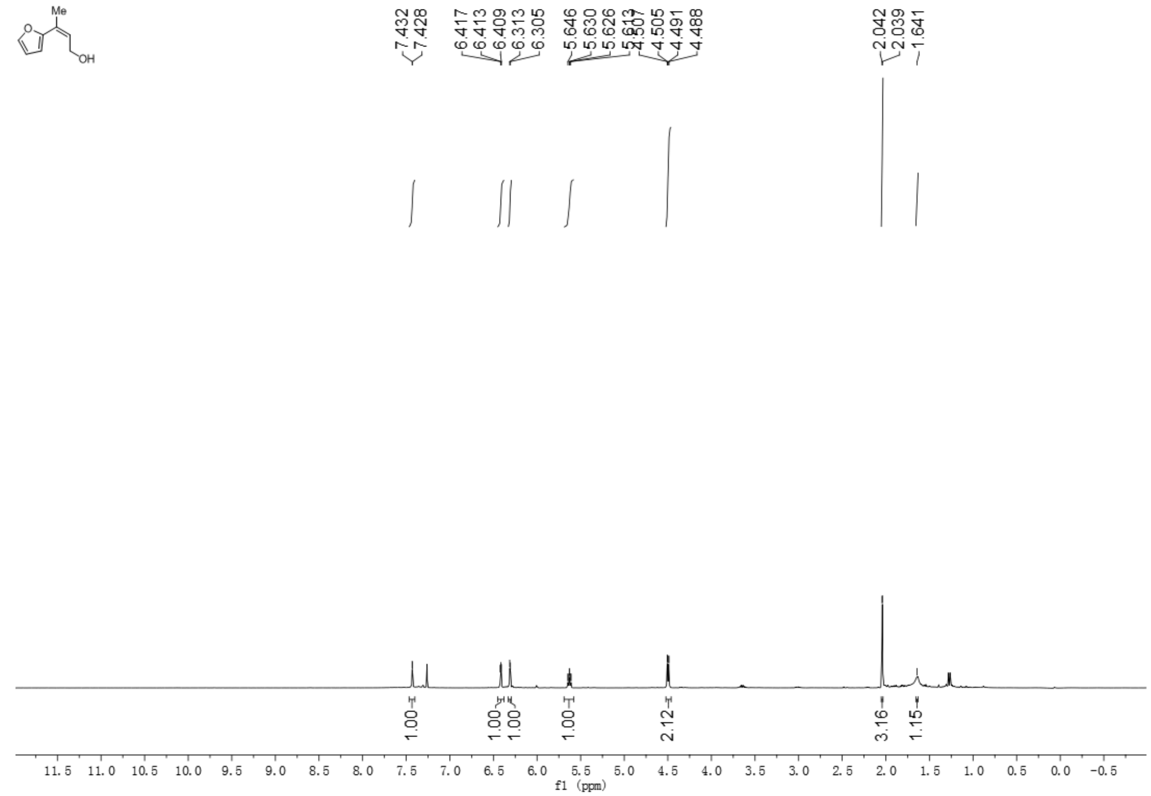

Supplementary Figure 103. ^{1}H NMR spectra for compound 5h

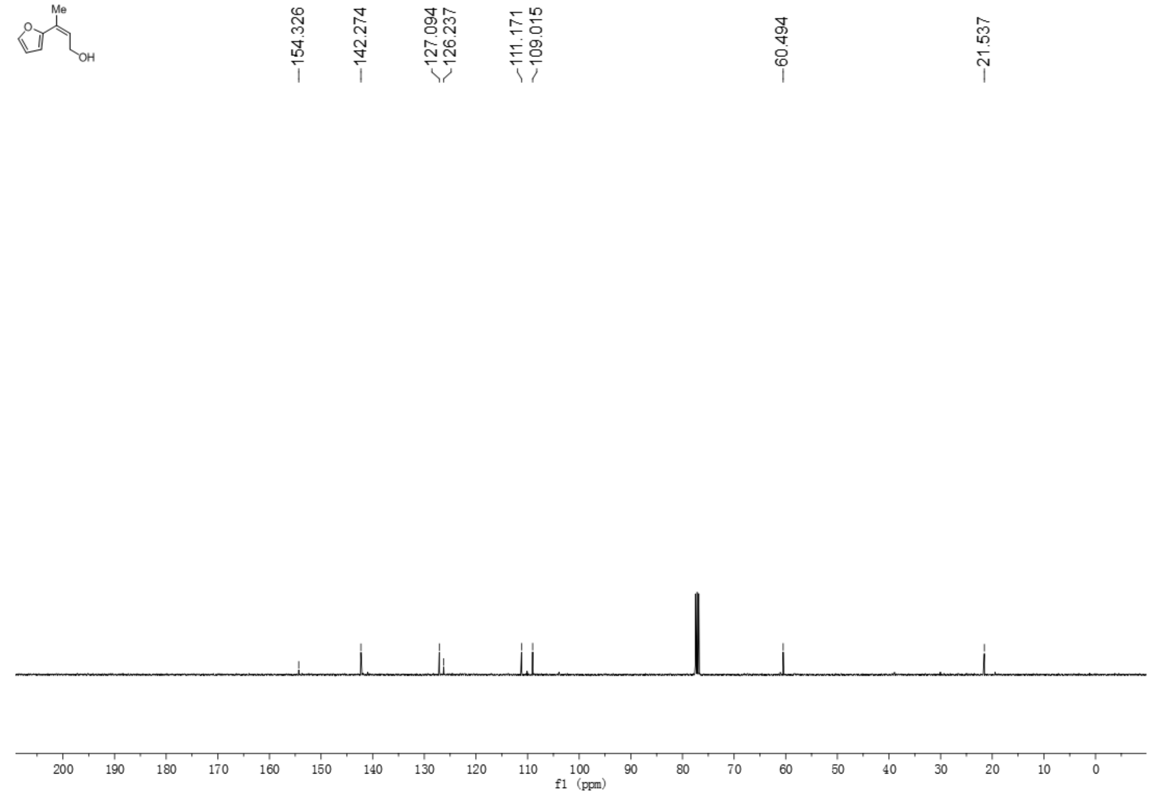

Supplementary Figure 104. ^{13}C NMR spectra for compound 5h

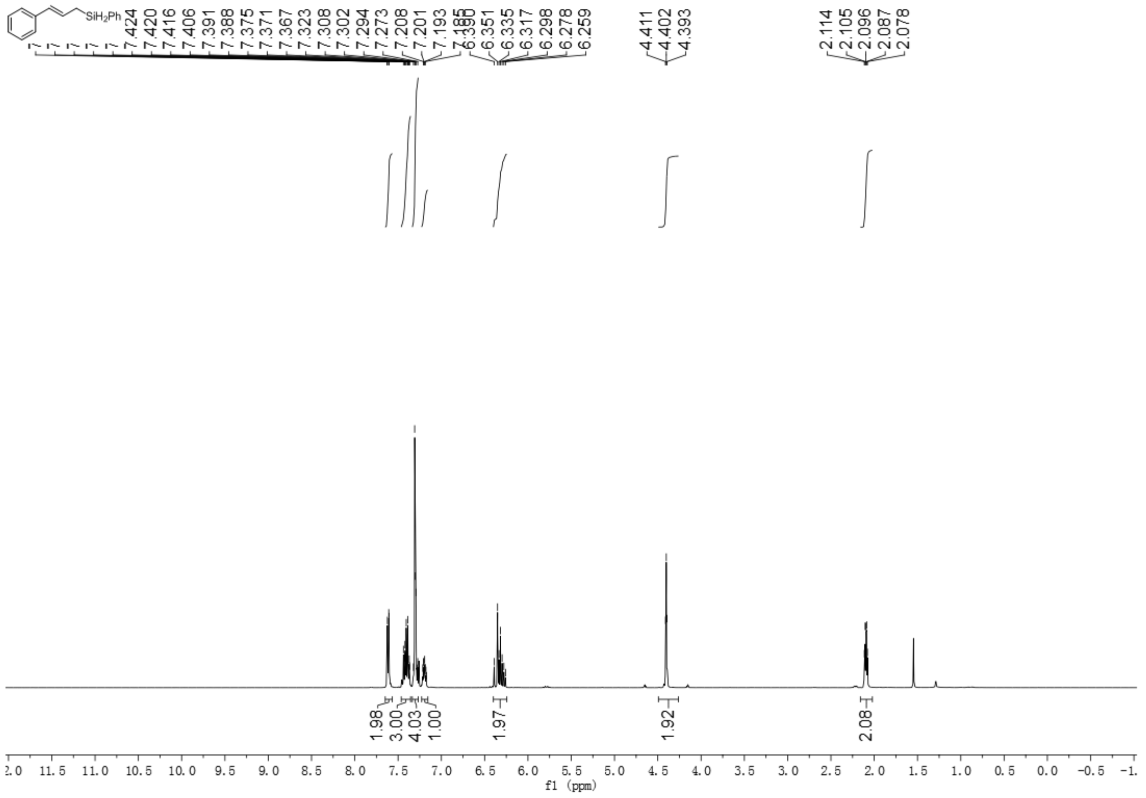

Supplementary Figure 105. ^{1}H NMR spectra for compound (*E*)-1l

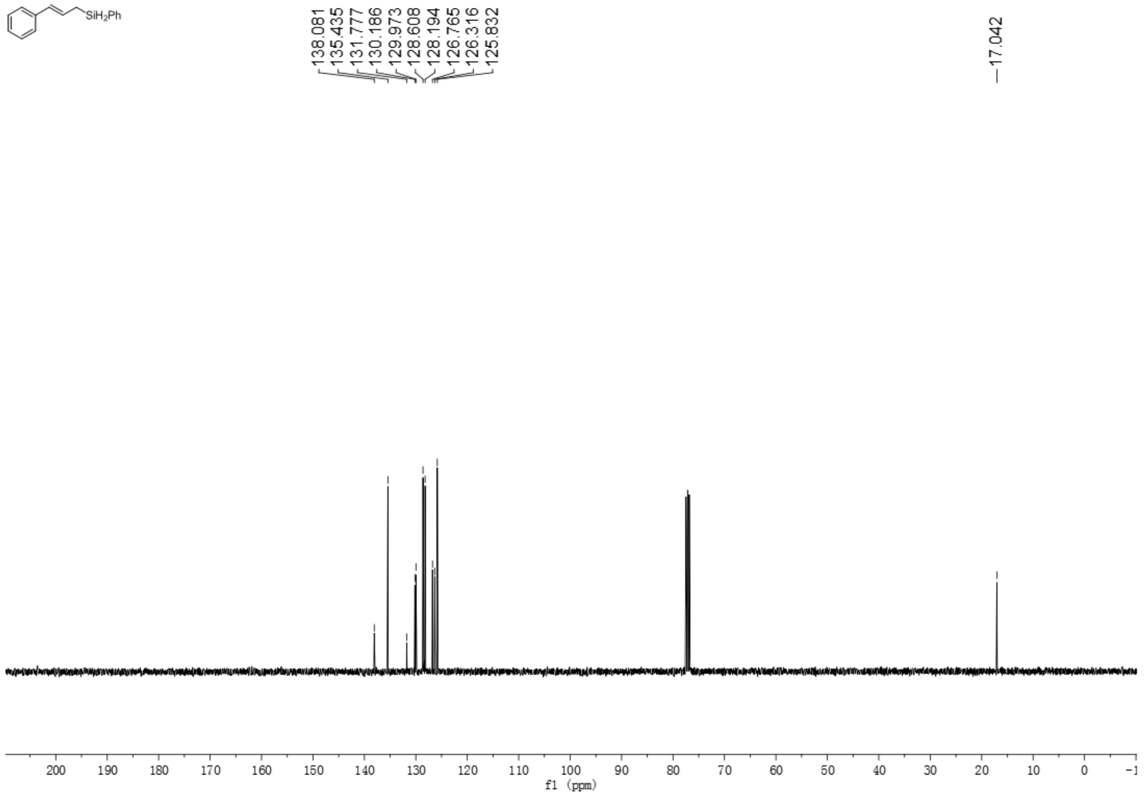

Supplementary Figure 106. ^{13}C NMR spectra for compound (*E*)-1l

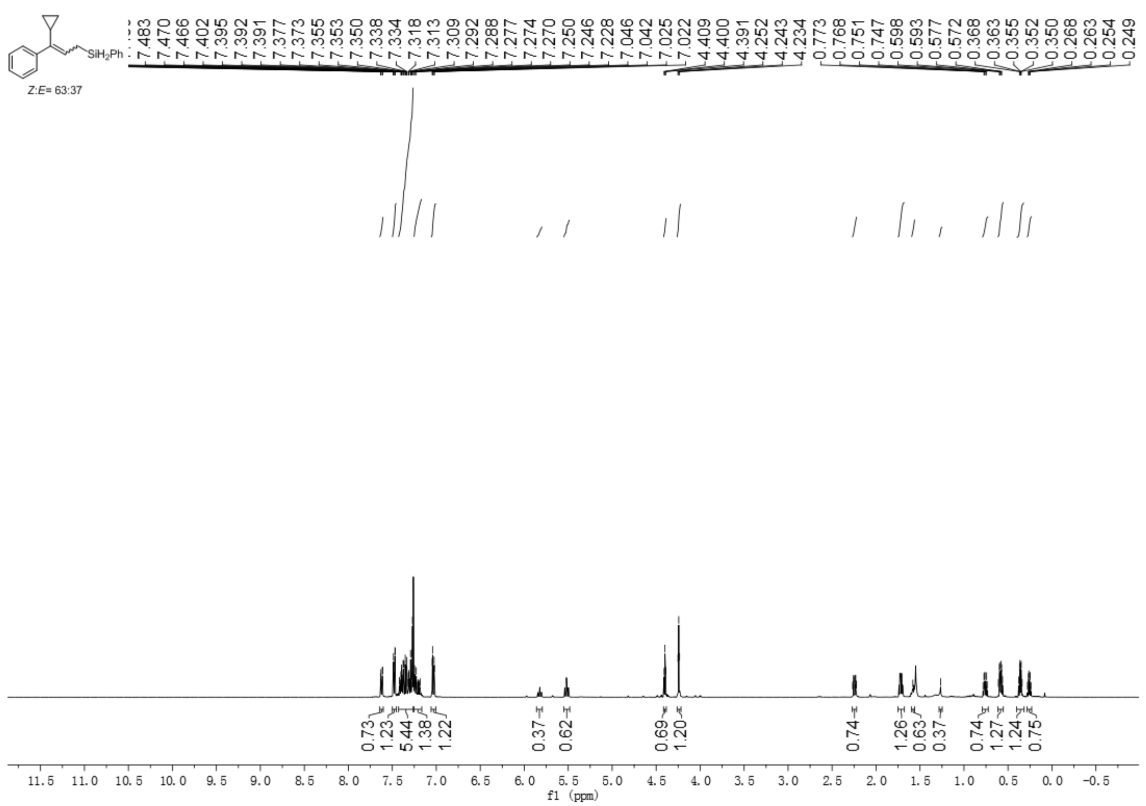

Supplementary Figure 107. ^{1}H NMR spectra for compound 3w

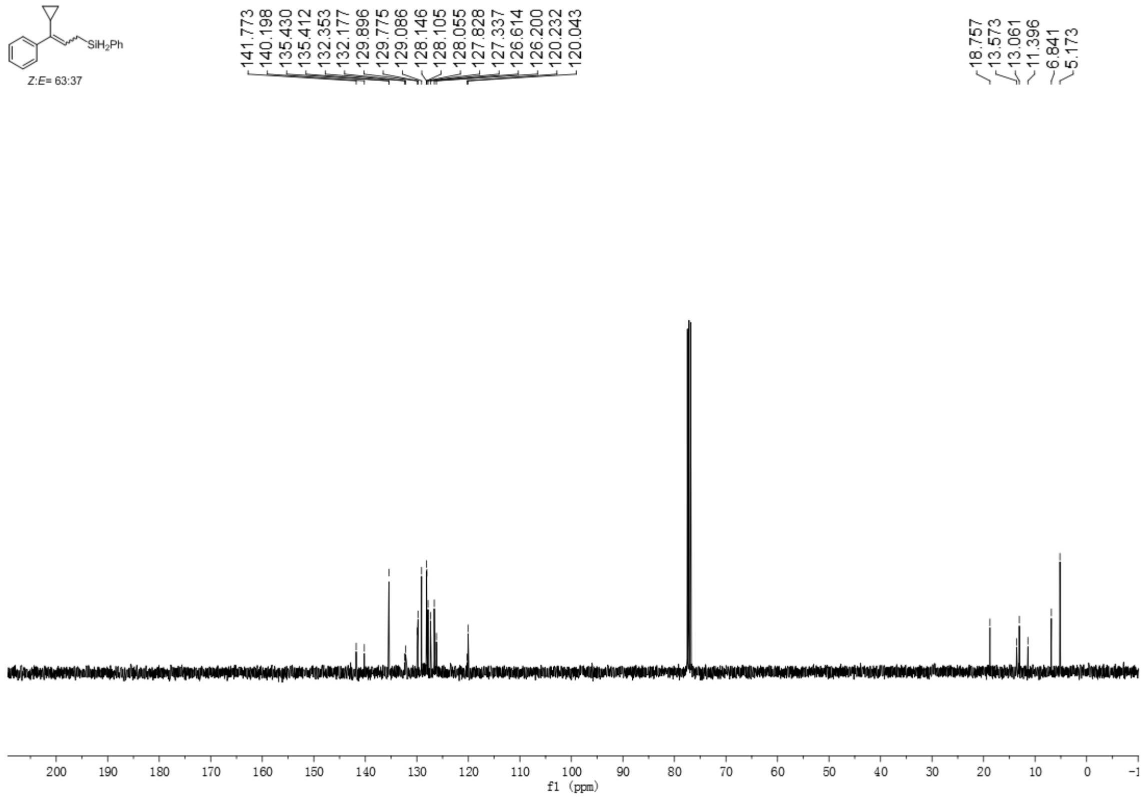

Supplementary Figure 108. ^{13}C NMR spectra for compound 3w

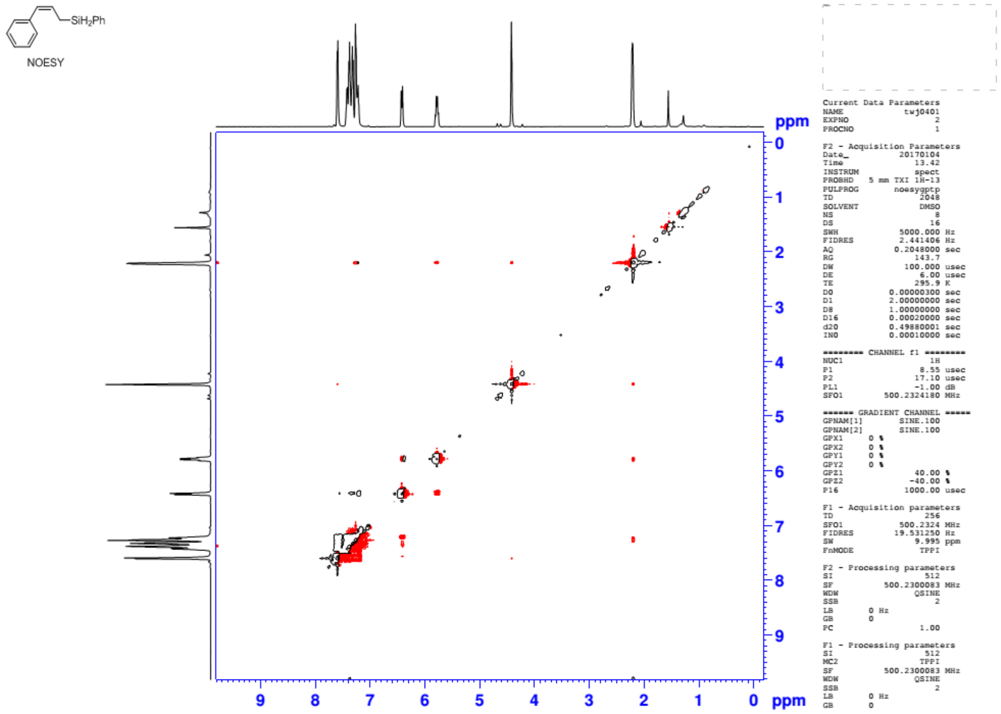

Supplementary Figure 109. 2D NOESY spectra for compound 1n

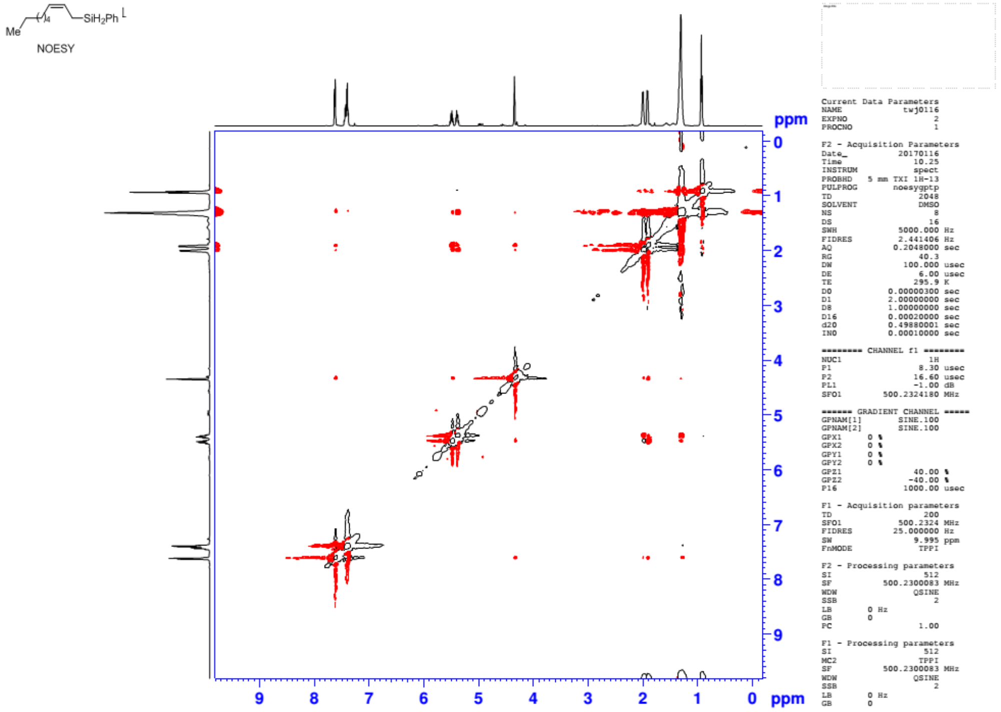

Supplementary Figure 110. 2D NOESY spectra for compound 1e

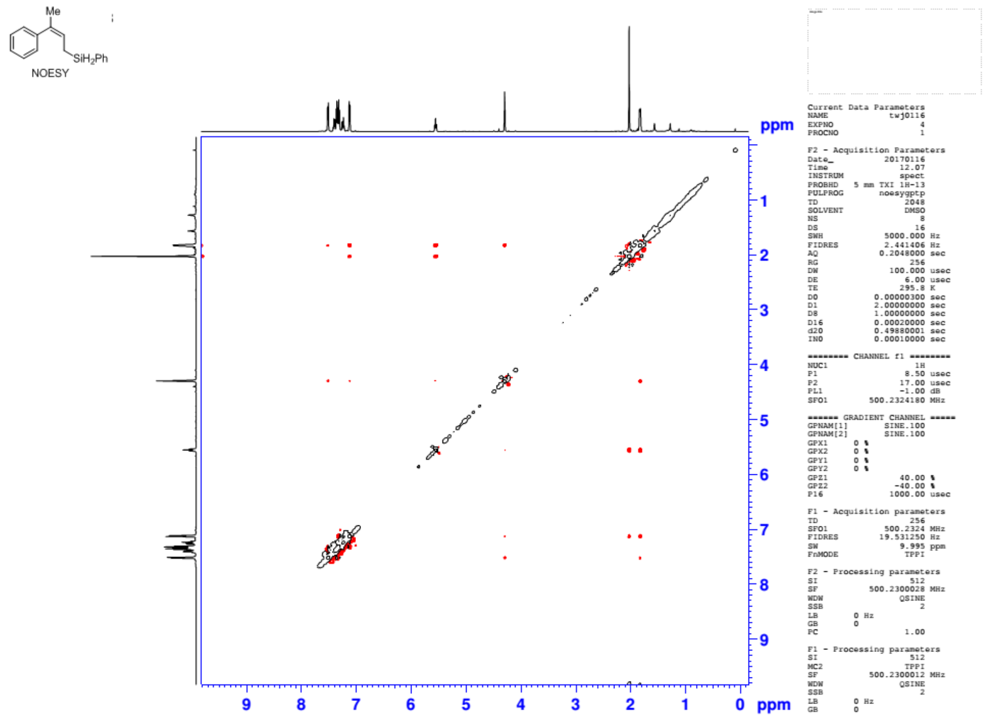

Supplementary Figure 111. 2D NOESY spectra for compound 3a

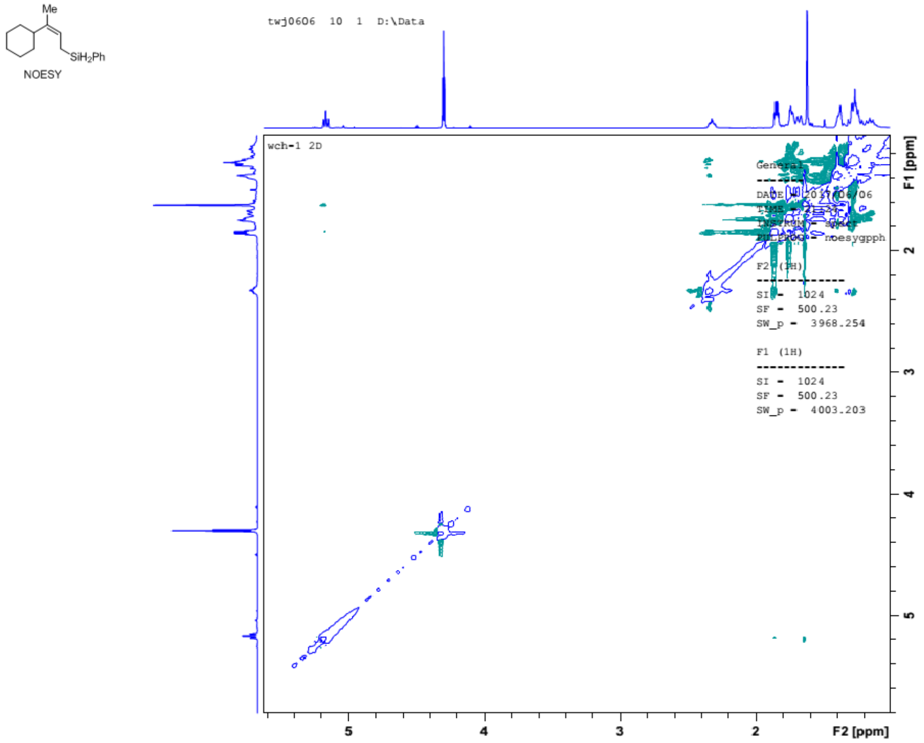

Supplementary Figure 112. 2D NOESY spectra for compound 3p

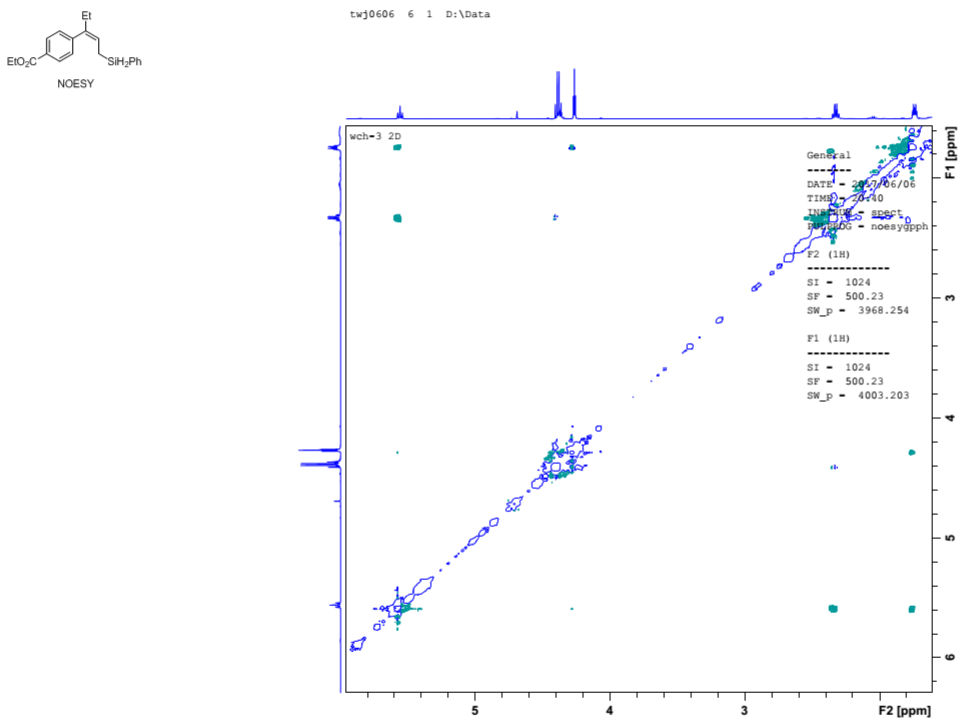

Supplementary Figure 113. 2D NOESY spectra for compound 3i

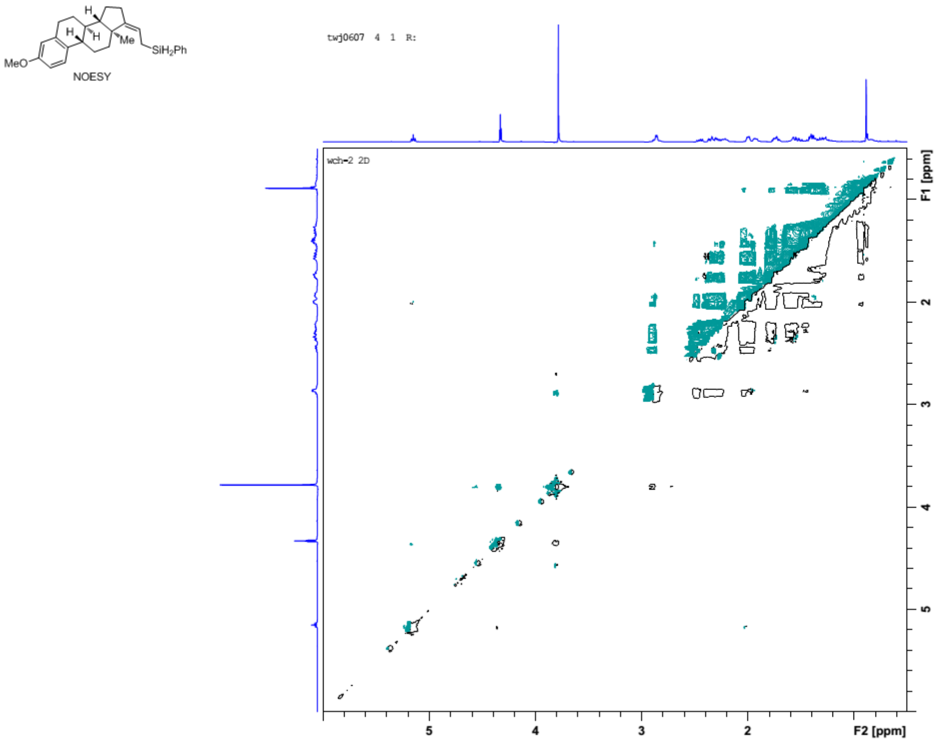

Supplementary Figure 114. 2D NOESY spectra for compound 3r

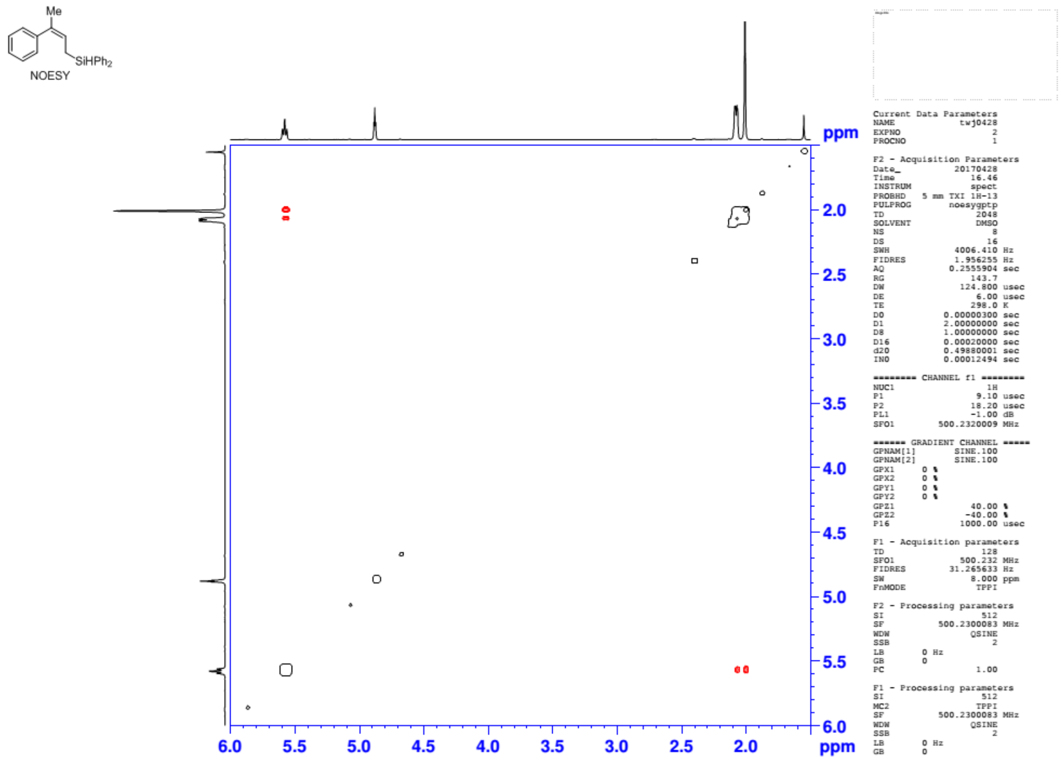

Supplementary Figure 115. 2D NOESY spectra for compound 3s

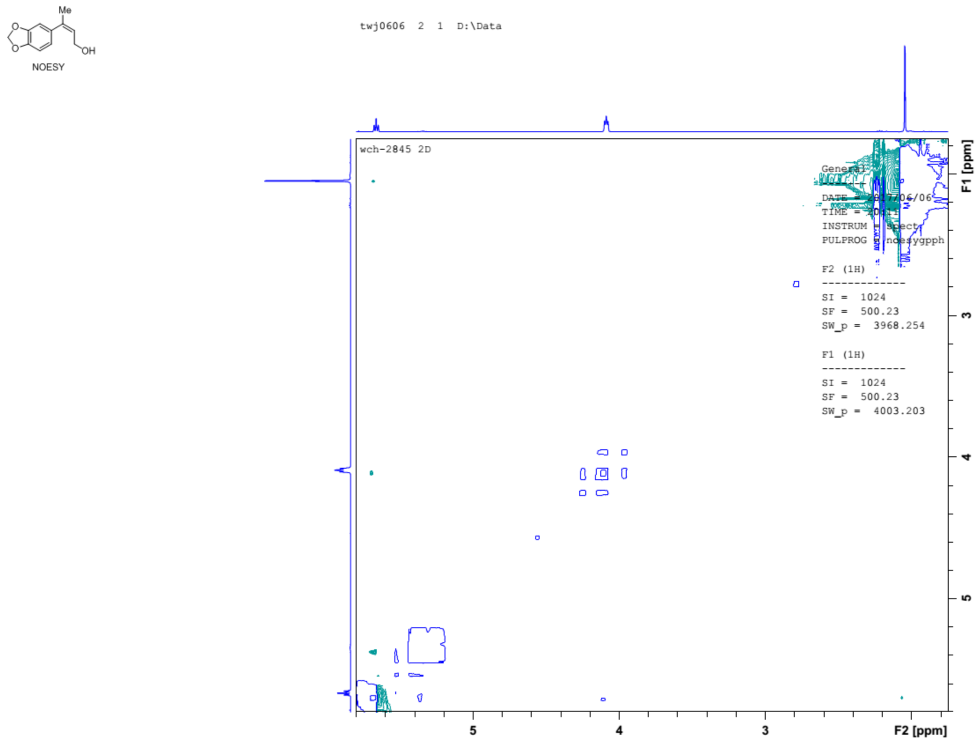

Supplementary Figure 116. 2D NOESY spectra for compound 5c

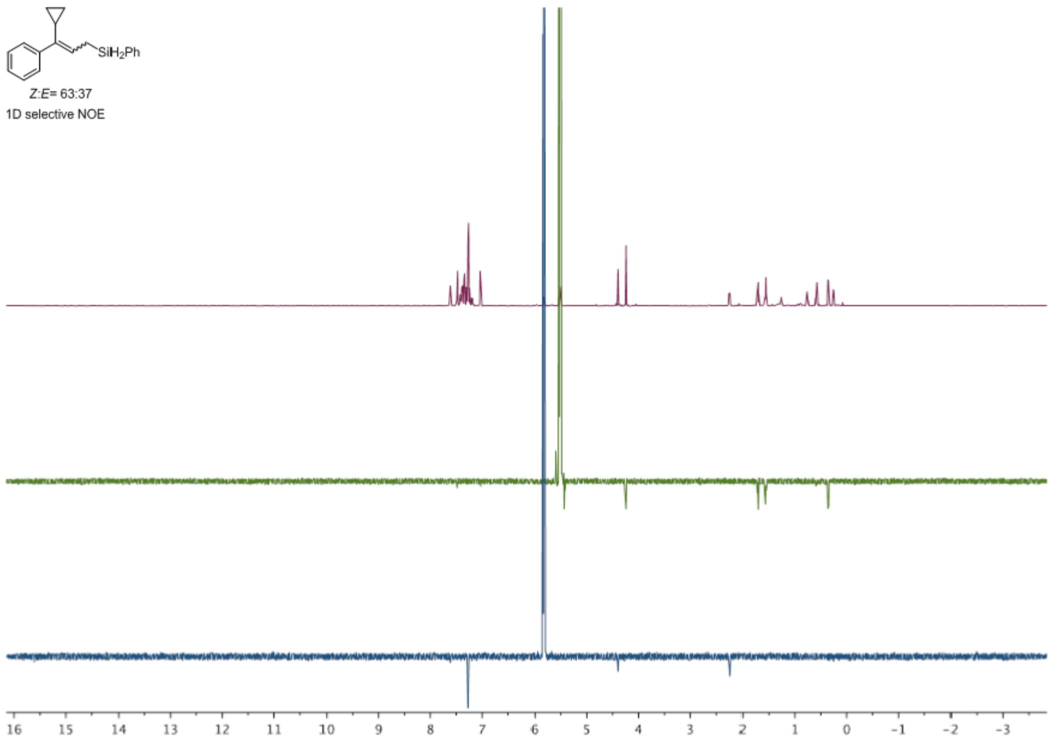

Supplementary Figure 117. 2D NOESY spectra for compound 5c

Supplementary Tables

Supplementary Table 1. Hydrosilylation of buta-2,3-dien-2-ylbenzene catalyzed by $(dppbz)_2CoH$[a]

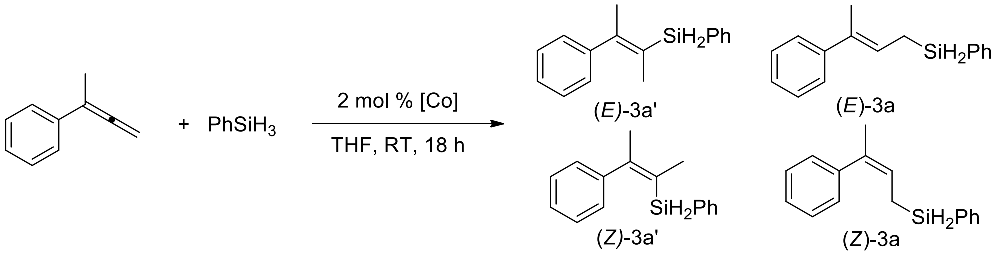

entry	Cobalt catalyst	yield[b]	product distribution[b]			
			(*E*)-3a'	(*Z*)-3a'	(*E*)-3a	(*Z*)-3a
1	$Co(acac)_2$ (2 mol %) + dppbz (2 mol %)	99%	13.4%	6.8%	14.7%	65.1%
2	$Co(acac)_2$ (2 mol %) + dppbz (4 mol %)	62%	11.7%	6.8%	15.3%	66.2%
3	$Co(acac)_2$ (2 mol %) + dppbz (8 mol %)	26%	11.1%	7.4%	14.0%	67.5%
4	$(dppbz)_2CoH$ (2 mol %)	48%	11.8%	6.7%	14.2%	67.3%

[a] Reaction conditions: buta-2,3-dien-2-ylbenzene (0.500 mmol), $PhSiH_3$ (0.550 mmol), THF (1 mL), RT, 18 h; [b] Overall yield of four products and product distribution determined by GC analysis with dodecane as the internal standard.

Supplementary Methods

General Remarks

All the manipulations were performed in an argon-filled glovebox, unless mentioned otherwise. THF, toluene, and hexane were purified by passing the degassed solvents (Ar) through a column of activated alumina (solvent purification system purchased from Innovative Technologies, Newburyport, MA). The following chemicals were purchased and used as received: $Co(acac)_2$ (99%, Sigma-Aldrich), $PhSiH_3$ (97%, Sigma-Aldrich), Ph_2SiH_2 (97%, Sigma-Aldrich), $PhMeSiH_2$ (98%, Sigma-Aldrich) , Et_2SiH_2 (99%, Sigma-Aldrich). ^{mes}PDI[1] was prepared according to previously reported procedures, and all phosphine ligands were purchased from commercial sources and used without further purification.

^{1}H, {^{1}H}^{13}C NMR spectra were recorded using Bruker 400 MHz and 500 MHz NMR spectrometer. ^{1}H NMR and {^{1}H}^{13}C NMR spectra were referenced to resonances of the residual protons in the deuterated solvents. Multiplicities are recorded as: s = singlet, d = doublet, t = triplet, dd = doublet of doublets, dt = doublet of triplets, br = broad singlet and m = multiplet. GC analysis was acquired on Agilent 6890N gas chromatography equipped with a flame-ionization detector. GC-MS analysis was performed on Shimadzu GC-2010 gas chromatography coupled to a Shimadzu QP2010 mass selective detector. HR-MS analyses were performed at a Thermo Scientific Exactive (APCI).

The ratio of Z/E isomers was determined by using GC System 6890N Network, Agilent Technologies. Samples were analysed on a capillary column of silice HP-5. The inlet: heater was 280 °C, the pressure was 8.21 psi, the total flow was 13.7 mL/min, split mode, the split ratio was 10:1, the split flow was 10 mL/min, and the mobile phase consisted in He gas. The GC measurement was conducted with a temperature program consisting of three steps: 80 ^{o}C for 2 min, ramp from 80–320 ^{o}C at the rate of 20 ^{o}C/min, and 320 ^{o}C for the remainder of the measurement. Each run ended after 18 min.

Cyclohexylallene and allenylboronic acid pinacol ester were purchased from Sigma-Aldrich Inc., and used as received.

The allene substrates for **1d**[2], **1e**[3], **1f**[4], **1g**[2], **1h**[2], **1k**[5], **1n-1q**[6], **3a-3h**[7], **3k**[8], **3l**[9], **3m**[10], **3n, 3o**[7] were prepared according to a general procedure reported by Baird.[2]

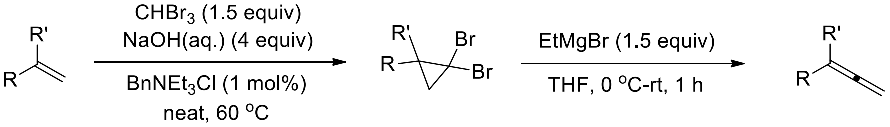

To a mixture of alkene (50 mmol), bromoform (6.6 mL, 75 mmol), and triethylbenzylammonium chloride (114 mg, 0.5 mmol) was added dropwise a solution of NaOH (8.0 g) in water (8.0 mL) over 1 h. The resulting mixture was stirred vigorously at 60 °C for 24 h, then cooled to room temperature, and quenched with water (50 mL). The mixture was extracted with dichloromethane and the organic phase was separated, dried over sodium sulfate, and concentrated. The residue was purified by column chromatography over silica gel with hexane as eluent to give 1,1-dibromocyclopropanes.

To a stirred solution of 1,1-dibromocyclopropane (45 mmol) in dry THF (50 mL) at 0 °C was added dropwise ethylmagnesium bromide (67.5 mL, 67.5 mmol, 1.0 M in THF) under nitrogen over 0.5 h. After stirred at 0 °C for 1 h, the mixture was quenched with 3M hydrochloric acid solution (20 mL) and diluted with ethyl ether (200 mL). The organic phase was washed with water (50 mL), dried over magnesium sulfate, and concentrated. The residue was purified by column chromatography over silica gel with hexane as eluent to give allenes.

(7-chlorohepta-1,2-dien-3-yl)benzene

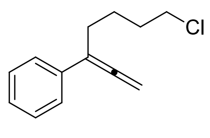

^{1}H NMR (400 MHz, $CDCl_3$) δ 7.40 (dd, *J* = 8.2, 1.0 Hz, 2H), 7.36–7.30 (m, 2H), 7.24–7.17 (m, 1H), 5.10 (s, 2H), 3.57 (t, *J* = 6.7 Hz, 2H), 2.46 (m, 2H), 1.89 (m, 2H), 1.77–1.68 (m, 2H); $^{13}C\{^1H\}$ NMR (101 MHz, $CDCl_3$) δ 208.7, 136.3, 128.6, 126.9, 126.1, 104.6, 78.6, 45.0, 32.4, 28.8, 25.2. GC-MS (EI): Calcd for $C_{13}H_{15}Cl$: 206.1, found: 206.1.

The allene substrates for **1h**[4], **1i**[5], **1k**[4] were prepared via Crabbé reaction from terminal alkynes.[11]

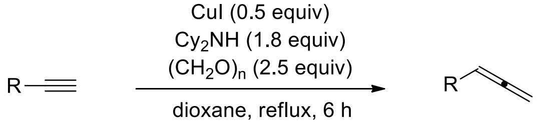

$(CH_2O)_n$ (5 mmol), CuI (1 mmol), dioxane (10 mL), alkyne (2 mmol), and amine (3.6 mmol) were added sequentially into anoven-dried reaction tube equipped with a reflux condenser under an argon atmosphere. The resulting mixture was stirred under reflux. When the reaction was complete as monitored by TLC, it was cooled to rt. Water (5 mL) and ether (10 mL) were added and then the aqueous solution was separated and extracted with ether (3 ×5 mL). The organic layer was then washed with brine and dried over anhydrous Na_2SO_4. Evaporation and column chromatographyon silica gel afforded the terminal allene.

The allene substrates for **3i**[12], **3j**, **3p**[7], **3q**[7] were prepared according to a procedure reported by Tsuji and co-workers.[13]

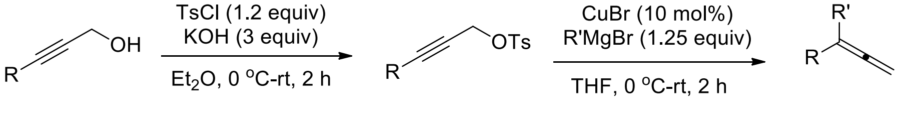

TsCl (1.2 eq.) was added to a solution of the corresponding propargylic alcohol in Et_2O (0.63 M). The reaction mixture was cooled to 0 °C, then KOH (3 eq.) was added in small portions. The solution was

allowed to warm to room temperature and stirred for 2h. The reaction mixture was poured on ice. The aqueous phase was extracted with Et_2O. The combined organic phases were washed with brine and dried over $MgSO_4$. The solvents were removed under reduced pressure and the crude product was purified by column chromatography, if necessary.

A mixture of CuBr (10 mol%), THF (0.5 M relative to the substrate) and the tosylate (1.0 eq.) was cooled to 0 °C. A corresponding Grignard reagent (1.25 eq.) was added drop wise. The reaction mixture was allowed to warm to room temperature and stirred for 2 h. It was quenched by addition of a saturated aqueous solution of NH_4Cl followed by extraction with Et_2O. The combined organic phases were washed with brine and dried over $MgSO_4$. The solvents were removed under reduced pressure and the crude product was purified by column chromatography.

2-methyl-2-(4-(penta-1,2-dien-3-yl)phenyl)-1,3-dioxolane

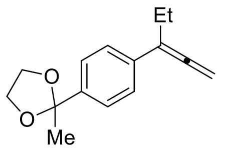

This substrate contains appreciable amount (≈15%) of internal alkyne 2-methyl-2-(4-(pent-1-yn-1-yl)phenyl)-1,3-dioxolane side product generated in the synthesis of this chemical. ^{1}H NMR (400 MHz, $CDCl_3$) δ 7.41 (m, 4H), 5.10 (m, 2H), 4.03 (m, 2H), 3.78 (m, 2H), 2.43 (m, 2H), 1.65 (s, 3H), 1.16 (t, J = 7.3 Hz, 3H); $^{13}C\{^1H\}$ NMR (101 MHz, $CDCl_3$) δ 208.6, 141.7, 136.3, 125.9, 125.5, 108.9, 106.6, 78.9, 64.6, 27.7, 22.6, 12.6. GC-MS (EI): Calcd for $C_{15}H_{18}O_2$: 230.1, found: 230.1.

Preparation of 1-iodo-4-(penta-3,4-dien-1-yloxy)benzene

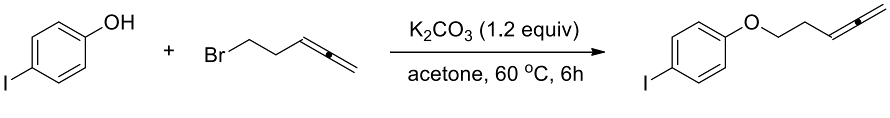

A solution of 4-iodophenol (2.20 g, 10.0 mmol) and K_2CO_3 (1.66 g, 12.0 mmol) in acetone (20 mL) was warmed to reflux and 5-bromopenta-1,2-diene (1.47 g, 10.0 mmol) was slowly added. After 6 h, the solvent was removed under reduced pressure. Column chromatography on silica gel afforded the product as colorless oil in 80% yield. ^{1}H NMR (400 MHz, $CDCl_3$) δ 7.57–7.52 (m, 2H), 6.70–6.65 (m, 2H), 5.20 (m, 1H), 4.73 (m, 2H), 4.00 (t, J = 6.7 Hz, 2H), 2.48 (m, 2H); $^{13}C\{^1H\}^{13}C$ NMR (101 MHz, $CDCl_3$) δ 209.2, 158.9, 138.3, 117.2, 86.2, 82.9, 75.6, 67.4, 28.3. GC-MS (EI): Calcd for $C_{11}H_{11}IO$: 286.0, found: 286.0.

General Procedure for the Co-catalyzed Hydrosilylation of Monosubstituted Terminal Allenes

In an Ar-filled dry box, $Co(acac)_2$ (2.6 mg, 10.0 μmol), rac-binap (6.2 mg, 10.0 μmol) and THF(1 mL) were added to a 4-mL screw-capped vial and stirred for 5 mins. Then monosubstituted terminal allenes (0.500 mmol) and $PhSiH_3$ (1.1 eq, 0.550 mmol) were added. The vial was sealed with a cap containing a PTFE septum and removed from the dry box. The reaction mixture was stirred at room temperature for 18 h and the resulting solution was concentrated in vacuum. The crude product was purified by column chromatography on silica gel with a mixture of ethyl acetate and hexane as eluent. The conditions for flash chromatography and data for characterization of the products are listed below.

(*Z*)-(3-cyclohexylallyl)(phenyl)silane (1a)

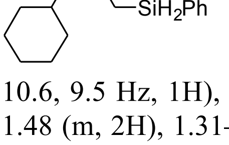

The title compound was isolated (97.8 mg, 0.425 mmol, 85%) as a colorless oil after chromatography on silica with ethyl acetate/hexane (1:20). ^{1}H NMR (400 MHz, $CDCl_3$) δ 7.64–7.56 (m, 2H), 7.45–7.34 (m, 3H), 5.42–5.30 (m, 1H), 5.21 (dd, J = 10.6, 9.5 Hz, 1H), 4.33 (t, J = 3.7 Hz, 2H), 2.24–2.11 (m, 1H), 1.91 (m, 2H), 1.74–1.63 (m, 3H), 1.56–1.48 (m, 2H), 1.31–1.15 (m, 3H), 1.03 (m, 2H); $^{13}C\{^1H\}$ NMR (101 MHz, $CDCl_3$) δ 135.8, 135.4, 132.3, 129.8, 128.1, 122.1, 36.3, 33.3, 26.2, 26.1, 12.0. GC-MS (EI): Calcd for $C_{15}H_{22}Si$: 230.1, found: 230.1. GC retention time, Z: 8.120 min; E: 8.255 min.

(*Z*)-(3-cyclohexylallyl)diphenylsilane (1b)

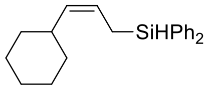

The title compound was isolated (127 mg, 0.415 mmol, 83%) as a colorless oil after chromatography on silica with ethyl acetate/hexane (1:20). ^{1}H NMR (400 MHz, $CDCl_3$) δ 7.65–7.50 (m, 4H), 7.46–7.30 (m, 6H), 5.40–5.30 (m, 1H), 5.15 (dd, *J* = 10.7, 9.6 Hz, 1H), 4.84 (t, *J* = 3.6 Hz, 1H), 2.17–2.06 (m, 3H), 1.69–1.57 (m, 3H), 1.40 (d, *J* = 13.2 Hz, 2H), 1.24–1.07 (m, 3H), 1.00–0.89 (m, 2H); ^{13}C{^{1}H} NMR (101 MHz, $CDCl_3$) δ 135.8, 135.4, 134.2, 129.8, 128.1, 121.7, 36.4, 33.2, 26.2, 26.1, 14.2. GC-MS (EI): Calcd for $C_{21}H_{26}Si$: 306.2, found: 306.2. GC retention time, *Z*: 10.096 min; *E*: 10.347 min.

(*Z*)-(3-cyclohexylallyl)(methyl)(phenyl)silane (1c)

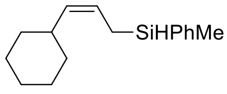

The title compound was isolated (105 mg, 0.430 mmol, 86%) as a colorless oil after chromatography on silica with ethyl acetate/hexane (1:20). ^{1}H NMR (400 MHz, $CDCl_3$) δ 7.59–7.50 (m, 2H), 7.43–7.30 (m, 3H), 5.34–5.25 (m, 1H), 5.15 (dd, *J* = 10.6, 9.5 Hz, 1H), 4.35 (m 1H), 2.17–2.07 (m, 1H), 1.85–1.74 (m, 2H), 1.71–1.60 (m, 3H), 1.49 (d, *J* = 12.8 Hz, 2H), 1.20 (m, 3H), 0.98 (m, 2H), 0.37 (d, *J* = 3.7 Hz, 3H); ^{13}C{^{1}H} NMR (101 MHz, $CDCl_3$) δ 136.2, 135.3, 134.5, 129.5, 128.0, 122.2, 36.3, 33.3, 33.3, 26.3, 26.2, 15.3, -5.9.. GC-MS (EI): Calcd for $C_{16}H_{24}Si$: 244.2, found: 244.2. GC retention time, *Z*: 8.365 min; *E*: 8.437 min.

(*Z*)-phenyl(5-phenylpent-2-en-1-yl)silane (1d)

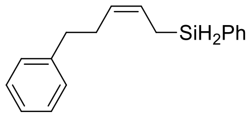

The title compound was isolated (111 mg, 0.440 mmol, 88%) as a colorless oil after chromatography on silica with ethyl acetate/hexane (1:20). ^{1}H NMR (400 MHz, $CDCl_3$) δ 7.60 (m, 2H), 7.44–7.36 (m, 3H), 7.33–7.28 (m, 2H), 7.24–7.16 (m, 3H), 5.58–5.48 (m, 1H), 5.42 (m, 1H), 4.32 (t, *J* = 3.7 Hz, 2H), 2.66–2.58 (m, 2H), 2.32 (m, 2H), 1.89 (m, 2H).; ^{13}C{^{1}H} NMR (101 MHz, $CDCl_3$) δ 142.2, 135.4, 132.2, 129.9, 128.5, 128.4, 128.4, 128.1, 128.1, 125.9, 124.9, 35.9, 29.1, 12.0, 1.2. HRMS (APCI$^+$) m/z calcd for $C_{17}H_{21}Si$, $[M+H]^+$ 253.1413, found: 253.1401. GC retention time, *Z*: 9.130 min; *E*: not found.

(*Z*)-non-2-en-1-yl(phenyl)silane (1e)

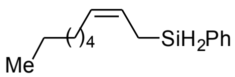

The title compound was isolated (104 mg, 0.448 mmol, 90%) as a colorless oil after chromatography on silica with ethyl acetate/hexane (1:20). ^{1}H NMR (400 MHz, $CDCl_3$) δ 7.60 (m, 2H), 7.44–7.34 (m, 3H), 5.52–5.42 (m, 1H), 5.41–5.31 (m, 1H), 4.31 (d, *J* = 3.7 Hz, 2H), 1.97 (d, *J* = 6.1 Hz, 2H), 1.89 (m, 2H), 1.28 (s, 9H), 0.90 (t, *J* = 6.9 Hz, 3H); ^{13}C{^{1}H} NMR (101 MHz, $CDCl_3$) δ 135.4, 132.4, 129.8, 129.8, 128.1, 124.0, 31.9, 29.7, 29.2, 27.2, 22.8, 14.2, 11.9. GC-MS (EI): Calcd for $C_{15}H_{24}Si$: 232.2, found: 232.2. GC retention time, *Z*: 10.096 min; *E*: 10.347 min.

(*Z*)-phenyl(undec-2-en-1-yl)silane (1f)

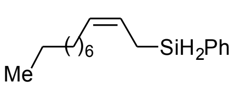

The title compound was isolated (118 mg, 0.453 mmol, 91%) as a colorless oil after chromatography on silica with ethyl acetate/hexane (1:20). ^{1}H NMR (400 MHz, $CDCl_3$) δ 7.59 (m, 2H), 7.43–7.33 (m, 3H), 5.53–5.41 (m, 1H), 5.41–5.29 (m, 1H), 4.32 (dd, *J* = 4.4, 3.1 Hz, 2H), 2.02–1.93 (m, 2H), 1.89 (m, 2H), 1.28 (s, 12H), 0.90 (d, *J* = 7.1 Hz, 3H); ^{13}C{^{1}H} NMR (101 MHz, $CDCl_3$) δ 135.4, 135.4, 132.4, 131.3, 129.9, 129.8, 129.8, 128.1, 128.1, 124.8, 124.0, 32.1, 29.8, 29.7, 29.6, 29.5, 27.2, 22.8, 14.3, 11.9. GC-MS (EI): Calcd for $C_{17}H_{28}Si$: 260.2, found: 260.2. GC retention time, *Z*: 7.925 min; *E*: not found.

(*Z*)-(7-chlorohept-2-en-1-yl)(phenyl)silane (1g)

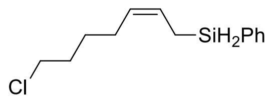

The title compound was isolated (95.2 mg, 0.400 mmol, 80%) as a colorless oil after chromatography on silica with ethyl acetate/hexane (1:20). ^{1}H NMR (400 MHz, $CDCl_3$) δ 7.59 (m, 2H), 7.41–7.34 (m, 3H), 5.50 (dt, *J* = 9.9, 8.5 Hz, 1H), 5.33 (dt, *J* = 10.7, 7.1 Hz, 1H), 4.31 (t, *J* = 3.7 Hz, 2H), 3.50 (t, *J* = 6.7 Hz, 2H), 1.99 (m, 2H), 1.91–1.83 (m, 2H), 1.78–1.70 (m, 2H), 1.43 (m, 2H); ^{13}C{^{1}H} NMR (101 MHz, $CDCl_3$) δ 135.4, 132.2, 129.9, 128.7, 128.1, 124.9, 45.1, 32.3, 26.9, 26.3, 12.0. GC-MS (EI): Calcd for $C_{13}H_{19}ClSi$: 238.1, found: 238.1. GC retention time, *Z*: 8.250 min; *E*: not found.

(*Z*)-(6-bromohex-2-en-1-yl)(phenyl)silane (1h)

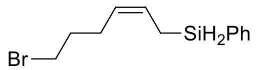

The title compound was isolated (104 mg, 0.387 mmol, 77%) as a pale yellow oil after chromatography on silica with ethyl acetate/hexane (1:20). ^{1}H NMR (400 MHz, $CDCl_3$) δ 7.63–7.56 (m, 2H), 7.43–7.34 (m, 3H), 5.61–5.46 (m, 1H), 5.35–5.23 (m, 1H), 4.32 (t, *J* = 3.7 Hz, 2H), 3.36 (t, *J* = 6.7 Hz, 2H), 2.12 (q, *J* = 6.8 Hz, 2H), 1.95–1.88 (m, 2H), 1.87–1.79 (m, 2H); ^{13}C{^{1}H} ^{13}C NMR (101 MHz, $CDCl_3$) δ 135.4, 132.0, 129.9, 128.1, 127.2, 126.0, 33.6, 32.6, 25.6, 12.1. HRMS (APCI$^+$) m/z calcd for $C_{12}H_{18}BrSi$, $[M+H]^+$ 269.0361, found: 269.0352. GC retention time, *Z*: 8.210 min; *E*: 8.475 min.

(*Z*)-(4-(4-iodophenoxy)but-2-en-1-yl)(phenyl)silane (1i)

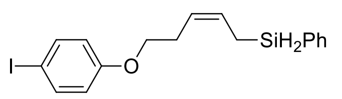

The title compound was isolated (112 mg, 0.284 mmol, 57%) as a colorless oil after chromatography on silica with ethyl acetate/hexane (1:20). ^{1}H NMR (400 MHz, $CDCl_3$) δ 7.60–7.52 (m, 4H), 7.42–7.33 (m, 3H), 6.66–6.59 (m, 2H), 5.69–5.58 (m, 1H), 5.46–5.37 (m, 1H), 4.32 (t, *J* = 3.6 Hz, 2H), 3.79 (t, *J* = 7.0 Hz, 2H), 2.47–2.39 (m, 2H), 1.97–1.88 (m, 2H); ^{13}C{^{1}H} NMR (101 MHz, $CDCl_3$) δ 158.9, 138.3, 135.4, 131.9, 130.0, 128.2, 127.3, 124.1, 117.1, 82.7, 67.6, 27.3, 12.3. HRMS (APCI$^+$) m/z calcd for $C_{17}H_{20}IOSi$, $[M+H]^+$ 395.0328, found: 395.0322. GC retention time, *Z*: 11.185 min; *E*: not found.

(*Z*)-tert-butyldimethyl((7-(phenylsilyl)hept-5-en-1-yl)oxy)silane (1j)

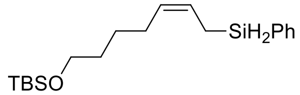

The title compound was isolated (154 mg, 0.461 mmol, 92%) as a colorless oil after chromatography on silica with ethyl acetate/hexane (1:10). ^{1}H NMR (400 MHz, $CDCl_3$) δ 7.62 (m, 2H), 7.41 (m, 3H), 5.57–5.45 (m, 1H), 5.40 (dt, *J* = 10.7, 7.1 Hz, 1H), 4.36 (t, *J* = 3.7 Hz, 2H), 3.64 (t, *J* = 6.5 Hz, 2H), 2.03 (m, 2H), 1.95–1.85 (m, 2H), 1.59–1.52 (m, 2H), 1.43–1.35 (m, 2H), 0.96 (s, 9H), 0.11 (s, 6H); ^{13}C{^{1}H} NMR (101 MHz, $CDCl_3$) δ 135.4, 132.3, 129.8, 129.5, 128.1, 124.2, 63.2, 32.7, 26.9, 26.1, 26.0, 18.5, 11.9, -5.1. GC-MS (EI): Calcd for $C_{19}H_{34}OSi_2$: 334.2, found: 334.2. GC retention time, *Z*: 9.440 min; *E*: not found.

(*Z*)-7-(phenylsilyl)hept-5-en-1-yl acetate (1k)

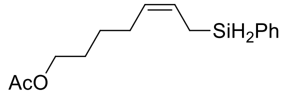

The title compound was isolated (104 mg, 0.397 mmol, 79%) as a colorless oil after chromatography on silica with ethyl acetate/hexane (1:10). ^{1}H NMR (400 MHz, $CDCl_3$) δ 7.57 (m, 2H), 7.40–7.33 (m, 3H), 5.58–5.41 (m, 1H), 5.38–5.26 (m, 1H), 4.33–4.23 (m, 2H), 4.03 (m, 2H), 2.04 (s, 3H), 1.98 (m, 2H), 1.91–1.82 (m, 2H), 1.58 (s, 2H), 1.38–1.31 (m, 2H); ^{13}C{^{1}H} NMR (101 MHz, $CDCl_3$) δ 171.4, 135.4, 132.2, 129.9, 128.9, 128.1, 124.8, 64.6, 28.4, 26.7, 26.0, 21.1, 12.0. HRMS (APCI$^+$) m/z calcd for $C_{15}H_{23}O_2Si$, $[M+H]^+$ 263.1467, found: 263.1464. GC retention time, *Z*: 8.745 min; *E*: not found.

(*Z*)-phenyl(3-(4,4,5,5-tetramethyl-1,3,2-dioxaborolan-2-yl)allyl)silane (1l)

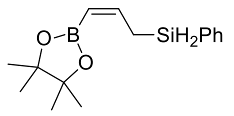

The title compound was isolated (112 mg, 0.409 mmol, 82%) as a colorless oil after chromatography on silica with ethyl acetate/hexane (1:20). ^{1}H NMR (400 MHz, $CDCl_3$) δ 7.64–7.59 (m, 2H), 7.41–7.34 (m, 3H), 6.61 (dt, *J* = 13.1, 8.6 Hz, 1H),

5.32 (d, J = 13.3 Hz, 1H), 4.34 (t, J = 3.6 Hz, 2H), 2.48–2.40 (m, 2H), 1.25 (s, 12H); $^{13}C\{^1H\}$ NMR (101 MHz, $CDCl_3$) δ 150.9, 135.4, 132.1, 129.8, 128.0, 82.8, 25.0, 18.3. HRMS ($APCI^+$) m/z calcd for $C_{15}H_{24}BO_2Si$, $[M+H]^+$ 275.1639, found: 275.1629. GC retention time, Z: 8.160 min; E: 8.295 min.

(*Z*)-2-(6-(phenylsilyl)hex-4-en-1-yl)isoindoline-1,3-dione (1m)

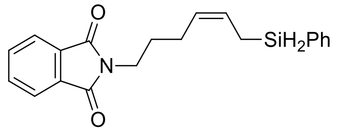

The title compound was isolated (141 mg, 0.421 mmol, 84%) as a white solid after chromatography on silica with ethyl acetate/hexane (1:7). ^1H NMR (400 MHz, $CDCl_3$) δ 7.84 (m, 2H), 7.70 (m, 2H), 7.57–7.52 (m, 2H), 7.37–7.30 (m, 3H), 5.55–5.43 (m, 1H), 5.39–5.27 (m, 1H), 4.27 (t, J = 3.6 Hz, 2H), 3.66–3.60 (m, 2H), 2.01 (m, 2H), 1.90–1.81 (m, 2H), 1.65 (m, 2H); $^{13}C\{^1H\}$ NMR (101 MHz, $CDCl_3$) δ 168.5, 135.4, 134.0, 132.3, 129.8, 128.1, 127.9, 125.3, 123.3, 37.9, 28.5, 24.6, 12.1. HRMS ($APCI^+$) m/z calcd for $C_{20}H_{22}NO_2Si$, $[M+H]^+$ 336.1420, found: 336.1411. GC retention time, Z: 14.035 min; E: 14.416 min.

(*Z*)-phenyl(3-phenylallyl)silane (1n)

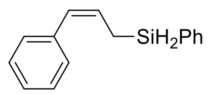

The title compound was isolated (88.5 mg, 0.395 mmol, 79%) as a colorless oil after chromatography on silica with ethyl acetate/hexane (1:20). ^1H NMR (400 MHz, $CDCl_3$) δ 7.61 (dd, J = 7.8, 1.4 Hz, 2H), 7.45–7.30 (m, 5H), 7.30–7.20 (m, 3H), 6.43 (d, J = 11.5 Hz, 1H), 5.79 (dt, J = 11.4, 8.9 Hz, 1H), 4.43 (t, J = 3.6 Hz, 2H), 2.26–2.17 (m, 2H); $^{13}C\{^1H\}$ NMR (101 MHz, $CDCl_3$) δ 137.7, 135.4, 131.7, 130.0, 128.7, 128.5, 128.3, 128.2, 127.5, 126.5, 13.3. HRMS ($APCI^+$) m/z calcd for $C_{15}H_{17}Si$, $[M+H]^+$ 225.1100, found: 225.1098. GC retention time, Z: 8.355 min; E: not found.

(*Z*)-(3-(4-methoxyphenyl)allyl)(phenyl)silane (1o)

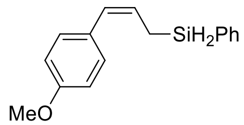

The title compound was isolated (103 mg, 0.405 mmol, 81%) as a colorless oil after chromatography on silica with ethyl acetate/hexane (1:10). ^1H NMR (400 MHz, $CDCl_3$) δ 7.63–7.56 (m, 2H), 7.38 (m, 3H), 7.20 (d, J = 8.6 Hz, 2H), 6.89–6.83 (m, 2H), 6.35 (d, J = 11.5 Hz, 1H), 5.69 (dt, J = 11.4, 8.9 Hz, 1H), 4.41 (t, J = 3.7 Hz, 2H), 3.82 (s, 3H), 2.19 (m, 2H); $^{13}C\{^1H\}$ NMR (101 MHz, $CDCl_3$) δ 158.3, 135.4, 131.8, 130.4, 130.0, 129.9, 128.2, 128.0, 126.0, 113.8, 55.4, 13.2. HRMS ($APCI^+$) m/z calcd for $C_{16}H_{19}OSi$, $[M+H]^+$ 255.1205, found: 255.1202. GC retention time, Z: 9.325 min; E: 9.620 min.

(*Z*)-(3-(4-bromophenyl)allyl)(phenyl)silane (1p)

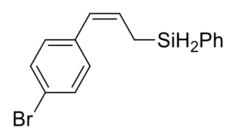

The title compound was isolated (112 mg, 0.371 mmol, 74%) as a colorless oil after chromatography on silica with ethyl acetate/hexane (1:20). ^1H NMR (400 MHz, $CDCl_3$) δ 7.56 (m, 2H), 7.45–7.32 (m, 5H), 7.07 (d, J = 8.4 Hz, 2H), 6.31 (d, J = 11.5 Hz, 1H), 5.79 (dt, J = 11.5, 8.9 Hz, 1H), 4.38 (t, J = 3.6 Hz, 2H), 2.14 (m, 2H); $^{13}C\{^1H\}$ NMR (101 MHz, $CDCl_3$) δ 136.6, 135.4, 131.4, 130.3, 130.1, 128.5, 128.3, 127.3, 121.0, 120.3, 13.4. HRMS ($APCI^+$) m/z calcd for $C_{15}H_{16}BrSi$, $[M+H]^+$ 303.0205, found: 303.0203. GC retention time, Z: 9.705 min; E: not found.

(*Z*)-(3-(4-(tert-butyl)phenyl)allyl)(phenyl)silane (1q)

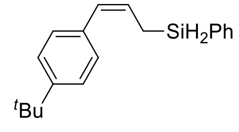

The title compound was isolated (112 mg, 0.400 mmol, 80%) as a colorless oil after chromatography on silica with ethyl acetate/hexane (1:20). ^1H NMR (400 MHz, $CDCl_3$) δ 7.63–7.54 (m, 2H), 7.43–7.32 (m, 5H), 7.21 (d, J = 8.3 Hz, 2H), 6.37 (d, J = 11.5 Hz, 1H), 5.73 (dt, J = 11.5, 8.9 Hz, 1H), 4.42 (t, J = 3.7 Hz, 2H), 2.22 (m,

2H), 1.33 (s, 9H); $^{13}C\{^1H\}$ NMR (101 MHz, $CDCl_3$) δ 149.5, 135.4, 134.9, 131.8, 130.0, 128.5, 128.3, 128.2, 126.9, 125.3, 34.6, 31.5, 13.4. HRMS ($APCI^+$) m/z calcd for $C_{19}H_{25}Si$, $[M+H]^+$ 281.1726, found: 281.1713. GC retention time, *Z*: 9.642 min; *E*: not found.

bis((*Z*)-3-cyclohexylallyl)(phenyl)silane (2)

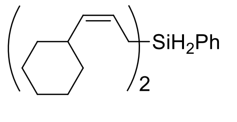

The title compound was isolated as a colorless oil after chromatography on silica with ethyl acetate/hexane (1:20). ^1H NMR (400 MHz, $CDCl_3$) δ 7.58–7.53 (m, 2H), 7.35 (m, 3H), 5.36–5.27 (m, 2H), 5.14 (dd, *J* = 10.6, 9.5 Hz, 2H), 4.24 (p, *J* = 3.3 Hz, 1H), 2.19–2.07 (m, 2H), 1.84 (m, 4H), 1.63 (m, 6H), 1.47 (m, 4H), 1.28–1.13 (m, 6H), 1.03–0.93 (m, 4H); $^{13}C\{^1H\}$ NMR (101 MHz, $CDCl_3$) δ 135.5, 134.9, 134.9, 129.6, 127.9, 122.1, 36.3, 33.3, 26.2, 26.2, 13.7. HRMS ($APCI^+$) m/z calcd for $C_{24}H_{37}Si$, $[M+H]^+$ 353.2665, found: 353.2660.

General Procedure for the Co-catalyzed Hydrosilylation of Disubstituted Termial Allenes

In an Ar-filled dry box, $Co(acac)_2$ (1.3 mg, 5.0 μmol), xantphos (2.9 mg, 5.0 μmol) and THF (1mL) were added to a 4-mL screw-capped vial and stirred for 5 mins. Then disubstituted terminal allenes (0.500 mmol) and $PhSiH_3$ (1.1 eq, 0.550 mmol) were added. The vial was sealed with a cap containing a PTFE septum and removed from the dry box. The reaction mixture was stirred at room temperature for 3 h and the resulting solution was concentrated in vacuum. The crude product was purified by column chromatography on silica gel with a mixture of ethyl acetate and hexane as eluent. The conditions for flash chromatography and data for characterization of the products are listed below.

(*Z*)-phenyl(3-phenylbut-2-en-1-yl)silane (3a)

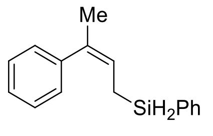

The title compound was isolated (112 mg, 0.470 mmol, 94%) as a colorless oil after chromatography on silica with ethyl acetate/hexane (1:20). ^1H NMR (400 MHz, $CDCl_3$) δ 7.51 (m, 2H), 7.43–7.27 (m, 5H), 7.23 (m, 1H), 7.15–7.09 (m, 2H), 5.55 (td, *J* = 8.4, 1.3 Hz, 1H), 4.29 (t, *J* = 3.6 Hz, 2H), 2.02 (d, *J* = 1.1 Hz, 3H), 1.87–1.75 (m, 2H); $^{13}C\{^1H\}$ NMR (101 MHz, $CDCl_3$) δ 142.2, 136.3, 135.4, 132.2, 129.8, 128.3, 128.1, 128.1, 126.5, 121.8, 25.9, 13.1. HRMS ($APCI^+$) m/z calcd for $C_{16}H_{19}Si$, $[M+H]^+$ 239.1256, found: 239.1253. GC retention time, *Z*: 8.320 min; *E*: not found.

(*Z*)-phenyl(3-(4-(trifluoromethyl)phenyl)but-2-en-1-yl)silane (3b)

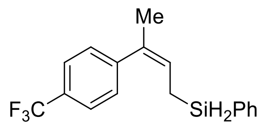

The title compound was isolated (122 mg, 0.399 mmol, 80%) as a colorless oil after chromatography on silica with ethyl acetate/hexane (1:20). ^1H NMR (400 MHz, $CDCl_3$) δ 7.56 (d, *J* = 8.2 Hz, 2H), 7.53–7.47 (m, 2H), 7.45–7.40 (m, 1H), 7.35 (m, 2H), 7.18 (d, *J* = 8.1 Hz, 2H), 5.63 (t, *J* = 8.5 Hz, 1H), 4.30 (t, *J* = 3.5 Hz, 2H), 2.02 (d, *J* = 0.8 Hz, 3H), 1.83–1.76 (m, 2H); $^{13}C\{^1H\}$NMR (101 MHz, $CDCl_3$) δ 145.9, 135.4, 134.9, 131.8, 129.9, 128.7 (q, *J* = 32.4 Hz), 128.5, 128.2, 125.3 (q, *J* = 3.7 Hz), 124.5 (q, *J* = 272 Hz), 25.6, 13.3. HRMS ($APCI^+$) m/z calcd for $C_{17}H_{18}F_3Si$, $[M+H]^+$ 307.1130, found: 307.1130. GC retention time, *Z*: 8.140 min; *E*: not found.

(*Z*)-(3-(2-methoxyphenyl)but-2-en-1-yl)(phenyl)silane (3c)

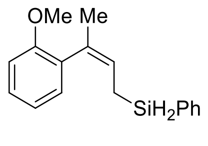

The title compound was isolated (123 mg, 0.459 mmol, 92%) as a colorless oil after chromatography on silica with ethyl acetate/hexane (1:10). ^1H NMR (400 MHz, $CDCl_3$) δ 7.46–7.35 (m, 2H), 7.30–7.20 (m, 3H), 7.17–7.11 (m, 1H), 6.87–6.76 (m, 3H), 5.51 (td, *J* = 8.2, 1.4 Hz, 1H), 4.16 (t, *J* = 3.7 Hz, 2H), 3.70 (s, 3H), 1.90 (d, *J* = 1.2 Hz, 3H), 1.59 (m, 2H); $^{13}C\{^1H\}$ NMR (101 MHz, $CDCl_3$) δ 156.6, 135.4, 133.9, 132.5, 130.8, 130.1, 129.7, 128.1, 128.0, 122.6, 120.6, 111.0, 55.5, 24.8, 13.2. HRMS ($APCI^+$) m/z calcd for $C_{17}H_{21}OSi$, $[M+H]^+$ 269.1362, found: 269.1358. GC retention time, *Z*: 9.505 min; *E*: not found.

(*Z*)-(3-(naphthalen-2-yl)but-2-en-1-yl)(phenyl)silane (3d)

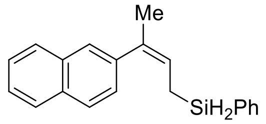

The title compound was isolated (133 mg, 0.462 mmol, 92%) as a colorless oil after chromatography on silica with ethyl acetate/hexane (1:20). ^{1}H NMR (400 MHz, $CDCl_3$) δ 7.87–7.83 (m, 1H), 7.81 (d, *J* = 8.5 Hz, 1H), 7.76 (m, 1H), 7.56–7.50 (m, 3H), 7.50–7.45 (m, 2H), 7.41 (m, 1H), 7.39–7.32 (m, 2H), 7.28 (m, 1H), 5.66 (td, *J* = 8.4, 1.4 Hz, 1H), 4.34 (t, *J* = 3.6 Hz, 2H), 2.12 (d, *J* = 1.2 Hz, 3H), 1.89 (m, 2H); ^{13}C{^{1}H} NMR (101 MHz, $CDCl_3$) δ 139.6, 136.2, 135.4, 133.5, 132.4, 132.1, 129.8, 128.1, 128.0, 127.8, 127.7, 126.8, 126.6, 126.0, 125.6, 122.3, 25.9, 13.3. HRMS (APCI^{+}) m/z calcd for $C_{20}H_{21}Si$, $[M+H]^{+}$ 289.1413, found: 289.1416. GC retention time, *Z*: 10.305 min; *E*: not found.

(*Z*)-(3-(benzo[d][1,3]dioxol-5-yl)but-2-en-1-yl)(phenyl)silane (3e)

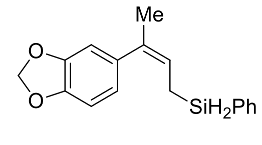

The title compound was isolated (124 mg, 0.440 mmol, 88%) as a colorless oil after chromatography on silica with ethyl acetate/hexane (1:10). ^{1}H NMR (400 MHz, $CDCl_3$) δ 7.54–7.48 (m, 2H), 7.41–7.31 (m, 3H), 6.75 (d, *J* = 8.0 Hz, 1H), 6.59–6.52 (m, 2H), 5.94 (s, 2H), 5.49 (td, *J* = 8.4, 1.4 Hz, 1H), 4.27 (t, *J* = 3.7 Hz, 2H), 1.96 (d, *J* = 1.3 Hz, 3H), 1.82 (m, 2H); ^{13}C{^{1}H} NMR (101 MHz, $CDCl_3$) δ 147.5, 146.1, 136.0, 135.8, 135.4, 132.1, 129.8, 128.1, 121.8, 121.2, 108.7, 108.2, 100.9, 26.0, 13.1. HRMS (APCI^{+}) m/z calcd for $C_{17}H_{19}O_2Si$, $[M+H]^{+}$ 283.1154, found: 283.1148. GC retention time, *Z*: 9.585 min; *E*: 9.870 min.

(*Z*)-tert-butyldimethyl(4-(4-(phenylsilyl)but-2-en-2-yl)phenoxy)silane (3f)

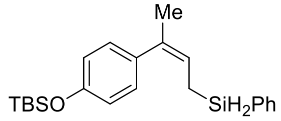

The title compound was isolated (136 mg, 0.369 mmol, 74%) as a colorless oil after chromatography on silica with ethyl acetate/hexane (1:10). ^{1}H NMR (400 MHz, $CDCl_3$) δ 7.29 (m, 2H), 7.21–7.16 (m, 1H), 7.12 (m, 2H), 6.79–6.73 (m, 2H), 6.60–6.52 (m, 2H), 5.28 (td, *J* = 8.4, 1.3 Hz, 1H), 4.06 (t, *J* = 3.7 Hz, 2H), 1.64–1.58 (m, 2H), 1.34 (s, 3H), 0.78 (d, *J* = 3.1 Hz, 9H), 0.00 (s, 6H); ^{13}C{^{1}H} NMR (101 MHz, $CDCl_3$) δ 154.2, 135.9, 135.4, 135.0, 132.3, 129.8, 129.1, 128.1, 121.4, 119.8, 25.9, 25.9, 18.4, 13.1, -4.2. HRMS (APCI^{+}) m/z calcd for $C_{22}H_{33}OSi_2$, $[M+H]^{+}$ 369.2070, found: 369.2066. GC retention time, *Z*: 8.265 min; *E*: not found.

(*Z*)-(3-(4-chlorophenyl)but-2-en-1-yl)(phenyl)silane (3g)

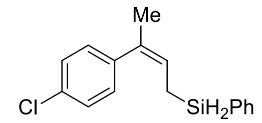

The title compound was isolated (121 mg, 0.445 mmol, 89%) as a colorless oil after chromatography on silica with ethyl acetate/hexane (1:20). ^{1}H NMR (400 MHz, $CDCl_3$) δ 7.55–7.47 (m, 2H), 7.44–7.32 (m, 3H), 7.30–7.24 (m, 2H), 7.07–6.96 (m, 2H), 5.57 (td, *J* = 8.5, 1.4 Hz, 1H), 4.29 (t, *J* = 3.6 Hz, 2H), 1.99 (d, *J* = 1.2 Hz, 3H), 1.80 (m, 2H); ^{13}C{^{1}H} NMR (101 MHz, $CDCl_3$) δ 140.5, 135.4, 135.0, 132.3, 131.9, 129.9, 129.5, 128.5, 128.1, 122.6, 25.7, 13.2. HRMS (APCI^{+}) m/z calcd for $C_{16}H_{18}ClSi$, $[M+H]^{+}$ 273.0866, found: 273.0869. GC retention time, *Z*: 9.590 min; *E*: not found.

(*Z*)-(3-(4-bromophenyl)but-2-en-1-yl)(phenyl)silane (3h)

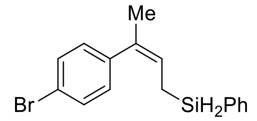

The title compound was isolated (139 mg, 0.440 mmol, 88%) as a colorless oil after chromatography on silica with ethyl acetate/hexane (1:20). ^{1}H NMR (400 MHz, $CDCl_3$) δ 7.53–7.49 (m, 2H), 7.45–7.40 (m, 3H), 7.40–7.33 (m, 2H), 6.99–6.93 (m, 2H), 5.57 (m, 1H), 4.30 (t, *J* = 3.6 Hz, 2H), 1.99 (m, 3H), 1.80 (m, 2H); ^{13}C{^{1}H} NMR (101 MHz, $CDCl_3$) δ 141.0, 135.4, 135.0, 131.9, 131.4, 129.9, 128.9, 128.1, 122.6, 120.4, 25.7, 13.3. HRMS (ESI) m/z calcd for $C_{16}H_{18}BrSi$, $[M+H]^{+}$ 317.0361, found: 317.0357. GC retention time, *Z*: 9.640 min; *E*: not found.

(*Z*)-ethyl 4-(1-(phenylsilyl)pent-2-en-3-yl)benzoate (3i)

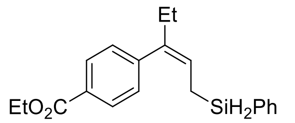

The title compound was isolated (135 mg, 0.415 mmol, 83%) as a colorless oil after chromatography on silica with ethyl acetate/hexane (1:10). ^{1}H NMR (400 MHz, $CDCl_3$) δ 7.98 (d, *J* = 8.1 Hz, 2H), 7.49 (m, 2H), 7.41–7.38 (m, 1H), 7.35 (d, *J* = 7.5 Hz, 2H), 7.08 (d, *J* = 8.2 Hz, 2H), 5.56 (t, *J* = 8.4 Hz, 1H), 4.42–4.36 (m, 2H), 4.27 (t, *J* = 3.6 Hz, 2H), 2.33 (q, *J* = 7.4 Hz, 2H), 1.75 (m, 2H), 1.41 (t, *J* = 7.1 Hz, 3H), 0.93 (t, *J* = 7.4 Hz, 3H); $^{13}C\{^1H\}$ NMR (101 MHz, $CDCl_3$) δ 166.8, 146.4, 141.9, 135.4, 131.9, 129.9, 129.6, 128.7, 128.7, 128.1, 121.3, 61.0, 32.2, 14.5, 13.3, 13.1. HRMS (ESI) m/z calcd for $C_{20}H_{25}O_2Si$, $[M+H]^+$ 325.1624, found: 325.1622. GC retention time, *Z*: 10.330 min; *E*: not found.

(*Z*)-(3-(4-(2-methyl-1,3-dioxolan-2-yl)phenyl)pent-2-en-1-yl)(phenyl)silane (3j)

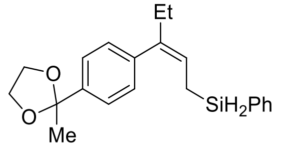

The title compound was isolated (142 mg, 0.420 mmol, 84%) as a colorless oil after chromatography on silica with ethyl acetate/hexane (1:10) (contains appreciable amount (≈10%) of hydrosilylated byproduct of internal alkyne from the starting material). ^{1}H NMR (400 MHz, $CDCl_3$) δ 7.49 (m, 2H), 7.39 (d, *J* = 8.3 Hz, 3H), 7.34 (d, *J* = 7.3 Hz, 2H), 7.00 (d, *J* = 8.3 Hz, 2H), 5.50 (t, *J* = 8.4 Hz, 1H), 4.27 (t, *J* = 3.6 Hz, 2H), 4.06–4.02 (m, 2H), 3.82 (m, 2H), 2.31 (m, 2H), 1.76 (dt, *J* = 8.1, 3.6 Hz, 2H), 1.67 (s, 3H), 0.93 (t, *J* = 7.4 Hz, 3H); $^{13}C\{^1H\}$ ^{1}NMR (101 MHz, $CDCl_3$) δ 142.5, 141.3, 135.7, 135.4, 132.2, 129.8, 128.4, 128.1, 125.1, 120.4, 109.1, 64.7, 32.4, 27.7, 13.4, 12.9. GC-MS (EI): Calcd for $C_{21}H_{26}O_2Si$: 338.2, found: 338.1. GC retention time, *Z*: 10.320 min; *E*: 10.895 min.

(*Z*)-phenyl(3-phenylhex-2-en-1-yl)silane (3k)

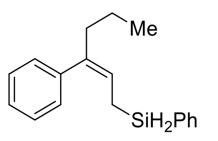

The title compound was isolated (120 mg, 0.451 mmol, 90%) as a colorless oil after chromatography on silica with ethyl acetate/hexane (1:20). ^{1}H NMR (400 MHz, $CDCl_3$) δ 7.51 (m, 2H), 7.42–7.27 (m, 5H), 7.25–7.19 (m, 1H), 7.10–6.98 (m, 2H), 5.52 (t, *J* = 8.4 Hz, 1H), 4.29 (t, *J* = 3.6 Hz, 2H), 2.29 (t, *J* = 7.4 Hz, 2H), 1.81–1.74 (m, 2H), 1.35–1.25 (m, 2H), 0.86 (t, *J* = 7.3 Hz, 3H); $^{13}C\{^1H\}$ NMR (101 MHz, $CDCl_3$) δ 141.3, 141.0, 135.4, 132.2, 129.8, 128.6, 128.2, 128.1, 126.4, 121.5, 41.7, 21.4, 13.7, 13.0. HRMS ($APCI^+$) m/z calcd for $C_{18}H_{23}Si$, $[M+H]^+$ 267.1569, found: 267.1569. GC retention time, *Z*: 8.890 min; *E*: 9.215 min.

(*Z*)-phenyl(3-phenylnon-2-en-1-yl)silane (3l)

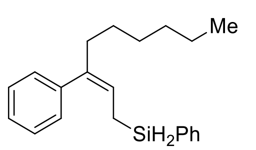

The title compound was isolated (140 mg, 0.454 mmol, 91%) as a colorless oil after chromatography on silica with ethyl acetate/hexane (1:20). ^{1}H NMR (400 MHz, $CDCl_3$) δ 7.53–7.48 (m, 2H), 7.41–7.27 (m, 5H), 7.22 (m, 1H), 7.04 (d, *J* = 7.2 Hz, 2H), 5.51 (t, *J* = 8.4 Hz, 1H), 4.28 (t, *J* = 3.5 Hz, 2H), 2.30 (d, *J* = 6.2 Hz, 2H), 1.82–1.73 (m, 2H), 1.25 (d, *J* = 5.0 Hz, 8H), 0.87 (t, *J* = 6.8 Hz, 3H); $^{13}C\{^1H\}$ NMR (101 MHz, $CDCl_3$) δ 141.4, 141.2, 135.4, 132.2, 129.8, 128.6, 128.2, 128.1, 126.4, 121.3, 39.6, 31.8, 28.9, 28.3, 22.8, 14.2, 13.0. HRMS ($APCI^+$) m/z calcd for $C_{21}H_{29}Si$, $[M+H]^+$ 309.2039, found: 309.2040. GC retention time, *Z*: 9.845 min; *E*: not found.

(*Z*)-(3,4-diphenylbut-2-en-1-yl)(phenyl)silane (3m)

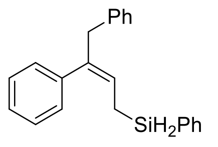

The title compound was isolated (130 mg, 0.413 mmol, 83%) as a colorless oil after chromatography on silica with ethyl acetate/hexane (1:20). ^{1}H NMR (400 MHz, $CDCl_3$) δ 7.48 (m, 2H), 7.42–7.37 (m, 1H), 7.36–7.30 (m, 2H), 7.24–7.13 (m, 6H), 7.11–7.03 (m, 2H), 6.97–6.90 (m, 2H), 5.55 (t, *J* = 8.4 Hz, 1H), 4.28 (t, *J* = 3.6 Hz, 2H), 3.60 (s, 2H), 1.80 (m, 2H); $^{13}C\{^1H\}$ NMR (101 MHz, $CDCl_3$) δ 140.8, 140.1, 139.9, 135.5, 132.0, 129.8, 129.2, 128.7, 128.2, 128.2, 128.1, 126.6, 126.0, 123.8, 46.0, 13.3. HRMS

(APCI$^+$) m/z calcd for $C_{22}H_{23}Si$, $[M+H]^+$ 315.1569, found: 315.1570. GC retention time, *Z*: 10.495 min; *E*: 10.875 min.

(*Z*)-(7-chloro-3-phenylhept-2-en-1-yl)(phenyl)silane (3n)

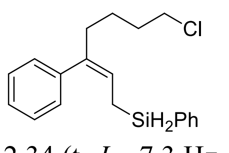

The title compound was isolated (130 mg, 0.414 mmol, 83%) as a colorless oil after chromatography on silica with ethyl acetate/hexane (1:20). ^{1}H NMR (400 MHz, $CDCl_3$) δ 7.54–7.45 (m, 2H), 7.42–7.27 (m, 5H), 7.23 (d, *J* = 7.3 Hz, 1H), 7.05–6.98 (m, 2H), 5.52 (t, *J* = 8.4 Hz, 1H), 4.27 (t, *J* = 3.6 Hz, 2H), 3.47 (t, *J* = 6.8 Hz, 2H), 2.34 (t, *J* = 7.3 Hz, 2H), 1.79–1.67 (m, 4H), 1.43–1.34 (m, 2H); $^{13}C\{^1H\}$ NMR (101 MHz, $CDCl_3$) δ 140.8, 140.2, 135.4, 132.1, 129.8, 128.6, 128.3, 128.1, 126.6, 122.2, 45.1, 38.7, 32.1, 25.5, 13.1. HRMS (APCI$^+$) m/z calcd for $C_{19}H_{24}ClSi$, $[M+H]^+$ 315.1336, found: 315.1332. GC retention time, *Z*: 10.215 min; *E*: 10.455 min.

(*Z*)-(3-(furan-2-yl)but-2-en-1-yl)(phenyl)silane (3o)

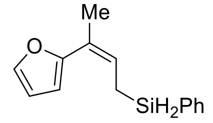

The title compound was isolated (98.1 mg, 0.430 mmol, 86%) as a pale yellow oil after chromatography on silica with ethyl acetate/hexane (1:10). ^{1}H NMR (400 MHz, $CDCl_3$) δ 7.63–7.58 (m, 2H), 7.41–7.33 (m, 4H), 6.40 (m, 1H), 6.25 (d, *J* = 3.3 Hz, 1H), 5.54 (td, *J* = 9.0, 1.2 Hz, 1H), 4.35 (t, *J* = 3.7 Hz, 2H), 2.40–2.35 (m, 2H), 2.01 (d, *J* = 0.9 Hz, 3H); $^{13}C\{^1H\}$ NMR (101 MHz, $CDCl_3$) δ 155.5, 141.2, 135.3, 132.5, 129.8, 128.1, 123.4, 123.2, 110.7, 107.6, 21.9, 14.2. HRMS (APCI$^+$) m/z calcd for $C_{14}H_{17}OSi$, $[M+H]^+$ 229.1049, found: 229.1040. GC retention time, *Z*: 7.900 min; *E*: not found.

(*Z*)-(3-cyclohexylbut-2-en-1-yl)(phenyl)silane (3p)

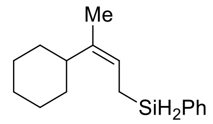

The title compound was isolated (103 mg, 0.459 mmol, 92%) as a colorless oil after chromatography on silica with ethyl acetate/hexane (1:10). ^{1}H NMR (400 MHz, $CDCl_3$) δ 7.66–7.57 (m, 2H), 7.46–7.35 (m, 3H), 5.19 (td, *J* = 8.3, 1.1 Hz, 1H), 4.33 (t, *J* = 3.7 Hz, 2H), 2.35 (m, 1H), 1.91–1.83 (m, 2H), 1.76 (d, *J* = 5.2 Hz, 2H), 1.73–1.68 (m, 1H), 1.65 (d, *J* = 1.1 Hz, 3H), 1.41 (d, *J* = 8.1 Hz, 2H), 1.33–1.25 (m, 4H), 1.19 (m, 1H); $^{13}C\{^1H\}$ NMR (101 MHz, $CDCl_3$) δ 140.0, 135.4, 132.7, 129.7, 128.0, 117.9, 39.6, 31.0, 26.8, 26.4, 19.7, 11.5. GC-MS (EI): Calcd for $C_{16}H_{24}Si$: 224.2, found: 224.2. GC retention time, *Z*: 8.420 min; *E*: 8.585 min.

(*Z*)-(3-methylnon-2-en-1-yl)(phenyl)silane (3q)

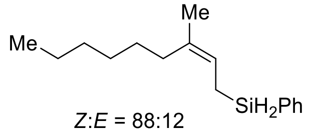

The title compound was isolated (117 mg, 0.475 mmol, 95%) as a colorless oil after chromatography on silica with ethyl acetate/hexane (1:20). ^{1}H NMR (400 MHz, $CDCl_3$) δ 7.62–7.58 (*Z*: m, 1.71H), 7.55 (*E*: m, 0.28H), 7.44–7.32 (m, 3H), 5.29–5.14 (m, 1H), 4.30 (t, *J* = 3.8 Hz, 2H), 2.02–1.94 (m, 2H), 1.85–1.80 (*Z*: m, 1.76H), 1.77 (*E*: m, 0.24H), 1.70 (d, *J* = 1.1 Hz, 2H), 1.68 (d, *J* = 1.0 Hz, 1H), 1.34–1.22 (m, 8H), 0.91 (t, *J* = 6.9 Hz, 3H).; $^{13}C\{^1H\}$ ^{13}C NMR (101 MHz, $CDCl_3$) δ 135.56, 135.38, 135.20, 134.83, 132.73, 132.67, 129.73, 129.70, 128.07, 128.03, 118.85, 118.46, 39.90, 32.02, 31.96, 31.75, 29.63, 29.59, 29.03, 28.18, 28.01, 27.96, 23.54, 22.81, 15.83, 14.26, 12.05, 11.86. HRMS (APCI$^+$) m/z calcd for $C_{16}H_{27}Si$, $[M+H]^+$ 247.1882, found: 247.1868. GC retention time, *Z*: 8.115 min; *E*: 8.225 min.

((*Z*)-2-((8S,9S,13S,14S)-3-methoxy-13-methyl-7,8,9,11,12,13,15,16-octahydro-6H-cyclopenta[a]phenanthren-17(14H)-ylidene)ethyl)(phenyl)silane (3r)

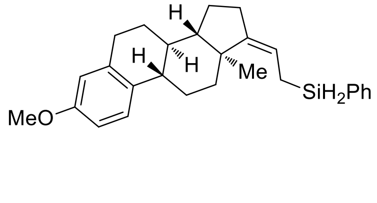

The title compound was isolated (165 mg, 0.410 mmol, 82%) as a white solid after chromatography on silica with ethyl acetate/hexane (1:7). ^{1}H

NMR (400 MHz, $CDCl_3$) δ 7.56–7.43 (m, 2H), 7.35–7.20 (m, 3H), 7.10 (d, *J* = 8.7 Hz, 1H), 6.62 (m, 1H), 6.54 (d, *J* = 2.5 Hz, 1H), 5.06 (m, 1H), 4.24 (t, *J* = 3.7 Hz, 2H), 3.68 (s, 3H), 2.83–2.70 (m, 2H), 2.36 (m, 1H), 2.29–2.06 (m, 4H), 1.98–1.86 (m, 2H), 1.82 (m, 1H), 1.63 (m, 2H), 1.49–1.41 (m, 1H), 1.32–1.17 (m, 4H), 0.79 (s, 3H); $^{13}C\{^1H\}$ NMR (101 MHz, $CDCl_3$) δ 157.6, 149.0, 138.1, 135.4, 132.9, 132.6, 129.8, 128.1, 126.4, 114.0, 113.9, 111.6, 55.3, 55.3, 44.8, 43.9, 38.6, 37.5, 31.8, 30.0, 27.7, 27.1, 24.2, 17.5, 12.0. HRMS ($APCI^+$) m/z calcd for $C_{27}H_{35}OSi$, $[M+H]^+$ 403.2457, found: 403.2455. GC retention time, *Z*: 17.215 min; *E*: not found.

(*Z*)-diphenyl(3-phenylbut-2-en-1-yl)silane (3s)

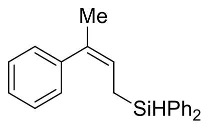

The title compound was isolated (145 mg, 0.460 mmol, 92%) as a colorless oil after chromatography on silica with ethyl acetate/hexane (1:10). ^1H NMR (400 MHz, $CDCl_3$) δ 7.55–7.48 (m, 4H), 7.45–7.39 (m, 2H), 7.39–7.33 (m, 4H), 7.33–7.28 (m, 2H), 7.25–7.20 (m, 1H), 7.07 (m, 2H), 5.58 (td, *J* = 8.3, 1.4 Hz, 1H), 4.88 (t, *J* = 3.5 Hz, 1H), 2.09–2.05 (m, 2H), 2.00 (d, *J* = 1.3 Hz, 3H); $^{13}C\{^1H\}$ NMR (101 MHz, $CDCl_3$) δ 142.2, 136.2, 135.4, 134.0, 129.8, 128.3, 128.2, 128.1, 126.4, 121.5, 25.9, 15.2. HRMS ($APCI^+$) m/z calcd for $C_{22}H_{23}Si$, $[M+H]^+$ 315.1569, found: 315.1567. GC retention time, *Z*: 10.433 min; *E*: 10.957 min.

(*Z*)-methyl(phenyl)(3-phenylbut-2-en-1-yl)silane (3t)

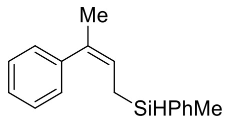

The title compound was isolated (111 mg, 0.440 mmol, 88%) as a colorless oil after chromatography on silica with ethyl acetate/hexane (1:10). ^1H NMR (400 MHz, $CDCl_3$) δ 7.45–7.38 (m, 2H), 7.34–7.20 (m, 5H), 7.17–7.12 (m, 1H), 7.05 (m, 2H), 5.46 (td, *J* = 8.4, 1.4 Hz, 1H), 4.35–4.22 (m, 1H), 1.96 (d, *J* = 1.2 Hz, 3H), 1.77–1.64 (m, 2H), 0.26 (d, *J* = 3.7 Hz, 3H); $^{13}C\{^1H\}$ NMR (101 MHz, $CDCl_3$) δ 142.3, 136.0, 135.7, 134.5, 129.5, 128.2, 128.2, 128.0, 126.4, 122.0, 25.9, 16.3, -5.7. HRMS ($APCI^+$) m/z calcd for $C_{17}H_{21}Si$, $[M+H]^+$ 253.1413, found: 253.1405. GC retention time, *Z*: 8.530 min; *E*: not found.

(*Z*)-diethyl(3-phenylbut-2-en-1-yl)silane (3u)

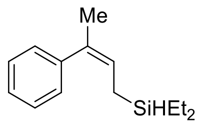

The title compound was isolated (62.2 mg, 0.285 mmol, 57%) as a colorless oil after chromatography on silica with ethyl acetate/hexane (1:20). ^1H NMR (400 MHz, $CDCl_3$) δ 7.38–7.31 (m, 2H), 7.26–7.17 (m, 3H), 5.52 (td, *J* = 8.5, 1.4 Hz, 1H), 3.66 (m, 1H), 2.04 (d, *J* = 1.2 Hz, 3H), 1.54 (m, 2H), 0.95–0.86 (m, 6H), 0.62–0.52 (m, 4H); $^{13}C\{^1H\}$ NMR (101 MHz, $CDCl_3$) δ 142.5, 134.7, 128.3, 128.2, 126.3, 123.0, 25.9, 13.6, 8.2, 2.9. HRMS ($APCI^+$) m/z calcd for $C_{14}H_{23}Si$, $[M+H]^+$ 219.1569, found: 219.1568. GC retention time, *Z*: 7.109 min; *E*: not found.

(*Z*)-diphenyl(3-phenylhex-2-en-1-yl)silane (3v)

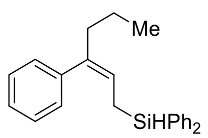

The title compound was isolated (308 mg, 0.899 mmol, 90%) as a colorless oil after chromatography on silica with ethyl acetate/hexane (1:10). ^1H NMR (400 MHz, $CDCl_3$) δ 7.47–7.39 (m, 4H), 7.34–7.25 (m, 6H), 7.20 (m, 2H), 7.16–7.11 (m, 1H), 6.90 (m, 2H), 5.45 (m, 1H), 4.78 (t, *J* = 3.5 Hz, 1H), 2.19 (t, *J* = 7.3 Hz, 2H), 1.94 (m, 2H), 1.18 (m, 2H), 0.75 (t, *J* = 7.3 Hz, 3H); $^{13}C\{^1H\}$ NMR (101 MHz, $CDCl_3$) δ 141.0, 136.0, 135.4, 134.1, 129.7, 128.7, 128.1, 128.0, 126.3, 121.3, 41.8, 21.4, 15.1, 13.7. HRMS ($APCI^+$) m/z calcd for $C_{24}H_{27}Si$, $[M+H]^+$ 343.1882, found: 343.1876. GC retention time, *Z*: 10.910 min; *E*: 11.270 min.

(3-cyclopropyl-3-phenylallyl)(phenyl)silane (3w)

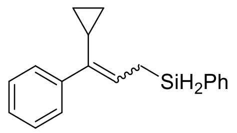

Z:*E*= 63:37

The title compound was isolated (59.5 mg, 0.225 mmol, 45%) as a colorless oil after chromatography on silica with ethyl acetate/hexane (1:20). ^{1}H NMR (400 MHz, $CDCl_3$) δ 7.64–7.60 (m, 0.73H), 7.50–7.46 (m, 1.23H), 7.43–7.26 (m, 5.44H), 7.25–7.16 (m, 1.38H), 7.03 (m, 1.22H), 5.83 (*E*: td, *J* = 8.6, 1.8 Hz, 0.37H), 5.52 (*Z*: td, *J* = 8.4, 0.9 Hz, 0.62H), 4.40 (*E*: t, *J* = 3.7 Hz, 0.69H), 4.24 (*Z*: t, *J* = 3.7 Hz, 1.20H), 2.24 (*E*: m, 0.74H), 1.75–1.68 (*Z*: m, 1.26H), 1.58 (*Z*: s, 0.63H), 1.27 (*E*: s, 0.37H), 0.76 (*E*: m, 0.74H), 0.61–0.55 (*Z*: m, 1.27H), 0.40–0.32 (*Z*: m, 1.24H), 0.26 (*E*: m, 0.75H); ^{13}C{^{1}H} NMR (101 MHz, $CDCl_3$) δ 142.5, 134.7, 128.3, 128.2, 126.3, 123.0, 25.9, 13.6, 8.2, 2.9. HRMS (APCI^{+}) m/z calcd for $C_{18}H_{21}Si$, $[M+H]^+$ 265.1413, found: 265.1410. GC retention time, *Z*: 9.145 min; *E*: 9.410min.

Hydrosilylation Reactions on 10.0 mmol Scale with Catalysts Weighted in Air

To a 50 mL Schlenk flask was added Co(acac)$_2$ (12.8 mg, 0.050 mmol) and Xantphos (29.0 mg, 0.050 mmol), pre-weighed on the open bench. The flask was back-filled with Ar three times and dry THF (10 mL) was added to give a pale pink solution. After stirring for 5 min, buta-2,3-dien-2-ylbenzene (1.30 g, 10.0 mmol) was added with air-tight syringe, followed by phenylsilane (1.19 g, 11.0 mmol) resulting in a pale yellow solution which turns dark after vigorous stirring. The reaction mixture was stirred at r.t. for 10 h and then the resulting solution was concentrated in vacuum. The crude product was purified by column chromatography on silica gel with hexane, yielding the product **3a** (2.12 g, 8.89 mmol, 89%) as a colorless oil.

To a 50 mL Schlenk flask was added Co(acac)$_2$ (12.8 mg, 0.050 mmol) and Xantphos (29.0 mg, 0.050 mmol), pre-weighed on the open bench. The flask was back-filled with Ar three times and dry THF (10 mL) was added to give a pale pink solution. After stirring for 5 min, buta-2,3-dien-2-ylbenzene (1.30 g, 10.0 mmol) was added with air-tight syringe, followed by diphenylsilane (2.03 g, 11.0 mmol). The reaction mixture was stirred at r.t. for 10 h and then the resulting solution was concentrated in vacuum. The crude product was purified by column chromatography on silica gel with hexane, yielding the product **3s** (2.99 g, 9.51 mmol, 95%) as a colorless oil.

Intramolecular Dehydrogenative Silylation of 3v

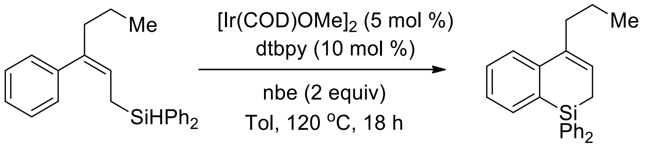

1,1-diphenyl-4-propyl-1,2-dihydrobenzo[b]siline (4)

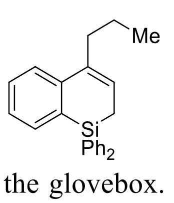

In an Ar-filled glovebox, a 4-mL screw-capped vial was charged with (1,5-Cyclooctadiene)(methoxy)iridium(I) dimer (16.6 mg, 25.0 μmol), 4,4-di-tert-butyl bipyridine (17.8 mg, 50.0 μmol), (*Z*)-diphenyl(3-phenylhex-2-en-1-yl)silane (171 mg, 0.500 mmol), 2-norbornene (94.3 mg, 1.000 mmol), toluene (1.0 mL) and a magnetic stirring bar. The vial was sealed with a cap containing a PTFE septum and removed from the glovebox. The reaction mixture was stirred for 18 h at 120 ºC after which, the solvent was removed under reduced pressure. The title compound was isolated (86.8 mg, 0.255 mmol, 51%) as a colourless oil after chromatography on silica gel with ethyl acetate/hexane (1:20). ^{1}H NMR (400 MHz, $CDCl_3$) δ 7.53 (m, 3H), 7.45–7.38 (m, 5H), 7.34 (m, 5H), 7.16 (m, 1H), 5.97 (t, *J* = 5.8 Hz, 1H), 2.44 (t, *J* = 7.3 Hz, 2H), 2.06 (d, *J* = 5.8 Hz, 2H), 1.41 (m, 2H), 0.74 (t, *J* = 7.4 Hz, 3H); ^{13}C{^{1}H} NMR (101 MHz, $CDCl_3$) δ 143.8, 138.6, 135.8, 135.2, 134.3, 131.7, 130.3, 129.7, 127.9, 126.2, 124.9, 123.0, 38.5, 22.1, 13.8, 11.5. HRMS (APCI^{+}) m/z calcd for $C_{24}H_{25}Si$, $[M+H]^+$ 341.1726, found: 341.1723.

One-pot Synthesis of (*Z*)-allylic alcohols

According to a modification of the procedure described by Tamao[14] and Buchwald[15], in an Ar-filled dry box, $Co(acac)_2$, binap or xantphos, and THF (1mL) were added to a 10-mL screw-capped vial and stirred for 5 mins. Then terminal allenes (0.500 mmol) and $PhSiH_3$ (1.1 eq, 0.550 mmol) were added. The vial was sealed with a cap containing a PTFE septum and removed from the dry box. The reaction mixture was stirred at room temperature for 18 h or 3h, then $Na_2EDTA\cdot(H_2O)_2$ (74.4 mg, 0.200 mmol), $KHCO_3$ (100 mg, 1.00 mmol), and THF (1 mL) was added to the resulting mixture. The resulting suspension was stirred at rt for 30 min, followed by adding MeOH (2 mL) and H_2O_2 (1.02 mL, 9 mmol). The mixture was stirred at room temperature for 20 h before being quenched by water (4 mL). The organic layer was separated and the aqueous layer was extracted with Et_2O (4 mL × 2). The combined organic phase was washed with brine (5 mL), and dried over anhydrous Na_2SO_4. Solvent was removed under reduced pressure and column chromatography on silica gel afforded the corresponding (*Z*)-allylic alcohols.

(*Z*)-3-phenylbut-2-en-1-ol (5a)

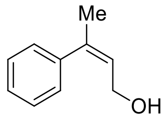

The title compound was isolated (62.2 mg, 0.420 mmol, 84%) as a colorless oil after chromatography on silica with ethyl acetate/hexane (1:20). ^{1}H NMR (400 MHz, $CDCl_3$) δ 7.30–7.23 (m, 2H), 7.20 (m, 1H), 7.13–7.07 (m, 2H), 5.64 (td, *J* = 7.0, 1.4 Hz, 1H), 4.00 (m, 2H), 2.02 (d, *J* = 1.2 Hz, 3H), 1.24 (s, 1H); $^{13}C\{^{1}H\}$ NMR (101 MHz, $CDCl_3$) δ 140.9, 140.4, 128.3, 127.9, 127.3, 126.2, 60.5, 25.5. This compound was known.[16] GC retention time, *Z*: 6.115 min; *E*: 6.280 min.

(*Z*)-3-(2-methoxyphenyl)but-2-en-1-ol (5b)

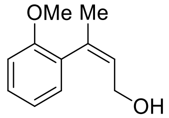

The title compound was isolated (57.0 mg, 0.320 mmol, 64%) as a colorless oil after chromatography on silica with ethyl acetate/hexane (1:20). ^{1}H NMR (400 MHz, $CDCl_3$) δ 7.28–7.23 (m, 1H), 7.04 (m, 1H), 6.97–6.90 (m, 2H), 5.80 (td, *J* = 7.2, 1.4 Hz, 1H), 3.85 (d, *J* = 5.5 Hz, 2H), 3.81 (s, 3H), 2.04 (d, *J* = 0.4 Hz, 3H), 1.63 (s, 1H); $^{13}C\{^{1}H\}$ NMR (101 MHz, $CDCl_3$) δ 156.1, 137.7, 130.0, 129.7, 128.6, 127.0, 120.8, 111.2, 60.8, 55.7, 25.0. GC-MS (EI): Calcd for $C_{11}H_{14}O_2$: 178.1, found: 178.1. GC retention time, *Z*: 6.930 min; *E*: 7.110 min.

(*Z*)-3-(benzo[d][1,3]dioxol-5-yl)but-2-en-1-ol (5c)

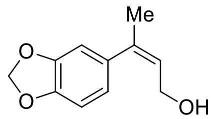

The title compound was isolated (79.8 mg, 0.415 mmol, 83%) as a colorless oil after chromatography on silica with ethyl acetate/hexane (1:20). ^{1}H NMR (400 MHz, $CDCl_3$) δ 6.78 (d, *J* = 7.9 Hz, 1H), 6.69 (d, *J* = 1.6 Hz, 1H), 6.64 (m, 1H), 5.96 (s, 2H), 5.66 (td, *J* = 7.0, 1.4 Hz, 1H), 4.12–4.06 (m, 2H), 2.05 (d, *J* = 1.1 Hz, 3H), 1.31 (s, 1H); $^{13}C\{^{1}H\}$ NMR (101 MHz, $CDCl_3$) δ 147.6, 146.8, 140.0, 134.8, 126.1, 121.3, 108.5, 108.2, 101.2, 60.5, 25.6. GC-MS (EI): Calcd for $C_{11}H_{12}O_3$: 192.1, found: 192.1. GC retention time, *Z*: 7.870 min; *E*: not found.

(*Z*)-3-(4-(trifluoromethyl)phenyl)but-2-en-1-ol (5d)

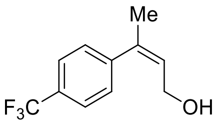

The title compound was isolated (73.4 mg, 0.340 mmol, 68%) as a colorless oil after chromatography on silica with ethyl acetate/hexane (1:20). ^{1}H NMR (400 MHz, $CDCl_3$) δ 7.60 (m 2H), 7.30 (d, *J* = 7.9 Hz, 2H), 5.79 (m 1H), 4.04 (dd, *J* = 7.1, 1.0 Hz, 2H), 2.10 (dd, *J* = 2.4, 1.1 Hz, 3H), 1.36 (s, 1H); $^{13}C\{^{1}H\}$ NMR (101 MHz, $CDCl_3$) δ 144.6, 139.1, 129.5 (q, *J* = 32.6 Hz), 128.3, 127.5, 125.3 (q, *J* = 3.8 Hz), 124.3 (q, *J* = 273 Hz), 60.1, 25.2. GC-MS (EI): Calcd for $C_{11}H_{11}F_3O$: 216.1, found: 216.1. GC retention time, *Z*: 5.985 min; *E*: not found.

(*Z*)-3-(naphthalen-2-yl)but-2-en-1-ol (5e)

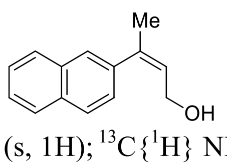

The title compound was isolated (64.4 mg, 0.325 mmol, 65%) as a colorless oil after chromatography on silica with ethyl acetate/hexane (1:20). ^{1}H NMR (400 MHz, $CDCl_3$) δ 7.83 (m 3H), 7.62 (d, J = 1.0 Hz, 1H), 7.52–7.44 (m, 2H), 7.33 (m 1H), 5.87–5.74 (m, 1H), 4.14 (dd, J = 7.0, 0.7 Hz, 2H), 2.18 (dd, J = 2.4, 1.1 Hz, 3H), 1.38 (s, 1H); ^{13}C{^{1}H} NMR (101 MHz, $CDCl_3$) δ 140.3, 138.4, 133.3, 132.7, 128.1, 127.9, 127.8, 126.7, 126.6, 126.3, 126.2, 126.0, 60.5, 25.5. GC-MS (EI): Calcd for $C_{14}H_{14}O$: 198.1, found: 198.1. GC retention time, *Z*: 8.700 min; *E*: not found.

(*Z*)-3-cyclohexylprop-2-en-1-ol (5f)

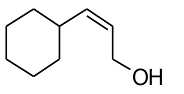

The title compound was isolated (58.9 mg, 0.420 mmol, 84%) as a colorless oil after chromatography on silica with ethyl acetate/hexane (1:20). ^{1}H NMR (400 MHz, $CDCl_3$) δ 5.42 (dt, J = 11.3, 6.6 Hz, 1H), 5.32 (t, J = 10.2 Hz, 1H), 4.13 (d, J = 6.6 Hz, 2H), 2.28–2.11 (m, 1H), 1.66–1.61 (m, 2H), 1.60–1.48 (m, 3H), 1.34 (s, 1H), 1.27–1.09 (m, 3H), 1.01 (m, 2H); ^{13}C{^{1}H} NMR (101 MHz, $CDCl_3$) δ 139.3, 126.6, 58.9, 36.7, 33.5, 26.0, 25.9. This compound was known.[17] GC retention time, *Z*: 5.605 min; *E*: not found.

(*Z*)-3-cyclohexylbut-2-en-1-ol (5g)

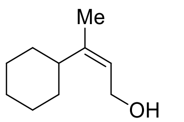

The title compound was isolated (54.7 mg, 0.355 mmol, 71%) as a colorless oil after chromatography on silica with ethyl acetate/hexane (1:20). ^{1}H NMR (400 MHz, $CDCl_3$) δ 5.33 (td, J = 7.0, 1.1 Hz, 1H), 4.15 (d, J = 6.3 Hz, 2H), 2.43 (m 1H), 1.78–1.72 (m, 2H), 1.66 (s, 4H), 1.47 (d, J = 8.2 Hz, 2H), 1.35–1.26 (m, 4H), 1.17 (m 2H); ^{13}C{^{1}H} NMR (101 MHz, $CDCl_3$) δ 145.4, 123.3, 58.7, 40.1, 31.5, 26.6, 26.3, 19.8. This compound was known.[18] GC retention time, *Z*: 6.180 min; *E*: 6.290 min.

(*Z*)-3-(furan-2-yl)but-2-en-1-ol (5h)

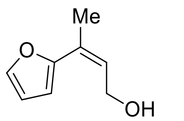

The title compound was isolated (47.0 mg, 0.340 mmol, 68%) as a colorless oil after chromatography on silica with ethyl acetate/hexane (1:20). ^{1}H NMR (400 MHz, $CDCl_3$) δ 7.43 (d, J = 1.5 Hz, 1H), 6.42 (m, 1H), 6.31 (d, J = 3.3 Hz, 1H), 5.63 (td, J = 6.5, 1.4 Hz, 1H), 4.50 (m, 2H), 2.04 (d, J = 1.2 Hz, 3H), 1.64 (s, 1H); ^{13}C{^{1}H} NMR (101 MHz, $CDCl_3$) δ 154.3, 142.3, 127.1, 126.2, 111.2, 109.0, 60.5, 21.5. This compound was known.[19] GC retention time, *Z*: 5.740 min; *E*: not found.

Isomerization Studies

Procedure for isomerization of **(Z)-1l**

Condition **A**: In an Ar-filled dry box, $Co(acac)_2$ (2.6 mg, 10.0 μmol), rac-binap (6.2 mg, 10.0 μmol) and THF(1 mL) were added to a 4-mL screw-capped vial and stirred for 5 mins. Then **(Z)-1l** (0.500 mmol) was added. The vial was sealed with a cap containing a PTFE septum and removed from the dry box. The reaction mixture was stirred at room temperature for 24 h. The *Z*/*E* ratio (*Z*/*E* = 99:1) was determined by GC analysis.

Condition **B**: In an Ar-filled dry box, $Co(acac)_2$ (1.3 mg, 5.0 μmol), xantphos (2.9 mg, 5.0 μmol) and THF(1 mL) were added to a 4-mL screw-capped vial and stirred for 5 mins. Then **(Z)-1l** (0.500 mmol) was added. The vial was sealed with a cap containing a PTFE septum and removed from the dry box. The reaction mixture was stirred at room temperature for 24 h. The *Z*/*E* ratio (*Z*/*E* = 5:95) was determined by GC analysis. The **(E)-1l** was isolated by column chromatography on silica gel.

(*E*)-cinnamyl(phenyl)silane ((*E*)-1l)

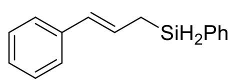

The title compound was isolated as a colorless oil after chromatography on silica with ethyl acetate/hexane (1:20). ^{1}H NMR (400 MHz, $CDCl_3$) δ 7.62 (m, 2H), 7.46–7.36 (m, 3H), 7.34–7.27 (m, 4H), 7.23–7.16 (m, 1H), 6.40–6.24 (m, 2H), 4.40 (t, J = 3.7 Hz, 2H), 2.10 (dt, J = 7.2, 3.7 Hz, 2H); $^{13}C\{^{1}H\}$ NMR (101 MHz, $CDCl_3$) δ 138.1, 135.4, 131.8, 130.2, 130.0, 128.6, 128.2, 126.8, 126.3, 125.8, 17.0. HRMS ($APCI^+$) m/z calcd for $C_{15}H_{17}Si$, $[M+H]^+$ 225.1100, found: 225.1098.

Procedure for mercury test

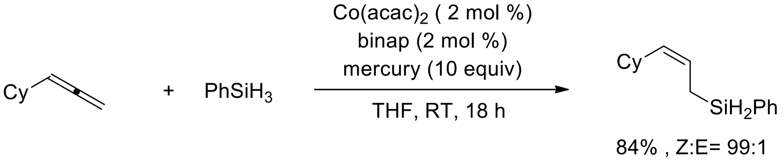

In an Ar-filled dry box, $Co(acac)_2$ (2.6 mg, 10.0 μmol), rac-binap (6.2 mg, 10.0 μmol) and THF (1mL) were added to a 4-mL screw-capped vial and stirred for 5 mins. Then cyclohexylallene (0.500 mmol), Hg (1.00 g, 5.00 mmol) and $PhSiH_3$ (61.2 mg, 1.1 eq, 0.550 mmol) were added. The vial was sealed with a cap containing a PTFE septum and removed from the dry box. The reaction mixture was stirred at room temperature for 18 h and the product was isolated by column chromatography on silica gel in 84% yield.

Procedure for Deuterium-labeling Experiments

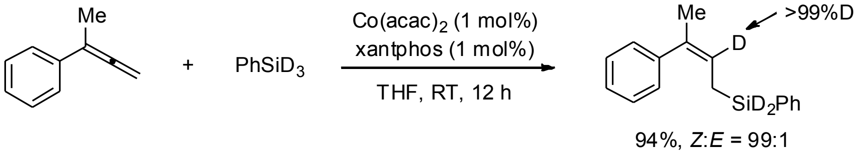

In an Ar-filled dry box, $Co(acac)_2$ (1.3 mg, 5.0 μmol), xantphos (2.9 mg, 5.0 μmol) and THF (1mL) were added to a 4-mL screw-capped vial and stirred for 5 mins. Then buta-2,3-dien-2-ylbenzene (0.500 mmol) and $PhSiD_3$ (61.2 mg, 1.1 eq, 0.550 mmol) were added. The vial was sealed with a cap containing a PTFE septum and removed from the dry box. The reaction mixture was stirred at room temperature for 12 h and the resulting solution was concentrated in vacuum. The crude product was purified by column chromatography on silica gel with a mixture of ethyl acetate and hexane as eluent, yielding the product as a colorless oil (113 mg, 0.468 mmol, 94%). The ^{2}H NMR spectroscopy was measured with C_6D_6 (1 equiv) as an internal standard.

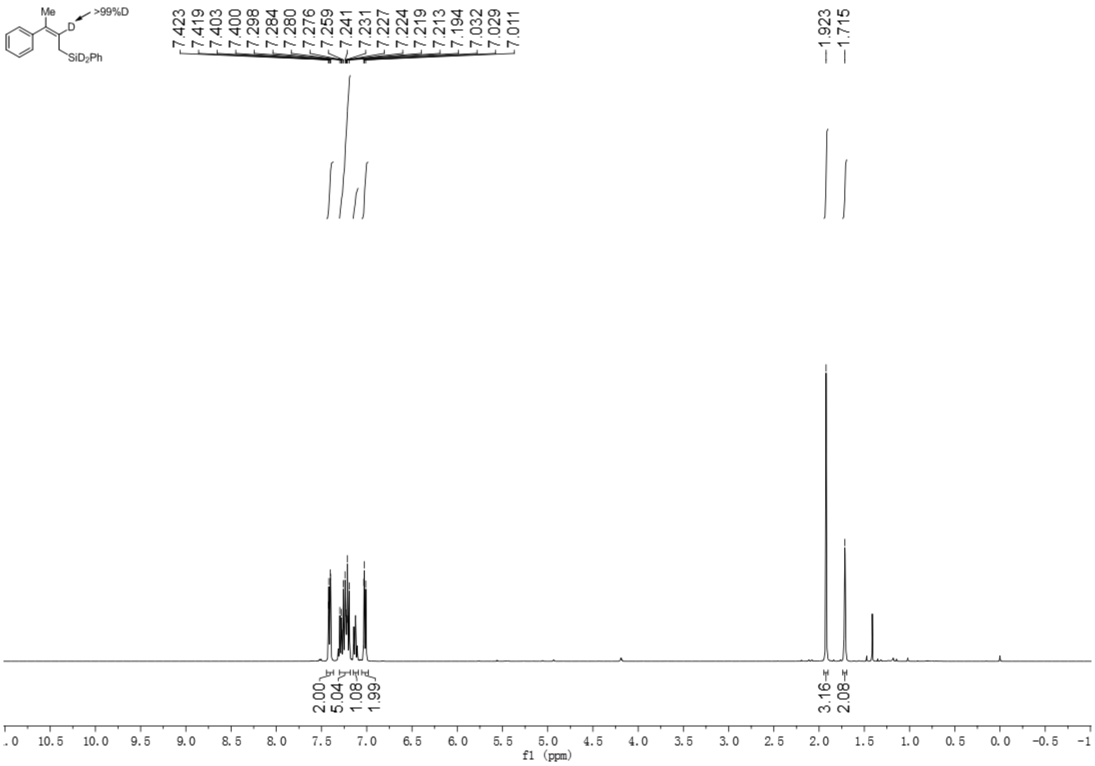

Supplementary Figure 118. ^{1}H NMR spectra for deuterated 3a

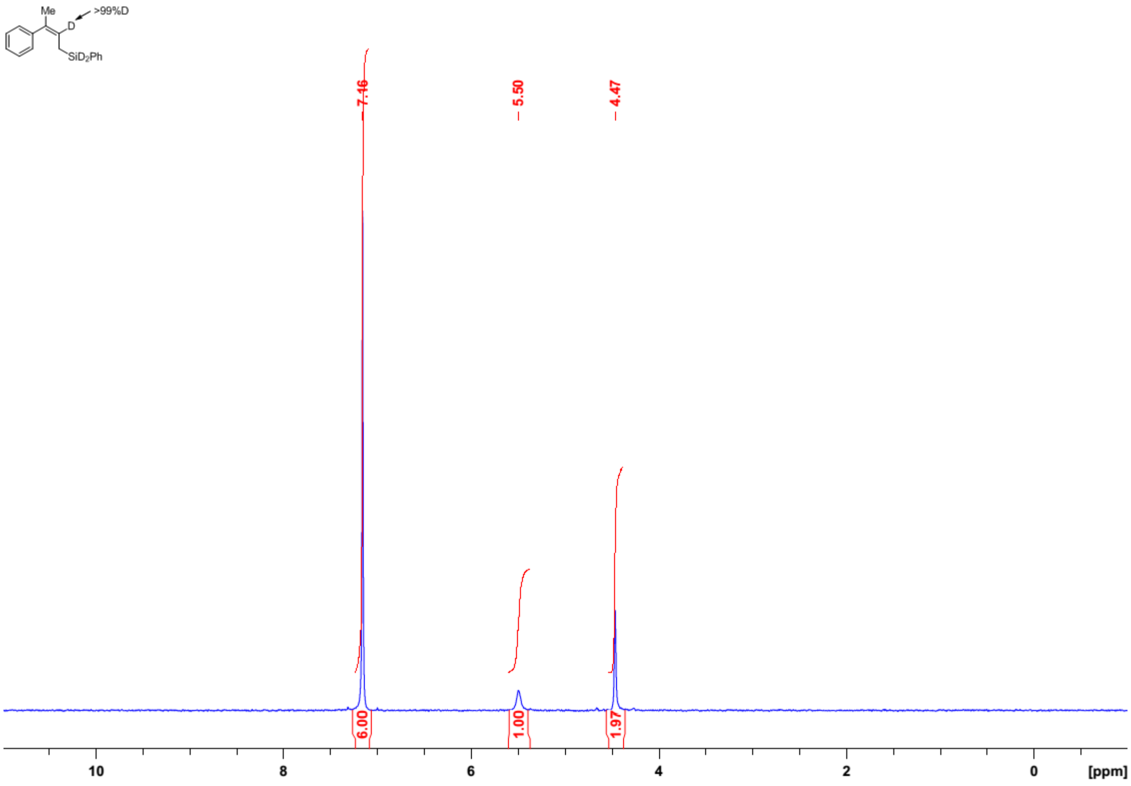

Supplementary Figure 119. ^{2}H NMR spectra for deuterated 3a

Synthesis of $(dppbz)_2CoH$ and Its reactivity towards the Hydrosilylation of buta-2,3-dien-2-ylbenzene

$$2\ \text{dppbz} + \text{Co(acac)}_2 + 8\ \text{PhSiH}_3 \xrightarrow[\text{RT, 12 h}]{\text{THF}} (\text{dppbz})_2\text{CoH}$$

isolated in 70%

Supplementary Equation 1. Synthesis of $(dppbz)_2CoH$

$Co(acac)_2$ (0.30 mmol) and dppbz (0.6 mmol) were dissolved in THF (1.0 mL) in a screw-capped vial under Ar atmosphere, to which was added $PhSiH_3$ (2.4 mmol). The mixture was stirred at RT for 12 h, which resulted in the formation of a red precipitate. The precipitate was collected by filtration and washed with pentane affording the $(dppbz)_2Co$-H complex in 70% yield. ^1H NMR (400 MHz, THF-*d*8) δ 7.18 (s, 4H), 7.10–6.91 (m, 28H), 6.83 (t, J = 7.5 Hz, 16H), -14.47 (p, J = 22.9 Hz, 1H); ^{31}P NMR (162 MHz, THF-*d*8) δ 73.2.

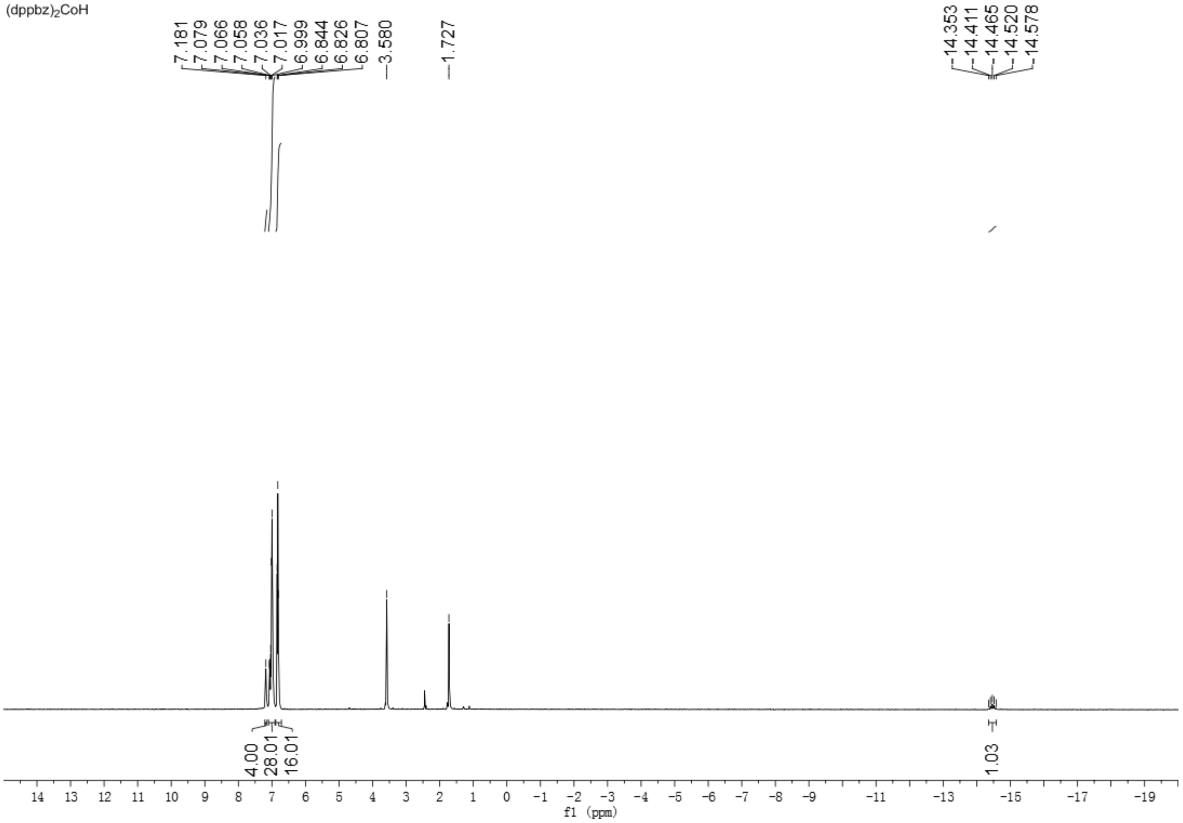

Supplementary Figure 120. ^1H NMR spectra for compound 6

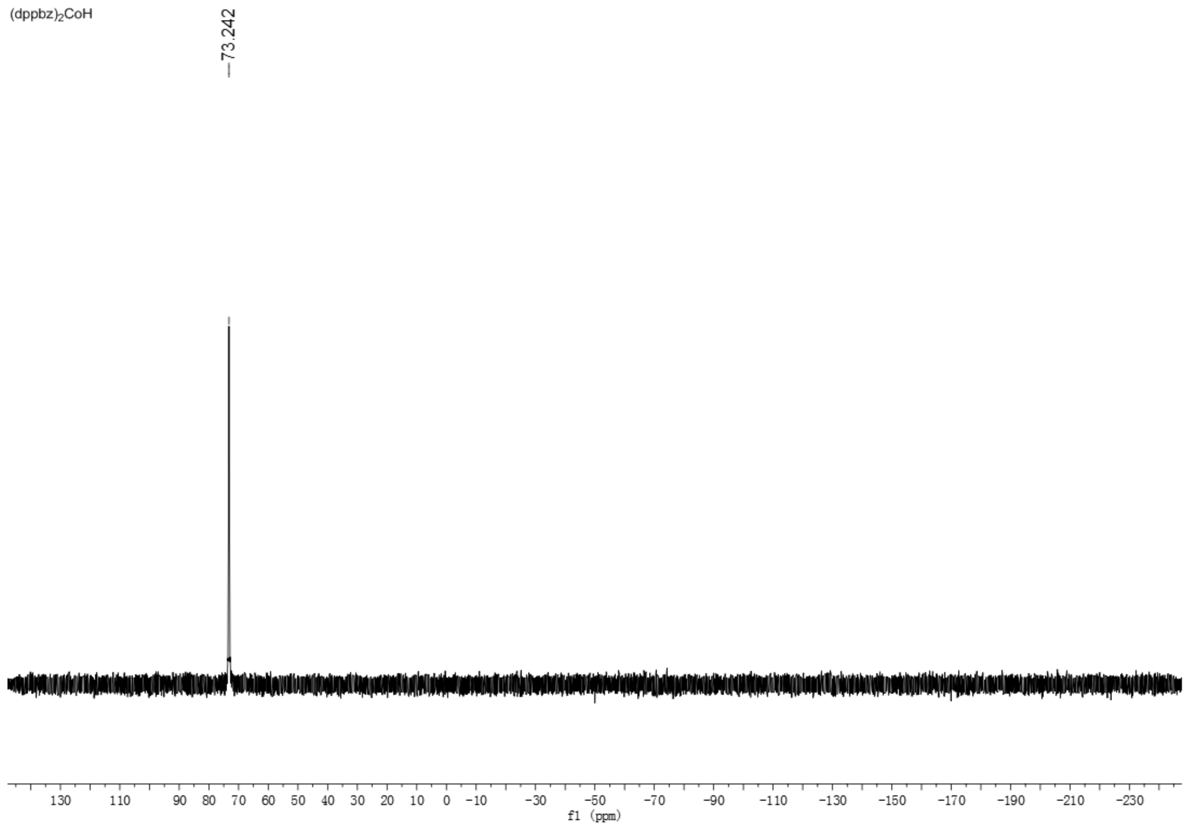

Supplementary Figure 121. ^{31}P NMR spectra for compound 6

Supplementary References

1 Britovsek, G. J. P. *et al.* Iron and Cobalt Ethylene Polymerization Catalysts Bearing 2,6-Bis(Imino)Pyridyl Ligands: Synthesis, Structures, and Polymerization Studies. *J. Am. Chem. Soc.* **121**, 8728-8740 (1999).

2 Li, C. Kahny, M. & Breit, B. Rhodium-Catalyzed Chemo-, Regio-, and Enantioselective Addition of 2-Pyridones to Terminal Allenes. *Angew. Chem. Int. Ed.* **53**, 13780-13784 (2014).

3 Baird, M. S. Nizovtsev, A. V. & Bolesov, I. G. Bromine–magnesium exchange in *gem*-dibromocyclopropanes using Grignard reagents. *Tetrahedron* **58**, 1581-1593 (2002).

4 Miller, Z. D. Li, W. Belderrain, T. R. & Montgomery, J. Regioselective Allene Hydrosilylation Catalyzed by N-Heterocyclic Carbene Complexes of Nickel and Palladium. *J. Am. Chem. Soc.* **135**, 15282−15285 (2013).

5 Trost, B. M. Pinkerton, A. B. & Seidel, M. Ruthenium-Catalyzed Two-Component Addition To Form 1,3-Dienes: Optimization, Scope, Applications, and Mechanism. *J. Am. Chem. Soc.* **123**, 12466−12476 (2001).

6 Lin, T.-Y. Zhu, C.-Z. Zhang, P. Wang, Y. Wu, H.-H. Feng, J.-J. & Zhang, J. Regiodivergent Intermolecular [3+2] Cycloadditions of Vinyl Aziridines and Allenes: Stereospecific Synthesis of Chiral Pyrrolidines. *Angew. Chem. Int. Ed.* **55**, 10844-10848 (2016).

7 Liu, J. Nie, M. Zhou, Q. Gao, S. Jiang, W. Chung, L. W. Tang, W. & Ding, K. Enantioselective palladium-catalyzed diboration of 1,1-disubstituted allenes. *Chem. Sci.* **8**, 5161-5165 (2007).

8 Jayanth, T. T., Jeganmohan, M., Cheng, M.-J., Chu, S.-Y. & Cheng, C.-H. Ene Reaction of Arynes with Alkynes. *J. Am. Chem. Soc.* **128**, 2232−2233 (2006).

9 Zhou, C., Li, J., Lu, B., Fu, C. & Ma, S. An Efficient Approach for Monofluorination via Highly Regioselective Fluorohydroxylation Reaction of 3-Aryl-1,2-allenes with Selectfluor. *Org. Lett.*, **10**, 581–583 (2008).
10 Ando, T. & Tokura, N. Benzylation of Acetylene. I. Formation of 3,4-Diphenyl-1-butyne and 3,4-Diphenyl-1,2-butadiene. *Bulletin of the Chemical Society of Japan* **30**, 259-263 (1957).
11 Kuang, J. & Ma, S. An Efficient Synthesis of Terminal Allenes from Terminal 1-Alkynes. *J. Org. Chem.* **74**, 1763-1765 (1999).
12 Kobayashi, K., Naka, H., Wheatley, A. E. H. & Kondo, Y. Organozinc Reagents in DMSO Solvent: Remarkable Promotion of SN2′ Reaction for Allene Synthesis. *Org. Lett.* **10**, 3375-3377 (2008).
13 Tani, Y., Fujihara, T., Terao, J. & Tsuji, Y. Copper-Catalyzed Regiodivergent Silacarboxylation of Allenes with Carbon Dioxide and a Silylborane. *J. Am. Chem. Soc.* **136**, 17706 (2014).
14 Tamao, K. & Ishida, N. Silafunctional compounds in organic synthesis: XXVI. Silyl groups synthetically equivalent to the hydroxy group. *J. Organomet. Chem.* **269**, c37-c39 (1984).
15 Gribble, M. W., Pirnot, M. T., Bandar, J. S., Liu, R. Y. & Buchwald, S. L. Asymmetric Copper Hydride-Catalyzed Markovnikov Hydrosilylation of Vinylarenes and Vinyl Heterocycles. *J. Am. Chem. Soc.* **139**, 2192-2195 (2017).
16 Morrill, C., Beutner, G. L. & Grubbs, R. H. Rhenium-Catalyzed 1,3-Isomerization of Allylic Alcohols: Scope and Chirality Transfer. *J. Org. Chem.* **71**, 7813-7825 (2006).
17 Dong, D.-J., Li, Y., Wang, J.-Q. & Tian, S.-K. Tunable stereoselective alkene synthesis by treatment of activated imines with nonstabilized phosphonium ylides. *Chem. commun.* **47**, 2158-2160 (2011).
18 Mantilli, L., Gerard, D., Torche, S., Besnard, C. & Mazet, C. Iridium-Catalyzed Asymmetric Isomerization of Primary Allylic Alcohols. *Angew. Chem. Int. Ed.* **48**, 5143-5147 (2009).
19 Wada, A. *et al.* Preparation and Biological Activities of Heteroarotinoids. *Letters in Drug Design & Discovery*, **4**, 442-445 (2007).
